# Total Synthesis of the *Schisandraceae* Nortriterpenoid Rubriflordilactone A

**DOI:** 10.1002/chem.201703229

**Published:** 2017-09-08

**Authors:** Guilhem Chaubet, Shermin S. Goh, Mujahid Mohammad, Birgit Gockel, Marie‐Caroline A. Cordonnier, Hannah Baars, Andrew W. Phillips, Edward A. Anderson

**Affiliations:** ^1^ Chemistry Research Laboratory University of Oxford 12 Mansfield Road Oxford OX1 3TA UK; ^2^ Institute of Organic Chemistry RWTH Aachen University Landoltweg 1 52074 Aachen Germany

**Keywords:** cascade cyclization, cyclotrimerization, natural products, total synthesis, transition-metal catalysis

## Abstract

Full details of the total synthesis of the *Schisandraceae* nortriterpenoid natural product rubriflordilactone A are reported. Palladium‐ and cobalt‐catalyzed polycyclizations were employed as key strategies to construct the central pentasubstituted arene from bromoendiyne and triyne precursors. This required the independent assembly of two AB ring aldehydes for combination with a common diyne component. A number of model systems were explored to investigate these two methodologies, and also to establish routes for the installation of the challenging benzopyran and butenolide rings.

## Introduction

**Figure 1:**
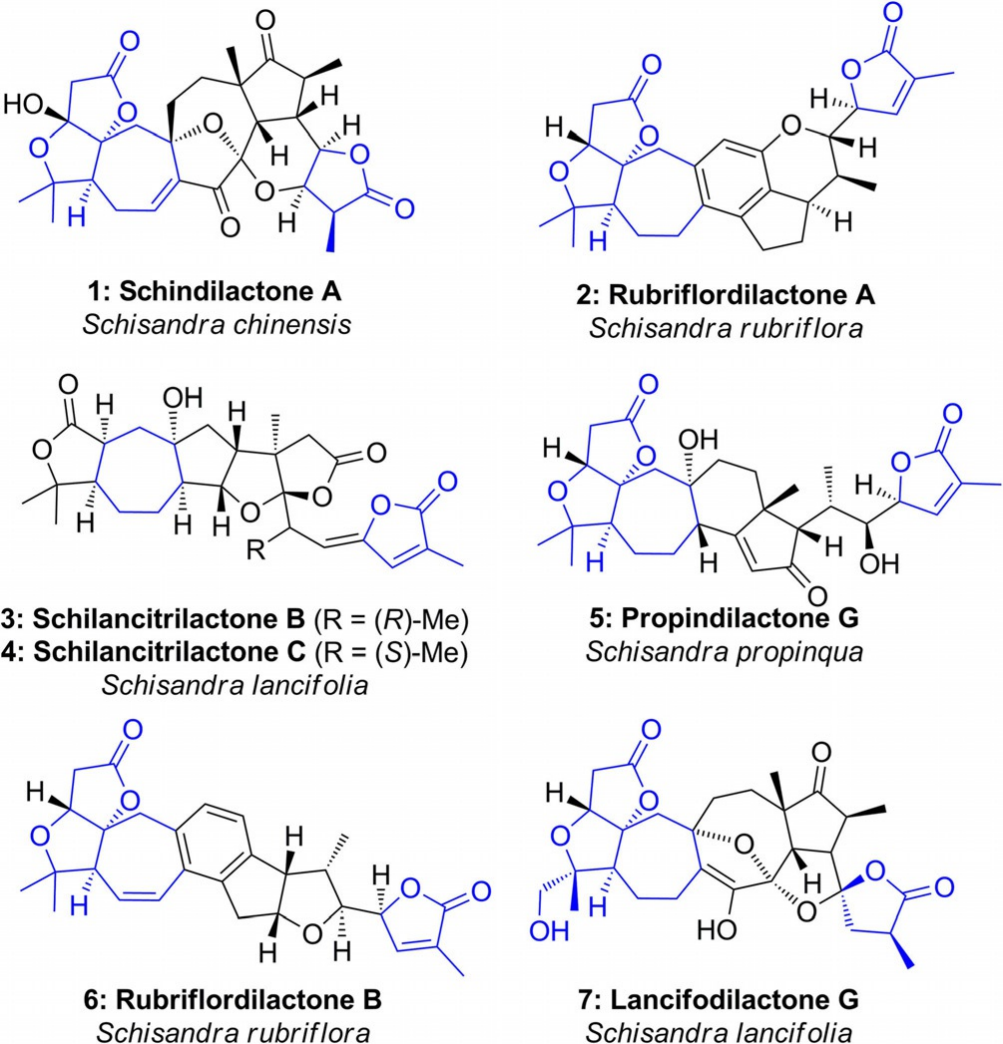
Schinortriterpenoid natural products prepared by total synthesis, and common structural features.

The *Schisandraceae* family of climbing plants are widely distributed throughout east Asia. Extracts of these plants have been employed in traditional medicine for thousands of years, with uses ranging from antihepatitis and anticancer properties, to antioxidant and immune regulatory activity.^1^ Due in part to this ethnopharmacological history, much effort has been dedicated to the characterization of their bioactive constituents, resulting in the isolation of more than 420 triterpenoids from *Schisandraceae* species since 1973.^2^ Around a third of these have been termed “schinortriterpenoids”, which specifically refers to nortriterpenoids isolated from the *Schisandra* genus; a subset of representative structures is depicted in Figure 1. The schinortriterpenoids are thought to derive from the rearrangement of a cycloartane‐type carbon skeleton,^2^ and invariably feature complex polyoxygenated ring systems with numerous stereocenters. Aside from their inherent skeletal complexity, they have attracted more recent attention due to their moderate anti‐HIV activity, coupled with low cytotoxicity.^2^

Following the first isolation of a schinortriterpenoid (micrandilactone A) in 2003,^3^ the first total synthesis of a member of this natural product family was achieved by Yang et al. in 2011 with the synthesis of (±)‐schindilactone A (**1**).^4^ Since then, total syntheses of several other members of this family have been reported, including rubriflordilactone A^5^ (**2**, Li, 2014),^6^ schilancitrilactones B and C (**3**, **4**, Tang, 2015),^7^ propindilactone G (**5**, Yang, 2015),^8^ rubriflordilactone B (**6**, Li, 2016),^9^ and lancifodilactone G (**7**, Yang, 2017).^10^ Despite pronounced structural diversity, several features remain common to most of these natural products, including a fused AB ring system comprising a γ‐lactone and *gem*‐dimethyl substituted tetrahydrofuran, a neighbouring seven‐membered C ring, and an α‐methylated‐γ‐lactone, which is unsaturated in most family members. Among this myriad of synthetically attractive targets,^11^ our group has been particularly interested in the rubriflordilactones,^12^ for which we reported a total synthesis rubriflordilactone A in 2015.^13^ Here we disclose full details of the evolution of our strategy towards this natural product, including the exploration of a number of model systems that provided valuable information in the synthetic campaign, and insight into the scalability of the ultimate synthetic route.

## Results and Discussion

**Scheme 1:**
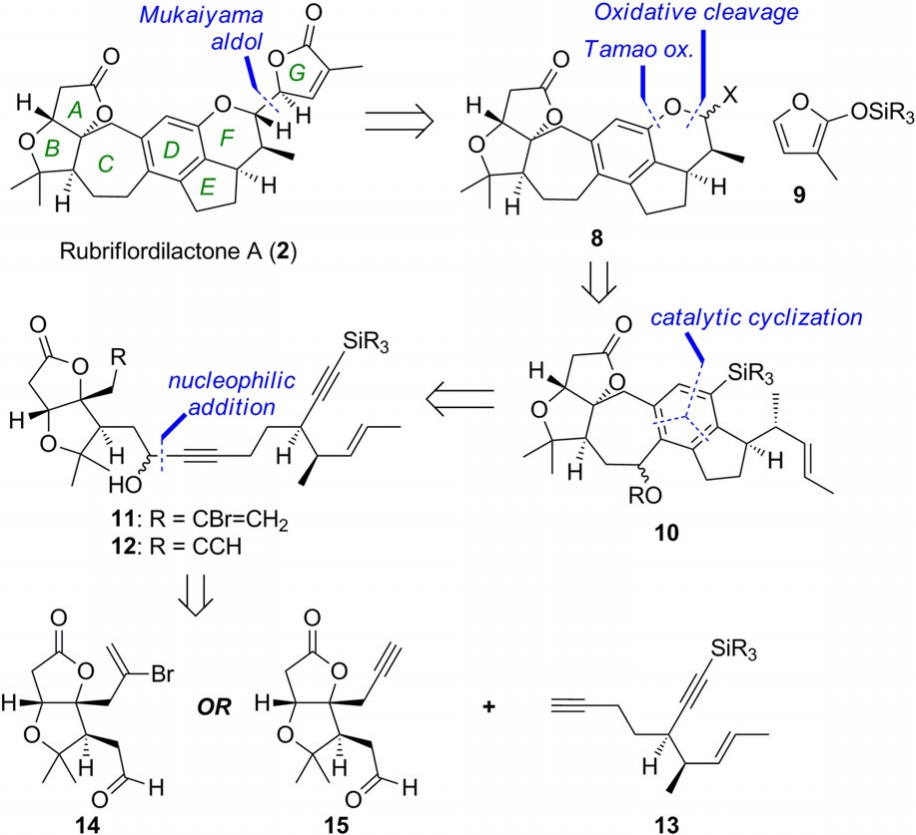
Retrosynthetic analysis of rubriflordilactone A.

From a retrosynthetic perspective (Scheme 1), we planned that the butenolide G ring could be introduced by a late‐stage Mukaiyama aldol‐type addition of silyloxyfuran **9** onto an oxocarbenium ion, which could be formed from an acetal derivative **8**. Following functional group manipulations including F ring formation, pentacycle **10** was further deconstructed via two metal‐catalyzed polycyclization strategies,^14^ involving either palladium‐catalyzed cyclization of bromoendiyne **11**,^15^ or cobalt‐catalyzed cyclotrimerization of triyne **12**,^16^ both of which offered a powerful means to construct the core CDE ring system and its central pentasubstituted arene D ring in a single step. These substrates would be accessed by addition of the diyne **13** to appropriate AB ring aldehydes **14** or **15**. Notably, this strategy neatly segregates the AB ring system, which is common to most schinortriterpenoid natural products, from the rest of the framework, where most variation is found.

**Scheme 2:**
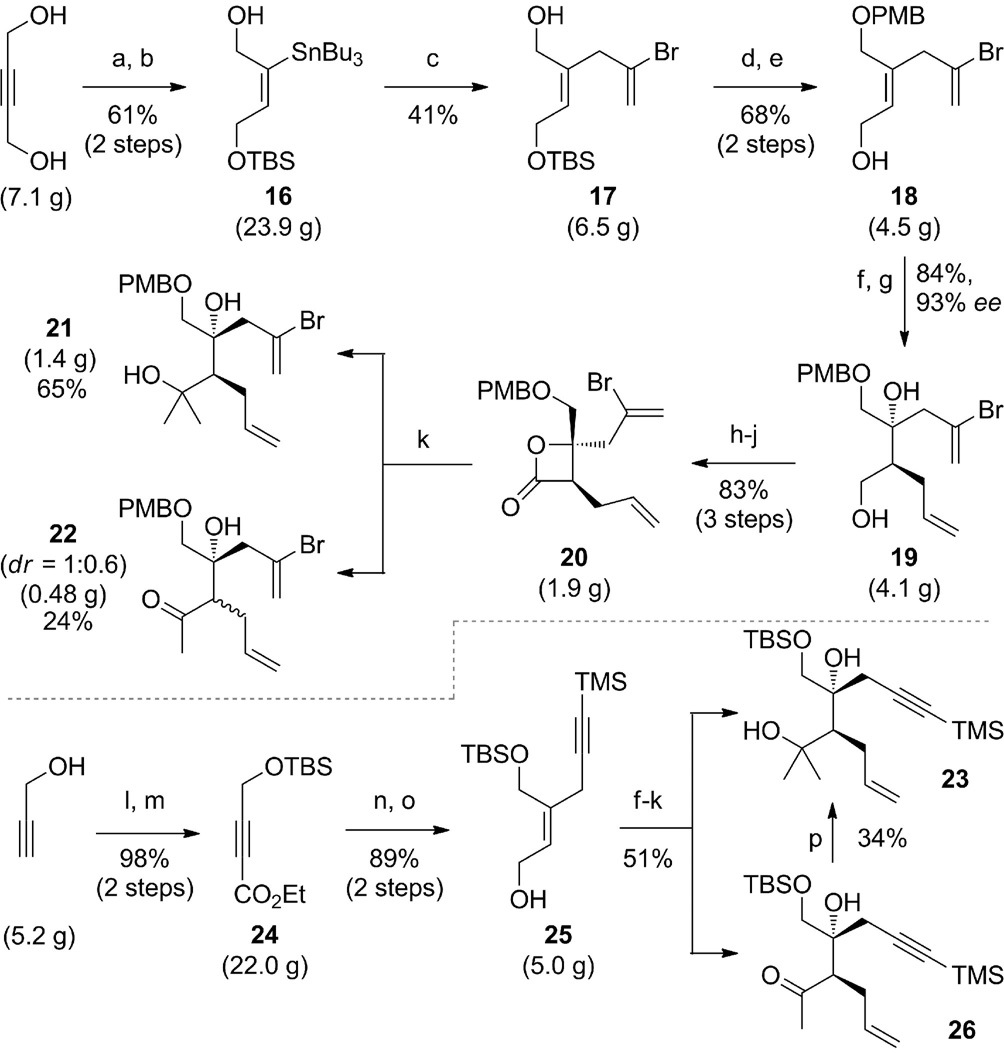
Reagents and conditions for large scale synthesis of **19** and **23**. Masses indicate the scale reactions were conducted on. a) PdCl_2_(PPh_3_)_2_. (3 mol %), Bu_3_SnH, THF; b) TBSCl, imid., DMF, 0 °C to RT; c) Pd(dba)_2_. (4 mol %), 2,3‐dibromopropene, toluene, 70 °C; d) PMBTCA, Sc(OTf)_3_ (5 mol %), PhMe; e) CSA (10 mol %), MeOH; then NEt_3_; f) Ti(O*i*Pr)_4_, d‐(−)‐diethyl tartrate, *t*BuOOH, 4 Å MS, CH_2_Cl_2_, −20 °C; g) allylMgBr, THF, 0 °C; h) Dess–Martin periodinane, NaHCO_3_, CH_2_Cl_2_; i) 2‐methyl‐2‐butene, NaClO_2_, NaH_2_PO_4_, *t*BuOH/H_2_O (2:1); j) BOPCl, py, MeCN; k) MeMgBr, THF −50 °C to RT; l) TBSCl, imid., DMAP, CH_2_Cl_2_; m) *n*BuLi, THF, −78 °C; EtOCOCl, −78 °C to RT; n) TMSC≡CCH_2_MgBr, CuBr⋅SMe_2_, THF, −40 °C; then **24**, THF, −78 °C; o) DIBALH, CH_2_Cl_2_, −78 °C→rt; p) MeMgBr, THF, −5 °C. BOPCl=bis(2‐oxo‐3‐oxazolidinyl) phosphinic chloride; CSA=(±)‐camphorsulfonic acid; dba=dibenzylidene acetone; DIBALH=diisobutylaluminium hydride; DMAP=4‐dimethylamino pyridine; PMB=4‐methoxybenzyl. PMBTCA=4‐methoxybenzyl trichloroacetimidate, TBS=*tert*‐butyldimethylsilyl.

Investigations commenced with the construction of the AB ring system, which would need to bear either a bromoalkene (**14**) or terminal alkyne (**15**) moiety. The former route began with hydrostannylation of butyne‐1,4‐diol,^17^ followed by a regioselective monosilylation (**16**, Scheme 2, yields are given for the largest scales these reactions were performed on). Stille coupling with 2,3‐dibromopropene, followed by a protecting group switch, gave allylic alcohol **18** bearing the requisite bromoalkene side chain. A Sharpless asymmetric epoxidation^18^ allowed the smooth installation of the key AB ring stereocentres, and the resulting epoxide underwent regioselective ring opening^19^ with allylmagnesium chloride to give primary alcohol **19**. Formation of the β‐lactone **20** in three steps enabled the introduction of the B ring *gem*‐dimethyl motif by double addition of methylmagnesium bromide, to generate diol **21**. The formation of significant amounts of byproduct ketone **22** was also observed (see below).

A similar strategy was followed for the synthesis of the analogous alkyne derivative **23**. The route this time started with propargyl alcohol, which was efficiently converted to alkyne **24** in two steps, before undergoing a *syn*‐carbocupration^20^ with a propargylic cuprate reagent^21^ generated in situ from the corresponding Grignard, to give enyne **25** after reduction of the ethyl ester. Compound **25** underwent an equivalent sequence of transformations as described above to give diol **23**, this time with a TMS‐protected alkyne sidechain.

**Figure 2:**
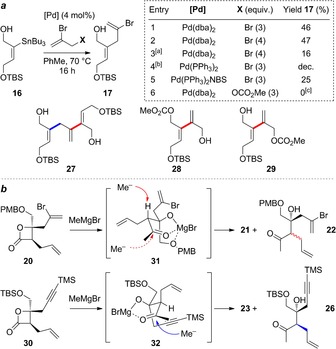
**a**: Attempted optimization of π‐allyl Stille coupling of stannane **26**. All reactions were carried out on a 150 mg scale. [a] Addition of stannane to a solution of bromide. [b] Catalyst formed in situ from Pd(dba)_2_/2 PPh_3_ on treatment with H_2_. [c] 32 % yield of a mixture of **28** and **29. b**: Addition of MeMgBr to β‐lactones **20** and **30**.

In comparing the efficiency of these two approaches, the first striking difference appears during the synthesis of Sharpless epoxidation substrates **18** and **25**, where the route to the latter proved significantly higher yielding (87 % to **25** from propargyl alcohol versus 17 % to **18** from butyne‐1,4‐diol). The root of this problem was the capricious Stille coupling of **16** with 2,3‐dibromopropene, which is not only inconvenient on large scale due to toxicity issues, but was also consistently plagued by the formation of side‐product **27**, which resulted from a second Stille coupling of desired product **17** with stannane **16**. Several conditions were screened in attempts to improve this step, a selection of which are depicted in Figure 2 a. Neither increasing the equivalents of dibromopropene (entry 2), nor slow addition of the stannane to a mixture of catalyst and electrophile (entry 3), improved the yield, albeit the latter conditions did suppress the formation of **27**. Switching to other sources of palladium did not improve matters (entries 4, 5), including the use of a Pd^II^‐succinimide complex reported by Taylor and Fairlamb to give superior yields in π‐allyl Stille couplings to common Pd^0^ sources.^22^ Surprisingly, use of an allylic carbonate coupling partner (X=OCO_2_Me, Entry 6) delivered none of the expected π‐allyl Stille coupling product,^23^ yielding instead a 1:0.86 mixture of alcohols **28** and **29**, again highlighting the unexpected reactivity of the vinylic C−Br bond over the allylic electrophile. The reluctance to form a π‐allylpalladium intermediate from dibromopropene may be due to the inductive electron‐withdrawing effect of the bromine atom at the 2‐position disfavoring π‐complexation of the metal prior to oxidative addition. In a further attempt to overcome this problem, we examined bromoalkene installation by alkyne bromoboration (using BBr_3_ or B‐Br‐9‐BBN); a number of desilylated alkynes were screened from the sequence towards alkyne **15**, but all led only to decomposition.

A second major difference between the two routes arose during the addition of methylmagnesium bromide to β‐lactone **20**, and its equivalent in the alkyne route (**30**, Figure 2 b). When using **20**, tertiary alcohol **21** was isolated in 66 % yield along with epimerized ketone **22** (24 %), which due to being a poorly‐separable epimeric mixture proved impossible to recycle in satisfactory yield. On performing the same reaction on alkyne‐lactone **30**, no epimerization of byproduct ketone **26** was observed, and its subsequent reaction with methylmagnesium bromide smoothly delivered an additional 34 % of diol **23** (an overall yield of 75 %). A possible explanation for this difference in reactivity involves formation of complex **31** following addition of MeMgBr to lactone **20**, via chelation of the tertiary alkoxide, OPMB group, and carbonyl oxygen to the magnesium ion. The stereoelectronically‐favored attack of a second methyl nucleophile necessitates pseudo‐axial approach from the hindered concave face of this chelate, which instead undergoes epimerization due to alignment of the σ_(C−H)_ and π*_(C=O)_ orbitals in this conformation. On the other hand, when performing this reaction on **30**, the presence of a non‐coordinating OTBS group affords a less constrained intermediate, which is able to access conformations such as **32** in which the methyl nucleophile can now approach with less steric hindrance, and epimerization is disfavored due to poor alignment of the σ_(C−H)_ and π*_(C=O)_ orbitals. Despite these complications, we were nonetheless able to access appreciable quantities of the AB ring carbon frameworks for each of the two derivatives, with the synthesis of **21** performed on more than gram scale with good yields. With these intermediates in hand, we now addressed completion of the AB rings, and the unveiling of the aldehyde sidechain from the pendent allyl group.

**Scheme 3:**
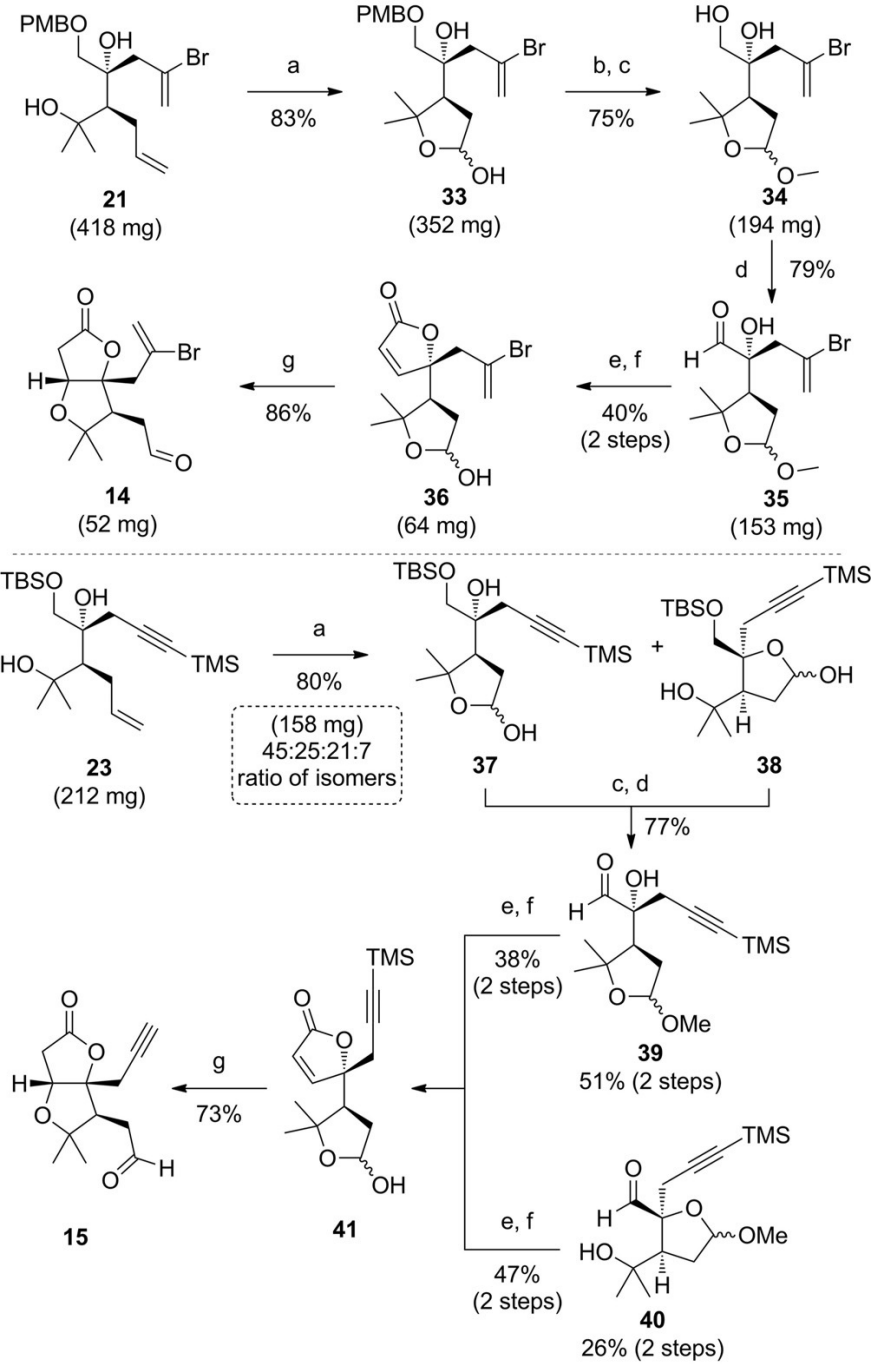
Reagents and conditions: a) OsO_4_ (2 mol %), NaIO_4_, 2,6‐lutidine, 1,4‐dioxane/ H_2_O; b) TFA, CH_2_Cl_2_, 0 °C; c) CSA (10 mol %), MeOH, 0 °C; d) SO_3_⋅py, *i*Pr_2_EtN, DMSO, CH_2_Cl_2_, 0 °C; e) (PhO)_2_P(O)CH_2_CO_2_Et, KHMDS, THF −20 °C→0 °C; f) TFA, CH_2_Cl_2_/H_2_O, 0 °C; g) K_2_CO_3_, MeOH. KHMDS=potassium bis(trimethylsilyl)amide; TFA=trifluoroacetic acid.

The second part of the optimized route towards the AB ring aldehyde **14** commenced with an oxidative cleavage of the alkene in **21**, which resulted in the regioselective formation of lactol **33** (Scheme 3). This ring was destined to act as a protecting group for the neighbouring tertiary alcohol, in order to avoid unreactive substrates later in the sequence.12a Oxidative PMB ether cleavage was found to proceed optimally by a short exposure to trifluoroacetic acid; subsequent methyl acetal formation delivered diol **34**. This was oxidized under Parikh–Doering conditions, and converted to lactol **36** via a (*Z*)‐selective Ando olefination/lactonization,^24^ followed by hydrolysis of the methyl acetal. Finally, cyclization of the B ring was readily achieved by a high‐yielding oxa‐Michael reaction under mildly basic conditions, delivering the AB ring aldehyde **14**.

When the same sequence was performed on the alkyne derivative **23**, we now observed the formation of a mixture of four isomers after the initial oxidative cleavage step, corresponding to two regioisomeric lactols, each as an epimeric mixture. The difference between this and the bromoalkene route can presumably be explained by the increased steric bulk of the bromoalkene sidechain disfavouring lactols equivalent to **38**. These isomers could not be separated, and were carried through the next two steps as a mixture, after which we were able to separate the isomeric aldehyde acetals **39** and **40** in good overall yield (77 % over two steps). Pleasingly, these successfully converged to lactol **41** after olefination and acetal hydrolysis, presumably due to acid‐catalyzed isomerization during methyl acetal deprotection. AB ring aldehyde **15** was isolated as a single compound after oxa‐Michael cyclization.

**Scheme 4:**
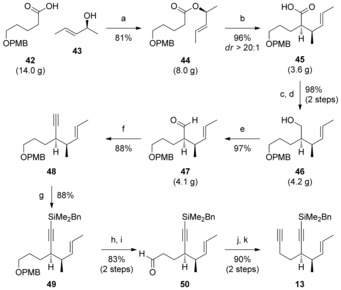
Reagents and conditions: a) EDC⋅HCl, NEt_3_, DMAP; b) LiHMDS, NEt_3_, PhMe, −78 °C→RT; then 5 % NaOH; then conc. HCl; c) TMSCHN_2_, MeOH/PhMe, 0 °C→RT; d) DIBALH, CH_2_Cl_2_, −78 °C→RT; e) DMP, NaHCO_3_, CH_2_Cl_2_; f) NaHMDS, [Ph_3_PCH_2_I]I, THF, −78 °C→RT; NaHMDS, −78 °C→RT; g) LiHMDS, THF, −78 °C; BnMe_2_SiCl, −78 °C to RT, 88 %; h) DDQ, CH_2_Cl_2_; i) DMP, NaHCO_3_, CH_2_Cl_2_; j) CBr_4_, PPh_3_, CH_2_Cl_2_, 0 °C; then **50**, NEt_3_, −30 °C→0 °C; k) *n*BuLi, THF, −78 °C→RT. DDQ=2,3‐Dichloro‐5,6‐dicyano‐1,4‐benzoquinone; DMP=Dess–Martin periodinane; EDC=1‐ethyl‐3‐(3‐dimethylaminopropyl) carbodiimide.

We now had in hand the aldehydes required for the different cyclization strategies, which were both due to be coupled with the CDE ring diyne **13**. The synthesis of this crucial component started from carboxylic acid **42** (Scheme 4, obtained in two steps from 1,5‐pentane diol),^25^ which underwent esterification with enantiopure alcohol **43** (prepared by enzymatic kinetic resolution, absolute configuration confirmed by Mosher ester analysis).^26^ The resulting ester **44** underwent a diastereoselective Ireland–Claisen rearrangement, setting up the two vicinal stereogenic centres of carboxylic acid **45**. The stereoselectivity of this rearrangement arises from the high (*Z*)‐selectivity of the enolization in the presence of excess triethylamine, as observed by Collum et al.^27^ It is worth noting that the use of other conditions, such as LiHMDS/TMSCl,^25^ led to inferior diastereoselectivity (9:1) compared to the direct rearrangement of the lithium enolate. Carboxylic acid was converted to its methyl ester (TMSCHN_2_) to improve the subsequent reduction to primary alcohol **46**, which was oxidized to aldehyde **47** (95 % yield over three steps); direct reduction of acid **45** to alcohol **46**, or of the intermediate methyl ester to aldehyde **47**, delivered poor yields/ decomposition. Stork‐Zhao olefination^28^ of **47** and in situ elimination of the intermediate (*Z*)‐vinyl iodide yielded alkyne **48**, which was converted to its benzyldimethylsilane derivative **49** in good yield. Installation of the second alkyne was achieved by PMB ether deprotection, oxidation, Ramirez olefination, and Fritsch–Buttenberg–Wiechell rearrangement to give diyne **13** in excellent yield. Overall, **13** could readily be prepared on multigram scale (≈3.5 g) in a total of 12 steps from alcohol **43** (43 % overall yield). With diyne **13** in hand, we turned our attention to exploration of its conversion to the full CDEFG ring system via metal‐catalyzed cyclization and FG ring construction, as a prelude to an assault on the natural product itself.

The palladium‐catalyzed polycyclization of bromoendiynes has been known for more than 25 years, since the pioneering work from the groups of de Meijere15c, 29 and Negishi.15a,15b Although this field has expanded to the synthesis of many different molecules, including natural product‐like polycyclic systems^30^ and axially chiral biaryls,^31^ no applications of this chemistry in natural product total synthesis had been reported at the outset of our work. A particular challenge in the present synthetic context is the need for the palladium catalyst to mediate a 7‐membered ring formation, a feat not reported in the bromoenediyne methodological context, albeit precedented in other carbopalladative cyclizations.^32^ Importantly, the bromoenediyne cyclization benefits from the presence of a silane substituent at the terminus of the diyne component, which avoids side reactions based on further carbopalladation processes. This is particularly convenient for our synthesis, where we envisaged that this alkynylsilane, which is converted to an arylsilane in the cyclization, would serve as a masked phenol through eventual aromatic Tamao oxidation.^33^ 7‐membered ring formation is similarly challenging for the cyclotrimerization approach,16a–16c but for both strategies we had already demonstrated that simple bromoendiynes and triynes could access the abridged CDE cores. Before embarking upon the synthesis of the full rubriflordilactone A framework, we now decided to explore advanced model systems to assess the feasibility of each route with diyne **13**, and also to establish conditions for F and G ring installation, and thereby the synthesis of a truncated CDEFG rubriflordilactone A analogue.

**Scheme 5:**
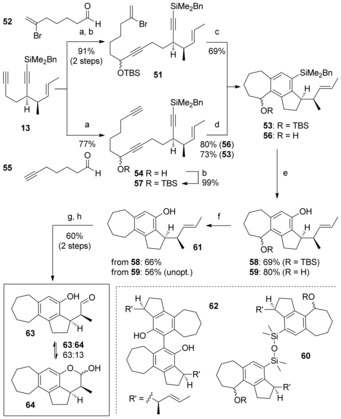
Reagents and conditions: a) **13**, LiHMDS, THF, −78 °C; then add aldehyde; b) TBSCl, imid., DMAP, CH_2_Cl_2_; c) Pd(PPh_3_)_4_ (5 mol %), NEt_3_, MeCN, 80 °C; d) CpCo(CO)_2_ (20 mol %), PPh_3_ (40 mol %), PhCl, MW (300 W), 150 °C; e) TBAF, THF; then MeOH, H_2_O_2_, KHCO_3_; f) Et_3_SiH, ZnCl_2_, CH_2_Cl_2_; TBAF, THF; g) OsO_4_ (4 mol %), NMO, acetone/H_2_O (3:1); h) NaIO_4_/SiO_2_, CH_2_Cl_2_. Cp=cyclopentadienyl; NMO=*N*‐methylmorpholine‐*N*‐oxide; TBAF=tetrabutylammonium fluoride.

The palladium‐catalyzed cyclization of bromoenediyne **51** (generated in two steps from **13** and aldehyde **52**, 73 %) was first studied (Scheme 5). We were pleased to find that the additional pendent alkene functionality in this diyne was tolerated, although a small optimization was necessary to reach a satisfying 69 % yield of tricycle **53**. When employing triyne **54** (obtained from **13** and aldehyde **55**, 77 %), we were delighted to obtain an 80 % yield of **56** for the cobalt‐catalyzed cyclotrimerization under microwave heating conditions, which were essential for success in 7‐membered ring formation.12b, 34 The cyclization also proved efficient with the TBS‐protected triyne variant **57**, albeit in slightly lower yield (73 %), which allowed the two parallel approaches to converge at a common intermediate **53** in readiness for Tamao oxidation and cationic reduction of the benzylic alcohol/ ether. In this oxidation, the conditions optimized previously required slight adjustment^35^ to achieve good yields of phenol **58**, due to the problematic formation of disiloxane **60** as observed in crude ^1^H NMR spectra. Cationic benzylic reduction of **59** proceeded smoothly, delivering **61** in 77 % yield. This two‐step sequence could similarly be performed on the free alcohol **56**.

With phenol **61** in hand, we next investigated oxidative cleavage of the (*E*)‐alkene sidechain. A Johnson‐Lemieux process was first tested, but this proved rather capricious under classical conditions (5 mol % aq. OsO_4_, 4.0 equiv NaIO_4_, 2.0 equiv 2,6‐lutidine, 35–64 % yield). Attempted ozonolysis in CH_2_Cl_2_ at −78 °C gave rise to a complex mixture of products, whilst the use of RuCl_3_ triggered the formation of biphenyl **62**, presumably via an oxidative radical coupling as observed by Ayres and Gopalan.^36^ Suspecting that formation of the intermediate diol was the problematic step in the one‐pot Johnson–Lemieux reaction, we tested a stepwise procedure; in the event, Upjohn dihydroxylation yielded 61 % of intermediate diols, which pleasingly underwent smooth oxidative cleavage in under 10 minutes using the Shing protocol of silica‐supported sodium periodate.^37^ This delivered a 63:13 mixture of open‐chain aldehyde **63** and lactol **64** (98 %).

**Scheme 6:**
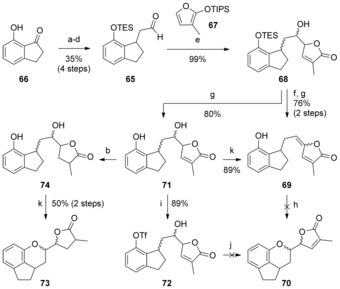
Reagents and conditions: a) Ph_3_PCHCO_2_Me, PhMe, 110 °C; b) H_2_, Pd/C, MeOH; c) TESCl, imid., DMAP, CH_2_Cl_2_; d) DIBAL‐H, CH_2_Cl_2_, −78 °C; e) BF_3_⋅OEt_2_, Et_2_O, −78 °C→0 °C; f) MsCl, NEt_3_, CH_2_Cl_2_, 0 °C→RT; g) TBAF, THF; h) Conditions (see text); i) Tf_2_O, *i*Pr_2_EtN, CH_2_Cl_2_, 0 °C; j) Pd(OAc)_2_ (10 mol %), dppf or (*S*)‐tolBINAP (12 mol %), K_2_CO_3_, PhMe, 90 °C; k) DIAD, PPh_3_, CH_2_Cl_2_, 0 °C. DIAD=diisopropyl azodicarboxylate; dppf=1,1′‐bis(diphenylphosphino)ferrocene; Ms=SO_2_Me; TES=SiEt_3_; TES=triethylsilyl; Tf=SO_2_CF_3_.

The introduction of the G ring could conceivably proceed through either of these intermediates. To explore these alternatives, we first studied two simpler model systems lacking the 7‐membered C ring and the F ring methyl group. The first of these was aldehyde **65**, an analogue of aldehyde **63**. This was prepared from known indanone **66** 38 (Scheme 6) by Wittig olefination, alkene and ester reduction, and protection of the phenol as a triethylsilyl ether (to prevent issues with equilibrium of the intermediate phenol‐aldehyde and the corresponding lactol). Promoted by BF_3_⋅OEt_2_, **65** was reacted with silyloxyfuran **67** (prepared in four steps from citraconic anhydride)^39^ to afford the DEG ring alcohol **68** in quantitative yield.

We first envisaged completion of the DEFG rings by intramolecular 1,6‐oxa‐Michael addition from diene **69**. Whilst formation of this diene from **68** was unproblematic, all attempts to effect the conjugate addition failed, including a wide range of basic (e.g. *t*BuOK, NaH, KHMDS, Cs_2_CO_3_) or acidic (e.g. InCl_3_, Sc(OTf)_3_, FeCl_3_, TiCl_4_, La(NO_3_)_3_⋅5 H_2_O, *p*‐TsOH) conditions. These reactions mostly resulted in no conversion (which we attributed to the poor nucleophilicity of the phenoxide ion and/ or the reversibility of addition) or decomposition.

Other pathways were then explored using phenol **71**, which was accessed by acidic deprotection of the TES group in **68**.^40^ However, attempted intramolecular palladium‐catalyzed C−O bond formation^41^ after regioselective conversion of the phenol group to triflate **72** proved unsuccessful. A final attempt to construct the F ring was made via an intramolecular Mitsunobu reaction,^42^ which resulted only in the elimination of the alcohol to diene **69** even at temperatures as low as −78 °C. We surmised that the stability deriving from conjugation of the diene in **69** was an insurmountable barrier to cyclization, a hypothesis that was confirmed by the successful isolation of dihydroDEFG ring product **73** when the equivalent Mitsunobu cyclization was carried out on the saturated derivative **74**.

**Scheme 7:**
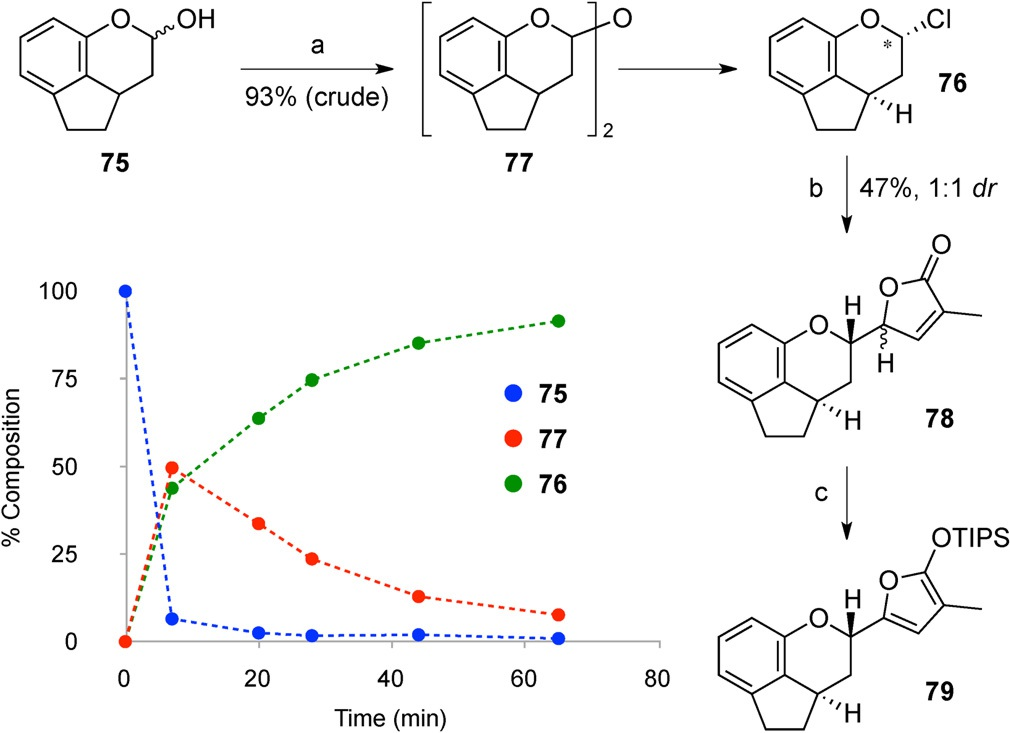
Reagents and conditions: a) SOCl_2_, ZnCl_2_, PhMe, 0 °C→RT; b) ZnCl_2_, CH_2_Cl_2_, −40 °C→RT; c) TIPSOTf, Et_3_N, CH_2_Cl_2_, 0 °C. TIPS=triisopropylsilyl.

With these unfruitful results in hands, we decided to adjust the order of FG ring formation, and examine the addition of a butenolide nucleophile to an oxocarbenium ion derived from an F ring acetal (or similar derivative). Based on some preliminary experiments using pyranyl acetals or acetates, it soon became apparent that a good leaving group would be required in the generation of the oxocarbenium ion, and we were attracted to the report of Vercellotti et al. on the use of thionyl chloride and zinc chloride to effect the formation of pyranosyl chlorides.^43^ Initial attempts to form an F ring chloropyran from lactol **75** 44 (Scheme 7) unexpectedly resulted in the formation of mixtures of the desired chloropyran **76**, and dimer **77**; however, we found that by extending the reaction time, **76** could be isolated in excellent yield (≈93 %, crude) and as a single diastereomer, which was assigned as the α‐anomer on the basis of coupling constants in the ^1^H NMR spectrum.^44^ Monitoring of the reaction by ^1^H NMR spectroscopy revealed a rapid initial formation of the dimer (<10 min), followed by slower conversion to **76**. We hypothesize that activation of lactol **75** by thionyl chloride/zinc chloride indeed leads to the formation of the corresponding oxocarbenium ion, which is rapidly trapped by unreacted lactol to give dimer **77**, a process that is favoured at the beginning of the reaction when lactol concentration is high. However, dimer formation appears to be a reversible process, where the action of zinc chloride reforms an oxocarbenium that is trapped by chloride as the reaction progresses.

With chloropyran **76** in hand, we next addressed the addition of siloxyfuran **67**. Optimal results were found using 1.5 equivalents of **67** with 40 mol % of zinc chloride in CH_2_Cl_2_, allowing the reaction mixture to slowly warm up from −40 °C to room temperature overnight. This led to the isolation of 47 % of the DEFG ring system **78**, as a 1:1 mixture of diastereomers. Pleasingly, facial selectivity for addition to the oxocarbenium ion was high, presumably directed by the proximal stereocentre and stereoelectronic effects; the low stereoselectivity at the newly formed butenolide stereocentre presumably reflects poor facial selectivity in an open transition state. In an effort to improve this ratio, we examined epimerization of this stereocentre, reasoning that the conversion of the newly appended G ring back to a silyloxyfuran could improve the *dr* (*dr*=diastereomeric ratio) upon reformation of the butenolide by hydrolysis. Siloxyfuran **79** was generated by treatment of **78** with TIPSOTf and triethylamine at 0 °C, and a variety of conditions were then screened to reform the G ring (e.g., TBAF, THF; (±)‐camphorsulfonic acid (CSA), MeOH; aq. citric acid/CH_2_Cl_2_; AcOH/THF/H_2_O). Unfortunately, these all yielded almost exclusively the elimination product **69**, likely again due to the good leaving group ability of the phenol, with only trace amounts of **78** observed as a mixture of diastereomers.

**Scheme 8:**
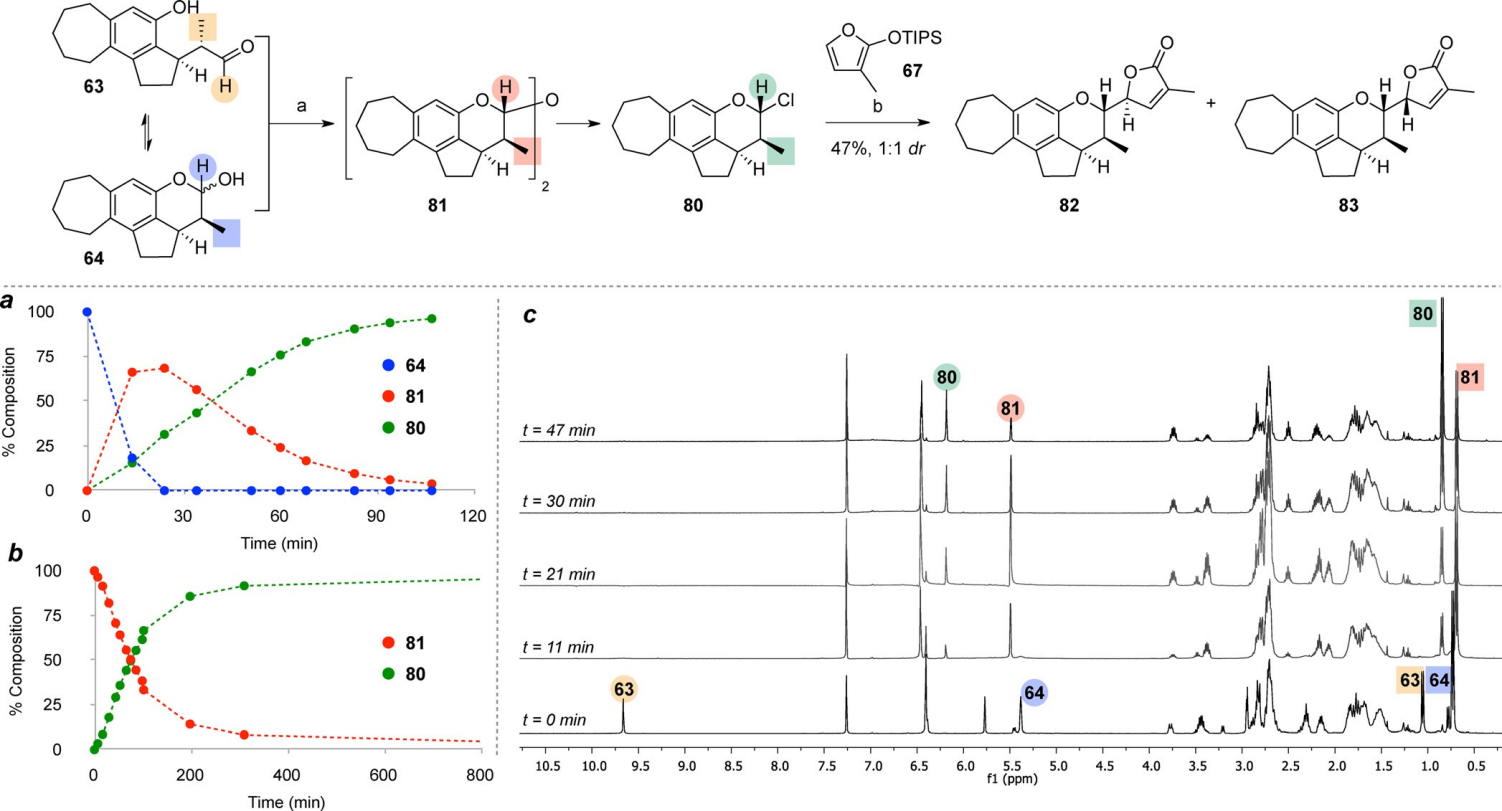
Reagents and conditions: a) SOCl_2_, ZnCl_2_, PhMe, 0 °C→RT; b) ZnCl_2_, CH_2_Cl_2_, −40 °C→RT. Graph **a**: Reaction profile (monitored by ^1^H NMR for conversion of lactol **64** to chloropyran **80** via dimer **81**; Graph **b**: Reaction profile for conversion of **81** to **80**; Graph **c**: Example ^1^H NMR timecourse experiment.

Despite the poor *dr* observed with this model system, the successful incorporation of the G ring was nonetheless encouraging. We were delighted to observe similar reactivity when switching to the more elaborate CDEF ring system **64** (Scheme 8), with formation of the chloropyran derivative **80** from the corresponding dimer **81** (4 equiv SOCl_2_, 5 equiv ZnCl_2_). Monitoring of the reaction by ^1^H NMR spectroscopy (Scheme 8 a–c) showed almost complete conversion of lactol **64** to a mixture of dimer **81** and chloropyran **80** over 45 minutes, although almost two hours reaction time was necessary to reach complete conversion (Scheme 8 a). The reaction pathway was confirmed by subjecting dimer **81** to similar reaction conditions (8 equiv SOCl_2_, 10 equiv ZnCl_2_, Scheme 8 b). The obtained chloropyran was used without further purification in the ZnCl_2_‐promoted butenolide addition step. Pleasingly, this delivered the CDEFG ring system as a single diastereomer at the F ring stereocentre, and a 1:1 diastereomeric mixture at the butenolide stereocentre (**82** and **83**)^45^ in 45 % yield over two steps. The truncated natural product **82** is of interest itself in the study of structure–activity relationships in the rubriflordilactones.

**Scheme 9:**
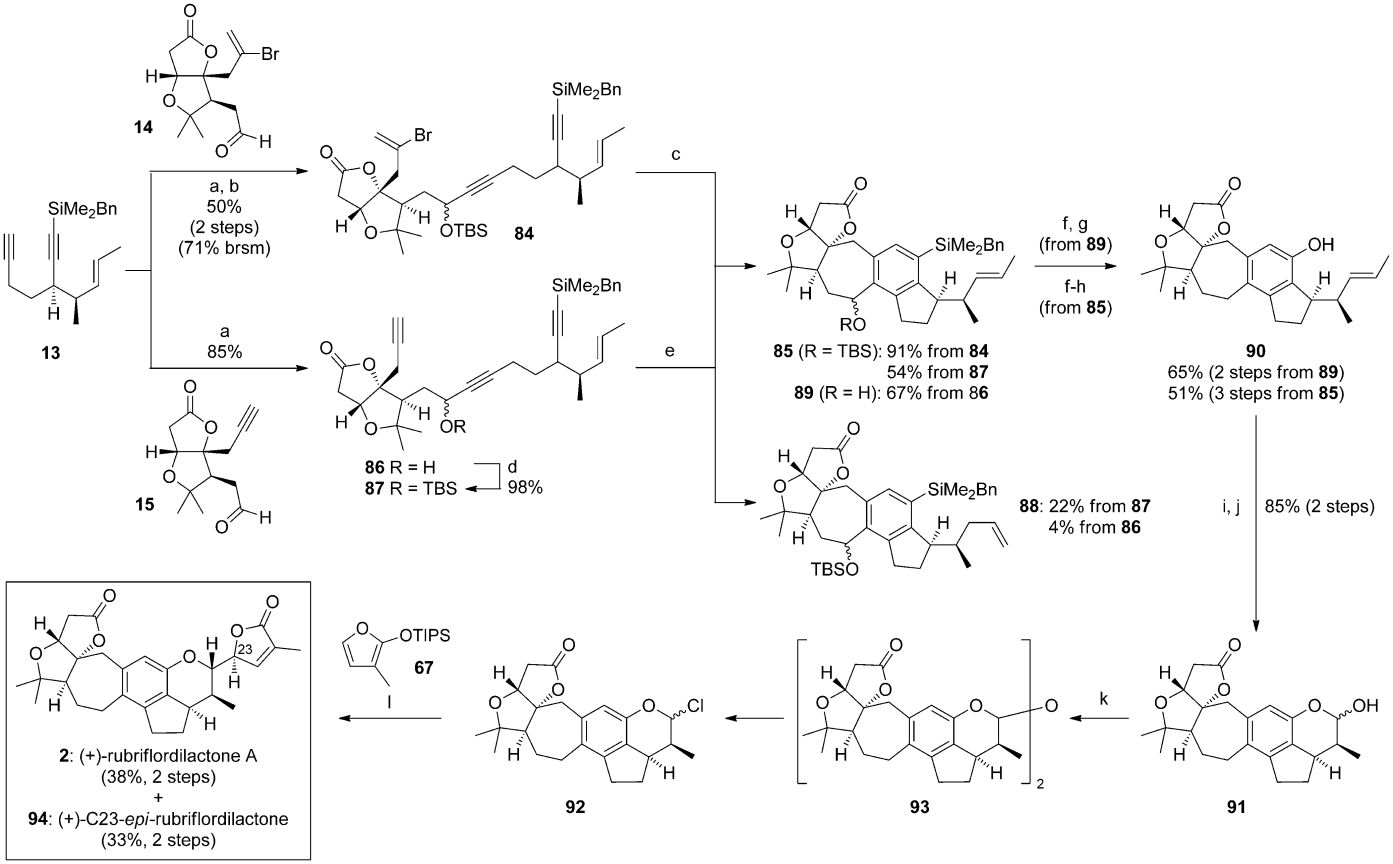
Reagents and conditions: a) **13**, *n*BuLi, THF, −78 °C; then add aldehyde **14** or **15**, −78 °C→−10 °C; b) TBSOTf, 2,6‐lutidine, CH_2_Cl_2_, 0 °C→RT; c) Pd(PPh_3_)_4_ (10 mol %), NEt_3_, MeCN, 80 °C; d) TBSCl, imid., DMAP, CH_2_Cl_2_; e) CpCo(CO)_2_ (20 mol %), PPh_3_ (40 mol %), PhCl, MW (300 W), 150 °C; f) TBAF, THF; then MeOH, H_2_O_2_, KHCO_3_; g) Et_3_SiH, ZnCl_2_, CH_2_Cl_2_; h) TBAF, THF; i) OsO_4_ (2 mol %), NMO, acetone/H_2_O (3:1); j) NaIO_4_/SiO_2_, CH_2_Cl_2_; k) SOCl_2_, ZnCl_2_, PhMe, 0 °C→RT; l) **67**, ZnCl_2_, CH_2_Cl_2_, −40 °C→RT.

These collected model systems had provided valuable information on suitable method been developed to optimize incorporation of the F and G rings. Despite poor diastereoselectivity at the butenolide stereocentre, the furan addition reaction exhibited high stereoselectivity at the F ring, and excellent regioselectivity on the butenolide itself, which gave us much confidence for our assault on the total synthesis of rubriflordilactone A. This commenced with the separate addition of the CDE diyne **13** to AB ring aldehydes **14** and **15** (Scheme 9). To our delight, the TBS‐protected secondary alcohol **84** (deriving from aldehyde **14**) underwent palladium‐catalyzed cascade cyclization to give **85** in an excellent 91 % yield. As observed previously, the triynes arising from addition of **13** to aldehyde **15** could be cyclotrimerized to the ABCDE ring system without (triyne **86**) or with (triyne **87**) protection of the secondary alcohol as a TBS ether. However, a decrease in yield was noted when using the protected triyne **87** (54 %), due to an unexpected isomerization of the alkene sidechain to terminal alkene **88** (22 %). As this behaviour was not observed in the model systems, or indeed for triyne **86** (67 % of ABCDE alcohol **89**), we assume that the presence of this bulky silyl ether substituent adjacent to the site of cyclotrimerization must retard the initial oxidative coupling, which allows isomerization to compete.^46^

Tamao oxidation and benzylic reduction were performed on both the free alcohol **89** and its silyl ether analogue **85**, delivering phenol **90** in 65 % yield (2 steps) and 51 % yield (3 steps) respectively; a stepwise alkene dihydroxylation/ diol cleavage then cleanly installed the F ring (**91**, 85 % yield). As observed before, treatment of lactol **91** with thionyl chloride and zinc chloride generated chloropyran **92** via dimer **93**, with a reaction time of 3 h required for complete chloropyran formation. Introduction of the G ring was carried out under the same conditions as used for the model systems, which led to the isolation of rubriflordilactone A **2** in 38 % yield, along with 33 % of its C23‐epimer **94** (over 2 steps).

Although all spectroscopic data for synthetic (+)‐**2** were in agreement with that of the natural product, and the Li group's synthetic sample, the specific rotation was found to be equal in value but of opposite sign to the isolation sample ([α]_D_^25^ +58.3 (*c=*0.114 g/100 mL MeOH); lit. [α]_D_^25^ −58.1 (*c=*0.114  g/100 mL MeOH)).^5^, ^26^ However, communications with the Li group revealed that both *synthetic* samples were in agreement. Although this at first suggests that both we (and Li et al.) had synthesized the unnatural enantiomer of the natural product, the stereoselective nature of our synthetic routes, combined with the conservation of the stereochemistry of the AB rings in other schinortriterpenoid compounds (including those calculated and measured using CD spectroscopy,^47^ and prepared by synthesis) indicates otherwise. An explanation for this discrepancy remains unclear.

## Conclusion

A series of model studies provided a firm synthetic footing for the formation of the CDE rings of (+)‐rubriflordilactone A using either a palladium‐catalyzed cascade cyclization or a cobalt‐catalyzed cyclotrimerization, and for the installation of the F and G rings though the intermediacy of a chloropyran. NMR studies revealed that this latter compound is formed via a pyran dimer, which over time is converted to the chloropyran. The two transition metal‐catalyzed routes were applied to the total synthesis, with late‐stage convergency between the approaches. Comparison of spectroscopic data revealed an inconsistency in the specific rotation between synthetic and isolation samples, which remains unresolved. The modular nature of the synthesis renders it suitable for application to other members of the *Schisandraceae* family; efforts towards the total synthesis of such compounds are currently underway in our laboratory.

## Experimental Section

Experimental details are given in the Supporting Information. These include details of the synthetic procedures, spectroscopic data, and copies of the ^1^H and ^13^C NMR spectra for novel compounds.

## Conflict of interest

The authors declare no conflict of interest.

## Supporting information

As a service to our authors and readers, this journal provides supporting information supplied by the authors. Such materials are peer reviewed and may be re‐organized for online delivery, but are not copy‐edited or typeset. Technical support issues arising from supporting information (other than missing files) should be addressed to the authors.

Supplementary
