# Supplementary material for: Total Synthesis of the Schisandraceae Nortriterpenoid Rubriflordilactone A
Source: Chemistry. 2017 Sep 8;23(56):14080–9. doi: 10.1002/chem.201703229 (PMC5656881; doi:10.1002/chem.201703229)

CHEMISTRY
A European Journal

Supporting Information

Total Synthesis of the *Schisandraceae* Nortriterpenoid Rubriflordilactone A

Guilhem Chaubet,[a] Shermin S. Goh,[a] Mujahid Mohammad,[a] Birgit Gockel,[a] Marie-Caroline A. Cordonnier,[a] Hannah Baars,[b] Andrew W. Phillips,[a] and Edward A. Anderson*[a]

chem_201703229_sm_miscellaneous_information.pdf

CONTENTS

1. EXPERIMENTAL

1.1 General Experimental Considerations 2

1.2 Experimental procedures and characterization data 3

1.2.1 Synthesis of AB rings – Scaled up to compound **21** 3

1.2.2 Synthesis of the CDEF ring model system 15

1.2.3 Synthesis of the DEFG ring model system 27

1.2.4 Synthesis of the CDEFG ring model system 40

1.2.5 Rubriflordilactone A 44

2. REFERENCES 48

3. NMR SPECTRA 49

1. EXPERIMENTAL

1.1 General Experimental Considerations

Nuclear Magnetic Resonance Spectroscopy: ^{1}H NMR spectra were acquired on Bruker DRX500, AVII500 (500 MHz, with cryoprobe) or AVIII400 (400 MHz) spectrometers and were referenced to residual non-deuterated solvent peaks in $CDCl_3$ (δ = 7.26) or C_5D_5N (δ = 8.74, 7.58, 7.22). Chemical shifts (δ_H and δ_C) are reported in parts per million (ppm) with signal splittings recorded as singlet (s), doublet (d), triplet (t), quartet (q), quintet (quin), and multiplet (m); app = apparent. Coupling constants (*J*) are measured to the nearest 0.1 Hz. ^{13}C NMR spectra were obtained on Bruker AVII500 (126 MHz, with cryoprobe) or AVIII400 (101 MHz) spectrometers and were referenced to solvent peaks in $CDCl_3$ (δ = 77.16) or C_5D_5N (δ = 150.35, 135.91, 123.87).

Mass Spectrometry: Low-resolution mass spectra (*m/z*) were recorded on a Waters LCT Premier EX mass spectrometer, using electrospray ionization (ESI). High-resolution mass spectra (HRMS) were recorded by the Departmental Mass Spectrometry Service, University of Oxford on a Bruker MicroTOF (resolution = 5000 FWHM) using electrospray ionisation (ES^+). The parent ion $[M]^+$, $[M+H]^+$ or $[M+Na]^+$ is calculated to 4 decimal places from the molecular formula, and all values are within a tolerance of 5 ppm.

Infrared Spectroscopy: Absorption spectra were obtained in $CHCl_3$ as solvent on a Bruker Tensor 27 FT-IR spectrometer. The sample was prepared as a thin film on a diamond/ZnSe PIKE Miracle ATR module. Wavelengths of maximum absorbance (ν_{max}) are quoted in wavenubers (cm^{-1}). Only selected, characteristic IR absorption data are provided for each compound.

Specific rotations: Optical rotations were recorded on a Perkin Elmer 241 or 341 polarimeter with a path length of 1 dm (using the sodium D line, 589 nm). Specific rotations ($[\alpha]_D^{25}$) are reported in units of 10^{-1} deg cm^2 g^{-1}. Concentrations are reported in g/100 mL. Temperatures are reported in °C (typically 25 °C).

Chromatography: Flash chromatography refers to normal phase column chromatography on silica gel using a head pressure of N_2, using either Merck Geduran® Silicagel 60 (40–63 mm) or Macherey-Nagel Silica 60 M (40 - 63 mm). Thin-layer chromatography was performed on Merck Kieselgel 60 F_{254} plates with visualization by ultraviolet light (254 nm) and/or heating the plate after staining with vanillin or $KMnO_4$. High performance liquid chromatography (HPLC) was performed on an Agilent 1200 Series running in normal phase under UV detection using a ZORBAX RX-SIL (150 mm x 4.6 mm ID) as the analytical column. Chiral analysis was carried out using DAICEL CHIRALPAK-IA, IB or IC (250 mm x 4.6 mm ID).

Materials: Unless otherwise stated, all reactions were carried out in oven-dried glassware under an atmosphere of argon, using anhydrous reaction solvents. Et_2O, CH_2Cl_2, THF and toluene were dried over activated alumina before use. All other commercially available reagents and solvents were either used as received, and/or dried and purified before use using standard procedures. Petroleum ether refers to the fraction of light petroleum ether boiling at 40–60 °C unless stated otherwise.

1.2 Experimental procedures and characterization data

1.2.1 Scaled up synthesis of AB rings

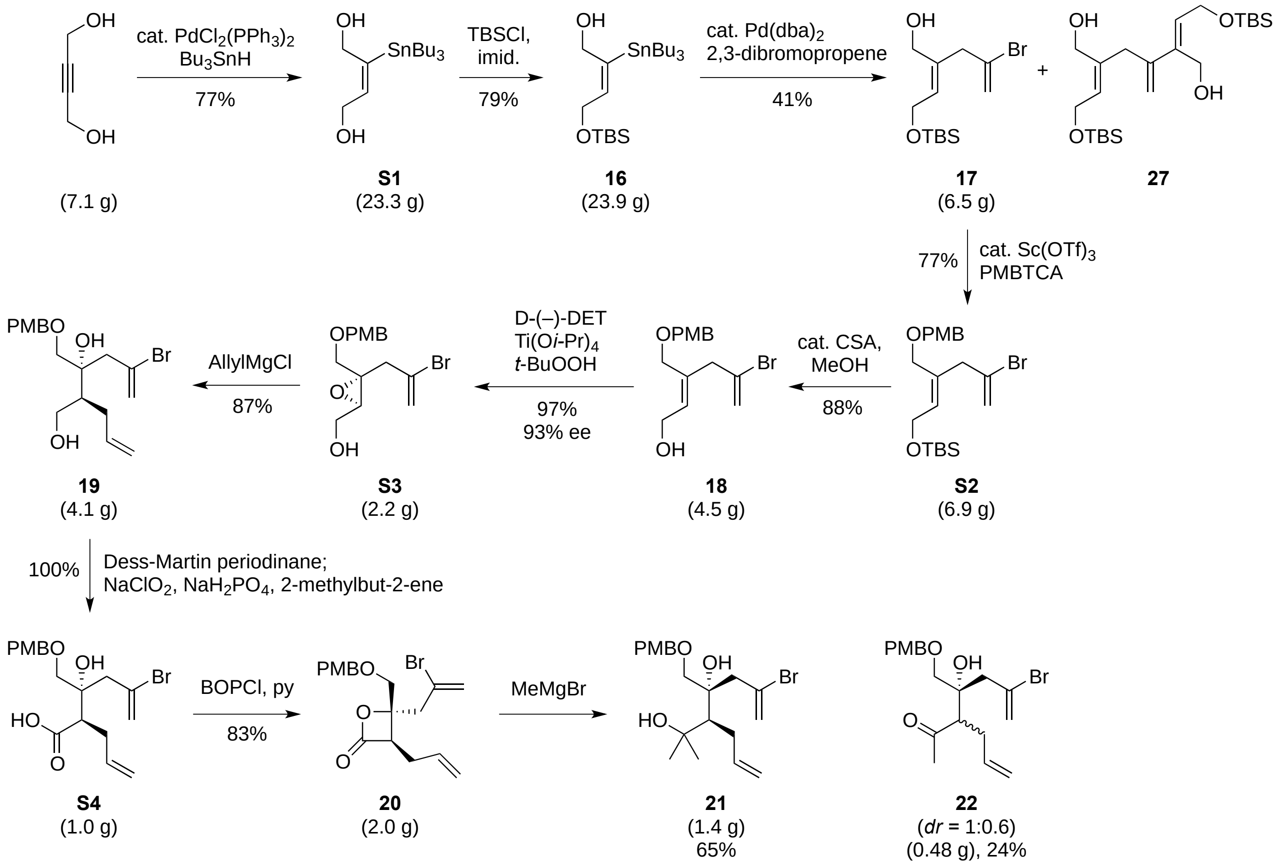

Scheme S1: Synthesis of diol **21**. Indicated masses correspond to the largest scale each compound was isolated from a single experiment.

(*E*)-2-(Tributylstannyl)but-2-ene-1,4-diol, S1

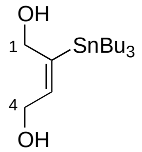

To a stirred solution of but-2-yne-1,4-diol (7.1 g, 82.4 mmol, 1.03 equiv.) in dry THF (150 mL) under argon was added $PdCl_2(PPh_3)_2$ (1.5 g, 2.13 mmol, 2.6 mol%). Tributyltin hydride (21 mL, 80 mmol, 1.0 equiv.) was added slowly, and the reaction mixture was stirred for 20 min. The solvent was then removed *in vacuo*, and the residue purified by flash chromatography (4:1→1:1 petroleum ether / EtOAc) to afford (*E*)-2-(tributylstannyl)but-2-ene-1,4-diol **S1** (23.3 g, 61.8 mmol, 77%) as light brown oil.

***R*$_f$** 0.43 (1:1 petroleum ether / EtOAc); **ν_{max} (thin film)/cm^{-1}** 3330 (br, OH), 2956 (s), 2924 (s), 2871 (s), 2853 (s), 1614 (w, C=C), 1462 (m); **^{1}H NMR (400 MHz, $CDCl_3$)** δ_H 5.79 (1H, tt, J_{HH} = 5.9 and 2.1 Hz; J_{SnH} = 66.7 Hz, H3), 4.38 (2H, m; J_{SnH} = 36.3 Hz, H1), 4.20 (2H, *app* t, J_{HH} = 5.4 Hz, H4), 1.74 (1H, t, J_{HH} = 4.9 Hz, OH), 1.73-1.27 (13H, m, $SnCH_2C\underline{H}_2C\underline{H}_2CH_3$, OH), 0.96-0.85 (15H, m, $SnC\underline{H}_2CH_2CH_2C\underline{H}_3$); **^{13}C NMR (100 MHz, $CDCl_3$)** δ_C 149.4, 138.2, 63.6, 59.8, 29.3, 27.5, 13.8, 10.1; **HRMS (ESI$^+$)** calc. for $C_{16}H_{34}O_2SnNa$ ([M+Na]$^+$): 401.1473; found: 401.1476. Data in accordance with the literature.[1]

(*E*)-4-((*tert*-Butyldimethylsilyl)oxy)-2-(tributylstannyl)but-2-en-1-ol, 16

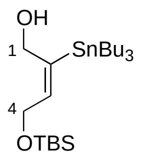

To a stirred solution of (*E*)-2-(tributylstannyl)but-2-ene-1,4-diol **S1** (23.3 g, 61.8 mmol, 1.0 equiv.) in dry DMF (60 mL) under argon at 0°C was added imidazole (4.21 g, 61.8 mmol, 1.0 equiv.) and TBSCl (9.31 g, 61.8 mmol, 1 equiv.). The reaction mixture was stirred for 60 min, then it was quenched with water. The layers were separated and the aqueous layer was extracted three times with diethyl ether (150 mL × 3). The combined organic layers were dried (Na_2SO_4) and the solvent was removed in vacuo. The crude product was purified by flash chromatography (10:1 petroleum ether / EtOAc) to yield **16** (23.9 g, 48.7 mmol, 79%) as a light brown oil.

***R**$_f$* 0.57 (4:1 petroleum ether / EtOAc); **ν_{max} (thin film)/cm^{-1}** 3443 (br, O-H), 2956 (s), 2928 (s), 2856 (s), 1930 (w), 1614 (w, C=C), 1463 (s), 1256 (s), 1079 (s), 837 (s); **^{1}H NMR (400 MHz, CDCl$_3$)** δ_H 5.70 (1H, tt, J_{HH} = 5.4 and 2.1 Hz; J_{SnH} = 68.2 Hz, H3) 4.34 (2H, br d with unresolved fine coupling, J_{HH} = 5.5; J_{SnH} = 37.0 Hz, H1), 4.25–4.16 (2H, m, H4) 1.81 (1H, t, *J* = 5.4 Hz, OH), 1.55-1.46 (6H, m, $SnCH_2C\underline{H_2}CH_2CH_3$), 1.37-1.27 (6H, m, $SnCH_2CH_2C\underline{H_2}CH_3$), 0.94–0.85 (15H, m, $SnC\underline{H_2}CH_2CH_2C\underline{H_3}$ and Si*t*-Bu), 0.09 (6H, s, $SiMe_2$); **^{13}C NMR (100 MHz, CDCl$_3$)** δ_C 156.1, 139.0, 63.9, 60.9, 29.3, 27.5, 26.0, 13.8, 10.1, –5.0; **HRMS (ESI$^+$)** calc. for $C_{22}H_{48}O_2SiSnNa$ ([M+Na]$^+$): 515.2338; found: 515.2341. Data in accordance with the literature.[1]

(*Z*)-4-Bromo-2-(2-((*tert*-butyldimethylsilyl)oxy)ethylidene)pent-4-en-1-ol, 17 and (2*Z*,5*Z*)-2,5-bis(2-((*tert*-butyldimethylsilyl)oxy)ethylidene)-3-methylenehexane-1,6-diol, 27

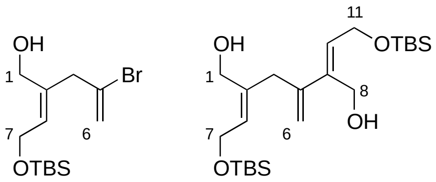

17 **27**

To a stirred solution of **16** (24.67 g, 50.2 mmol, 1.0 equiv.) in dry degassed toluene (375 mL) under argon was added Pd(dba)$_2$ (1.16 g, 2.01 mmol, 4 mol%). The solution was degassed (Ar purge) for a further 30 min, then 2,3-dibromopropene (14.7 mL, 150.6 mmol, 3.0 equiv.) was added and the reaction was heated to 70 °C overnight. The reaction was cooled to rt, and the solvent was removed *in vacuo*. The crude product was purified by flash chromatography (4:1 petroleum ether / EtOAc) to afford **17** (6.54 g, 20.4 mmol, 41%) as yellow oil, along with byproduct **27** (724 mg, 1.71 mmol, 21%) as a light yellow oil.

Characterization for 17: ***R**$_f$* 0.30 (4:1 petroleum ether / EtOAc); **ν_{max} (thin film)/cm^{-1}** 3377 (br, O-H), 2930 (s), 2857 (s), 1626 (w, C=C), 1471 (s), 1100 (s), 1061 (s), 836 (s); **^{1}H NMR (500 MHz, CDCl$_3$)** δ_H 5.69 (1H, app. q, *J* = 1.3 Hz, H6), 5.65 (1H, t, *J* = 6.1 Hz, H2), 5.52 (1H, d, *J* = 1.6 Hz, H6), 4.29 (2H, d, *J* = 6.1 Hz, H1), 4.14 (2H, s, H7), 3.25 (2H, s, H4), 2.20 (1H, s, OH), 0.91 (9H, s, Si*t*-Bu), 0.10 (6H, s, $SiMe_2$); **^{13}C NMR (125 MHz, CDCl$_3$)** δ_C 137.5, 131.8, 131.0, 118.9, 60.1, 59.6, 47.7, 26.0, 18.4, –5.1; **HRMS (ESI$^+$)**

calc. for $C_{13}H_{25}O_2BrSiNa$ ($[M+Na]^+$): 343.0699; found: 343.0697. Data in accordance with the literature.[1]

Characterization for 27: ***R*** R_f 0.07 (4:1 petroleum ether / EtOAc); **ν_{max} (thin film)/cm^{-1}** 3378 (br, OH), 3085 (m), 3052 (s), 2955 (s), 2930 (s), 2885 (s), 2857 (s), 2306 (w), 1720 (m), 1606 (m), 1472 (s); **^{1}H NMR (500 MHz, CDCl$_3$)** δ_H 5.83 (1H, t, *J* = 6.3 Hz, H10), 5.56 (1H, t, *J* = 6.3 Hz, H2), 5.29 (1H, d, *J* = 0.8 Hz, H6), 5.06 (1H, d, *J* = 0.8 Hz, H6), 4.32 (2H, d, *J* = 6.3 Hz, H11), 4.29 (2H, s, H8), 4.24 (2H, d, *J* = 6.3 Hz, H1), 4.09 (2H, s, H7), 3.10 (2H, s, H4), 2.48 (1H, s, OH), 1.63 (1H, s, OH), 0.90 (9H, s, Si*t*-Bu), 0.89 (9H, s, Si*t*-Bu), 0.08 (6H, s, SiMe$_2$), 0.07 (6H, s, SiMe$_2$); **^{13}C NMR (125 MHz, CDCl$_3$)** δ_C 144.9, 141.0, 140.1, 130.1, 129.3, 115.1, 60.6, 59.9, 59.6, 58.9, 41.0, 26.0 (2C), 18.4 (2C), –5.0, –5.1; **HRMS (ESI$^+$)** calc. for $C_{23}H_{46}O_4SiNa$ ($[M+Na]^+$): 465.2827; found: 465.2826.

(*Z*)-((5-Bromo-3-(((4-methoxybenzyl)oxy)methyl)hexa-2,5-dien-1-yl)oxy)(*tert*-butyl)dimethyl silane, S2

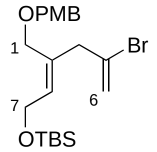

To a stirred solution of **17** (6.54 g, 20.4 mmol, 1 equiv.) in toluene (250 mL) under argon was added *p*-methoxybenzyl trichloroacetimidate (PMBTCA, 6.42 mL, 30.6 mmol, 1.5 equiv.) and Sc(OTf)$_3$ (350 mg, 0.71 mmol, 0.035 equiv.). After stirring for 5 min, sat. NaHCO$_3$ solution (180 mL) was added and the layers were separated. The aqueous layer was extracted three times with diethyl ether (150 mL × 3), the combined organic layers were dried (Na$_2$SO$_4$) and the solvent was removed *in vacuo*. The crude product was purified by flash chromatography (4:1 petroleum ether / EtOAc) to yield (*Z*)-((5-bromo-3-(((4-methoxybenzyl)oxy)methyl)hexa-2,5-dien-1-yl)oxy)(*tert*-butyl)dimethylsilane **S2** (6.9 g, 15.6 mmol, 77%) as light yellow oil.

R_f 0.52 (4:1 petroleum ether / EtOAc); **ν_{max} (thin film)/cm^{-1}** 2954 (s), 2932 (s), 2903 (s), 2856 (s), 1738 (m), 1613 (s), 1586 (m); **^{1}H NMR (500 MHz, CDCl$_3$)** δ_H 7.25 (2H, d, *J* = 8.5 Hz, Ar), 6.87 (2H, d, *J* = 8.5 Hz, Ar), 5.65 (1H, t, *J* = 6.2 Hz, H2), 5.62 (1H, d, *J* = 1.3 Hz, H6), 5.48 (1H, d, *J* = 1.3 Hz, H6), 4.38 (2H, s, C$\underline{H}_2$Ar), 4.23 (2H, d, *J* = 6.2 Hz, H1), 3.97 (2H, s, H7), 3.80 (3H, s, OMe), 3.23 (2H, s, H4), 0.89 (9H, s, Si*t*-Bu), 0.06 (6H, s, SiMe$_2$); **^{13}C NMR (125 MHz, CDCl$_3$)** δ_C 159.3, 133.1, 132.7, 131.7, 130.4, 129.5, 118.8, 113.9, 71.9, 65.9, 59.7, 55.4, 46.9, 26.0, 18.4, –4.9; **HRMS (ESI$^+$)** calc. for $C_{21}H_{33}O_3BrSiNa$ ($[M+Na]^+$): 463.1275; found: 463.1274. Data in accordance with literature.[1]

(*Z*)-5-Bromo-3-(((4-methoxybenzyl)oxy)methyl)hexa-2,5-dien-1-ol, 18

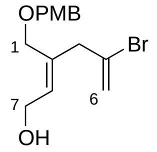

To a stirred solution of **S2** (6.9 g, 15.6 mmol, 1.0 equiv.) in methanol (300 mL) was added camphorsulfonic acid (371 mg, 1.6 mmol, 0.1 equiv.). The reaction mixture was stirred for 20 min, then Et$_3$N (230 µL, 1.6 mmol, 0.1 equiv.) was added and the solvent was removed *in vacuo*. The crude product was purified by flash

chromatography (1:1 petroleum ether / EtOAc) to afford **18** (4.49 g, 13.7 mmol, 88%) as a light yellow oil.

R_f 0.43 (1:1 petroleum ether / EtOAc); **ν$_{max}$ (thin film)/cm^{-1}** 3406 (br, OH), 2909 (s), 2862 (s), 2837 (s), 1625 (s), 1612 (s), 1513 (s); **^{1}H NMR (500 MHz, $CDCl_3$)** δ_H 7.25 (2H, d, *J* = 8.7 Hz, Ar), 6.88 (2H, d, *J* = 8.7 Hz, Ar), 5.79 (1H, t, *J* = 6.8 Hz, H2), 5.62 (1H, d, *J* = 1.5 Hz, H6), 5.49 (1H, d, *J* = 1.5 Hz, H6), 4.42 (2H, s, C$\underline{H_2}$Ar), 4.18 (2H, d, *J* = 6.4 Hz, H1), 4.00 (2H, s, H7), 3.80 (3H, s, OMe), 3.23 (2H, s, H4), 1.78 (1H, t, *J* = 5.9 Hz, OH); **^{13}C NMR (125 MHz, $CDCl_3$)** δ_C 159.4, 135.4, 132.0, 131.3, 129.9, 129.6, 119.1, 114.0, 72.3, 66.3, 58.9, 55.4, 47.5; **HRMS (ESI$^+$)** calc. for $C_{15}H_{19}O_3BrNa$ ([M+Na]$^+$): 349.0410; found: 349.0406. Data in accordance with literature.[1]

((2*R*,3*S*)-3-(2-Bromoallyl)-3-((4-methoxybenzyloxy)methyl)oxiran-2-yl)methanol, (+)-S3

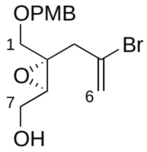

Activated 4 Å molecular sieves (645 mg, 30 w/w%) were heated under vacuum for 5 min in a Schlenk tube and cooled under Argon. CH_2Cl_2 (20 mL) and Ti(O*i*-Pr)$_4$ (2.00 mL, 6.57 mmol, 1.0 equiv.) was added and the solution was cooled to –30 °C, then D-(–)-diethyl tartrate (1.40 mL, 7.88 mmol, 1.2 equiv.) was added dropwise. After stirring the reaction mixture for 30 min at –30 °C, a solution of **18** (2.15 g, 6.57 mmol, 1.0 equiv.) in CH_2Cl_2 (7 mL) was added dropwise, followed again by stirring for 30 min at –30 °C. *Tert*-butyl hydroperoxide (5.5 M in hexanes, 3.60 mL, 19.7 mmol, 3.0 equiv.) was added dropwise and the reaction flask was placed in a freezer at –20 °C. After 19 h the reaction was cooled down to –30 °C and a solution of tartaric acid (2.96 g, 19.7 mmol, 3 equiv.) and $FeSO_4$•$7H_2O$ (16.4 g, 59.1 mmol, 9.0 equiv.) in water (20 mL) was poured in, with vigorous stirring of the reaction mixture. After warming slowly to rt (~1 h), water (10 mL) was added, the layers were separated and the aqueous layer was extracted three times with CH_2Cl_2 (25 mL × 3). The combined organic layers were dried (Na_2SO_4) and the solvent was removed *in vacuo*. Et_2O (26 mL) was added to the crude product, which was cooled to 0 °C, then a solution of NaOH (0.75 M in brine, 32 mL) was added dropwise. After stirring for 1.5 h at 0 °C, water (10 mL) was added, the layers were separated and the aqueous layer was extracted three times with Et_2O (40 mL × 3). The combined organic layers were dried (Na_2SO_4) and the solvent was removed *in vacuo*. The crude product was purified by flash chromatography (1:1 petroleum ether / EtOAc) to afford **S3** (2.19 g, 6.38 mmol, 97%) as a highly viscous yellow oil.

R_f 0.38 (1:1 petroleum ether / EtOAc); **$[\alpha]_D^{25}$** +9.3 (c 1.04, $CHCl_3$); **ν$_{max}$ (thin film)/cm^{-1}** 3454 (br, OH), 3053 (m), 2962 (s), 2906 (s), 1612 (m), 1513 (s); **^{1}H NMR (400 MHz, $CDCl_3$)** δ_H 7.24 (2H, d, *J* = 8.5 Hz, Ar), 6.87 (2H, d, *J* = 8.5 Hz, Ar), 5.68 (1H, s, H6), 5.53 (1H, d, *J* = 1.3 Hz, H6), 4.50 (1H, d, *J* = 11.4 Hz, CH_2Ar), 4.40 (1H, d, *J* = 11.4 Hz, CH_2Ar), 3.79 (3H, s, OMe), 3.72-3.66 (2H, m, H1), 3.61 (1H, d, *J* = 10.9 Hz, H7), 3.59 (1H, d, *J* = 10.9 Hz, H7), 3.19 (1H, t, *J* = 5.8 Hz, H2), 3.02 (1H, d, *J* = 14.9 Hz, H4), 2.60 (1H, d, *J* = 14.9 Hz, H4), 2.32-2.27 (1H, m, OH); **^{13}C NMR (100 MHz, $CDCl_3$)** δ_C 159.6, 129.7, 129.5, 127.8, 120.8, 114.1, 73.3, 69.1, 61.5, 61.0, 60.9, 55.4, 45.9; **HRMS (ESI$^+$)** calc. for $C_{15}H_{19}O_4BrNa$ ([M+Na]$^+$):

365.0359; found: 365.0360; ***ee***: 93% as determined by chiral HPLC: CHIRALPAK IA, 5 mL/min, 5% IPA/hexane. Data in accordance with the literature.[1]

(2*S*,3*R*)-2-Allyl-5-bromo-3-((4-methoxybenzyloxy)methyl)hex-5-ene-1,3-diol, 19

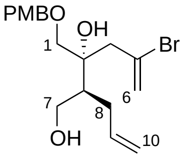

To a stirred solution of **S3** (4.19 g, 12.16 mmol, 1.0 equiv.) in THF (100 mL) at 0 °C was added allylmagnesium chloride (1.7 M in THF, 28.6 mL, 48.64 mmol, 4.0 equiv.) dropwise. The reaction mixture was stirred for 10 min, then it was quenched with NH_4Cl (10 mL, aq., sat.). The resulting suspension was filtered through a pad of celite, and the solvent was removed in vacuo. The crude product was purified by flash chromatography on (1:1 petroleum ether / EtOAc) to afford **19** (4.07 g, 2.96 mmol, 87%) as a slightly yellow, highly viscous oil.

R_f 0.47 (1:1 petroleum ether / EtOAc); $[\alpha]_D^{25}$ –4.9 (*c* 1.05, $CHCl_3$); **ν_{max} (thin film)/cm^{-1}** 3531 (br, OH), 3054 (s), 2985 (s), 2936 (s), 2305 (m), 1613 (s), 1514 (s); ^{1}H NMR (500 MHz, $CDCl_3$) δ_H 7.25 (2H, d, *J* = 8.5 Hz, Ar), 6.89 (2H, d, *J* = 8.5 Hz, Ar), 5.83-5.73 (1H, m, H9), 5.71 (1H, s, H6), 5.63 (1 H, s, H6), 5.05 (1H, d, *J* = 16.9 Hz, H10), 5.03 (1H, d, *J* = 11.6 Hz, H10), 4.49 (1H, d, *J* = 11.6 Hz, CH_2Ar), 4.48 (1H, d, *J* = 11.6 Hz, CH_2Ar), 3.81 (3H, s, OMe), 3.82-3.77 (1H, m, H1), 3.74-3.69 (1H, m, H1), 3.53 (1H, d, *J* = 9.3 Hz, H7), 3.43 (1H, d, *J* = 9.3 Hz, H7), 3.13 (1H, s, OH-3), 3.10-3.07 (1H, m, H2), 2.91 (1H, d, *J* = 15.0 Hz, H4), 2.81 (1H, d, *J* = 15.0 Hz, H4), 2.25-2.16 (1H, m, H8), 2.02-1.93 (2H, m, H8 and OH-1); **^{13}C NMR (125 MHz, $CDCl_3$)** δ_C 159.5, 137.1, 129.6, 129.5, 127.5, 121.9, 116.7, 114.0, 77.0, 73.3, 72.0, 61.6, 55.4, 47.3, 45.0, 30.8; **HRMS (ESI$^+$)** calc. for $C_{18}H_{25}O_4BrNa$ ($[M+Na]^+$): 407.0828; found: 407.0827. Data in accordance with the literature.[1]

(2*R*,3*R*)-2-Allyl-5-bromo-3-hydroxy-3-(((4-methoxybenzyl)oxy)methyl)hex-5-enoic acid, S4

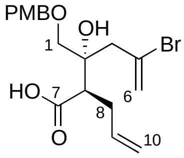

To a stirred solution of **19** (1.02 g, 2.65 mmol, 1.0 equiv.) in CH_2Cl_2 (80 mL) was added $NaHCO_3$ (222 mg, 2.52 mmol, 0.95 equiv.), followed by Dess-Martin periodinane (1.69 g, 3.40 mmol, 1.5 equiv.). The reaction mixture was stirred for 1 h, then it was quenched with $NaHCO_3$ (40 mL, aq., sat.) and $Na_2S_2O_3$ (40 mL, aq., sat.). The mixture was stirred vigorously until the layers became clear (~15 min). The layers were then separated, and the aqueous layer was extracted three times with CH_2Cl_2 (70 mL × 3). The combined organic layers were dried (Na_2SO_4) and the solvent was removed *in vacuo*. The crude product was used without purification in the following Pinnick oxidation. The crude aldehyde (2.65 mmol, 1 equiv.) was dissolved in *t*-BuOH (40 mL) and 2-methyl-2-butene (8.4 mL, 79.5 mmol, 30 equiv.) was added. Into the reaction mixture was poured a solution of sodium chlorite (80%, 2.99 g, 26.5 mmol, 10 equiv.) and NaH_2PO_4 (3.38 g, 21.2

mmol, 8 equiv.) in water (16 mL). After stirring overnight, the reaction was diluted with brine, and the layers were separated. The aqueous layer was extracted four times with EtOAc. The combined organic layers were dried (Na_2SO_4) and the solvent was removed *in vacuo*. The crude product was purified by flash chromatography (1:1 petroleum ether / EtOAc) to yield **S4** (1.06 g, 2.65 mmol, 100%) as a colourless highly viscous oil.

***R**f* 0.35 (1:1 petroleum ether / EtOAc); $[\alpha]_D^{25}$ –2.1 (c 1.04, $CHCl_3$); **ν_{max} (thin film)/cm^{-1}** 3412 (br, OH), 3077 (s), 2979 (s), 2957 (s), 2933 (s), 2874 (s), 1707 (s), 1613 (s), 1514 (s); **^{1}H NMR (500 MHz, CDCl$_3$)** δ_H 7.24 (2H, d, *J* = 9.1 Hz, Ar), 6.88 (2H, d, *J* = 9.1 Hz, Ar), 5.80-5.71 (1H, m, H9), 5.70 (1 H, s, H6), 5.63 (1H, d, *J* = 1.5 Hz, H6), 5.09 (1 H, dd, *J* = 17.0 and 1.5 Hz, H10), 5.03 (1H, d, *J* = 10.2 Hz, H10), 4.48 (1H, d, *J* = 11.7 Hz, CH_2Ar), 4.42 (1H, d, *J* = 11.7 Hz, CH_2Ar), 3.80 (3H, s, OMe), 3.57 (1H, d, *J* = 9.4 Hz, H7), 3.46 (1H, d, *J* = 9.4 Hz, H7), 3.19 (1H, s, OH), 2.99 (1H, d, *J* = 14.9 Hz, H4), 2.91-2.88 (1H, m, H2), 2.72 (1H, d, *J* = 14.9 Hz, H4), 2.40-2.37 (2H, m, H8); **^{13}C NMR (125 MHz, CDCl$_3$)** δ_C 177.6, 159.4, 134.9, 129.6, 129.5, 126.4, 122.3, 117.3, 113.8, 74.3, 73.0, 70.9, 55.3, 51.0, 46.6, 31.1; **HRMS (ESI$^+$)** calc. for $C_{18}H_{23}O_5BrNa$ ($[M+Na]^+$): 421.0621; found: 421.0623. Data in accordance with literature.[1]

(3*R*,4*R*)-3-Allyl-4-(2-bromoallyl)-4-((4-methoxybenzyloxy)methyl)oxetan-2-one, 20

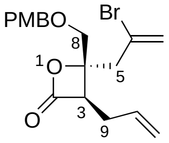

To a stirred solution of **S4** (2.46 g, 6.15 mmol, 1 equiv.) in dry acetonitrile (50 mL) was added dry pyridine (37 mL) and *bis*-(2-oxo-3-oxazolidinyl)phosphonic chloride (BOP-Cl) (4.70 g, 18.5 mmol, 3.0 equiv.). The reaction mixture was stirred for 2.5 h before it was quenched with water. The layers were separated and the aqueous layer was extracted four times with EtOAc. The combined organic layers were dried with (Na_2SO_4) and the solvent was removed *in vacuo*. The crude product was purified by flash chromatography (4:1 petroleum ether / EtOAc) to yield **20** (1.95 g, 5.11 mmol, 83%) as light yellow oil.

***R**f* 0.66 (1:1 petroleum ether / EtOAc); $[\alpha]_D^{25}$ –2.7 (c 1.05, $CHCl_3$); **ν_{max} (thin film)/cm^{-1}** 3418 (br, OH), 3079 (m), 2918 (s), 2851 (s), 2358 (w), 2254 (m), 1835 (s), 1722 (m), 1612 (s); **^{1}H NMR (500 MHz, CDCl$_3$)** δ_H 7.24 (2 H, d, *J* = 8.6 Hz, ArH), 6.88 (2H, d, *J* = 8.6 Hz, ArH), 5.88-5.80 (1H, m, H10), 5.77 (1H, s, H7), 5.65 (1H, d, *J* = 1.7 Hz, H7), 5.13 (1H, ddd, *J* = 17.1, 2.8 and 1.5 Hz, H11), 5.09 (1H, ddd, *J* = 10.2, 2.6 and 1.5 Hz, H11), 4.52 (1H, d, *J* = 11.6 Hz, CH_2Ar), 4.49 (1H, d, *J* = 11.6 Hz, CH_2Ar), 3.81 (3H, s, OMe), 3.77-3.69 (3H, m, 2 x H8 and H3), 3.21 (1H, d, *J* = 14.9 Hz, H5), 2.93 (1H, d, *J* = 14.9 Hz, H5), 2.60-2.49 (2H, m, H9); **^{13}C NMR (125 MHz, CDCl$_3$)** δ_C 170.0, 159.5, 134.0, 129.6, 129.4, 125.0, 123.0, 117.6, 114.0, 80.5, 73.6, 69.3, 56.1, 55.4, 46.4, 28.3; **HRMS (ESI$^+$)** calc. for $C_{18}H_{21}O_4BrNa$ ($[M+Na]^+$): 403.0515; found: 403.0512. Data in accordance with literature.[1]

(3*S*,4*R*)-3-Allyl-6-bromo-4-((4-methoxybenzyloxy)methyl)-2-methylhept-6-ene-2,4-diol, 21 and (*R*)-3-Allyl-6-bromo-4-hydroxy-4-((4-methoxybenzyloxy)methyl)hept-6-en-2-one, 22

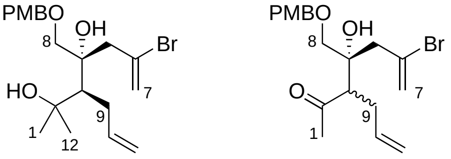

To a stirred solution of **20** (1.93 g, 5.06 mmol, 1 equiv.) in THF (10.5 mL) at –50°C was added dropwise MeMgBr (3 M in Et_2O, 10.12 mL, 30.36 mmol, 6.0 equiv.). The reaction mixture was warmed slowly to rt (6 h) and was then hydrolysed with NH_4Cl (5 mL, aq., sat.). The resulting suspension was filtered through a pad of celite, and the solvent was removed *in vacuo*. The crude product was purified by flash column chromatography on silica gel (10:1→4:1 petroleum ether / EtOAc) to yield **21** (1.37 g, 0.73 mmol, 66%) as colourless oil, along with the ketone **22** (480 mg, 24%, mixture of C3-epimers, ratio of **22** : **3-*epi*-22** = 5:3) as a light yellow oil.

Characterization for 21: ***R**_f*** 0.58 (1:1 Petroleum Ether / EtOAc); $[\alpha]_D^{25}$ –20.4 (c 1.01, $CHCl_3$); **ν_{max} (thin film)/cm^{-1}** 3358 (br, OH), 3074 (m), 2972 (s), 2934 (s), 2873 (s), 2836 (s), 1613 (s), 1513 (s); **^{1}H NMR (500 MHz, $CDCl_3$)** δ_H 7.25 (2H, d, *J* = 8.6 Hz, Ar), 6.89 (2H, d, *J* = 8.6 Hz, Ar), 5.85-5.77 (1H, m, H10), 5.75 (1H, s, H7), 5.63 (1H, d, *J* = 1.4 Hz, H7), 5.00-4.95 (2H, m, H11), 4.51 (1H, d, *J* = 11.3 Hz, CH_2Ar), 4.46 (1H, d, *J* = 11.3 Hz, CH_2Ar), 4.22 (1H, br, OH), 3.81 (3H, s, OMe), 3.80 (1H, d, *J* = 9.3 Hz, H8), 3.53 (1H, d, *J* = 9.3 Hz, H8), 2.94 (1H, d, *J* = 15.1 Hz, H5), 2.72 (1H, d, *J* = 15.1 Hz, H5), 2.18-2.04 (3H, m, H3 and H9), 1.26 (3H, s, H1), 1.23 (3H, s, H12); **^{13}C NMR (125 MHz, $CDCl_3$)** δ_C 159.5, 139.6, 129.6, 129.5, 127.8, 121.8, 115.4, 114.0, 78.1, 74.9, 73.3, 72.3, 55.4, 52.9, 49.7, 32.5, 32.2, 26.8; **HRMS (ESI$^+$)** calc. for $C_{20}H_{29}O_4BrNa$ ($[M+Na]^+$): 435.1141; found: 435.1130. Data in accordance with literature.[1]

Characterization for 22 (major = *= **22**; minor = no* = **3-*epi*-22**): ***R**_f*** 0.67 (1:1 petroleum ether / EtOAc); **ν_{max} (thin film)/cm^{-1}** 3531 (br, OH), 3054 (s), 2985 (s), 2936 (s), 2305 (m), 1613 (s), 1514 (s); **^{1}H NMR (500 MHz, $CDCl_3$)** δ_H 7.23-7.21 (4H, m, H^*_{Ar} and H_{Ar}), 6.88-6.86 (4H, m, H^*_{Ar} and H_{Ar}), 5.73-5.74 (4H, m, H10*, H7*, H10 and H7), 5.60 (1H, d, *J* = 1.5 Hz, H7*), 5.59 (1H, d, *J* = 1.3 Hz, H7), 5.05-4.97 (4H, m, H11* and H11), 4.47-4.37 (4H, m, CH_2*Ar and CH_2Ar), 3.79 (6H, s, OCH_3* and OCH_3), 3.55 (1H, s, OH), 3.53-3.44 (2H, m, H8* and H8), 3.38 (1H, d, *J* = 9.4 Hz, H8*), 3.34 (1H, *J* = 9.6 Hz, H8), 3.25 (1H, s, OH*), 3.09 (1H, dd, *J* = 9.5 and 5.0 Hz, H3*), 3.05 (1H, dd, *J* = 9.8 and 5.0 Hz, H3), 2.96 (1H, d, *J* = 14.9 Hz, H5*), 2.84 (1H, d, *J* = 14.7 Hz, H5), 2.69 (1H, d, *J* = 14.7 Hz, H5), 2.65 (1H, d, *J* = 14.9 Hz, H5*), 2.40-2.24 (4H, m, H9* and H9), 2.17 (3H, s, H1*), 2.12 (3H, s, H1); **^{13}C NMR (125 MHz, $CDCl_3$)** δ_C 214.6 (C2), 213.6 (C2*), 159.4 (C_{Ar}* and C_{Ar}), 135.4 (C_{Ar}* and C_{Ar}), 135.2 (C10*), 129.8 (C_{Ar}* and C_{Ar}), 129.7 (C-10), 129.6 (C_{Ar}* and C_{Ar}), 127.2 (C6*), 127.0 (C6), 122.1 (C7), 121.9 (C7*), 117.5 (C11), 117.2 (C11*), 113.9 (C_{Ar}*) 113.8 (C_{Ar}), 75.2 (C4*), 73.5 (C8), 73.1 (C4), 73.0 ($C_{Ar}\underline{C}H_2$* and $C_{Ar}\underline{C}H_2$), 71.3 (C8*), 55.6 (C3*), 55.3 (OCH_3* and OCH_3), 55.1 (C3), 46.7 (C5*) 45.9 (C5), 34.3 (C1*) 33.9 (C1), 31.8 (C9), 31.6 (C9*); **HRMS (ESI$^+$)** calc. for $C_{19}H_{25}O_4BrNa$ ($[M+Na]^+$): 419.0828; found: 419.0827.

Compound **21** provides a convenient resting stage for completion of the AB rings, and was taken forward as required on only moderate scales.

(*Z*)-4-((*tert*-butyldimethylsilyl)oxy)-2-(3-hydroxyprop-1-en-2-yl)but-2-en-1-yl methyl carbonate, 28 and (*Z*)-5-((*tert*-butyldimethylsilyl)oxy)-3-(hydroxymethyl)-2-methylenepent-3-en-1-yl methyl carbonate, 29

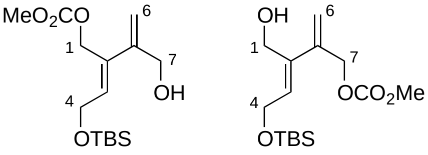

28 (major) **29** (minor)

To a solution of stannane **16** (113.0 mg, 0.23 mmol, 1.0 equiv.) in degassed toluene (1.77 mL) was added Pd(dba)$_2$ (5.3 mg, 9.2 μmol, 4 mol%) and 2-bromoallyl methyl carbonate (133.8 mg, 0.69 mmol, 3 equiv.). The resulting purple solution was heated at 70 °C for 16 h (the solution turned to a greenish brown after 15 min. of heating). The reaction mixture was then cooled down to room temperature and concentrated. The resulting crude was then purified by flash chromatography on (8:2 petroleum ether / EtOAc using 10 wt% crushed K_2CO_3 in silica) to afford an inseparable mixture of carbonates **28** and **29** (1:0.86, 23.0 mg, 0.073 mmol, 32%) as a colourless oil.

R$_f$ 0.26 (8:2 petroleum ether / EtOAc); **ν$_{max}$ (thin film) /cm^{-1}** 3412.5 (br), 2956.0, 2857.8, 2360.4, 1749.4, 1444.1, 1261.7, 837.9, 783.; **HRMS (ESI$^+$)** calc. for $C_{15}H_{28}O_5NaSi$ [M+Na]$^+$ 339.15982, found 339.16000

Characterisation for 28: ^{1}H NMR (400 MHz, CDCl$_3$) δ$_H$ 5.96 (1H, t, *J* = 6.1 Hz, H3), 5.29 (1H, s, H6), 5.27 (1H, s, H6), 4.86 (2H, s, H1), 4.43 (2H, d, *J* = 6.1 Hz, H4), 4.32 (2H, s, H7), 3.77 (3H, s, HOMe), 0.90 (9H, s, H*t*Bu), 0.08 (6H, s, HSiMe$_2$); **^{13}C NMR (101 MHz, CDCl$_3$)** δ$_C$ 155.8, 145.5, 134.5, 131.9, 113.8, 64.4, 63.4, 60.1, 55.0, 26.0, 18.4, -5.1.

Characterisation for 29: ^{1}H NMR (400 MHz, CDCl$_3$) δ$_H$ 5.85 (1H, t, *J* = 6.1 Hz, H3), 5.45 (1H, s, H6), 5.33 (1H, s, H6), 4.82 (2H, s, H7), 4.37 (2H, d, *J* = 6.1 Hz, H4), 4.32 (2H, s, H1), 3.78 (3H, s, HOMe), 0.90 (9H, s, H*t*Bu), 0.09 (6H, s, HSiMe$_2$); **^{13}C NMR (101 MHz, CDCl$_3$)** δ$_C$ 155.7, 141.4, 138.7, 130.8, 116.3, 68.7, 59.8, 58.9, 55.0, 26.0, 18.4, -5.1.

(4*R*)-4-((*R*)-4-bromo-2-hydroxy-1-((4-methoxybenzyl)oxy)pent-4-en-2-yl)-5,5-dimethyltetrahydrofuran-2-ol, 33

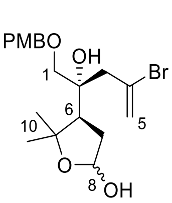

33

To a stirred solution of alkene **21** (418 mg, 1.01 mmol, 1.0 equiv.) in 1,4-dioxane (10 mL) and water (2.5 mL) was sequentially added 2,6-lutidine (230 µL, 2.02 mmol, 2.0 equiv.), osmium(VII) tetroxide (4% wt in water, 130 µL, 0.02 mmol, 0.02 equiv.) and sodium periodate (864 mg, 4.04 mmol, 4.0 equiv.). The reaction

mixture was stirred at RT for 2 h before being diluted with water (10 mL) and diethyl ether (10 mL). The layers were separated and the aqueous layer extracted three times with diethyl ether (15 mL × 3). The combined organic layers were dried with $MgSO_4$ and the solvent removed carefully *in vacuo*. The crude product was filtered through a short plug of silica (7:3 Pet. Ether/ EtOAc), then concentrated to yield lactol **33** (353 mg, 0.838 mmol, 83%, inseparable mixture of epimers and some regional isomers) as a colourless oil, which was used in the next step without further purification. **R_f** 0.36 (Pet. Ether/ EtOAc (1:1)); **IR** (thin film, ν_{max} / cm^{-1}) 3422 (br), 2932 (m), 1613 (m), 1514 (s), 1463 (m), 1368 (m), 1249 (2), 1090 (s), 1034 (s), 818 (m); **HRMS** (ESI^+) calc. for $C_{19}H_{27}O_5BrNa$ $[M+Na]^+$ 437.0934, found 437.0921.

(2*R*)-4-Bromo-2-((3*R*)-5-methoxy-2,2-dimethyltetrahydrofuran-3-yl)pent-4-ene-1,2-diol, 34

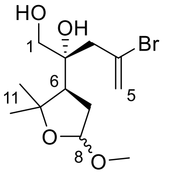

34

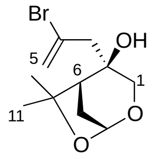

Intermediate acetal

To a stirred solution of PMB ether **33** (352 mg, 0.85 mmol) in dichloromethane (42.5 mL) at 0 °C was added TFA (4.7 mL) dropwise. The reaction mixture was stirred for 15 min at 0 °C before being quenched with $NaHCO_3$ solution (30 mL, sat. *aq.*). The layers were separated and the aqueous layer extracted three times with dichloromethane (20 mL × 3). The combined organic layers were dried with $MgSO_4$ and the solvent removed carefully *in vacuo*. The crude product was purified by flash column chromatography on a short plug of silica (7:3 Pet. Ether/ EtOAc), to yield an intermediate acetal (212 mg) as a colourless oil. **R_f** 0.54 (Pet. Ether/ EtOAc (1:1)); **IR** (thin film, ν_{max} / cm^{-1}) 3428 (br), 2927 (m), 1738 (w), 1624 (m), 1468 (m), 1292 (m), 1119 (m), 1089 (s), 1030 (s), 881 (s); **^{1}H NMR** (500 MHz, $CDCl_3$) δ_H 5.76 (1H, t, *J* = 1.6 Hz, H5), 5.70 (1H, d, *J* = 1.6 Hz, H5), 5.28 (1H, d, *J* = 3.2 Hz, H8), 4.01 (1H, d, *J* = 11.3 Hz, H1), 3.75 (1H, br s, OH), 3.53 (1H, d, *J* = 11.3 Hz, H1), 3.23 (1H, d, *J* = 15.1 Hz, H3), 2.75 (1H, dd, *J* = 15.1, 1.0 Hz, H3), 2.39 (1H, s, OH), 2.24 (1H, ddd, *J* = 12.6, 5.0, 3.2 Hz, H7), 2.17 (1H, d, *J* = 5.0 Hz, H6), 1.89 (1H, d, *J* = 12.6 Hz, H7), 1.69 (3H, s, H11), 1.28 (3H, s, H11); **^{13}C NMR** (126 MHz, $CDCl_3$) δ_C 127.6, 122.5, 98.3, 84.2, 72.4, 68.4, 50.9, 48.4, 36.0, 30.5, 25.0; The data for this compound is in accordance with the literature, however we now suggest its structure to be an acetal, and not a diol as originally proposed,[1] due to its relatively high R_f value compared to that of compounds **33** and **34**, and its formation as a single diastereomer.

The stirred solution of the acetal (212 mg, 0.718 mmol, 1.0 equiv.) in methanol (30 mL) was cooled to 0 °C. To this solution, (±)-CSA (17 mg, 0.072 mmol, 0.1 equiv.) was added. The reaction mixture was stirred at this temperature for 2.5 h before being quenched with $NaHCO_3$ (5 mL, sat. *aq.*). The solvent was removed carefully *in vacuo,* and the residue was diluted with Et_2O (10 mL). Water was added (5 mL), the layers separated and the aqueous layer extracted three times with Et_2O (10 mL × 3). The combined organic layers were dried with $MgSO_4$, and concentrated. The crude product was purified by flash chromatography using a short plug of SiO_2 (7:3→3:2 Pet. Ether / EtOAc), to yield the methyl acetals **34** (194 mg, 0.181 mmol, 75% over two steps, as a 2:1 mixture of acetal epimers) as a colourless oil, together with recovered acetal starting

material (29 mg, 0.086 mmol, 12%). These anomers were generally not readily separated by chromatography, and they were generally carried forward to bromoene AB rings **14**; the data presented below was obtained for the purpose of characterization by careful chromatography. **R_f** 0.37 (Pet. Ether/ EtOAc (1:1)); **IR** (thin film, ν_{max} / cm^{-1}) 3455 (br), 2971 (m), 1739 (m), 1625 (m), 1371 (m), 1229 (m), 1103 (m), 1042 (s), 979 (m); **HRMS** (ESI^+) calc. for $C_{12}H_{21}O_4BrNa$ $[M+Na]^+$ 331.0508; found 331.0515. **Major epimer: ^{1}H NMR** (500 MHz, $CDCl_3$) δ_H 5.77 (1H, br s, H5), 5.68 (1H, d, *J* = 1.3 Hz, H5), 4.86 (1H, d, *J* = 5.0 Hz, H8), 3.65 (1H, dd, *J* = 18.0 and 11.0 Hz, H1), 3.58-3.51 (1H, m, H1), 3.31 (3H, s, OCH_3), 2.93 (1H, d, *J* = 14.8 Hz, H3), 2.84 (1H, d, *J* = 14.8 Hz, H3), 2.48 (1H, dd, *J* = 12.6 and 6.6 Hz, H6), 2.40 (1H, s, OH), 2.18 (1H, *app* td, *J* = 12.6 and 5.0 Hz, H7), 1.96 (1H, dd, *J* = 12.6 and 6.6 Hz, H7), 1.43 (3H, s, H11), 1.32 (3H, s, H11'); **^{13}C NMR** (126 MHz, $CDCl_3$) δ_C 127.6, 122.4, 102.0, 83.7, 74.4, 66.0, 54.2, 50.1, 47.3, 34.6, 32.7, 25.8. **Minor epimer: ^{1}H NMR** (500 MHz, $CDCl_3$) δ_H 5.77 (1H, br s, H5), 5.67 (1H, d, *J* = 1.3 Hz, H5), 4.94 (1H, dd, *J* = 6.1 and 3.9 Hz, H8), 3.65 (1H, dd, *J* = 18.0 and 11.0 Hz, H1), 3.58-3.51 (1H, m, H1), 3.35 (3H, s, OCH_3), 2.89 (1H, d, *J* = 14.8 Hz, H3), 2.85 (1H, d, *J* = 14.8 Hz, H3), 2.67 (1H, s, OH), 2.36 (1H, ddd, *J* = 13.2, 8.8 and 6.1 Hz, H7), 2.27 (1H, *app* t, *J* = 9.3 Hz, H6), 2.10 (1H, ddd, *J* = 13.2, 9.8 and 3.9 Hz, H7), 1.43 (3H, s, H11), 1.36 (3H, s, H11); **^{13}C NMR** (126 MHz, $CDCl_3$) δ_C 127.9, 122.2, 103.2, 83.3, 74.4, 66.0, 55.3, 52.0, 47.8, 34.6, 31.2, 25.6. Data in accordance with the literature.[1]

(2*R*)-4-Bromo-2-hydroxy-2-((3*R*)-5-methoxy-2,2-dimethyltetrahydrofuran-3-yl)pent-4-enal, 35

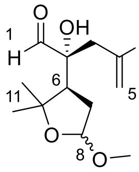

35

Dry DMSO (1.88 mL, 26.42 mmol, 42 equiv.) was added to SO_3•py (802 mg, 5.02 mmol, 8.0 equiv.) under argon, and the suspension was stirred at RT for 15 min. Dichloromethane (20 mL) was added, then the mixture was cooled to 0 °C and stirred for a further 10 min. A solution of diol **34** (194 mg, 0.63 mmol, 1.0 equiv.) in dry dichloromethane (3.0 mL), and *i*-$PrEt_2N$ (2.2 mL, 12.6 mmol, 20 equiv.) were added simultaneously, and the resulting mixture stirred for 1 h between 0 and 10 °C. The reaction was then quenched with NH_4Cl (10 mL, sat. *aq.*). Et_2O (15 mL) and water (10 mL) was added, the phases were separated, and the aqueous layer extracted three times with Et_2O (10 mL × 3). The combined organic layers were washed sequentially with $NaHCO_3$ (sat. *aq.*), followed by brine. The organic layer was dried ($MgSO_4$) and concentrated. The crude product was purified by flash chromatography through a short plug of silica (5:1 Pet. Ether / EtOAc), to afford aldehyde **35** (153 mg, 0.498 mmol, 79%, 2:1 mixture of C8-epimers) as a white solid. These epimers were generally not readily separated by chromatography, and they were generally carried forward bromoene AB rings **104**; the data presented below was obtained for the purpose of characterization by careful chromatography. **IR** (thin film, ν_{max} / cm^{-1}) 3485 (br), 2976 (m), 1729 (s), 1627 (m), 1368 (m), 1327 (m), 1226 (m), 1103 (s), 1045 (s), 978 (m), 813 (m); **HRMS** (ESI^+) calc. for $C_{12}H_{19}O_4BrNa$ $[M+Na]^+$ 329.0359; found 329.0363. **Major epimer: R_f** 0.39 (7:3 Pet. Ether / EtOAc); **^{1}H NMR** (500 MHz, $CDCl_3$) δ_H 9.77 (1H, s, H1), 5.70 (1H, s, H5), 5.59 (1H, d, *J* = 1.9 Hz, H5), 4.86 (1H, d, *J* =

4.7 Hz, H8), 3.51 (1H, br s, OH), 3.31 (3H, s, OCH_3), 3.11 (1H, d, *J* = 14.8 Hz, H3), 3.02 (1H, d, *J* = 14.8 Hz, H3), 2.59 (1H, dd, *J* = 13.2 and 6.4 Hz, H6), 2.11 (1H, *app* td, *J* = 12.9 and 4.7 Hz, H7), 1.74 (1H, dd, *J* = 12.6 and 6.4 Hz, H7), 1.49 (3H, s, H11), 1.22 (3H, s, H11'); **^{13}C NMR** (126 MHz, $CDCl_3$) δ_C 202.2, 124.7, 123.2, 102.1, 83.8, 79.7, 54.2, 50.5, 48.6, 34.3, 32.7, 25.8. **Minor epimer: R_f** 0.58 (7:3 Pet. Ether / EtOAc); **^{1}H NMR** (500 MHz, $CDCl_3$) δ_H 9.88 (1H, s, H1), 5.71 (1H, s, H5), 5.59 (1H, d, *J* = 1.9 Hz, H5), 4.97-4.95 (1H, m, H8), 3.73 (1H, br s, OH), 3.34 (3H, s, OCH_3), 3.05 (1H, d, *J* = 14.5 Hz, H3), 2.99 (1H, d, *J* = 14.5 Hz, H3), 2.33-2.25 (2H, m, H7 and H6), 2.14-2.07 (1H, m, H7), 1.40 (3H, s, H11),1.28 (3H, s, H11'); **^{13}C NMR** (126 MHz, $CDCl_3$) δ_C 201.8, 124.6, 123.2, 103.2, 83.2, 79.8, 55.3, 53.0, 49.0, 34.6, 30.9, 25.5. Data in accordance with the literature.[1]

(5*S*)-5-(2-Bromoallyl)-5-((3*S*)-5-hydroxy-2,2-dimethyltetrahydrofuran-3-yl)furan-2(5*H*)-one, 36

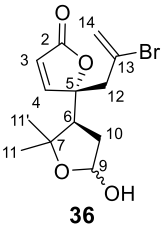

36

To a stirred solution of anhydrous ethyl 2-(diphenoxyphosphoryl)acetate (319 mg, 0.996 mmol, 2.0 equiv.) in THF (10.0 mL) under argon at 0 °C was added KHMDS (0.5 M in toluene, 1.89 mL, 0.946 mmol, 1.9 equiv.) and the resulting mixture was stirred at 0 °C for 20 min. This solution was then added dropwise to a solution of aldehyde **35** (153 mg, 0.498 mmol, 1.0 equiv.) in THF (5.0 mL) under argon at –20 °C, and the reaction mixture was stirred for 2 h between –20 and 0 °C, before being quenched with NH_4Cl (10 mL, sat. *aq.*). Et_2O (10 mL) was added, the layers were separated, and the aqueous layer was extracted three times with Et_2O (2 mL × 3). The combined organic layers were dried ($MgSO_4$) and concentrated. The product was purified by flash chromatography through a short plug of silica gel (6:1→4:1 Pet. Ether / EtOAc), to afford a colourless oil which co-eluted with the excess phosphonate ester. This mixture was used in the next step without further purification.

To a stirred solution of the crude mixture (from the previous reaction) in dichloromethane (20.0 mL) at 0 °C was added distilled water (93 µL) and TFA (1.86 mL) dropwise. The reaction mixture was stirred for 15 min at 0 °C before being quenched with $NaHCO_3$ (10 mL, sat. *aq.*). The layers were separated and the aqueous layer extracted three times with dichloromethane (20 mL × 3). The combined organic layers were dried ($MgSO_4$) and concentrated. The product was purified by flash chromatography through a short plug of silica gel (7:3→1:1 Pet. Ether / EtOAc), to afford lactol **36** (64 mg, 0.199 mmol, 40% over 2 steps, as a 2:1 mixture of C8-epimers) as a white solid. These epimers were generally not readily separated by chromatography, and they were generally carried forward bromoene AB rings **14**; the data presented below was obtained for the purpose of characterization by careful chromatography. **R_f** 0.15 (7:3 Pet. Ether / EtOAc); **IR** (thin film, ν_{max} / cm^{-1}) 3469 (br), 3002 (w), 2944 (m), 1749 (s), 1437 (m), 1367 (s), 1230 (m), 1216 (s), 1203 (m); **HRMS** (ESI$^+$) calc. for $C_{13}H_{17}BrNaO_4$ $[M+Na]^+$ 339.0202; found 339.0212. **Major epimer: ^{1}H NMR** (500 MHz, $CDCl_3$) δ_H 7.51 (1H, d, *J* = 5.7 Hz, H4), 6.10 (1H, d, *J* = 5.7 Hz, H3), 5.67

(1H, d, J = 1.6 Hz, H14), 5.60 (1H, d, J = 1.6 Hz, H14), 5.38 (1H, dd, J = 4.8 and 2.5 Hz, H8), 3.39 (1H, d, J = 14.6 Hz, H12), 2.89 (1H, d, J = 14.6 Hz, H12), 2.71 (1H, J = 12.9, 6.5 Hz, H6), 2.50 (1H, t, J = 1.9 Hz, OH), 1.92 (1H, tdd, J = 12.9, 4.8 and 1.6 Hz, H7), 1.81 (1H, dd, J = 12.9 and 6.5 Hz, H7), 1.52 (3H, s, H11), 1.24 (3 H, s, H11'); **^{13}C NMR** (126 MHz, $CDCl_3$) δ_C 172.4, 157.7, 124.2, 123.8, 121.5, 95.3, 88.4, 83.6, 51.0, 48.4, 35.8, 32.4, 25.5. **Minor epimer: ^{1}H NMR** (500 MHz, $CDCl_3$) δ_H 7.55 (1H, d, J = 5.7 Hz, H4), 6.12 (1H, d, J = 5.7 Hz, H3), 5.68 (1H, d, J = 1.8 Hz, H13), 5.61 (1H, d, J = 1.8 Hz, H13), 5.48 (1H, ddd, J = 6.2, 4.7 and 3.5 Hz, H9), 3.30 (1H, d, J = 14.5 Hz, H11), 2.84 (1H, d, J = 14.5 Hz, H11), 2.70 (1H, d, J = 3.5 Hz, OH), 2.39 (1H, dd, J = 12.0 and 8.2 Hz, H6), 2.31 (1H, ddd, J = 13.2, 8.2 and 6.2 Hz, H10), 1.87 (1H, m, H10), 1.39 (3H, s, H14), 1.32 (3H, s, H14); **^{13}C NMR** (126 MHz, $CDCl_3$) δ_C 172.1, 156.9, 124.3, 123.7, 122.0, 96.1, 88.5, 82.6, 54.3, 49.3, 36.6, 30.3, 25.9. Data in accordance with the literature.[1]

2-((3*S*,3a*R*,6a*R*)-3a-(2-bromoallyl)-2,2-dimethyl-5-oxohexahydrofuro[3,2-*b*]furan-3-yl)acetaldehyde (104)

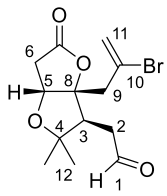

14

To a stirred solution of lactol **36** (64 mg, 0.2 mmol, 1.0 equiv.) in MeOH (8.0 mL) was added K_2CO_3 (54 mg, 0.4 mmol, 2.0 equiv.). The reaction mixture was stirred at RT for 2 h before being quenched with NH_4Cl (5.0 mL, sat. *aq.*). The layers were separated and the aqueous layer extracted three times with EtOAc (4 x 3 mL). The combined organic layers were dried (Na_2SO_4) and the solvent removed *in vacuo*. The product was purified by flash chromatography through a short plug of silica (2:1 Pet. Ether / EtOAc), to give AB ring aldehyde **14** (52 mg, 0.172 mmol, 86%) as a white solid. **R_f** 0.38 (3:2 Pet. Ether / EtOAc); **IR** (thin film, ν_{max} / cm^{-1}) 2971 (m), 1774 (s), 1740 (s), 1723 (s), 1624 (m), 1437 (m), 1375 (m), 1231 (m), 1215 (s), 1175 (m), 1021 (m), 927 (m); **^{1}H NMR** (500 MHz, $CDCl_3$) δ_H 9.78 (1H, t, J = 1.9 Hz, H1), 5.79 (1H, d, J = 1.3 Hz, H11), 5.72 (1H, d, J = 1.3 Hz, H11), 4.86 (1H, d, J = 6.6 Hz, H4), 3.05 (1H, d, J = 14.8 Hz, H9), 2.89 (1H, dd, J = 18.6 and 6.6 Hz, H6), 2.78 (1H, dd, J = 9.1 and 6.0 Hz, H3), 2.67 (1H, d, J = 18.6 Hz, H6), 2.62 (1H, d, J = 14.8 Hz, H9), 2.51 (1H, ddd, J = 16.7, 9.1 and 1.9 Hz, H2), 2.38 (1H, ddd, J = 16.7, 6.0 and 1.9 Hz, H2), 1.34 (3H, s), 1.12 (3H, s); **^{13}C NMR** (126 MHz, $CDCl_3$) δ_C 199.4 (C1), 175.2 (C7), 125.1 (C11), 123.4 (C10), 94.6 (C8), 82.7 (C4), 77.0 (C5), 53.6 (C3), 45.7 (C9), 40.8 (C2), 37.3 (C6), 27.7 (C12), 21.1 (C12); **HRMS** (ESI$^+$) calc. for $C_{13}H_{17}BrO_2Na$ [M+Na]$^+$ 339.0202; found 339.020. Data in accordance with the literature.[1]

1.2.2 Synthesis of the model CDE ring system

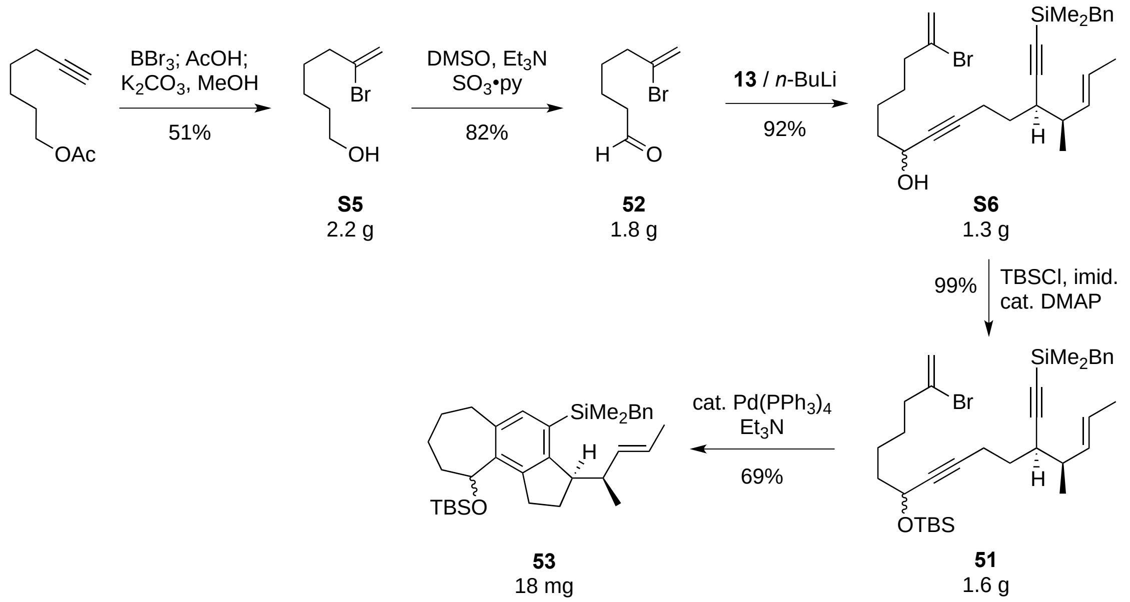

Scheme S2: Pd-catalyzed cyclization to CDE rings **53**. Indicated masses correspond to the largest scale each compound was isolated from a single experiment.

6-Bromohept-6-en-1-ol, S5

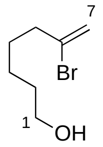

Boron tribromide (1 M in dichloromethane, 11.0 mL, 11.0 mmol, 0.5 equiv.) was cooled under argon to –78 °C, and hept-6-ynyl acetate (3.44 g, 22.0 mmol, 1.0 equiv.) was added dropwise. The reaction was rapidly warmed to room temperature and stirred for 5 h, before being quenched with acetic acid (22 mL). After stirring for 1 h, water was added, the layers separated and the aqueous layer extracted twice with dichloromethane. The combined organic layers were dried with Na_2SO_4 and the solvent removed carefully *in vacuo*. The crude 6-bromohept-6-enyl acetate was used in the next step without further purification.

To a solution of the crude acetate in wet methanol (220 mL) was added potassium carbonate (9.14 g, 66.1 mmol, 3.0 equiv.). The reaction mixture was stirred for 2 h before it was concentrated carefully *in vacuo*. Water was added, the layers separated and the aqueous layer extracted three times with diethyl ether. The combined organic layers were dried with Na_2SO_4 and the solvent removed carefully *in vacuo*. The crude product was purified by flash column chromatography on silica gel (4:1 to 2:1 Petroleum Ether / Et_2O) to yield bromoalkene **S5** (2.19 g, 11.3 mmol, 51% over 2 steps) as a pale yellow oil.

R_f 0.32 (Petroleum Ether / EtOAc (4:1)); **^{1}H NMR (400 MHz, $CDCl_3$)** δ_H 5.56-5.55 (1H, m, H7), 5.39 (1H, d, J = 1.6 Hz, H7), 3.67-3.63 (2H, m, H1), 2.43 (2H, t, J = 7.3 Hz, H5), 1.63-1.55 (4H, m, H2 and H4), 1.42-1.34 (2H, m, H3), 1.28 (1H, bs, OH); **^{13}C NMR (101 MHz, $CDCl_3$)** δ_C 134.7, 116.7, 62.9, 41.5, 32.5, 27.7, 24.6. Data in accordance with the literature.[2]

6-Bromohept-6-enal, 52

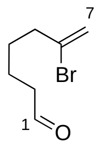

To a stirred solution of alcohol **S5** (2.15 g, 11.1 mmol) in dichloromethane (56 mL) under argon was added triethylamine (10.8 mL, 77.7 mmol, 7.0 equiv.) and DMSO (7.5 mL, 106 mmol, 9.5 equiv.). The reaction mixture was cooled to 0 °C and sulfur trioxide pyridine complex (5.30 g, 33.3 mmol, 3.0 equiv.) was added portionwise. The reaction mixture was stirred for 1 h at 0 °C before being allowed to warm to room temperature and stirred for a further 1 h. The reaction mixture was quenched by addition of 1 N HCl. The layers were separated and the aqueous layer extracted three times with dichloromethane. The combined organic layers were dried with Na_2SO_4 and the solvent removed *in vacuo*. Flash column chromatography on silica gel (6:1 Petroleum Ether / Et_2O) afforded aldehyde **52** (1.75 g, 9.16 mmol, 82%) as a pale yellow oil.
R$_f$ 0.46 (Petroleum Ether / EtOAc (9:1)); **^{1}H NMR (400 MHz, CDCl$_3$)** δ_H 9.77 (1H, t, *J* = 3.2 Hz, H1), 5.58-5.57 (1H, m, H7), 5.40 (1H, d, *J* = 1.6 Hz, H7), 2.48-2.42 (4H, m, H2 and H5), 1.69-1.55 (4H, m, H3 and H4); **^{13}C NMR (101 MHz, CDCl$_3$)** δ_C 202.3, 134.0, 117.0, 43.6, 41.2, 27.4, 20.9. Data in accordance with the literature.[2]

(12*R*,13*R*,*E*)-12-((Benzyldimethylsilyl)ethynyl)-2-bromo-13-methylhexadeca-1,14-dien-8-yn-7-ol, S6

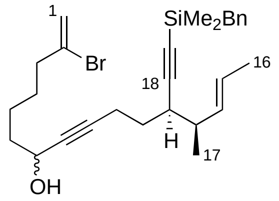

To a stirred solution of diyne **13** (871 mg, 2.82 mmol, 1.0 equiv.) in dry THF (21 mL) under argon at –78 °C was added *n*-BuLi (2.5 M in hexane, 1.13 mL, 2.82 mmol, 1.0 equiv.) dropwise. The reaction mixture was stirred for 30 min before a solution of 6-bromohept-6-enal **52** (908 mg, 4.75 mmol, 1.7 equiv.) in dry THF (1.2 mL) was added. The stirred reaction mixture was warmed over 3 h from –78 °C to –20 °C, before being quenched with NH_4Cl (2 mL, sat., *aq.*) and warmed to room temperature. The mixture was filtered through a plug of Celite® and the solvent removed *in vacuo*. The crude product was purified by flash column chromatography on silica gel (19:1 Petroleum Ether / Et_2O) to yield **S6** (1.30 g, 2.60 mmol, 92%) as a colourless oil.
R$_f$ 0.17 (Petroleum Ether / Et_2O (17:3)); **ν_{max} (thin film) /cm^{-1}** 3370 (br), 2935 (m), 2167 (m), 1630 (m), 1601 (m), 1494 (m), 1452 (m), 1250 (m), 1208 (m), 968 (m), 837 (s), 670 (m); **^{1}H NMR (500 MHz, CDCl$_3$)** δ_H 7.24-7.19 (2H, m, ArH), 7.11-7.05 (3H, m, ArH), 5.56 (1H, d, *J* = 1.5 Hz, H1), 5.44 (1H, dq, *J* = 15.1, 6.4 Hz, H15), 5.39 (1H, d, *J* = 1.5 Hz, H1), 5.31 (1H, ddq, *J* = 15.1, 8.1, 1.3 Hz, H14), 4.41-4.32 (1H, m, H7), 2.44 (2H, t, *J* = 7.3 Hz, H3), 2.42-2.36 (1H, m, H10), 2.33 (1H, ddd, *J* = 10.6, 6.5, 4.4 Hz, H12), 2.29-2.21 (1H, m, H10), 2.17 (2H, s, SiC$\underline{H}_2$Ph), 2.14 (1H, q, *J* = 7.0 Hz, H13), 1.74-1.68 (2H, m, H6), 1.67 (3H, dd, *J* = 6.4, 1.3 Hz, H16), 1.66-1.64 (1H, m, H11), 1.62 (2H, *app* t, *J* = 7.6 Hz, H4), 1.56-1.44 (3H, m, H11 and H5), 1.05 (3H, d, *J* = 7.0 Hz, H17), 0.11 (6H, s, Si(CH$_3$)$_2$)); **^{13}C NMR (126 MHz, CDCl$_3$)** δ_C 139.4, 134.9,

134.6, 128.5, 128.2, 125.1, 124.4, 116.7, 109.7, 85.8, 85.4, 81.5, 62.7, 41.4, 40.8, 38.3, 37.9, 31.9, 27.7, 26.7, 24.1, 18.1, 18.1, 17.1, –1.6; **HRMS (ESI$^+$)** calc. for $C_{28}H_{39}BrOSiNa$ $[M+Na]^+$ 521.1846, found 521.1835.

Benzyl((3*R*)-13-bromo-8-((*tert*-butyldimethylsilyl)oxy)-3-((*R*,*E*)-pent-3-en-2-yl)tetradeca-13-en-1,6-diyn-1-yl)dimethylsilane, 51

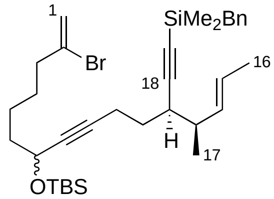

To a stirred solution of alcohol **S6** (1.30 g, 2.60 mmol, 1.0 equiv.) in dry dichloromethane (33 mL) under argon at 0 °C was added sequentially imidazole (266 mg, 3.90 mmol, 1.5 equiv.), 4-dimethylaminopyridine (31.8 mg, 0.260 mmol, 0.1 equiv.) and TBSCl (510 mg, 3.38 mmol, 1.5 equiv.). The reaction mixture was warmed to room temperature and stirred for 2 h before being quenched with $NaHCO_3$ solution (30 mL, sat. *aq.*). The layers were separated and the aqueous layer extracted three times with dichloromethane (3 x 30 mL). The combined organic layers were dried with Na_2SO_4 and the solvent carefully removed *in vacuo*. The crude product was purified by flash column chromatography on silica gel (19:1 Petroleum Ether / Et_2O) to yield TBS ether **51** (1.58 g, 2.57 mmol, 99%) as a colourless oil.

R_f 0.76 (Petroleum Ether / Et_2O (19:1)); **ν_{max} (thin film) /cm^{-1}** 2955 (s), 2859 (s), 2172 (m), 1494 (m), 1452 (m), 1250 (m), 1142 (m), 837 (s); **^{1}H NMR (500 MHz, $CDCl_3$)** δ_H 7.24-7.19 (2H, m, ArH), 7.11-7.25 (3H, m, ArH), 5.55 (1H, d, *J* = 1.5 Hz, H1), 5.43 (1H, dq, *J* = 15.1, 6.3 Hz, H15), 5.38 (1H, d, *J* = 1.5 Hz, H1), 5.31 (1H, dqd, *J* = 15.1, 8.0, 1.3 Hz, H14), 4.34 (1H, t, *J* = 6.3 Hz, H7), 2.43 (2H, *app* t, *J* = 7.3 Hz, H3), 2.40-2.32 (2H, m, H5 and H12), 2.29-2.20 (1H, m, H5), 2.17 (2H, s, SiC$\underline{H}_2$Ph), 2.16-2.07 (1H, m, H13), 1.69-1.61 (3H, m, H10 and H6), 1.66 (3H, dd, *J* = 6.3, 1.3 Hz, H16), 1.58 (2H, *app* dt, *J* = 15.3, 7.8 Hz, H4), 1.53-1.39 (3H, m, H6 and H11), 1.05 (3H, d, *J* = 6.9 Hz, H17), 0.91 (9H, s, $SiC(CH_3)_3$), 0.13 (3H, s, $Si(CH_3)_2$), 0.11 (9H, s, $Si(CH_3)_2$); **^{13}C NMR (126 MHz, $CDCl_3$)** δ_C 139.4, 135.0, 134.8, 128.5, 128.2, 125.1, 124.4, 116.6, 109.8, 85.6, 84.2, 82.2, 63.2, 41.5, 40.8, 38.8, 38.2, 32.0, 27.7, 26.7, 26.0, 24.3, 18.5, 18.1, 18.1, 17.1, –1.6, –1.6, –4.3, –4.8; **HRMS (EI$^+$)** calc. for $C_{30}H_{44}BrOSi_2$ $[M-{}^tBu]^+$ 555.2114, found 555.2233.

Benzyl((3*R*)-10-((*tert*-butyldimethylsilyl)oxy)-3-((*R*,*E*)-pent-3-en-2-yl)-1,2,3,6,7,8,9,10-octahydrocyclohepta[*e*]inden-4-yl)dimethylsilane, 53

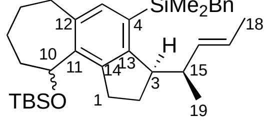

Bromoenediyne **51** (30 mg, 0.049 mmol, 1.0 equiv.) was dissolved in dry acetonitrile (1.95 mL), and the solution degassed with argon bubbling for 30 min. Triethylamine was separately degassed with argon bubbling for 30 min. A vial equipped with a stirrer bar was charged with tetrakis(triphenylphosphine)palladium(0) (2.8 mg, 0.002 mmol, 5 mol%) in the glovebox, and subsequently

degassed with argon bubbling for 15 min. The degassed solution of starting material was added to the catalyst by syringe, followed by the degassed triethylamine (54 µL, 0.39 mmol, 8.0 equiv.). The reaction mixture was stirred at 80 °C overnight, then cooled to room temperature and the solvent carefully removed *in vacuo*. The crude product was purified by flash column chromatography on silica gel (99:1 Petroleum Ether / Et_2O) to yield tricycle **53** (17.9 mg, 0.036 mmol, 69%, inseparable 1:1 mixture of diastereomers) as a yellow oil.

R_f 0.86 (Petroleum Ether / Et_2O (19:1)); **ν_{max} (thin film) /cm^{-1}** 2928 (s), 2855 (m), 1493 (m), 1451 (m), 1250 (m), 1154 (m), 1088(m), 1056 (m), 833 (s); **HRMS (EI^+)** calc. for $C_{34}H_{52}OSi_2$ $[M]^+$ 532.3557, found 532.3568.

Characterisation for diastereomer 1: ^{1}H NMR (500 MHz, $CDCl_3$) δ_H 7.15 (2H, t, *J* = 7.3 Hz, $SiCH_2\underline{Ph}$), 7.04 (1H, t, *J* = 7.3 Hz, $SiCH_2\underline{Ph}$), 6.91 (1H, s, H5), 6.91-6.89 (2H, m, $SiCH_2\underline{Ph}$), 5.26 (1H, dqd, *J* = 15.4, 6.3, 1.3 Hz, H17), 5.13 (1H, ddq, *J* = 15.4, 5.7, 1.3 Hz, H16), 5.06-5.00 (1H, m, H10), 3.40-3.33 (1H, m, H6), 3.24-3.19 (1H, m, H3), 2.81-2.74 (2H, m, H1), 2.49-2.39 (2H, m, H12 and H15), 2.32 (2H, s, $SiC\underline{H_2}Ph$), 2.31-2.19 (1H, m, H8), 2.05-1.96 (1H, m, H9), 1.96-1.89 (3H, m, H2 and H7), 1.73-1.64 (1H, m, H8), 1.55-1.52 (1H, m, H9), 1.52 (3H, d, *J* = 6.3 Hz, H18), 1.35-1.26 (1H, m, H2), 1.10 (3H, d, *J* = 6.6 Hz, H19), 0.92 (9H, s, $SiC(CH_3)_3$), 0.30 (3H, s, $Si(CH_3)_2$), 0.24 (3H, s, $Si(CH_3)_2$), 0.07 (3H, s, $Si(CH_3)_2$), –0.18 (3H, s, $Si(CH_3)_2$); **^{13}C NMR** (126 MHz, $CDCl_3$) δ_C 150.6, 141.1, 140.8, 140.5, 140.1, 136.1, 131.9, 131.6, 128.6, 128.1, 124.2, 124.1, 71.5, 50.7, 41.8, 35.3, 34.4, 30.9, 29.4, 27.5, 26.1, 25.1, 24.9, 19.0, 18.5, 18.3, –1.3, –1.7, –4.4, –4.9.

Characterisation for diastereomer 2: ^{1}H NMR (500 MHz, $CDCl_3$) δ_H 7.15 (2H, t, *J* = 7.3 Hz, $SiCH_2\underline{Ph}$), 7.04 (1H, t, *J* = 7.4 Hz, $SiCH_2\underline{Ph}$), 6.92 (1H, s, H5), 6.90-6.88 (2H, m, $SiCH_2\underline{Ph}$), 5.07-5.01 (2H, m, H16 and H10), 4.94 (1H, dqd, *J* = 15.5, 6.3, 0.6 Hz, H17), 3.40-3.33 (1H, m, H6), 3.03 (1H, d, *J* = 15.5 Hz, H3), 2.83-2.79 (1H, m, H1), 2.61 (1H, dt, *J* = 15.8, 9.3 Hz, H1), 2.50-2.46 (1H, m, H6), 2.31-2.28 (1H, m, H15), 2.30 (2H, s, $SiC\underline{H_2}Ph$), 2.27-2.20 (2H, m, H8), 2.04-1.99 (1H, m, H9), 1.95-1.84 (3H, m, H2 and H7), 1.71-1.67 (1H, m, H8), 1.45 (3H, d, *J* = 6.0 Hz, H18), 1.44-1.40 (1H, m, H9), 1.35-1.26 (1H, m, H2), 1.11 (3H, d, *J* = 6.9 Hz, H19), 0.90 (9H, s, $SiC(CH_3)_3$), 0.31 (3H, s, $Si(CH_3)_2$), 0.26 (3H, s, $OSi(CH_3)_2$), 0.08 (3H, s, $OSi(CH_3)_2$), –0.15 (3H, s, $Si(CH_3)_2$); **^{13}C NMR (126 MHz, $CDCl_3$)** δ_C 151.0, 140.5, 140.4, 140.2, 140.2, 135.5, 132.0, 130.9, 128.5, 128.1, 124.1, 124.0, 71.5, 51.2, 42.6, 35.4, 35.0, 31.2, 29.4, 27.6, 26.1, 25.3, 25.2, 19.0, 18.4, 18.3, –1.2, –1.5, –4.6, –4.7.

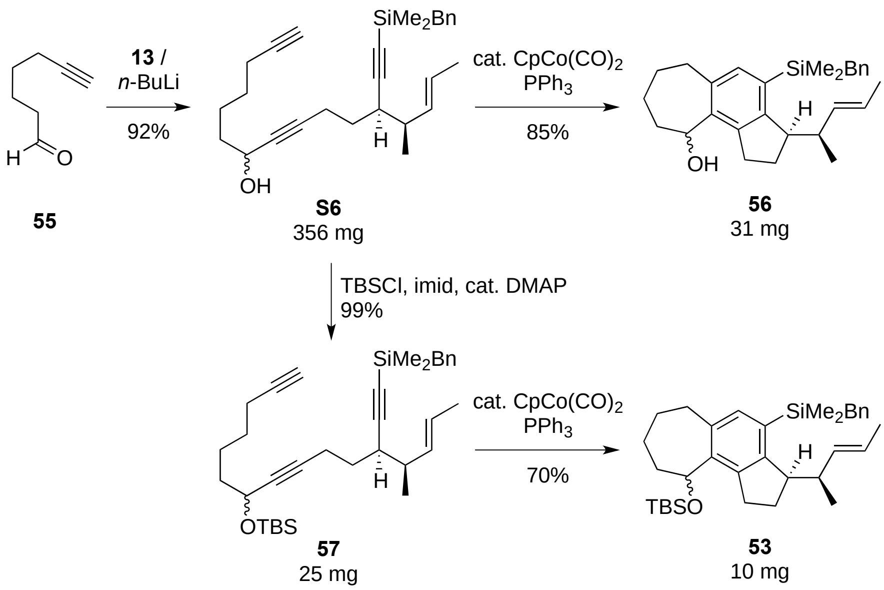

Scheme S3: Co-catalyzed cyclization to CDE rings **53** and **56**. Indicated masses correspond to the largest scale each compound was isolated from a single experiment.

(12*R*,13*R*,*E*)-12-((Benzyldimethylsilyl)ethynyl)-13-methylhexadeca-14-en-1,8-diyn-7-ol, 54

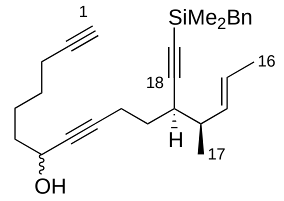

To a stirred solution of diyne **13** (343 mg, 1.11 mmol, 1.0 equiv) in dry THF (6.9 mL) under argon at –78 °C was added *n*-BuLi (2.5 M in hexane, 0.44 mL, 0.440 mmol, 0.99 equiv) dropwise. The reaction mixture was stirred for 30 min before a solution of hept-6-ynal (147 mg, 1.33 mmol, 1.2 equiv) in dry THF (2.3 mL) was added. The reaction mixture was warmed over 3 h from –78 °C to –20 °C before being quenched with NH_4Cl (0.6 mL, sat. *aq.*) and warmed to room temperature. The mixture was filtered through a plug of Celite® and the solvent removed *in vacuo*. The crude product was purified by flash column chromatography on silica gel (9:1 Petroleum Ether / Et_2O) to yield the triyne **54** (356 mg, 0.85 mmol, 77%, 97% brsm) as a colourless oil.

R_f 0.47 (Petroleum Ether / Et_2O (9:1)); **ν_{max} (thin film) /cm^{-1}** 3380 (br), 3307 (m), 2933 (m), 2166 (w), 1601 (m), 1494 (m), 1250 (m), 969 (m), 837 (s), 763 (m), 699 (m), 633 (m); **^{1}H NMR (500 MHz, $CDCl_3$)** δ_H 7.22 (2H, t, *J* = 7.7 Hz, ArH), 7.11-7.06 (3H, m, ArH), 5.44 (1H, dq, *J* = 15.1, 6.2 Hz, H15), 5.31 (1H, ddd, *J* = 15.1, 8.1, 1.3 Hz, H14), 4.37 (1H, *app* q, *J* = 6.4 Hz, H7), 2.40 (1H, dddd, *J* = 16.5, 8.5, 4.5, 1.9, H10), 2.33 (1H, ddd, *J* = 10.5, 6.5, 4.5 Hz, H12), 2.29-2.24 (1H, m, H10), 2.24-2.19 (2H, m, H3), 2.17 (2H, s, SiC$\underline{H}_2$Ph), 2.13 (1H, dqd, *J* = 8.1, 6.8, 6.5 Hz, H13), 1.95 (1H, t, *J* = 2.6 Hz, H1), 1.73 (1H, *app* dd, *J* = 5.4, 1.2 Hz, H6), 1.72- 1.68 (2H, m, H11), 1.67 (3H, dd, *J* = 6.2, 1.3 Hz, H16), 1.60- 1.56 (4H, m, H4 and H5), 1.55-1.49 (1H, m, H11), 1.05 (3H, d, *J* = 6.8 Hz, H17), 0.11 (6H, s, $Si(CH_3)_2$); **^{13}C NMR (126 MHz, $CDCl_3$)** δ_C 139.4, 134.9 , 128.5, 128.2, 125.1, 124.4, 109.7, 85.6, 85.3, 84.5, 81.5, 68.5, 62.7, 40.7, 38.3, 37.7, 31.9, 28.3, 26.6, 24.5, 18.5, 18.1, 18.1, 17.1, −1.6; **HRMS (ESI$^+$)** calc. for $C_{28}H_{38}NaO_{Si}$ [M+Na]$^+$ 441.2584, found 441.2583.

(3*R*)-4-(Benzyldimethylsilyl)-3-((*R*,*E*)-pent-3-en-2-yl)-1,2,3,6,7,8,9,10-octahydrocyclohepta[*e*]inden-10-ol, 56a and 56b.

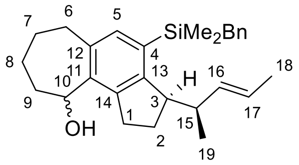

To a solution of triyne **54** (37.1 mg, 0.089 mmol, 1.0 equiv) in chlorobenzene (2.3 mL, 0.04 M) in a microwave tube was added triphenylphosphine (9.3 mg, 0.035 mmol, 40 mol%) and the mixture was degassed with argon bubbling for 30 min. Cyclopentadienylcobalt dicarbonyl (3.2 mg, 0.018 mmol, 20 mol%) was added and the reaction mixture was heated in a microwave (300 W) at 150 °C for 25 min. Upon cooling, the solvent was removed *in vacuo*. The crude product was purified by flash column chromatography on silica gel (9:1 Petroleum Ether / Et_2O) to yield two CDE ring system diastereomers **56a** (14.5 mg, 0.035 mmol, 39%) and **56b** (17.2 mg, 0.041 mmol, 46%) (85% combined yield) as colourless oils.

ν_{max} (thin film) /cm^{-1} 3308 (br), 2929 (s), 1493 (m), 1452 (m), 1248 (m), 1231 (m), 1148 m), 870 (m), 833 (m); **HRMS (ESI$^+$)** calc. for $C_{28}H_{38}NaOSi$ $[M+Na]^+$ 441.2584, found 441.2583.

Characterisation for diastereomer 56a: R$_f$ 0.27 (9:1 Petroleum Ether / EtOAc); **^{1}H NMR (500 MHz, CDCl$_3$)** δ_H 7.20 (2H, t, *J* = 7.4 Hz, ArH), 7.07 (1H, t, *J* = 7.4 Hz, ArH), 7.04 (1H, s, H5), 6.97 (2H, d, *J* = 7.4 Hz, ArH), 5.14 (1H, d, *J* = 5.3 Hz, H10), 5.13-5.09 (1H, m, H16), 5.09-5.02 (1H, dqd, *J* = 15.4, 6.1, 0.6 Hz, H17), 3.24 (1H, *app* t, *J* = 9.8 Hz, H1), 3.21 (1H, dt, *J* = 6.7, 3.1 Hz, H3), 2.84-2.72 (2H, m, H6), 2.64 (1H, dd, *J* = 14.1, 6.7 Hz, H1), 2.40-2.36 (1H, m, H15), 2.35 (2H, d, *J* = 4.7 Hz, SiC<u>H</u>$_2$Bn), 2.23-2.12 (2H, m, H9 and H8), 2.01-1.88 (3H, m, H2 and 2 × H7), 1.82-1.73 (1H, m, H9), 1.70 (1H, td, *J* = 14.4, 3.0 Hz, H8), 1.51 (3H, d, *J* = 6.1 Hz, H18), 1.48 (d, *J* = 3.1 Hz, OH), 1.47-1.37 (1H, *app* qdd, *J* = 12.3, 3.2, 1.6 Hz, H2), 1.11 (3H, d, *J* = 6.9 Hz, H19), 0.29 (3H, s, $Si(CH_3)_2$), 0.25 (3H, s, $Si(CH_3)_2$); **^{13}C NMR (126 MHz, CDCl$_3$)** δ_C 151.4, 142.4, 140.1, 140.1, 139.3, 136.2, 132.7, 131.8, 128.5, 128.2, 124.3, 124.2, 71.5, 51.1, 42.2, 35.9, 33.0, 31.0, 28.9, 27.3, 25.0, 24.9, 19.1, 18.4, −1.3, −1.6.

Characterisation for diastereomer 56b: R$_f$ 0.19 (9:1 Petroleum Ether / EtOAc); **^{1}H NMR (500 MHz, CDCl$_3$)** δ_H 7.19 (2H, t, *J* = 7.4 Hz, ArH), 7.07 (1H, t, *J* = 7.4 Hz, ArH), 6.99 (1H, s Hz, H5), 6.96 (2H, d, *J* = 7.4 Hz, ArH), 5.15-5.11 (1H, m, H10), 5.08 (1H, dqd, *J* = 15.3, 6.6, 1.6 Hz, H16), 4.95 (1H, dqd, *J* = 15.3, 6.0, 0.6 Hz, H17), 3.22 (1H, *app* t, *J* = 12.9 Hz, H1), 3.16 (1H, *app* dt, *J* = 7.3, 3.5 Hz, H3), 2.86 (1H, ddd, *J* = 15.8, 8.6, 2.2 Hz, H6), 2.72-2.64 (1H, m, H6), 2.61 (1H, dd, *J* = 14.3, 6.8 Hz, H1), 2.38-2.33 (1H, m, H15), 2.34 (2H, d, *J* = 3.0 Hz, SiC<u>H</u>$_2$Bn), 2.24- 2.15 (2H, m, H8 and H9), 2.01-1.89 (3H, m, 2 × H7 and H2), 1.76 (1H, *app* dt, *J* = 14.2, 4.7 Hz, H9), 1.61 (1H, d, *J* = 2.8 Hz, OH), 1.60-1.53 (1H, m, H8), 1.46 (3H, dt, *J* = 6.3, 1.3 Hz, H18), 1.45-1.36 (1H, m, H2), 1.11 (3H, d, *J* = 6.9 Hz, H19), 0.30 (3H, s, $Si(CH_3)_2$), 0.24 (3H, s, $Si(CH_3)_2$); **^{13}C NMR (126 MHz, CDCl$_3$)** δ_C 151.3, 142.0, 140.0, 139.8, 139.0, 135.9, 132.3, 131.6, 128.4, 128.0, 124.2, 124.0, 71.1, 51.0, 42.4, 35.6, 33.2, 31.0, 28.8, 27.2, 24.9, 24.8, 18.9, 18.1, −1.5, −1.8.

Benzyl((3*R*)-13-bromo-8-((*tert*-butyldimethylsilyl)oxy)-3-((*R*,*E*)-pent-3-en-2-yl)tetradeca-13-en-1,6-diyn-1-yl)dimethylsilane, 57

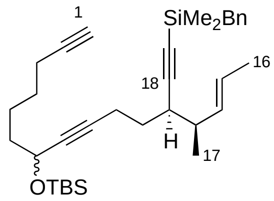

To a stirred solution of the triynol **54** (20.0 mg, 0.048 mmol, 1.0 equiv.) in dry dichloromethane (0.5 mL) under argon at 0 °C was added sequentially imidazole (4.9 mg, 0.072 mmol, 1.5 equiv.), 4-dimethylaminopyridine (0.6 mg, 0.005 mmol, 0.1 equiv.) and TBSCl (8.6 mg, 0.057 mmol, 1.2 equiv.). The reaction mixture was warmed to room temperature and stirred for 2 h before being quenched with $NaHCO_3$ solution (0.5 mL, sat. *aq.*). The layers were separated and the aqueous layer extracted three times with dichloromethane (3 x 0.5 mL). The combined organic layers were dried with Na_2SO_4 and the solvent carefully removed *in vacuo*. The crude product was purified by flash column chromatography on silica gel (19:1 Petroleum Ether / Et_2O) to yield TBS ether **57** (25.3 mg, 0.047 mmol, 99%) as a colourless oil.

$\mathbf{R_f}$ 0.74 (Petroleum Ether / Et_2O (9:1)); $\mathbf{\nu_{max}}$ **(thin film) /cm^{-1}** 2951 (s), 2858 (s), 2171 (w), 1494 (w), 1452 (w), 1250 (m), 1097 (m), 837 (s); **^{1}H NMR (400 MHz, $CDCl_3$)** δ_H 7.22 (2H, dd, *J* = 10.0, 5.3 Hz, ArH), 7.14- 7.03 (3H, m, ArH), 5.43 (1H, dq, *J* = 15.2, 6.2 Hz, H15), 5.31 (1 H, ddq, *J* = 15.2, 8.1, 1.2 Hz, H1), 4.34 (1H, dd, *J* = 8.3, 4.2 Hz, H7), 2.43-2.31 (2H, m, H6 and H12), 2.27 (1H, td, *J* = 8.1, 1.7 Hz, H6), 2.24-2.19 (2H, m, H10), 2.17 (2H, s, SiC$\underline{H}_2$Ph), 2.11 (1H, *app* sextet, *J* = 6.8 Hz, H13), 1.94 (1H, t, *J* = 2.6 Hz, H1), 1.71-1.60 (3H, m, H3 and H11), 1.66 (3H, dd, *J* = 6.2, 1.2 Hz, H16), 1.60-1.44 (5H, m, H4, H5 and H11), 1.05 (3H, d, *J* = 6.8 Hz, H17), 0.91 (9H, s, SiC(CH_3)$_3$), 0.13 (3H, s, Si(CH_3)$_2$), 0.11 (9H, s, Si(CH_3)$_2$); **^{13}C NMR (101 MHz, $CDCl_3$)** δ_C 139.4, 135.0, 128.5, 128.2, 125.1, 124.4, 109.8, 85.6, 84.6, 84.2, 82.2, 68.4, 63.2, 40.8, 38.6, 38.3, 38.2, 32.0, 28.4, 26.7, 26.0, 24.70, 18.5, 18.4, 18.1, 17.1, −1.6, −4.3, −4.8; **HRMS (EI$^+$)** calc. for $C_{34}H_{52}OSi_2$ [M]$^+$ 532.3557, found 532.3568.

Benzyl((3*R*)-10-((*tert*-butyldimethylsilyl)oxy)-3-((*R*,*E*)-pent-3-en-2-yl)-1,2,3,6,7,8,9,10-octahydrocyclohepta[*e*]inden-4-yl)dimethylsilane, 53

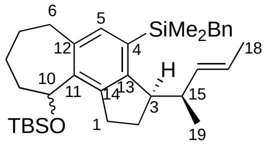

To a solution of triyne **57** (14.0 mg, 0.026 mmol, 1.0 equiv) in chlorobenzene (0.7 mL, 0.04 M) in a microwave tube was added triphenylphosphine (2.8 mg, 0.011 mmol, 40 mol%) and the mixture was degassed with argon bubbling for 30 min. Cyclopentadienyl cobalt dicarbonyl (1.0 mg, 0.005 mmol, 20 mol%) was added and the reaction mixture was heated in a microwave (300 W) at 150 °C for 25 min. Upon cooling, the solvent was removed *in vacuo*. The crude product was purified by flash column chromatography on silica gel (99:1 Petroleum Ether / Et_2O) to yield tricycle **53** (9.8 mg, 0.018 mmol, 70%,

inseparable 1:1 mixture of diastereomers) as a yellow oil. The data for this compound was identical to that recorded from the Pd-catalyzed route (*see above*).

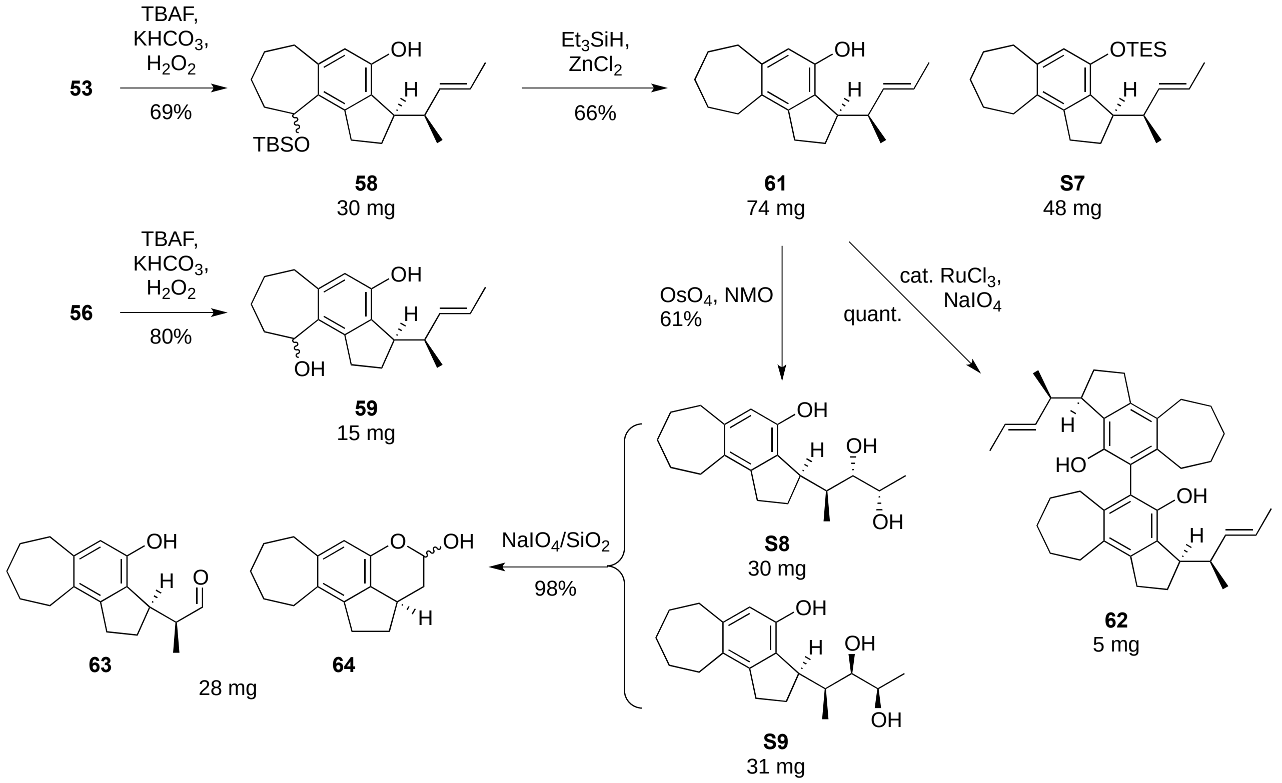

Scheme S4. Progression of the CDE rings **53** and **56** to CDEF pyran **64**. Indicated masses correspond to the largest scale each compound was isolated from a single experiment.

(3*R*)-10-((*tert*-Butyldimethylsilyl)oxy)-3-((*R*,*E*)-pent-3-en-2-yl)-1,2,3,6,7,8,9,10-octahydrocyclohepta[*e*]inden-4-ol, 58

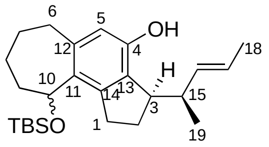

To a solution of benzyldimethylarylsilane **53** (56.5 mg, 0.106 mmol, 1.0 equiv.) in THF (0.95 mL) was added TBAF (1 M in THF, 223 µL, 0.223 mmol, 2.1 equiv.). The reaction mixture was stirred for 30 min, then hydrogen peroxide (30% w/v in water, 72 µL, 0.636 mmol, 6 equiv.) in methanol (0.95 mL) and potassium hydrogen carbonate (5.3 mg, 0.0530 mmol, 0.5 equiv.) were added. The reaction mixture was stirred at room temperature overnight, then quenched with $Na_2S_2O_3$ (1 mL, sat. *aq.*), the layers separated and the aqueous layer extracted three times with diethyl ether (3 x 1 mL). The combined organic layers were dried with Na_2SO_4 and the solvent carefully removed *in vacuo*. The crude product was purified by flash column chromatography on silica gel (19:1 Petroleum Ether / Et_2O) to yield phenol **58** (29.4 mg, 0.073 mmol, 69%, inseparable 1:1 mixture of diasteromers) as a colourless oil.

R_f 0.38 (Petroleum Ether / Et_2O (9:1)); **ν_{max} (thin film) /cm^{-1}** 3387 (br), 2920 (s), 2849 (m), 2174 (m), 1597 (m), 1439 (m), 1373 (m), 1297 (s), 1088 (s), 1053 (s), 1022 (s), 834 (s), 801 (s); **HRMS (EI$^+$)** calc. for $C_{25}H_{40}O_2Si$ [M]$^+$ 400.2798, found 400.2798.

Characterisation for diastereomer a: ^{1}H NMR (500 MHz, $CDCl_3$) δ_H 6.37 (1H, s, H5), 5.59-5.36 (2H, m, H16 and H17), 4.98 (1H, *J* = 6.3 Hz, H10), 4.82 (1H, s, OH), 3.38-3.24 (2H, m, H3 and H6), 2.90 (1H, *app* dt, *J* = 16.0, 8.2 Hz, H1), 2.78 (1H, tdd, *J* = 16.3, 9.5, 2.7 Hz, H1), 2.69-2.59 (1H, m, H15), 2.42 (1H, dd, *J* = 13.6, 6.6 Hz, H6), 2.28-2.18 (1H, m, H8), 2.16-2.05 (1H, m, H2), 2.03-1.87 (3H, m, H9, H2 and H7), 1.74-1.65 (1H, m, H8), 1.63 (2H, d, *J* = 5.7 Hz, H18), 1.52-1.46 (1H, m, H9), 1.34-1.27 (1H, m, H7), 0.98 (2H, d, *J* = 6.9 Hz, H19), 0.87 (9H, s, $SiC(CH_3)_3$), 0.03 (3H, s, $Si(CH_3)_2$), –0.23 (3H, s, $Si(CH_3)_2$); **^{13}C NMR (126 MHz, $CDCl_3$)** δ_C 150.9, 144.7, 144.3, 135.3, 132.3, 126.9, 125.7, 116.1, 71.1, 48.7, 39.8, 35.4, 34.9, 31.5, 29.3, 28.5, 26.0, 25.1, 18.3, 18.3, 16.7, –4.6, –5.0.

Characterisation for diastereomer 2: ^{1}H NMR (500 MHz, $CDCl_3$) δ_H 6.35 (1H, s, H5), 5.59-5.36 (2H, m, H16 and H17), 4.97 (1H, d, *J* = 6.6 Hz, H10), 4.79 (1H, s, OH), 3.35-3.26 (1H, m, H6), 3.26-3.20 (1H, m, H3), 2.81 (2H, *app* dd, *J* = 9.0, 5.6 Hz, H1), 2.59-2.51 (1H, m, H15), 2.42 (1H, dd, *J* = 13.6, 6.6 Hz, H6), 2.28-2.18 (1H, m, H8), 2.16-2.05 (1H, m, H2), 2.03-1.87 (3H, m, H9, H2 and H7), 1.74-1.65 (1H, m, H8), 1.64 (2H, d, *J* = 4.4 Hz, H18), 1.52-1.46 (1H, m, H9), 1.34-1.27 (1H, m, H7), 0.99 (2H, d, *J* = 6.9 Hz, H19), 0.87 (9H, s, $SiC(CH_3)_3$), 0.03 (3H, s, $Si(CH_3)_2$), –0.20 (3H, s, $Si(CH_3)_2$); **^{13}C NMR (126 MHz, $CDCl_3$)** δ_C 150.7, 144.2, 144.2, 135.7, 132.3, 126.9, 125.6, 115.9, 71.2, 48.6, 40.0, 35.4, 35.2, 31.4, 29.2, 29.1, 26.0, 25.3, 18.3, 18.2, 17.0, –4.7, –4.7.

(3*R*)-3-((*R*,*E*)-Pent-3-en-2-yl)-1,2,3,6,7,8,9,10-octahydrocyclohepta[*e*]indene-4,10-diol, 59

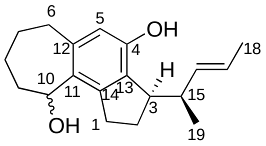

To a stirred solution of arylsilane **56** (27.7 mg, 0.066 mmol, 1.0 equiv.) in THF (0.7 mL) was added TBAF (0.26 mL, 1M in THF, 0.260 mmol, 4.0 equiv.), and the reaction mixture was stirred for 15 min. Upon disappearance of silane by TLC, MeOH (0.7 mL), $KHCO_3$ (13.2 mg, 0.132 mmol, 2.0 equiv.) and H_2O_2 (0.17 mL, 30 w/w in H_2O, mmol, 20 equiv.) were added sequentially. The reaction mixture was stirred at room temperature overnight before being quenched with $Na_2S_2O_3$ (0.5 mL, sat. *aq.*) and NH_4Cl (0.5 mL, sat. *aq.*). The layers were separated and the aqueous layer extracted three times with diethyl ether (3 x 1 mL). The combined organic layers were dried with $MgSO_4$ and the solvent removed carefully *in vacuo*. The crude product was purified by flash column chromatography on a short plug of silica (9:1 Petroleum Ether / EtOAc), then concentrated to yield phenol **59** (15.1 mg, 0.053 mmol, 80%, as a 46:54 mixture of diastereomers) as a colourless oil.

ν_{max} (thin film) /cm^{-1} 3308 (br), 2927 (s), 2854 (m), 1719 (m), 1648 (m), 1596 (m), 1449 (s), 1376 (m), 1254 (m), 1084 (s), 970 (s); **HRMS (ESI$^+$)** calc. for $C_{19}H_{25}NaO_2$ [M+Na]$^+$: 285.1860; found: 285.1861.

Characterisation for diastereomer a: R_f 0.55 (Petroleum Ether / EtOAc (4:1)); **^{1}H NMR (400 MHz, $CDCl_3$)** δ_H 6.34 (1H, s, H5), 5.47-5.42 (2H, dd, H16 and H17), 5.02 (1H, d, *J* = 6.0 Hz, H10), 4.90 (1H, s,

ArOH), 3.22 (1H, dt, *J* = 8.5, 4.2 Hz, H3), 3.12 (1H, *app* tt, *J* = 13.0, 2.0 Hz), 2.88 (1H, dt, *J* = 16.0, 8.1 Hz, H1), 2.71 (1H, ddd, *J* = 16.0, 9.4, 4.3 Hz, H1), 2.59-2.52 (1H, m, H15), 2.49 (1H, ddt, *J* = 14.2, 6.7, 1.6 Hz, H6), 2.11-2.01 (3H, m, H9, H8 and H2), 1.91-1.82 (2H, m, H2 and H7), 1.71-1.63 (1H, m, H8), 1.60 (3H, dd, *J* = 4.5, 1.3 Hz, H18), 1.55-1.49 (1H, m, H9), 1.38-1.31 (1H, m, H7), 0.92 (3H, d, *J* = 6.9 Hz, H19); **^{13}C NMR (101 MHz, $CDCl_3$)** δ_C 151.5, 146.1, 143.9, 135.8, 131.1, 127.4, 126.0, 116.5, 71.1, 49.0, 39.6, 35.8, 33.5, 31.2, 29.3, 28.9, 24.9, 18.3, 16.6.

Characterisation for diastereomer 2: R_f 0.35 (Petroleum Ether / EtOAc (4:1)); **^{1}H NMR (400 MHz, $CDCl_3$)** δ_H 6.40 (1H, s, H5), 5.55-5.39 (2H, m, H16 and H17), 5.08 (1H, d, *J* = 5.5 Hz, H10), 4.90 (1H, s, ArOH), 3.25 (1H, ddd, *J* = 8.6, 4.2, 3.2 Hz, H3), 3.16 (1H, t, *J* = 13.0 Hz, H6), 2.87 (2H, *app* dd, *J* = 9.1, 5.8 Hz), 2.62-2.51 (m, H6 and H15), 2.25-2.03 (3H, m, H9, H2 and H8), 2.03-1.83 (2H, m, H2 and H7), 1.79-1.70 (1H, m, H8), 1.65 (3H, dd, *J* = 4.5, 0.8 Hz, H18), 1.63-1.55 (1H, m, H9), 1.55-1.38 (2H, m, H7 and OH), 0.99 (3H, d, *J* = 6.9 Hz, H19); **^{13}C NMR (101 MHz, $CDCl_3$)** δ_C 151.4, 146.0, 143.7, 135.7, 131.1, 127.6, 125.9, 116.3, 71.1, 48.8, 40.1, 35.8, 33.7, 31.4, 29.2, 28.7, 25.0, 18.2, 16.9.

(*R*)-3-((*R*,*E*)-Pent-3-en-2-yl)-1,2,3,6,7,8,9,10-octahydrocyclohepta[e]inden-4-ol, 61 and triethyl(((*R*)-3-((*R*,*E*)-pent-3-en-2-yl)-1,2,3,6,7,8,9,10-octahydrocyclohepta[*e*]inden-4-yl)oxy)silane, S7

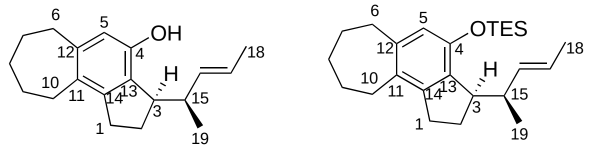

61 **S7**

To a stirred solution of TBS ether **58** (167 mg, 0.417 mmol, 1.0 equiv.) in dry dichloromethane (18.5 mL) was added zinc(II) chloride (85 mg, 0.625 mmol, 1.50 equiv.) and triethylsilane (0.13 mL, 0.834 mmol, 2.0 equiv.). The reaction mixture was stirred at room temperature for 3 h before being quenched with NH_4Cl (18 mL, sat. *aq.*). The layers were separated and the aqueous layer extracted three times with dichloromethane (3 x 18 mL). The combined organic layers were dried with $MgSO_4$ and the solvent removed *in vacuo*. The crude product was purified by flash column chromatography on a short plug of silica (Petroleum Ether / EtOAc (49:1)) to yield phenol **61** (74 mg, 0.274 mmol, 66%) as a colourless oil, and the triethylsilylaryl ether **S7** (48 mg, 189 mmol, 30%) as a colourless oil.

Characterisation for phenol 61: $[\alpha]_D^{25}$ +18.0 (*c* = 0.968, $CHCl_3$); **R_f** 0.36 (Petroleum Ether / EtOAc (9:1)); **ν_{max} (thin film) /cm^{-1}** 3427 (br), 2921 (s), 2849 (m), 1600 (m), 1447 (m), 1298 (m), 1261 (m), 1077 (m); **^{1}H NMR (500 MHz, $CDCl_3$)** δ_H 6.41 (1H, s, H5), 5.51-5.46 (2H, m, H16 and H17), 4.80 (1H, s, OH), 3.35-3.24 (1H, m, H3), 2.82 (1H, dt, *J* = 16.6, 8.4 Hz, H1), 2.77-2.72 (1H, m, H1), 2.72-2.68 (2H, m, H6), 2.67-2.61 (2H, m, H10), 2.60-2.50 (1H, m, H15), 2.19-2.04 (1H, m, H2), 1.93 (1H, ddt, *J* = 11.9, 8.3, 3.4 Hz, H2), 1.89-1.80 (1H, m, H8), 1.80-1.73 (1H, m, H8), 1.71-1.68 (1H, m, H7), 1.66 (3H, dd, *J* = 4.7, 1.3 Hz, H18), 1.64-1.59 (1H, m, H9), 1.54-1.51 (1H, m, H7), 1.51-1.45 (1H, m, H9), 0.98 (3H, d, *J* = 6.9 Hz, H19); **^{13}C NMR (126 MHz, $CDCl_3$)** δ_C 150.0, 145.1, 144.0, 136.0, 131.4, 127.2, 125.7, 114.8, 49.2, 40.1 36.4, 33.0, 31.4, 31.1, 29.3, 28.7, 28.1, 18.2, 16.6; **HRMS (EI$^+$)** calc. for $C_{19}H_{26}O$ [M]$^+$ 270.1984, found 270.1987.

Characterisation for silane S7: $[\alpha]_D^{25}$ +7.5 (*c* = 0.1648, $CHCl_3$); **R_f** 0.85 (Petroleum Ether / EtOAc (9:1));

ν_{max} (thin film) /cm^{-1} 2955 (s), 2920 (s), 1589 (m), 1481 (m), 1456 (m), 1297 (m), 1111 (m), 1086 (m), 1009 (m), 807 (m); **^{1}H NMR (500 MHz, CDCl$_3$)** δ_H 6.36 (1H, s, H5), 5.26-5.13 (2H, m, H16 and H17), 3.22-3.13 (1H, m, H3), 2.72- 2.61 (7H, m, H1, H15, H6 and H10), 1.98-1.85 (2H, m, H2), 1.79 (2H, *app* td, *J* = 12.1, 6.8, H8), 1.57-1.53 (2H, m, H7 and H9), 1.54 (3H, dd, *J* = 5.0, 1.0 Hz, H18), 1.04 (3H, d, *J* = 6.9 Hz, H19), 1.00 (9H, t, *J* = 7.9 Hz, Si(CH$_2$C$\underline{H}_3$)$_3$), 0.75 (6H, dd, *J* = 7.9, 1.5 Hz, Si(C$\underline{H}_2$CH$_3$)$_3$; **^{13}C NMR (126 MHz, CDCl$_3$)** δ_C 149.7, 144.6, 143.1, 134.2, 133.2, 131.6, 123.7, 117.5, 49.3, 38.6, 36.6, 33.0, 31.5, 31.2, 28.8, 28.1, 26.4, 19.1, 18.3, 7.0, 5.6; **HRMS (FI$^+$)** calc. for $C_{25}H_{40}OSi$ [M]$^+$ 384.2849, found 384.2867.

(2*R*,3*R*,4*S*)-4-((*R*)-4-hydroxy-1,2,3,6,7,8,9,10-octahydrocyclohepta[*e*]inden-3-yl)pentane-2,3-diol, S8 and (2*S*,3*S*,4*S*)-4-((*R*)-4-hydroxy-1,2,3,6,7,8,9,10-octahydrocyclohepta[*e*]inden-3-yl)pentane-2,3-diol, S9

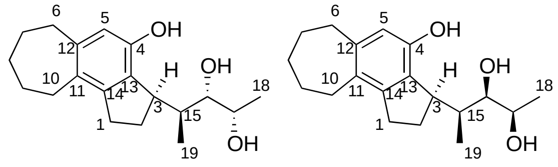

S8 **S9**

To a stirred solution of alkene **61** (49.0 mg, 0.181 mmol, 1.0 equiv.) in acetone (1.3 mL) and water (0.45 mL) was sequentially added osmium(VII) tetroxide (4% wt in water, 46 µL, 0.007 mmol, 0.04 equiv.) and NMO (50% wt in water, 88 µL, 0.362 mmol, 2.0 equiv.). The reaction mixture was stirred at room temperature for 2 h before being quenched with $Na_2S_2O_3$ (1.2 mL, sat. *aq*.). The layers were separated and the aqueous layer extracted three times with ethyl acetate (3 x 1.5 mL). The combined organic layers were dried with $MgSO_4$ and the solvent removed carefully *in vacuo*. The crude product was purified by flash column chromatography on a short plug of silica (4:1 Petroleum Ether / EtOAc → 3:1 Petroleum Ether / EtOAc), then concentrated to yield triols **S8** (16.8 mg, 30%) and **S9** (16.9 mg, 31%) as colourless oil. The stereochemistry of these diols is arbitrarily assigned, and of no consequence for the subsequent chemistry.

ν_{max} (thin film) /cm^{-1} 3442 (br), 2920 (s), 2848 (m), 1587 (m), 1449 (m), 1360 (m), 1279 (m), 1050 (m); **HRMS (ESI$^+$)** calc. for $C_{19}H_{28}NaO_3$ [M+Na]$^+$: 327.1931; found: 327.1928.

Characterisation for diastereomer S8: R$_f$ 0.21 (Petroleum Ether / EtOAc (7:3)); **^{1}H NMR (400 MHz, CDCl$_3$)** δ_H 8.20 (1H, br s, ArOH), 6.48 (1H, s, H5), 3.98 (1H, qd, *J* = 6.4, 3.2 Hz, H17), 3.69 (1H, ddd, *J* = 9.2, 2.8, 1.7 Hz, H3), 3.16 (1H, dd, *J* = 8.6, 3.2 Hz, H16), 2.92 (1H, ddd, *J* = 16.2, 10.3, 8.7 Hz, H1), 2.77 (1H, ddd, *J* = 16.2, 9.8, 2.0 Hz, H1), 2.73-2.58 (4H, m, H6 and H10), 2.37 (1H, *app* dq, *J* = 13.0, 9.7 Hz, H2), 2.06 (1H, dqd, *J* = 8.6, 6.9. 2.8 Hz, H15), 1.89-1.81 (2H, m, H2 and H8), 1.81-1.65 (2H, m, H8 and OH), 1.65-1.46 (4H, m, H7 and H9), 1.29 (3H, d, *J* = 6.4 Hz, H18), 0.76 (3H, d, *J* = 6.9 Hz, H19); **^{13}C NMR (101 MHz, CDCl$_3$)** δ_C 151.6, 145.4, 144.5, 130.4, 124.6, 115.2, 78.6, 67.6, 42.3, 41.6, 36.4, 33.0, 32.9, 32.1, 31.2, 28.7, 28.3, 21.0, 12.6.

Characterisation for diastereomer S9: R$_f$ 0.15 (Petroleum Ether / EtOAc (7:3)); **^{1}H NMR (400 MHz, CDCl$_3$)** δ_H 6.47 (1H, s, H5), 4.58 (2H, br s, OH), 3.74 (1H, dq, *J* = 8.3, 6.2 Hz, H17), 3.58 (1H, dd, *J* = 8.3, 2.2 Hz, H16), 3.36 (1H, ddd, *J* = 8.9, 3.2, 2.2 Hz, H3), 2.87 (1H, dt, *J* = 16.5, 8.4, H1), 2.80-2.56 (5H, m, H1, H6 and H10), 2.36 (1H, dq, *J* = 12.7, 8.9 Hz, H2), 1.96 (1H, qt, *J* = 7.2, 2.2 Hz, H15), 1.85 (2H, m, H2 and H8), 1.80-1.60 (3H, m, H8, H7 and H9), 1.57 (2H, m, H7 and H9), 1.22 (3H, d, *J* = 6.2 Hz, H18), 0.72 (3H,

d, J = 7.3 Hz, H19); **^{13}C NMR (101 MHz, CDCl$_3$)** δ_C 151.2, 145.0, 144.2, 130.7, 126.7, 115.8, 81.3, 69.6, 47.3, 40.5, 36.4, 35.8, 33.0, 31.3, 31.2, 28.7, 28.3, 19.9, 9.7.

(3*R*,3'*R*)-3,3'-di((*R*,*E*)-pent-3-en-2-yl)-1,1',2,2',3,3',6,6',7,7',8,8',9,9',10,10'-hexadecahydro-[5,5'-bi(cyclohepta[*e*]indene)]-4,4'-diol, 62

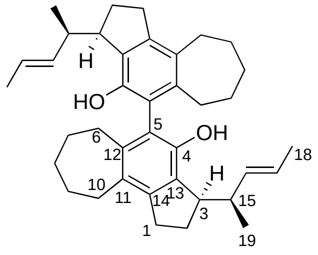

To a stirred solution of alkene **61** (5.0 mg, 0.0185 mmol, 1.0 equiv.) in 1,2-dichloroethane (0.1 mL) and water (80 µL) was sequentially added a solution of ruthenium(III) chloride in water (12.9 µL, 0.05 M in H_2O, 0.6 µmol, 3.5 mol%) and sodium periodate (7.9 mg, 0.0370 mmol, 2.0 equiv.). The reaction mixture was stirred at room temperature overnight before being quenched with $Na_2S_2O_3$ (1 mL, sat. *aq.*) and diluted with ethyl acetate (1 mL). The layers were separated and the aqueous layer was extracted three times with ethyl acetate (3 x 1 mL). The combined organic layers were dried ($MgSO_4$), and the solvent removed *in vacuo*. The crude product was purified by flash column chromatography on a short plug of silica (99:1 Petroleum Ether / Et_2O), then concentrated to yield diol **62** (5.0 mg, 0.0185 mmol, quant.) as a colourless oil. **$[\alpha]_D^{25}$** +16.9 (c = 0.455, $CHCl_3$); **R_f** 0.85 (Petroleum Ether / Et_2O (19:1)); **ν_{max} (thin film) /cm^{-1}** 3512 (2), 2954 (s), 2920 (s), 2849 (s), 1616 (m), 1437 (m), 1310 (m), 1075 (m), 966 (m); **^{1}H NMR (500 MHz, CDCl$_3$)** δ_H 5.29-5.19 (4H, m, 2 x H16 and 2 x H17), 4.47 (2H, s, 2 x OH), 3.32 (2H, dt, J = 8.7, 4.2 Hz, 2 x H3), 2.90-2.78 (6H, m, 4 x H1 and 2 x H15), 2.78-2.63 (4H, m, 4 x H10), 2.50-2.32 (4H, m, 4 x H6), 2.06 (2H, dq, J = 13.0, 8.7 Hz, 2 x H2), 2.00-1.93 (2H, m, 2 x H2), 1.82-1.69 (4H, m, 4 x H8), 1.65-1.55 (4H, m, 4 x H9), 1.53 (6H, d, J = 4.4 Hz, H18), 1.48-1.39 (4H, m, 4 x H7), 1.05 (6H, d, J = 6.9 Hz, H19); **^{13}C NMR (126 MHz, CDCl$_3$)** δ_C 148.1, 145.1, 142.6, 134.1, 131.7, 128.9, 124.3, 117.8, 49.6, 38.6, 32.8, 31.7, 31.6, 31.4, 28.3, 28.0, 26.2, 19.2, 18.3; **HRMS (ESI$^+$)** calc. for $C_{38}H_{51}O_2$ $[M+H]^+$ 539.3884, found 539.3886.

Equilibrium mixture of aldehyde (S)-2-((R)-4-hydroxy-1,2,3,6,7,8,9,10-octahydrocyclohepta[e]inden-3-yl)propanal, 63 and lactols (2a*R*,3*S*)-3-Methyl-1,2,2a,3,4,7,8,9,10,11-decahydrocyclohepta[*g*]cyclopenta[*de*]chromen-4-ol, 64

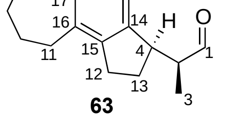

63

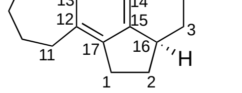

64

To a stirred solution of diols **S8** and **S9** (33.7 mg, 0.111 mmol, 1.0 equiv.) in dichloromethane (1.7 mL) at 0 °C was added 10 wt% sodium periodate on silica (308 mg, 0.144 mmol, 1.3 equiv.).[166] The suspension was stirred for a further 15 min at 0 °C before being loaded onto a short plug of silica and purified by flash

column chromatography (9:1 Petroleum Ether / Et_2O), then concentrated to yield lactol **181** (27.9 mg, 0.108 mmol, 98%, as a 63:13:24 inseparable mixture of epimers and open-chain aldehyde form) as a colourless oil. $\mathbf{R_f}$ 0.15 (Petroleum Ether / Et_2O (9:1)); $\mathbf{\nu_{max}}$ **(thin film) /cm^{-1}** 2918 (s), 2849 (m), 1739 (s), 1610 (m), 1453 (m), 1366 (m), 1261 (m), 1092 (m), 1017 (m), 966 (s), 798 (m); **HRMS (ESI$^+$)** calc. for $C_{17}H_{22}NaO_2$ $[M+Na]^+$ 281.1512, found 281.1516.

Characterisation for major epimer of lactol 64: ^{1}H NMR (500 MHz, $CDCl_3$) δ_H 6.40 (1H, s, H6), 5.38 (1H, dd, J = 2.6, 2.1 Hz, H4), 3.51-3.35 (1H, m, H16), 2.85-2.80 (3H, m, H1 and H7), 2.74-2.68 (3H, m, H7 and H11), 2.33-2.29 (1H, m, H3), 2.18-2.12 (1H, m, H2), 1.87-1.82 (1H, m, H9), 1.80-1.73 (2H, m, H2 and H9), 1.72-1.65 (2H, m, H8 and H10), 1.50 (2H, *app* ddd, J = 12.3, 8.9, 5.1 Hz, H8 and H10), 0.73 (3H, d, J = 7.2 Hz, H18); **^{13}C NMR (126 MHz, $CDCl_3$)** δ_C 146.5, 144.4, 143.2, 131.9, 124.1, 113.0, 97.4, 36.9, 35.3, 33.0, 32.3, 31.3, 31.2, 29.9, 28.7, 28.2, 11.0.

Characterisation for aldehyde 63: ^{1}H NMR (500 MHz, $CDCl_3$) δ_H 9.66 (1H, s, H1), 6.42 (1H, s, H6), 5.81 (1H, s, OH), 3.77 (1 H, dt, J = 9.1, 3.5 Hz, H4), 2.93-2.92 (1H, m, H2), 2.91-2.85 (1H, m, H12), 2.85–2.80 (2H, m, H12 and H7), 2.70–2.59 (3H, m, H7 and H11), 2.14-2.19 (2H, m, H13), 1.92-1.81 (1H, m, H10), 1.81-1.73 (2H, m, H13 and H10), 1.73-1.65 (2H, m, H8 and H9), 1.56-1.45 (2H, m, H8 and H9), 1.06 (3H, d, J = 7.6 Hz); **^{13}C NMR (126 MHz, $CDCl_3$)** δ_C 207.4, 150.0, 144.9, 144.8, 131.4, 125.1, 115.1, 51.6, 42.3, 36.3, 32.0, 31.4, 31.1, 30.8, 28.6, 28.1, 10.5.

1.2.3 Synthesis of the DEFG ring model system

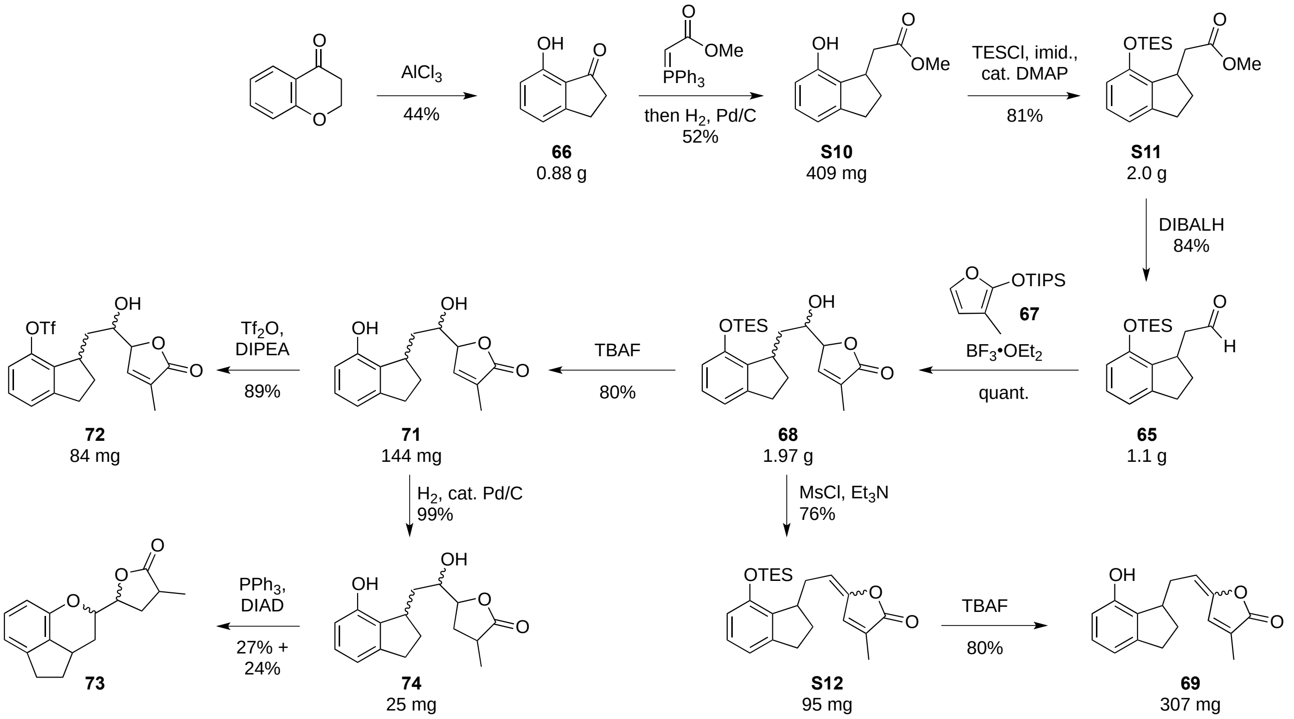

Scheme S5: The following reactions correspond to manuscript Schemes 6 and 7.

7-Hydroxyindan-1-one, 66

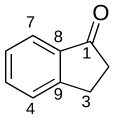

A mixture of 4-chromanone (2.00 g, 13.5 mmol, 1.0 equiv.) and anhydrous, powdered aluminum(III) chloride (5.20 g, 39 mmol, 2.9 equiv.) was heated with a heat gun for 10 min; the mixture turned black. The mixture was allowed to cool to room temperature, then CH_2Cl_2 (40 mL) and ice cold HCl (1 M, 10 mL) were added to the mixture. The resulting black slurry was diluted with water (40 mL) and extracted three times with dichloromethane (40 mL). The combined organic layers were dried ($MgSO_4$) and the solvent removed *in vacuo*. The crude product was purified by flash column chromatography on a short plug of silica (4:1 Petroleum Ether / EtOAc) to yield hydroxyindanone **66** (0.88 g, 5.94 mmol, 44%) as a yellow solid.
^{1}H NMR (200 MHz, $CDCl_3$) δ_H 9.07 (1H, s, OH), 7.47 (1H, *app* t, *J* = 7.8 Hz, H5), 6.95 (1H, dd, *J* = 7.3, 0.5 Hz, H4), 6.76 (1H, dd, *J* = 8.3, 0.5 Hz, H6), 3.12 (2H, t, *J* = 6.0 Hz, H3), 2.72 (2H, t, *J* = 6.0 Hz, H2); **^{13}C NMR (101 MHz, $CDCl_3$)** δ_C 210.2, 157.4, 155.4, 137.7, 117.6, 113.6, 36.1, 26.0. Data in accordance with the literature.[3]

Methyl 2-(7-hydroxy-2,3-dihydro-*1H*-inden-1-yl)acetate, S10

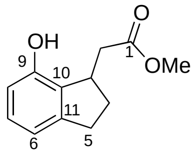

7-Hydroxy-2,3-dihydro-1*H*-inden-1-one **66** (565 mg, 3.81 mmol, 1.0 equiv.) and methyl 2-(triphenyl-λ^5-phosphanylidene)acetate (1.90 g, 5.72 mmol, 1.5 equiv.) in toluene (6.8 mL) was heated to reflux overnight, then the mixture was cooled to room temperature, and concentrated *in vacuo*. The residue was passed through a short pad of silica (4:1 Petroleum Ether / EtOAc) to yield a crude mixture of α,β-unsaturated ester and 4,5-dihydro-*2H*-cyclopenta[*de*]chromen-2-one. To a stirred solution of this mixture in methanol (7 mL) was added palladium on carbon (10 wt%, 406 mg, 10 mol%). Hydrogen gas was bubbled through the stirred reaction mixture for 1 h, then the resulting suspension was filtered through a pad of Celite and the solvent was removed *in vacuo*. The crude product was purified by flash column chromatography on silica (9:1 Petroleum Ether / EtOAc) to yield ester **S10** (409 mg, 1.98 mmol 52%) as a colourless oil.
R_f 0.30 (9:1 Petroleum Ether / EtOAc); **ν_{max} (thin film) /cm^{-1}** 3378 (br), 2951 (m), 1708 (s), 1591 (m), 1467 (s), 1439 (m), 1262 (m), 1168 (m), 991 (m); **^{1}H NMR (400 MHz, $CDCl_3$)** δ_H 7.07 (1H, t, *J* = 7.7 Hz), 6.78 (1H, d, *J* = 7.3 Hz), 6.69 (1H, d, *J* = 8.0 Hz), 3.73 (3H, s, $CO_2C\underline{H}_3$), 3.64 (1H, dddd, *J* = 10.0, 8.1, 3.8, 1.8 Hz, H3), 3.00 (1H, ddd, *J* = 15.7, 10.8, 7.3 Hz, H5), 2.79 (1H, *J* = 15.7, 8.4, 1.8 Hz, H5), 2.77 (1H, dd, *J* = 17.8, 10.0 Hz, H2), 2.67 (1H, dd, *J* = 17.8, 3.8 Hz, H2), 2.33 (1H, *app* ddt, *J* = 12.6, 10.8, 8.4 Hz, H4), 1.85 (1H, ddt, *J* = 12.6, 7.3, 1.8 Hz, H4); **^{13}C NMR (101 MHz, $CDCl_3$)** δ_C 177.0, 153.3, 146.0, 131.5, 128.9, 116.6, 114.8, 52.6, 39.5, 37.6, 34.4, 31.4; **HRMS (ESI$^+$)** calc. for $C_{12}H_{14}NaO_3$ $[M+Na]^+$ 229.0835, found 229.0835.

Methyl 2-(7-((triethylsilyl)oxy)-2,3-dihydro-*1H*-inden-1-yl)acetate, S11

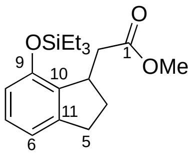

To a stirred solution of phenol **S10** (1.58 g, 7.66 mmol, 1.0 equiv.) in dichloromethane (31 mL) under argon at room temperature was added sequentially imidazole (782 mg, 11.5 mmol, 1.5 equiv.), DMAP (cat.) and chlorotriethylsilane (1.55 mL, 9.19 mmol, 1.2 equiv.). The reaction mixture was stirred for 2 h before being quenched with sat. *aq.* NH_4Cl solution. The layers were separated and the aqueous layer extracted three times with dichloromethane. The combined organic layers were dried with $MgSO_4$ and the solvent removed *in vacuo*. The crude product was purified by flash column chromatography on silica (19:1 Petroleum Ether / Et_2O) to yield silyl ether **S11** (2.00 g, 6.24 mmol, 81%) as a colourless oil.

R_f 0.51 (19:1 Petroleum Ether / Et_2O); **ν_{max} (thin film) /cm^{-1}** 2954 (m), 2877 (m), 1739 (s), 1588 (s), 1472 (s), 1269 (s), 1169 (m), 1007 (s), 775 (s); **^{1}H NMR (400 MHz, $CDCl_3$)** δ_H 7.04 (1H, t, *J* = 7.7 Hz, H7), 6.81 (1H, d, *J* = 7.4 Hz, H6), 6.60 (1H , d, *J* = 8.0 Hz, H8), 3.73-3.65 (1H, m, H3), 3.70 (3H, s, OC$\underline{H}_3$), 3.04 (1H, dd, *J* = 15.4, 3.2 Hz, H2), 2.97 (1 H, dd, *J* = 16.1, 8.2 Hz, H5), 2.83 (1 H, ddd, *J* = 16.1, 9.1, 4.0 Hz, H5), 2.33-2.22 (1H, m, H4), 2.20 (1H, dd, *J* = 15.4, 11.5 Hz, H2), 1.84 (1 H, ddd, *J* = 12.7, 8.2, 4.0 Hz, H4), 1.00 (9H, t, *J* = 7.6 Hz, Si($CH_2C\underline{H}_3$)$_3$), 0.78 (4H, d, *J* = 7.6 Hz, Si($C\underline{H}_2CH_3$)$_3$), 0.75 (2H, dd, *J* = 7.6, 1.7 Hz, Si($C\underline{H}_2CH_3$)$_3$); **^{13}C NMR (101 MHz, $CDCl_3$)** δ_C 173.8, 152.5, 146.1, 135.3, 128.3, 117.6, 116.2, 51.6, 39.9, 38.1, 31.5, 30.8, 6.8, 5.4; **HRMS (EI$^+$)** calc. for $C_{18}H_{28}O_3Si$ $[M]^+$ 320.1808, found 320.1801.

2-(7-((Trimethylsilyl)oxy)-2,3-dihydro-*1H*-inden-1-yl)acetaldehyde, 65

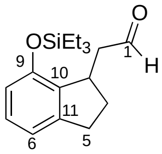

To a stirred solution of ester **S11** (1.44 g, 4.46 mmol, 1.0 equiv.) in dichloromethane (15 mL) under argon at –78 °C was added a solution of DIBALH (4.46 mL, 1.0 M in hexanes, 4.46 mmol, 1.0 equiv.) dropwise. The reaction mixture was stirred at –78 °C for 30 min before being quenched with Na K tartrate (sat. *aq.*). The layers were separated and the aqueous layer extracted three times with CH_2Cl_2. The combined organic layers were dried ($MgSO_4$) and the solvent removed *in vacuo*. The crude product was purified by flash column chromatography on silica (19:1 Petroleum Ether / Et_2O) to yield aldehyde **65** (1.10 g, 3.79 mmol, 84%) as a colourless oil.

R_f 0.51 (19:1 Petroleum Ether / Et_2O); **ν_{max} (thin film) /cm^{-1}** 2956 (m), 2877 (m), 1725 (s), 1588 (m), 1472 (s), 1271 (s), 1023 (s), 745 (s); **^{1}H NMR (400 MHz, $CDCl_3$)** δ_H 9.81 (1H, dd, *J* = 2.3, 1.4 Hz, H1), 7.05 (1H, t, *J* = 7.7 Hz, H7), 6.83 (1H, d, *J* = 7.4 Hz, H6), 6.61 (1H, d, *J* = 8.0 Hz, H8), 3.75 (1H, ddt, *J* = 9.6, 8.7, 3.9 Hz, H3), 3.00 (1H, ddd, *J* = 16.5, 3.9, 1.4 Hz, H2), 2.96 (1H, dd, *J* = 16.0, 8.3 Hz, H5), 2.86 (1H, ddd, *J* = 16.0, 8.9, 4.2 Hz, H5), 2.55 (1H, ddd, *J* = 16.5, 9.6, 2.3 Hz, H2), 2.33 (1H, dq, *J* = 12.9, 8.3 Hz, H4), 1.77 (1H, ddt, *J* = 12.9, 8.3, 4.2 Hz, H4), 0.92 (9H, t, *J* = 7.9 Hz, Si($CH_2C\underline{H}_3$)$_3$), 0.79 (4H, t, *J* = 7.9 Hz,

$Si(C\underline{H}_2CH_3)_3$), 0.76 (2H, dd, *J* = 7.8, 1.8 Hz, $Si(C\underline{H}_2CH_3)_3$); **^{13}C NMR (101 MHz, $CDCl_3$)** δ_C 202.8, 152.4, 146.1, 136.0, 128.4, 117.7, 116.2, 48.2, 37.6, 31.7, 31.3, 6.8, 5.4; **HRMS (EI$^+$)** calc. for $C_{17}H_{26}O_2Si$ $[M]^+$ 290.1702, found 290.1707.

5-(1-Hydroxy-2-(7-((triethylsilyl)oxy)-2,3-dihydro-*1H*-inden-1-yl)ethyl)-3-methylfuran-2(*5H*)-one, 68

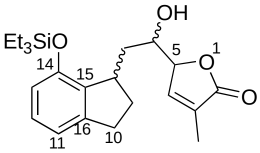

To a stirred solution of aldehyde **65** (1.47 g, 5.06 mmol, 1.0 equiv.) and triisopropyl((3-methylfuran-2-yl)oxy)silane **67** (1.54 g, 6.07 mmol, 1.2 equiv.) in diethyl ether (50 mL) under argon at –78 °C was added $BF_3{\cdot}OEt_2$ (0.25 mL, 2.02 mmol, 0.4 equiv.) dropwise. The reaction mixture was stirred warming to 0 °C over 3 h before being quenched with sat. *aq.* $NaHCO_3$ solution. The layers were separated and the aqueous layer extracted three times with diethyl ether. The combined organic layers were dried with $MgSO_4$ and the solvent removed *in vacuo*. The crude product was purified by flash column chromatography on silica 17:3 Petroleum Ether / EtOAc) to yield aldol adduct **68** (1.97 g, 5.06 mmol, quant.) as a colourless oil.

ν_{max} (thin film) /cm^{-1} 3473 (br), 2955 (w), 1761 (s), 1587 (m), 1471 (s), 1270 (s), 1059 (m), 1019 (m), 743 (m); **HRMS (ESI$^+$)** calc. for $C_{22}H_{32}NaO_4Si$ $[M+Na]^+$ 411.1962, found 411.1953.

Characterisation for major diastereomer: R_f 0.15 (Petroleum Ether / EtOAc (7:3)); **^{1}H NMR (500 MHz, $CDCl_3$)** δ_H 6.94 (1H, t, *J* = 7.7 Hz, H12), 6.92 (1H, quin, *J* = 1.6 Hz, H4), 6.77 (1H, d, *J* = 7.4 GHz, H11), 6.52 (1H, d, *J* = 8.0 Hz, H13), 4.72 (1H, dquin, *J* = 5.6, 1.9, H6), 3.67 (1H, td, *J* = 9.2, 4.9 Hz, H6), 3.33 (1H, td, *J* = 8.0, 1.3 Hz, H8), 3.11 (1 H, d, *J* = 4.7 Hz, OH), 2.91 (1H, ddd, *J* = 16.0, 10.4, 5.6 Hz, H10), 2.67 (1H, ddd, *J* = 16.0, 8.6, 1.6 Hz, H10), 2.13 (1H, ddt, *J* = 12.5, 10.4, 8.6 Hz, H9), 1.82 (3 H, t, *J* = 1.8 Hz, H17), 1.78 (1H, ddt, *J* = 12.4, 7.4, 1.7 Hz, H9), 1.61-1.55 (2H, m, H7), 0.88 (9H, t, *J* = 7.9 Hz, $SiCH_2C\underline{H}_3$), 0.69 (6 H, ddd, *J* 13.8, 7.9, 5.1, $SiC\underline{H}_2CH_3$); **^{13}C NMR (126 MHz, $CDCl_3$)** δ_C 174.2, 152.1, 146.7, 146.3, 136.1, 131.2, 128.2, 118.6, 116.1, 83.7, 70.8, 39.0, 36.6, 33.1, 31.4, 10.9, 6.7, 5.2.

Characterisation for minor diastereomer: R_f 0.20 (Petroleum Ether / EtOAc (7:3)); **^{1}H NMR (400 MHz, $CDCl_3$)** δ_H 6.92 (1H, t, *J* = 7.7 Hz, H12), 6.91-6.89 (1H, m, H4), 6.72 (1H, d, *J* = 7.3 Hz, H13), 6.50 (1H, d, *J* = 8.0 Hz, H11), 4.75 (1H, dquin, *J* = 5.3, 2.0 Hz, H5), 3.74 (1H, dtd, *J* = 10.3, 5.2, 2.7 Hz, H6), 3.47-3.30 (1H, m, H8), 2.87 (1H, dt, *J* = 16.0, 8.4 Hz, H10), 2.70 (1 H, ddd, *J* = 16.0, 8.9, 3.6 Hz, H10), 2.41-2.35 (1H, m, OH), 2.11 (1H, dq, *J* = 12.7, 8.7 Hz, H9), 1.95 (1H, ddd, *J* = 13.9, 10.3, 4.6 Hz, H7), 1.81 (3H, t, *J* = 1.7 Hz, H17), 1.74-1.68 (1H, m, H9), 1.43 (1 H, ddd, *J* = 13.8, 9.2, 2.6 Hz, H7), 0.89 (9H, t, *J* = 7.9 Hz, $SiCH_2C\underline{H}_3$), 0.72- 0.64 (6H, m, $SiC\underline{H}_2CH_3$); **^{13}C NMR (101 MHz, $CDCl_3$)** δ_C 174.0, 152.0, 146.1, 146.1, 136.5, 131.4, 128.0, 117.9, 116.4, 84.3, 71.2, 39.4, 36.6, 31.5, 31.1, 10.9, 6.8, 5.4.

(*E*)-3-Methyl-5-(2-(7-((triethylsilyl)oxy)-2,3-dihydro-*1H*-inden-1-yl)ethylidene)furan-2(*5H*)-one, S12

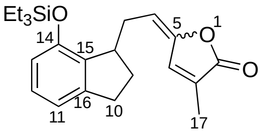

To a stirred solution of alcohol **68** (130 mg, 0.335 mmol, 1.0 equiv.) in CH_2Cl_2 (0.7 mL) under argon at 0 °C was added triethylamine (0.12 mL, 0.836 mmol, 2.5 equiv.), followed by methanesulfonyl chloride (28.5 µL, 0.368 mmol, 1.1 equiv.) dropwise. The reaction mixture was stirred warming to room temperature overnight before being quenched with sat. *aq.* NH_4Cl solution. The layers were separated and the aqueous layer extracted three times with CH_2Cl_2. The combined organic layers were dried with $MgSO_4$ and the solvent removed *in vacuo*. The crude product was purified by flash column chromatography on silica (19:1 Petroleum Ether / EtOAc) to yield alkene **338b** (94.5 mg, 0.255 mmol, 76%, as a 65:35 mixture of diastereomers) as a colourless oil.

R_f 0.79 (Petroleum Ether / EtOAc (4:1)); **ν_{max} (thin film) /cm^{-1}** 2955 (m), 1765 (s), 1587 (w), 1472 (m), 1366 (m), 1270 (m), 1217 (m), 1017 (m), 745 (m); **HRMS (ESI$^+$)** calc. for $C_{22}H_{30}NaO_3Si$ $[M+Na]^+$ 393.1856, found 393.1855.

Characterisation for major diastereomer: ^{1}H NMR (400 MHz, CDCl$_3$) δ_H 7.20 (1H, d, *J* = 0.7 Hz, H4), 7.03 (1H, t, *J* = 7.7 Hz, H12), 6.81 (1H, d, *J* = 7.4 Hz, H11), 6.60 (1H, d, *J* = 8.0 Hz, H13), 5.64 (1H, t, *J* = 8.6 Hz, H6), 3.44 (1H, *app* ddq, *J* = 11.8, 8.0, 3.9 Hz, H8), 3.00-2.88 (1H, m, H10), 2.83 (1H, dd, *J* = 9.1, 3.8 Hz, H10), 2.72 (1H, ddd, *J* = 14.0, 8.4, 4.5 Hz, H7), 2.43 (1H, dt, *J* = 14.0, 8.7 Hz, H7), 2.16 (1H, dq, *J* = 13.0, 8.7 Hz, H9), 1.99 (3H, s, H17), 1.79 (1H, dtd, *J* = 12.7, 8.3, 3.7 Hz, H9), 1.01 (9H, t, *J* = 7.7 Hz, $SiCH_2C\underline{H}_3$), 0.82-0.76 (6H, m, $SiC\underline{H}_2CH_3$); **^{13}C NMR (101 MHz, CDCl$_3$)** δ_C 171.4, 152.5, 149.5, 146.1, 135.3, 134.0, 130.2, 128.3, 117.7, 116.2, 112.5, 43.2, 31.7, 30.5, 30.0, 10.9, 6.9, 5.5.

Characterisation for minor diastereomer: ^{1}H NMR (400 MHz, CDCl$_3$) δ_H 7.03 (1H, t, *J* = 7.7 Hz, H12), 6.95 (1H, s, H4), 6.81 (1H, d, *J* = 7.4 Hz, H11), 6.60 (1H, d, *J* = 8.0 Hz, H13), 5.12 (1H, t, *J* = 8.0 Hz), 3.50-3.41 (1H, m, H8), 3.00-2.88 (1H, m, H10), 2.90-2.82 (1H, m, H7), 2.83 (1H, dd, *J* = 9.1, 3.8 Hz, H10), 2.65 (1H, dd, *J* = 14.8, 8.0 Hz, H7), 2.22-2.10 (1H, m, H9), 1.99 (3H, s, H17), 1.84-1.75 (1H, m, H9), 1.01 (9H, t, *J* = 7.7 Hz, $SiCH_2C\underline{H}_3$), 0.82-0.76 (6H, m, $SiC\underline{H}_2CH_3$); **^{13}C NMR (101 MHz, CDCl$_3$)** δ_C 171.2, 152.6, 149.2, 146.5, 137.9, 135.5, 129.1, 128.1, 117.6, 116.2, 113.6, 42.9, 31.8, 30.3, 30.0, 10.6, 6.9, 5.5.

(*E*)-5-(2-(7-hydroxy-2,3-dihydro-*1H*-inden-1-yl)ethylidene)-3-methylfuran-2(*5H*)-one, 69

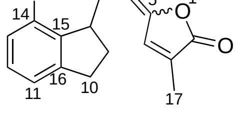

To a stirred solution of **S12** (555 mg, 1.50 mmol, 1.0 equiv.) in THF (21 mL) under argon was added TBAF (1.25 mL, 1.0 M in THF, 1.57 mmol, 1.05 equiv.). The reaction mixture was stirred overnight before being quenched with NH_4Cl (sat. *aq.*). The layers were separated and the aqueous layer extracted three times with dichloromethane. The combined organic layers were dried ($MgSO_4$) and the solvent removed *in vacuo*. The

crude product was purified by flash column chromatography on silica (4:1 Petroleum Ether / EtOAc) to yield alkene **339** (307 mg, 1.19 mmol, 80%, 87:13 mixture of diastereomers) as a colourless oil.

R_f 0.41 (Petroleum Ether / EtOAc (4:1)); **ν_{max} (thin film) /cm^{-1}** 3383 (br), 2944 (m), 1735 (s), 1591 (m), 1466 (m), 1278 (m), 1087 (m), 991 (m), 760 (m); **^{1}H NMR (400 MHz, $CDCl_3$)** δ_H 7.22 (1H, s, H4^{min}), 7.04 (1H, t, *J* = 7.7 Hz, H12), 6.97 (1H, d, *J* = 1.2 Hz, H4^{maj}), 6.79 (1H, d, *J* = 7.4 Hz, H11), 6.59 (1H, d, *J* = 7.9 Hz, H13), 5.70 (1H, t, *J* = 8.5 Hz, H6 ^{min}),5.28 (1H, br s, OH), 5.20 (1H, t, *J* = 8.0 Hz, H6^{maj}), 3.49 (1H, qd, *J* = 8.1, 3.7 Hz, H8), 2.95 (1H, dt, *J* = 16.1, 8.1 Hz, H10), 2.89-2.76 (2H, m, H7 and H10), 2.70 (1H, dt, *J* = 14.7, 8.1 Hz, H7^{maj}), 2.47 (1H, dt, *J* = 14.2, 8.5 Hz, H7^{min}),2.19 (1H, dq, *J* = 12.6, 8.4 Hz, H9), 1.97 (3H, s, H17), 1.83 (1H, ddd, *J* = 12.6, 7.9, 3.8 Hz, H9); **^{13}C NMR (101 MHz, $CDCl_3$)** δ_C 171.6, 152.5, 149.4, 146.7, 138.0, 131.5, 129.2, 128.4, 117.2, 113.5, 113.3, 42.4, 31.7, 30.9, 30.3, 10.6; **HRMS (ESI$^+$)** calc. for $C_{16}H_{16}NaO_3$ [M+Na]$^+$ 279.0992, found 279.0989

5-(1-Hydroxy-2-(7-hydroxy-2,3-dihydro-*1H*-inden-1-yl)ethyl)-3-methylfuran-2(*5H*)-one, 71

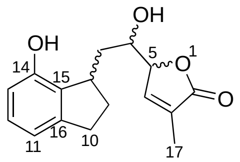

To a stirred solution of silyl ether **68** (256 mg, 0.659 mmol, 1.0 equiv.) in THF (11.5 mL) under argon was added TBAF (0.69 mL, 1.0 M in THF, 0.690 mmol, 1.05 equiv.). The reaction mixture was stirred overnight before being quenched with NH_4Cl (sat. *aq.*). The layers were separated and the aqueous layer extracted three times with CH_2Cl_2. The combined organic layers were dried ($MgSO_4$) and the solvent removed *in vacuo*. The crude product was purified by flash column chromatography on silica (3:1 Petroleum Ether / EtOAc) to yield phenol **71** (143.8 mg, 0.524 mmol, 80%, 55:45 mixture of diastereomers) as a white foam.

R_f 0.27 (Petroleum Ether / EtOAc (1:1)); **ν_{max} (thin film) /cm^{-1}** 3339 (br), 2919 (m), 2850 (m), 1737 (s), 1658 (m), 1466 (s) 1271 (m), 1061 (m); **^{1}H NMR (400 MHz, $CDCl_3$)** δ_H 7.11 (1H, m, H4^{maj}), 7.09 (1H, t, *J* = 7.7 Hz, H12), 7.04 (1H, *app* quin, *J* = 1.5 Hz, H4^{min}), 6.82 (1H, d, *J* = 7.4 Hz, H11), 6.67 (1H, d, *J* = 8.0 Hz, H13), 4.94-4.82 (1H, m, H5), 4.17 (1H, ddd, *J* = 10.7, 3.9, 2.5 Hz, H6^{maj}), 3.90 (1H, ddd, *J* = 10.6, 6.0, 2.9 Hz, H6^{min}), 3.48 (1H, *app* qd, *J* = 8.6, 4.8 Hz, H8), 3.01 (1H, *app* dtd, *J* = 12.1, 8.3, 3.9 Hz, H10), 2.91-2.78 (2H, m, H10), 2.35 (1H, *app* dquin, *J* = 12.7, 8.3 Hz, H9), 2.07 (1H, ddd, *J* = 13.4, 10.6, 4.8 Hz, H7^{min}), 2.03-1.97 (1H, m, H7^{maj}), 1.96 (3H, s, H17^{min}), 1.96 (3H, s, H17^{maj}), 1.93-1.85 (1H, m, H9), 1.85-1.78 (1H, m, H7^{maj}), 1.73 (1H, ddd, *J* = 14.4, 6.8, 2.8 Hz, H7^{min}), 1.64 (1H, br s, OH); **^{13}C NMR (101 MHz, $CDCl_3$)** δ_C 174.8, 174.7, 152.4, 146.0, 145.9, 145.8, 145.7, 132.1, 132.0, 132.0, 131.9, 128.6, 116.9, 114.2, 84.7, 84.3, 72.2, 71.3, 38.9, 38.5, 36.8, 36.8, 32.4, 32.3, 31.4, 10.9; **HRMS (ESI$^+$)** calc. for $C_{16}H_{18}NaO_4$ [M+Na]$^+$ 297.1097, found 297.1093.

3-(2-Hydroxy-2-(4-methyl-5-oxo-2,5-dihydrofuran-2-yl)ethyl)-2,3-dihydro-*1H*-inden-4-yl trifluoromethanesulfonate, 72

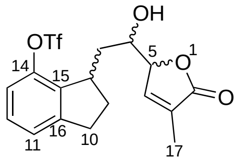

To a stirred solution of phenol **71** (64.3 mg, 0.235 mmol, 1.0 equiv.) in dichloromethane (2.2 mL) under argon at 0 °C was added sequentially DIPEA (86 µL, 0.493mmol, 2.1 equiv.) and trifluoromethanesulfonic anhydride (42 µL, 0.246 mmol, 1.05 equiv.). The reaction mixture was stirred for 1 h before being quenched with NH_4Cl (2 mL, sat. *aq.*). The layers were separated and the aqueous layer extracted three times with dichloromethane. (3 x 2 mL) The combined organic layers were dried with $MgSO_4$ and the solvent removed *in vacuo*. The crude product was purified by flash column chromatography on silica (3:1 Petroleum Ether / EtOAc) to yield triflate **72** (83.5 mg, 0.205 mmol, 89%, as a 1:1 mixture of diastereomers) as a yellow oil.

R_f 0.24 (Petroleum Ether / EtOAc (4:1)); **ν_{max} (thin film) /cm^{-1}** 3440 (br), 2921 (m), 1748 (s), 1463 (m), 1418 (s), 1211 (m), 1138 (m), 1061 (m), 963 (m), 853 (m); **HRMS (ESI$^+$)** calc. for $C_{17}H_{17}FNaO_6S$ $[M+Na]^+$ 429.0590, found 429.0583; **^{1}H NMR (400 MHz, $CDCl_3$) (diastereomer a = ^{a}; diastereomer b = ^{b})** δ_H 7.25-7.20 (2H, m, H7 and H5), 7.15 (1H, t, *J* = 1.6 Hz, H16^a), 7.07-7.01 (2.5H, m, H6 and H16^b), 4.88 (1H, dquin, *J* = 5.2, 1.9 Hz, H12^b), 4.76 (1 H, dquin, *J* = 5.2 Hz, H12^a), 3.90-3.81 (1H, m, H11), 3.81-3.71 (1H, m, H3), 3.07 (1H, dtd, *J* = 16.4, 8.1, 3.8 Hz, H1), 2.93 (1H, ddt, *J* = 16.4, 8.6, 4.1 Hz, H1), 2.41-2.28 (1H, m, H2), 2.28 (1H, m, OH^b), 2.22 (1H, d, *J* = 6.5 Hz, OH^a), 2.08-1.99 (1H, m, H10), 1.99-1.89 (1H, m, H2), 1.94 (3 H, t, *J* = 1.6 Hz, H17^a), 1.92 (3H, t, *J* = 1.7 Hz, H17^b), 1.77 (1H, dd, *J* = 13.9, 11.0, 1.9 Hz, H10^b), 1.62 (1H, ddd, *J* = 13.9, 10.8, 2.8 Hz, H10^a).

Characterisation for diastereomer a: ^{13}C NMR (101 MHz, $CDCl_3$) δ_C 174.0, 148.2, 146.4, 145.8, 138.8, 131.7, 129.1, 125.0, 119.0, 118.2 (q, $\underline{C}F_3$), 84.0, 70.6, 39.7, 36.8, 31.6, 30.4, 10.8.

Characterisation for diastereomer b: ^{13}C NMR (101 MHz, $CDCl_3$) δ_C 174.1, 148.2 , 146.4, 145.7, 138.8, 131.7, 129.1, 124.9, 119.0, 118.2 (q, $\underline{C}F_3$), 84.1, 70.4, 39.6, 36.3, 31.6, 30.6, 10.9

3-Methyl-5-(3,3a,4,5-tetrahydro-*2H*-cyclopenta[*de*]chromen-2-yl)dihydrofuran-2(*3H*)-one, 73

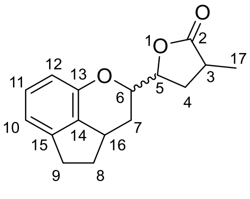

To a stirred solution of diol **74** (25.0 mg, 0.0905 mmol, 1.0 equiv.) in THF (0.9 mL) under argon at 0 °C was added sequentially triphenylphosphine (35.6 mg, 0.136 mmol, 1.5 equiv.) and DIAD (27.5 µL, 0.1346 mmol, 1.5 equiv.) dropwise. The reaction mixture was stirred warming to room temperature for 2 h before being filtered through a short pad of Celite®, and washed with diethyl ether. The solvent was removed *in vacuo* and the crude product-2-was purified by to yield **diastereomer a** (6.3 mg, 0.024 mmol, 27%) as a colourless oil and **diastereomer b** (5.7 mg, 0.022 mmol, 24%) as a colourless oil.

ν_{max} (thin film) /cm^{-1} 2922 (m), 1781 (s), 1597 (m), 1469 (s), 1246 (m), 1186 (m), 1169 (m), 1026 (m); **HRMS (FI$^+$)** calc. for $C_{16}H_{18}O_3$ $[M]^+$: 258.1256; found: 258.1251.

Characterisation for diastereomer a: R_f 0.69 (Et_2O/ CH_2Cl_2 (1:99)); **^{1}H NMR (400 MHz, $CDCl_3$)** δ_H7.07 (1H, t, *J* = 7.7 Hz, H11), 6.83 (1H, d, *J* = 7.3 Hz, H10), 6.61 (1H, d, *J* = 8.1 Hz, H12), 4.44 (1H, td, *J* = 9.3, 5.1 Hz, H5), 4.36 (1H, ddd, *J* = 9.1, 4.5, 1.0 Hz, H6), 3.08 (1H, *app* tt, *J* = 11.6, 5.9 Hz, H16), 3.01-2.87 (1H, m, H9), 2.77 (1H, dd, *J* = 15.4, 7.9 Hz, H9), 2.74 -2.62 (2H, m, H8 and H3), 2.59 (1H, dd, *J* = 13.6, 5.3, 1.0 Hz, H7), 2.44 (1 H, dt, *J* = 11.8, 6.5 Hz, H8), 1.94-1.80 (1H, m, H4), 1.72-1.55 (2H, m, H4 and H7), 1.32 (3H, d, *J* = 6.6 Hz, H17); **^{13}C NMR (101 MHz, $CDCl_3$)** δ_C 179.2, 151.5, 145.6, 129.0, 128.7, 116.6, 112.2, 78.1, 75.8, 35.9, 35.9, 35.6, 32.9, 32.5 , 29.3, 15.4.

Characterisation for diastereomer b: R_f 0.51 (Et_2O/ CH_2Cl_2 (1:99)); **^{1}H NMR (400 MHz, $CDCl_3$)** δ_H 7.06 (1H, t, *J* = 7.7 Hz, H11), 6.80 (1H, d, *J* = 7.3 Hz, H10), 6.67 (1H, d, *J* = 8.1 Hz, H12), 4.51 (1H, dt, *J* = 10.6, 6.2 Hz, H5), 4.39 (1H, td, *J* = 5.7, 1.2 Hz, H6), 3.04 (1H, *app* tt, *J* = 11.7, 5.9 Hz, H16), 2.99-2.87 (1H, m, H9), 2.76 (1H, dd, *J* = 15.4, 8.0 Hz, H9), 2.67 (1H, ddt, *J* = 12.0, 8.6, 7.0 Hz, H3), 2.50 (1H, ddd, *J* = 12.3, 8.6, 5.9 Hz, H4), 2.42 (1H, dt, *J* = 11.6, 6.5 Hz, H8), 2.28 (1H, ddd, *J* = 13.7, 5.3, 1.2 Hz, H7), 1.80 (1H, td, *J* = 12.0, 10.8 Hz, H4), 1.70 (1H, *app* td, *J* = 13.1, 5.6 Hz, H7), 1.63-1.47 (1H, m, H8), 1.32 (3H, d, *J* = 7.0 Hz, H17); **^{13}C NMR (101 MHz, $CDCl_3$)** δ_C 178.8, 151.6, 145.0, 128.9, 128.7, 116.4, 112.6, 79.7, 76.9, 36.2, 35.4, 33.5, 33.3, 32.4, 29.9, 15.2 .

5-(1-Hydroxy-2-(7-hydroxy-2,3-dihydro-*1H*-inden-1-yl)ethyl)-3-methyldihydrofuran-2(*3H*)-one, 74

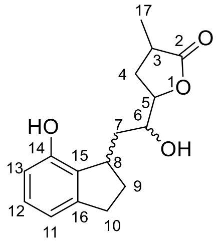

To a stirred solution of alkene **71** (25.0 mg, 0.0911 mmol, 1.0 equiv.) in methanol (0.5 mL) was added 10% Pd/C (9.7 mg, 0.0091 mmol, 10 mol%). The hydrogen gas was bubbled through the resulting suspension for 30 min, before the reaction mixture was filtered through a pad of Celite® to yield **74** as a colourless oil (25.0 mg, 0.904 mmol, 99%). The crude product was used in the following step without further purification.

R_f 0.31 (Petroleum Ether / EtOAc (1:1)); **ν_{max} (thin film) /cm^{-1}** 3375 (br), 1752 (s), 1590 (m), 1466 (s), 1201 (m); **HRMS (ESI$^+$)** calc. for $C_{22}H_{32}NaO_4Si$ $[M+Na]^+$: 411.1962; found: 411.1953. **^{1}H NMR (400 MHz, $CDCl_3$) (diastereomer a = ^{a}; diastereomer b = ^{b})** δ_H 7.06 (1H, t, *J* = 7.7 Hz, H12^a), 7.06 (1H, t, *J* = 7.7 Hz, H12^b), 6.77 (2H, d, *J* = 7.3 Hz, H11), 6.70 (1H, dd, *J* = 8.0 Hz, H13^a), 6.69 (1H, dd, *J* = 8.0 Hz, H13^b), 5.52 (4H, br s, OH), 4.32 (1H, ddd, *J* = 9.9, 6.2, 3.2 Hz, H5^a), 4.30 -4.21 (2H, m, H5^b and H6^a), 3.82 (1H, ddd, *J* = 11.0, 6.8, 2.2 Hz, H6^b), 3.57-3.43 (2H, m, H8), 2.97 (2H, *app* dt, *J* = 16.4, 8.3 Hz, H10), 2.84 (1H, dd, *J* = 8.7, 3.6 Hz, H10^a), 2.80 (1H, dd, *J* = 8.8, 3.5 Hz, H10^b), 2.75-2.66 (2H, m, H3), 2.40-2.21 (4H, m, H4^b, H9 and H4^a), 1.98-1.89 (3H, m, H4^a, H9 and H6^b), 1.88-1.76 (3H, m, H9 and H7^a), 1.69 – 1.59 (1H, m, H4^a), 1.59-1.51 (2H, m, H7), 1.27 (3H, d, *J* = 7.0 Hz, H17^a), 1.26 (3H, d, *J* = 3.6 Hz, H17^b);

Characterisation for diastereomer a: ^{13}C NMR (101 MHz, $CDCl_3$) δ_C 180.1, 152.7, 145.7, 132.2, 128.6, 116.6, 114.3, 80.8, 70.9, 39.0, 36.0, 35.6, 33.1, 31.5, 29.8, 15.1;

Characterisation for diastereomer b: ^{13}C NMR (101 MHz, $CDCl_3$) δ_C 179.7, 152.8, 145.7, 132.3, 128.6, 116.6, 114.3, 81.9, 73.9, 38.5, 36.2, 35.9, 33.2, 32.7, 31.4, 15.1.

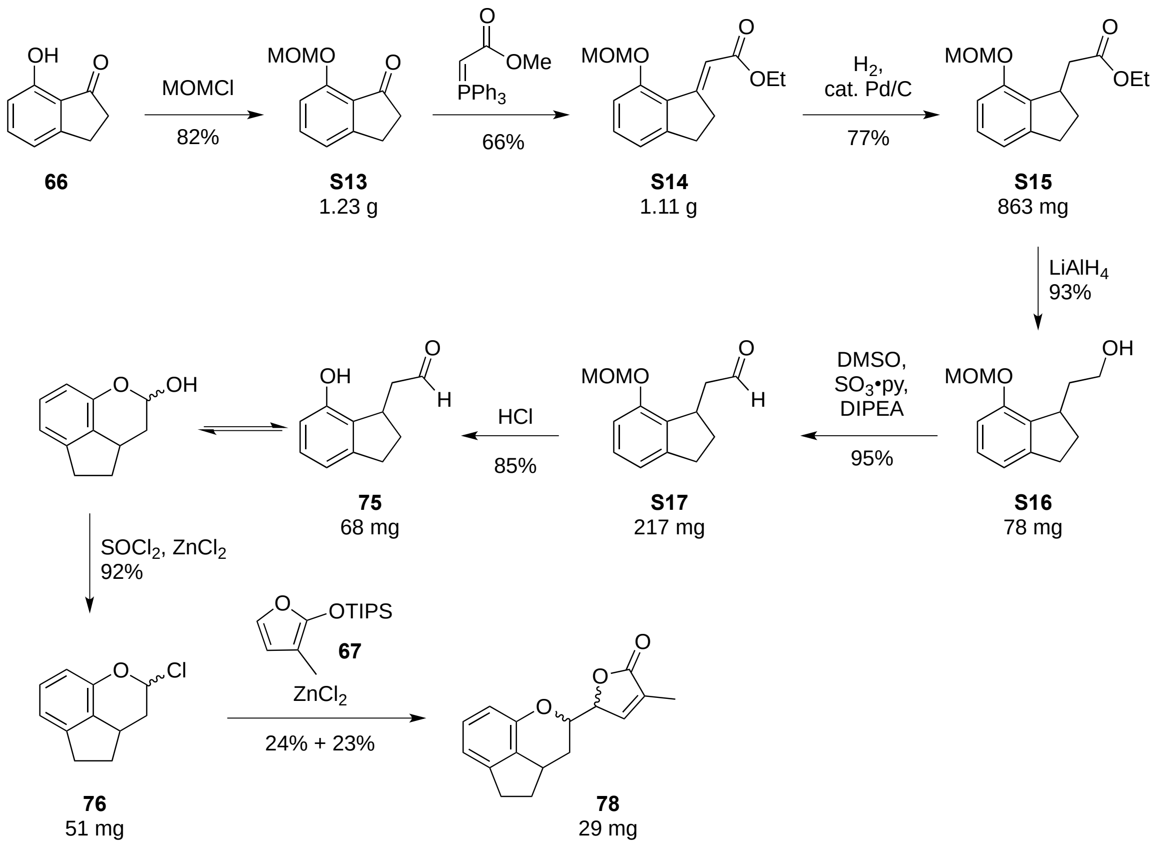

Scheme S6: Synthetic route to DEFG rings **78**.

7-(Methoxymethoxy)-2,3-dihydro-1*H*-inden-1-one, S13

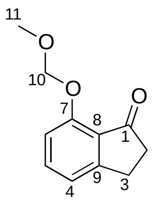

To a stirred solution of phenol **66** (1.16 g, 7.83 mmol, 1.0 equiv.) in anhydrous THF (14 mL) under argon at 0 °C was added sodium hydride (328 mg, 60% in mineral oil, 7.83 mmol, 1.0 equiv.). The resulting solution was stirred at 0 °C for 30 min before MOMCl (1.33 g, 15.7 mmol, 2.0 equiv.) was added dropwise. The ice bath was removed, and the reaction mixture stirred at RT for 2 h before being quenched with $NaHCO_3$ (14 mL, sat. *aq*.). The layers were separated and the aqueous layer extracted three times with ethyl acetate (3 x 14 mL). The combined organic layers were dried with $MgSO_4$ and the solvent removed carefully *in vacuo*. The crude product was purified by flash column chromatography on a short plug of silica (3:1 Petroleum Ether / Et_2O), to yield MOM ether **S13** (1.23 g, 6.40 mmol, 82%) as a yellow oil.

R_f 0.14 (Petroleum Ether / Et_2O (4:1)); **ν_{max} (thin film) /cm^{-1}** 2924 (m), 2362 (m), 1708 (s), 1599 (s), 1237 (m), 1153 (s), 1008 (s), 925 (m); **^{1}H NMR (400 MHz, $CDCl_3$)** δ_H 7.49 (1H, *app* t, *J* = 7.9 Hz, H5), 7.07 (1H, *app* dd, *J* = 7.6, 0.7 Hz, H4), 7.04 (1H, *app* dd, *J* = 8.2, 0.5 Hz, H6), 5.34 (2H, s, H10), 3.52 (3H, s, H11), 3.13-3.05 (2H, m, H3), 2.74-2.63 (2H, m, H2); **^{13}C NMR (101 MHz, $CDCl_3$)** δ_C 204.7, 157.8, 155.7, 136.3, 126.1, 119.9, 113.2, 94.7, 56.6, 37.0, 25.6; **HRMS (ESI$^+$)** calc. for $C_{11}H_{12}O_3$ $[M+Na]^+$ 215.0679, found 215.0681.

Ethyl (*E*)-2-(7-(methoxymethoxy)-2,3-dihydro-1*H*-inden-1-ylidene)acetate, S14

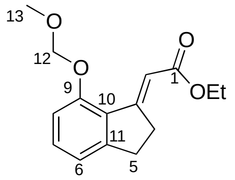

To a stirred suspension of sodium hydride (536 mg, 60% in mineral oil, 13.4 mmol, 2.1 equiv.) in anhydrous toluene (24 mL) under argon at 0 °C was added triethyl phosphonoacetate (2.63 mL, 13.4 mmol, 2.1 equiv.) dropwise. The resulting solution was stirred at room temperature, before being cooled to 0 °C. A solution of ketone **S13** (1.23 g, 6.40 mmol, 1.0 equiv.) in anhydrous toluene (37 mL) was added dropwise. The ice bath was removed, and the reaction mixture was heated to reflux overnight. The deep red solution was allowed to cool to room temperature before being quenched with NaCl (60 mL, sat. *aq.*). The layers were separated and the aqueous layer extracted three times with ethyl acetate (3 x 60 mL). The combined organic layers were dried with $MgSO_4$ and the solvent removed carefully *in vacuo*. The crude product was purified by flash column chromatography on a short plug of silica (19:1 Petroleum Ether / EtOAc), to yield α,β-unsaturated ester **S14** (1.11g, 4.47 mmol, 66%) as a yellow oil.
R_f 0.42 (Petroleum Ether / EtOAc (9:1)); **ν_{max} (thin film) /cm^{-1}** 2926 (m), 1703 (m), 1622 (m), 1598 (m), 1478 (m), 1258 (m), 1154 (s), 1037 (m); **^{1}H NMR (400 MHz, $CDCl_3$)** δ_H 7.25 (1H, t, *J* = 7.8 Hz, H7), 7.00-6.96 (2H, m, H8 and H2), 6.87 (1H, t, *J* = 2.4 Hz, H6), 5.32 (2H, s, H12), 4.22 (2H, q, *J* = 7.1 Hz, $OC\underline{H}_2CH_3$), 3.51 (3H, s, H13), 3.34-3.27 (2H, m, H5), 3.8-3.01 (2H, m, H4), 1.33 (3H, t, *J* = 7.1 Hz, $OCH_2C\underline{H}_3$); **^{13}C NMR (101 MHz, $CDCl_3$)** δ_C 168.3, 161.5 , 155.6, 152.0, 131.5, 128.4, 118.5, 111.9, 111.6, 93.9, 59.5, 56.2, 31.5, 30.7, 14.5; **HRMS (ESI$^+$)** calc. for $C_{15}H_{28}NaO_4$ $[M+Na]^+$ 285.1097, found 285.1093.

Ethyl 2-(7-(methoxymethoxy)-2,3-dihydro-1*H*-inden-1-yl)acetate, S15

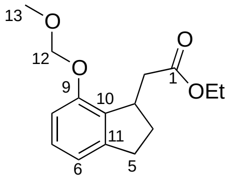

To a stirred solution of α,β-unsaturated ester **S14** (1.11 g, 4.23 mmol, 1.0 equiv.) in methanol (17 mL) was added 10% Pd/C (450 mg, 0.423 mmol, 10 mol%). The H_2 was bubbled through the resulting suspension for 30 min, before the reaction mixture was filtered through a pad of Celite® to yield ester **S15** as a colourless oil (863 mg, 3.26 mmol, 77%). The crude product was used in the following step without further purification.

R_f 0.38 (Petroleum Ether / Et_2O (9:1)); **ν_{max} (thin film) /cm^{-1}** 2921 (m), 2851 (m), 2359 (m), 2335 (m), 1734 (s), 1476 (m), 1254 (m), 1154 (m), 1032 (s); **^{1}H NMR (400 MHz, $CDCl_3$)** δ_H 7.12 (1H, t, *J* = 7.8 Hz, H7), 6.88 (2H, *app* dd, *J* = 7.7, 3.8 Hz, H8 and H6), 5.19 (2H, s, H12), 4.16 (2H, q, *J* = 7.1 Hz, $OC\underline{H}_2CH_3$), 3.80-3.72 (1H, m, H3), 3.47 (3H, s, H13), 3.04-2.93 (2H, m, H2 and H5), 2.80 - 2.89 (1H, m, H5), 2.28 - 2.38 (2H, m, H2 and H4), 1.86 (1H, ddt, *J* = 13.1, 8.7, 4.4 Hz, H4), 1.27 (3H, t, *J* = 7.1 Hz, $OCH_2C\underline{H}_3$); **^{13}C NMR (101 MHz, $CDCl_3$)** δ_C 173.1, 153.9, 146.9, 133.5, 128.3, 118.1, 111.4, 94.0, 60.2, 56.0, 39.6, 38.4, 31.3, 31.0, 14.3; **HRMS (ESI$^+$)** calc. for $C_{15}H_{20}NaO_4$ $[M+Na]^+$ 287.1254, found 287.1243.

2-(7-(Methoxymethoxy)-2,3-dihydro-1*H*-inden-1-yl)ethan-1-ol, S16

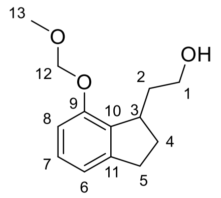

To a stirred solution of ester **S15** (100 mg, 0.378 mmol, 1.0 equiv.) in anhydrous diethyl ether (47 mL) under argon at 0 °C was added lithium aluminium hydride (4 M in Et_2O, 0.95 mL, 0.38 mmol, 1.0 equiv.) dropwise. The stirred reaction mixture was warmed to room temperature over 2 h, before being cooled to 0 °C and quenched dropwise with water (0.1 mL), then NaOH (0.2 mL, 10% *aq.*), and then water (0.3 mL). The resulting suspension was stirred at room temperature for 1 h, then filtered through a pad of Celite® and the solvent removed *in vacuo*. The crude product was purified by flash column chromatography on a short plug of silica (3:2 Petroleum Ether / EtOAc), to yield alcohol **S16** (78 mg, 93%) as a colourless oil.

R_f 0.15 (Petroleum Ether / Et_2O (9:1)); **ν_{max} (thin film) /cm^{-1}** 3358 (br), 2941 (m), 1588 (m), 1475 (m), 1251 (m), 1152 (m), 1033 (s), 934 (s); **^{1}H NMR (400 MHz, $CDCl_3$)** δ_H 7.14-7.09 (1H, m, H7), 6.90 (2H, *app* d, *J* = 7.8 Hz, H6 and H8), 5.22 (2H, s, H12), 3.74-3.62 (2H, m, H1), 3.50 (3H, s, H13), 3.49-3.40 (1H, m, H3), 3.07-2.96 (1H, m, H5), 2.81 (1H, ddd, *J* = 16.0, 8.9, 2.6 Hz, H5), 2.31-2.19 (1H, m, H4), 2.11 (1H, br s, OH), 1.95-1.75 (3H, m, H2 and H4); **^{13}C NMR (101 MHz, $CDCl_3$)** δ_C 154.0, 146.4, 134.9, 128.2, 118.6, 111.5, 94.6, 60.2, 56.4, 38.9, 37.0, 32.2, 31.5, 15.4; **HRMS (ESI$^+$)** calc. for $C_{13}H_{18}NaO_3$ $[M+Na]^+$ 245.1148, found 245.1145.

2-(7-(Methoxymethoxy)-2,3-dihydro-1*H*-inden-1-yl)acetaldehyde, S17

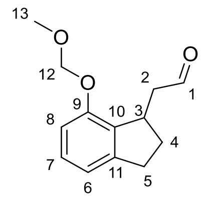

Dry DMSO (3.2 mL, 43.5 mmol, 42 equiv.) was added to the sulfur trioxide pyridine complex (1.45 g, 8.38 mmol, 8.1 equiv.) under argon, and the suspension was stirred at room temperature for 15 min. Dichloromethane (20 mL) was added, then the mixture was cooled to 0 °C and stirred for a further 10 min. A solution of alcohol **S16** (230 mg, 1.03 mmol, 1.0 equiv.) in dry dichloromethane (20 mL) and *N*,*N*-diisopropylethylamine (3.85 mL, 21.2 mmol, 20.5 equiv.) were added simultaneously, and the resulting

mixture stirred warming to room temperature for 2 h before being quenched with NH_4Cl (50 mL, sat. *aq*). The layers separated and the organic layer was washed with NaCl solution (40 mL, sat. *aq.*). The organic layer was dried with $MgSO_4$ and the solvent removed carefully *in vacuo*. The crude product was purified by flash column chromatography on a short plug of silica (9:1 Petroleum Ether / EtOAc), to yield aldehyde **S17** (217 mg, 0.985 mmol, 95%) as a white solid.

R_f 0.32 (Petroleum Ether / Et_2O (9:1)); **ν_{max} (thin film) /cm^{-1}** 2947 (m), 1722 (m), 1589 (m), 1475 (m), 1253 (m), 1152 (m), 1031 (s); **^1H NMR (400 MHz, $CDCl_3$)** δ_H 9.84 (1H, t, *J* = 2.2 Hz, H1), 7.19-7.06 (1 H, m, H7), 6.96-6.83 (2H, m, H6 and H8), 5.18 (2H, s, H12), 3.87-3.74 (1H, m, H3), 3.46 (3H, s, H13), 3.04-2.93 (2H, m, H2 and H5), 2.92-2.83 (1H, m, H5), 2.56 (1H, ddd, *J* = 16.6, 8.6, 2.2 Hz, H2), 2.43-2.31 (1H, m, H4), 1.79 (1H, ddt, *J* = 13.0, 8.4, 4.8 Hz, H4); **^{13}C NMR (101 MHz, $CDCl_3$)** δ_C 202.7, 153.7, 145.9, 133.1, 128.5, 118.1, 111.3, 93.9, 56.1, 48.1, 37.4, 31.6, 31.4; **HRMS (ESI$^+$)** calc. for $C_{13}H_{16}NaO_3$ $[M+Na]^+$ 243.0992, found 243.0993.

Equilibrium mixture of aldehyde 2-(7-hydroxy-2,3-dihydro-1*H*-inden-1-yl)acetaldehyde and lactols 3,3a,4,5-tetrahydro-2*H*-cyclopenta[*de*]chromen-2-ol, 75

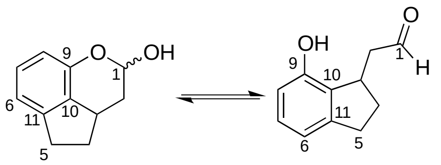

To a stirred solution of **S17** (100 mg, 0.454 mmol, 1.0 equiv.) in wet THF (1.5 mL) was added HCl (1.5 mL, 6 N *aq.*). The resulting mixture was stirred at room temperature for 2 h, before being diluted with ethyl acetate (2 mL) and NaCl solution (2 mL, sat. *aq.*). The layers were separated and the aqueous layer extracted three times with ethyl acetate (3 x 2 mL). The combined organic layers were dried with $MgSO_4$ and the solvent removed carefully *in vacuo*. The crude product was purified by flash column chromatography on a short plug of silica (9:1 Petroleum Ether / EtOAc), to yield lactol **75** (68 mg, 0.386 mmol, 85%, as a 53:37:10 inseparable mixture of open-chain aldehyde form and epimeric lactols) as a colourless oil.

R_f 0.23 (Petroleum Ether / EtOAc (9:1)); **ν_{max} (thin film) /cm^{-1}** 2926 (s), 1731 (m), 1455 (m), 1378 (m), 1237 (m), 1125 (m); **HRMS (ESI$^+$)** calc. for $C_{11}H_{12}NaO_2$ $[M+Na]^+$ 199.0730, found 199.0735.

Characterisation for aldehyde form: ^1H NMR (500 MHz, $CDCl_3$) δ_H 9.82 (1H, s, C1), 7.13-7.02 (2H, m, H7 and OH), 6.79 (1 H, d, *J* = 7.3 Hz, H6), 6.66 (1 H, d, *J* = 7.9 Hz, H8), 3.72-3.63 (1H, m, H3), 3.04 (1H, dd, *J* = 19.2, 9.5 Hz, H2), 3.01-2.92 (1H, m, H5), 2.87 (1H, dd, *J* = 19.2, 4.1 Hz, H2), 2.85-2.79 (1H, m, H5), 2.37-2.31 (1H, m, H4), 1.82 (1H, ddt, *J* = 12.9, 7.3, 1.9 Hz, H4); **^{13}C NMR (126 MHz, $CDCl_3$)** δ_C 204.9, 152.7, 145.8, 131.6, 128.8, 116.6, 114.3, 50.1, 34.9, 33.9, 31.2.

Characterisation for lactol form: ^1H NMR (500 MHz, $CDCl_3$) δ_H 7.13-7.02 (2H, m, H7), 6.85 (1H, d, *J* = 7.3 Hz, H6), 6.62 (1 H, d, *J* = 7.9 Hz, H8), 5.76 (1H, t, *J* = 2.0 Hz, H1), 3.28 (1H, tt, *J* = 11.7, 6.1 Hz, H3), 3.01-2.92 (1H, m, H5), 2.79 (2 H, dd, *J* =16.4, 7.9 Hz, H5), 2.49-2.40 (1H, m, H4), 2.40-2.31 (1H, m, H2), 1.64 (1 H, qd, *J* = 11.3, 7.9 Hz, H4), 1.45 (1H, td, *J* = 12.9, 2.5 Hz, H2); **^{13}C NMR (126 MHz, $CDCl_3$)** δ_C 150.0, 144.8, 129.6, 128.3, 116.7, 112.0, 93.0, 35.2, 33.1, 32.3, 30.9.

2-Chloro-3,3a,4,5-tetrahydro-*2H*-cyclopenta[*de*]chromene, 76

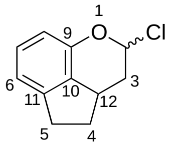

To an oven-dried vial charged with a stirrer bar and $ZnCl_2$ (15.3 mg, 0.113 mmol, 0.4 equiv.) under argon at 0 °C was added a cooled solution of lactol-aldehyde mixture **75** (50.0 mg, 0.284 mmol, 1.0 equiv.) in dry toluene (1.1 mL), followed by thionyl chloride (32 µL, 0.426 mmol, 1.5 equiv.) dropwise. The reaction mixture was warmed to room temperature and stirred for 6 h before being filtered through a short pad of oven-dried Celite®, and washed with dry dichloromethane. The solvent was removed *in vacuo* to yield chloropyran **76** (50.7 mg, 92%) as a pale green oil which was used in the next step without further purification. N.B.: this compound is unstable to water and silica.

ν_{max} (thin film) /cm^{-1} 2957 (m), 1623 (m), 1598 (m), 1469 (s), 1232 (s), 1203 (s), 1097 (s), 753 (s); **^{1}H NMR (400 MHz, $CDCl_3$)** δ_H 7.10 (1H, dd, *J* = 8.1, 7.3 Hz, H7), 6.92 (1H, d, *J* = 7.3 Hz, H6), 6.68 (1H, d, *J* = 8.1 Hz, H8), 6.53 (1H, t, *J* = 2.3 Hz, H2, ***indicates equatorial H***), 3.62-3.51 (1H, m, H12), 3.08-2.95 (1H, m, H5), 2.80 (1H, dd, *J* = 15.4, 7.9 Hz, H5), 2.57 (1H, ddd, *J* = 13.5, 5.3, 2.0 Hz, H3), 2.48 (1H, dt, *J* = 11.7, 6.4 Hz, H4), 1.89 (1H, ddd, *J* = 13.5, 12.1, 2.6 Hz, H3), 1.70 (1H, qd, *J* = 11.7, 7.9 Hz, H4); **^{13}C NMR (101 MHz, $CDCl_3$)** δ_C 148.7, 145.3, 129.0, 128.7, 118.3, 112.7, 90.1, 37.1, 35.3, 32.4, 31.9; **HRMS (FI$^+$)** calc. for $C_{11}H_{11}OCl$ $[M]^+$: 194.0498; found: 194.0495.

3-Methyl-5-(3,3a,4,5-tetrahydro-*2H*-cyclopenta[*de*]chromen-2-yl)furan-2(*5H*)-one, 78

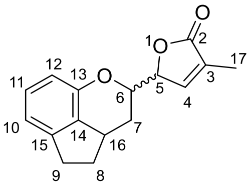

To an oven-dried vial charged with a stirrer bar and $ZnCl_2$ (14.1 mg, 0.099 mmol, 0.4 equiv.) under argon at –20 °C was added a cooled solution of chloropyran **76** (48.0 mg, 0.246 mmol, 1.0 equiv.) and siloxyfuran **67** (80.0 mg, 0.296 mmol, 1.2 equiv.) in dry dichloromethane (2.4 mL). The reaction mixture was stirred warming to room temperature overnight before being filtered through a short pad of oven-dried Celite®, and washed with dry dichloromethane. The solvent was removed *in vacuo* and the crude product was purified by flash column chromatography on silica (9:1 → 5:1 Petroleum Ether / EtOAc) to yield DEFG rings **78** as a 1:1 mixture of diastereomer **78a** (14.5 mg, 24%) and **78b** (14.1 mg, 23%), as white powders.

ν_{max} (thin film) /cm^{-1} 2925 (m), 1739 (s), 1596 (m), 1470 (m), 1366 (m), 1251 (m), 1217 (m), 771 (m); **HRMS (ESI$^+$)** calc. for $C_{16}H_{16}NaO_3$ $[M+Na]^+$: 279.0992; found: 279.0986.

Characterisation for diastereomer a: R_f 0.42 (Petroleum Ether / EtOAc (20:3)); **^{1}H NMR (400 MHz, $CDCl_3$)** δ_H 7.38 (1H, quin, *J* = 1.7 Hz, H4), 7.05 (1H, dd, *J* = 8.1, 7.3 Hz, H11), 6.81 (1H, d, *J* = 7.3 Hz, H10), 6.59 (1H, d, *J* = 8.1 Hz, H12), 4.92 (1H, dq, *J* = 7.2, 1.7 Hz, H5), 4.03 (1H, ddd, *J* = 11.4, 7.2, 2.2 Hz, H6), 3.10 (1H, *app* tt, *J* = 11.3, 5.9 Hz, H16), 3.03-2.92 (1H, m, H9), 2.77 (1H, dd, *J* = 15.4, 8.0 Hz, H9), 2.50 (1H, ddd, *J* = 12.8, 5.1, 2.2 Hz, H7), 2.42 (1H, dt, *J* = 12.0, 6.4 Hz, H8), 1.98 (3H, t, *J* = 1.7 Hz, H17),

1.63 (1H, tdd, J = 12.0, 10.8, 8.2 Hz, H8), 1.44 (1H, dt, J = 12.8, 11.4 Hz, H7); **^{13}C NMR (101 MHz, CDCl$_3$)** δ_C 173.9, 151.8, 146.9, 145.2, 131.0, 129.5, 128.6, 116.8, 111.9, 82.0, 78.0, 37.3, 35.5, 32.7, 31.6, 10.9.

Characterisation for diastereomer b: R$_f$ 0.32 (Petroleum Ether / EtOAc (20:3)); **^{1}H NMR (400 MHz, CDCl$_3$)** δ_H 7.15-7.12 (1H, m, H4), 7.04 (1H, t, J = 7.7 Hz, H11), 6.80 (1H, d, J = 7.2 Hz, H10), 6.58 (1H, d, J = 8.1 Hz, H12), 5.20-5.12 (1H, m, H5), 4.47 (1H, ddd, J = 11.7, 4.2, 2.2 Hz, H6), 3.12 (1H, *app* tt, J = 11.0, 5.2 Hz, H16), 3.03-2.88 (1H, m, H9), 2.75 (1H, dd, J = 15.4, 8.0 Hz, H9), 2.39 (1H, dt, J = 12.3, 6.5 Hz, H8), 2.17 (1H, ddd, J 12.7, 5.1, 2.2 Hz, H7), 1.97 (3H, s, H17), 1.59 (1H, ddt, J = 12.3, 11.0, 8.0 Hz, H8), 1.35 (1H, dt, J = 24.3, 11.7 Hz, H7); **^{13}C NMR (101 MHz, CDCl$_3$)** δ_C 173.8, 152.0, 145.6, 145.1, 131.7, 129.5, 128.7, 116.8, 112.0, 81.4, 76.1, 37.5, 35.5, 32.7, 29.5, 10.9.

1.2.4 Synthesis of the CDEFG ring model system

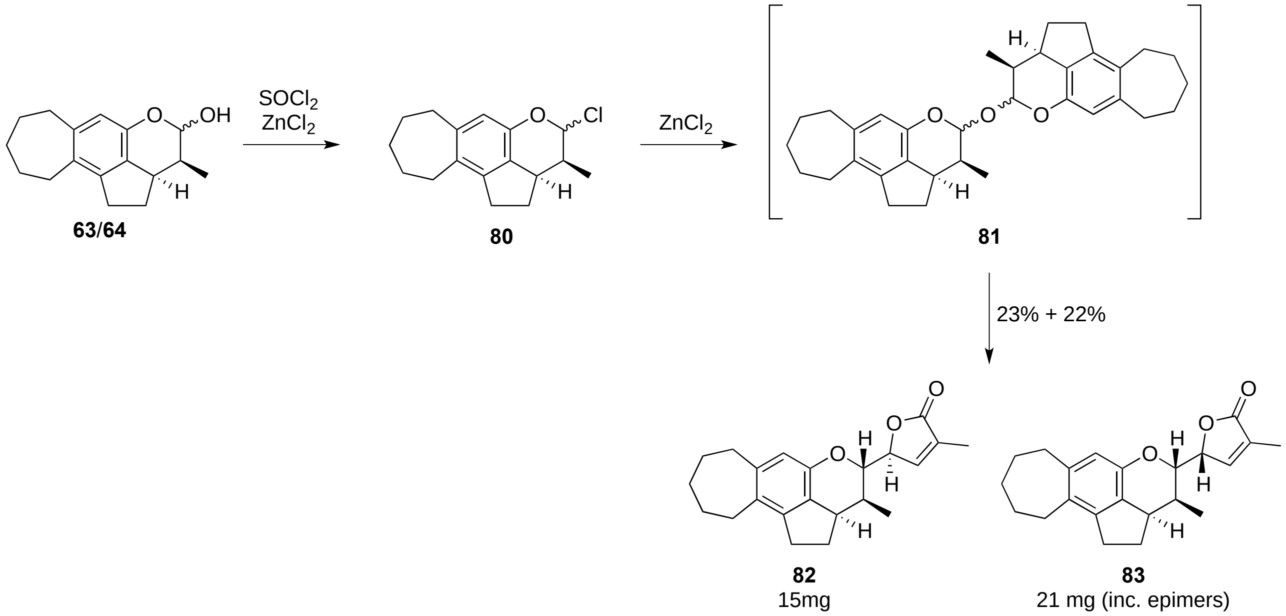

Scheme S7: Synthesis of the CDEFG ring system **82**.

(2a*R*,3*S*)-4-Chloro-3-methyl-1,2,2a,3,4,7,8,9,10,11-decahydrocyclohepta[*g*]cyclopenta[*de*]chromene, 80

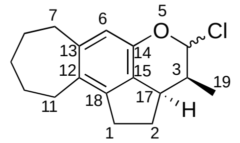

To an oven-dried vial charged with a stirrer bar and ZnCl$_2$ (132 mg, 0.965 mmol, 5.0 equiv.) under argon was added a cooled solution of CDEF rings lactol-aldehyde mixture **63-64** (49.9 mg, 0.193 mmol, 1.0 equiv.) in dry toluene (1.0 mL), followed by thionyl chloride (56 µL, 0.772 mmol, 4.0 equiv.) dropwise. The reaction mixture was stirred at room temperature for 6 h before being filtered through a short pad of oven-dried Celite®, and washed with dry dichloromethane. The solvent was removed *in vacuo* to yield crude chloropyran **80** as a white foam which was used in the next step without further purification. N.B.: this compound is unstable to water and silica.

^{1}H NMR (400 MHz, CDCl$_3$) δ_H 6.45 (1H, s, H6), 6.19 (1H, d, *J* = 2.0 Hz, H4), 3.74 (1H, ddd, *J* = 11.7, 6.9, 5.4 Hz, H17), 2.93-2.78 (2H, m, H1), 2.78-2.64 (4H, m, H7 and H11), 2.50 (1H, qdd, *J* = 7.3, 5.4, 2.0 Hz, H3), 2.20 (1H, dddd, *J* = 11.7, 6.9, 5.6, 1.3 Hz, H2), 1.91-1.71 (3H, m, H9 and H2), 1.70-1.61 (2H, m, H8), 1.60-1.48 (2H, m, H10), 0.85 (3H, d, *J* = 7.3 Hz, H19); **^{13}C NMR (101 MHz, CDCl$_3$)** δ_C 145.1, 144.7, 143.3, 133.4, 123.5, 113.5, 94.5, 37.0, 36.8, 35.7, 32.9, 31.4, 31.2, 30.5, 28.6, 28.0, 12.4; **HRMS (FI$^+$)** calc. for $C_{17}H_{21}OCl$ [M]$^+$: 276.1281; found: 286.1271.

(2a*R*,2a'*R*,3*S*,3'*S*,4*R*,4'*R*)-4,4'-oxybis(3-methyl-1,2,2a,3,4,7,8,9,10,11-decahydrocyclohepta[*g*]cyclopenta[*de*]chromene), 81

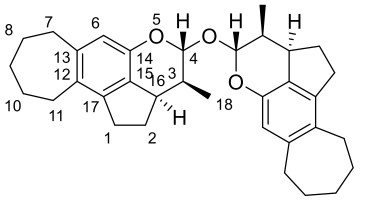

R$_f$ 0.87 (Petroleum Ether / Et$_2$O (4:1)); **ν_{max} (thin film) /cm^{-1}** 2980 (m), 2880 (m), 1610 (m), 1476 (m), 1282 (m), 1126 (s), 1084 (s), 945 (s), 908 (s); **^{1}H NMR (400 MHz, CDCl$_3$)** δ_H 6.47 (1H, s, H6), 5.50 (1H, d, *J* = 1.7 Hz, H4), 3.38 (1H, dt, *J* = 11.4, 6.3 Hz, H16), 2.83- 2.76 (2H, m, H1), 2.76-2.72 (2H, m, H7), 2.72- 2.66 (2H, m, H11), 2.17 (1H, qdd, *J* = 7.2, 6.1, 1.7 Hz, H3), 2.13- 2.02 (1H, m, H2), 1.87-1.76 (2H, m, H9), 1.76-1.71 (1H, m, H2), 1.70-1.55 (4H, m, H8 and H10), 0.69 (3H, d, *J* = 7.2 Hz, H18); **^{13}C NMR (101 MHz, CDCl$_3$)** δ_C 146.9, 144.1, 143.1, 131.6, 124.8, 113.2, 98.1, 36.9, 35.9, 33.0, 31.9, 31.3, 31.2, 29.8, 28.7, 28.2, 10.8; **HRMS (FI$^+$)** calc. for $C_{34}H_{42}O_3$ [M]$^+$: 498.3134; found: 498.2904.

(*S*)- and (*R*)- 3-Methyl-5-((2a*R*,3*S*,4*S*)-3-methyl-1,2,2a,3,4,7,8,9,10,11-decahydrocyclohepta[*g*]cyclopenta[*de*]-chromen-4-yl)furan-2(5*H*)-one, 82 and 83

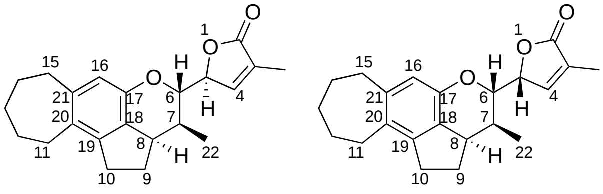

82 **83**

To an oven-dried vial charged with a stirrer bar and $ZnCl_2$ (10.5 mg, 0.077 mmol, 0.4 equiv.) under argon at –40 °C was added a cooled solution of the above chloropyran **80** and siloxyfuran **67** (98 mg, 0.386 mmol, 2.0 equiv.) in dry dichloromethane (1.3 mL). The reaction mixture was stirred warming to room temperature overnight before being filtered through a short pad of oven-dried Celite®, and washed with dry dichloromethane. The solvent was removed *in vacuo* and the crude product was purified by flash column chromatography on silica (9:1 → 7:3 Petroleum Ether / Et$_2$O) to yield CDEFG rings **82** (15.1 mg, 23%) and its C5-epimer **83** (20.7 mg, as a 71:14:14 mixture with its C6-epimer and another unidentified compound, 22%) as white foams.

Characterisation for 82: R$_f$ 0.11 (Petroleum Ether / Et$_2$O (4:1)); **ν_{max} (thin film) /cm^{-1}** 2922 (s), 1760 (s), 1608 (m), 1475 (m), 1364 (w), 1287 (m), 1058 (m), 999 (m); **^{1}H NMR (400 MHz, CDCl$_3$)** δ_H 7.07 (1H,

quin, J = 1.7 Hz, H4), 6.46 (1H, s, H16), 5.06 (1H, dquin, J = 7.2, 1.7 Hz, H5), 4.03 (1H, dd, J = 7.2, 1.6 Hz, H6), 3.17 (1H, *app* dt, J = 10.9, 6.1 Hz, H8), 2.80 (2H, dd, J = 9.6, 3.4 Hz, H10), 2.72 (2H, dt, J = 7.1, 2.8 Hz, H15), 2.70-2.67 (2H, m, H11), 2.29 (1H, qdd, J = 7.1, 5.4, 1.6 Hz, H7), 2.19-2.11 (1H, m, H9), 1.95 (3H, t, J = 1.7 Hz, H23), 1.88-1.80 (1H, m, H13), 1.80-1.69 (2H, m, H13 and H9), 1.69-1.63 (2H, m, H14 and H12), 1.63-1.47 (2H, m, H14 and H12), 0.87 (3H, d, J = 7.1 Hz, H22); **^{13}C NMR (101 MHz, $CDCl_3$)** δ_C 173.6, 147.7, 144.9, 144.4, 143.2, 132.3, 131.6, 122.4, 113.5 82.5, 82.1, 37.9, 36.8, 32.9, 31.3, 31.1, 30.9, 30.2, 28.6, 28.2, 13.7, 11.1; **HRMS (ESI$^+$)** calc. for $C_{22}H_{26}NaO_3$ $[M+Na]^+$: 361.1774; found: 361.1771.

Characterisation for 83: R_f 0.43 (Petroleum Ether / Et_2O (4:1)); **ν_{max} (thin film) /cm^{-1}** 2925 (s), 1761 (s), 1605 (m), 1476 (m), 1364 (w), 1289 (m), 1059 (m), 999 (m); **^{1}H NMR (400 MHz, $CDCl_3$)** δ_H 7.38 (1H, quin, J = 1.7 Hz, H4), 6.41 (1H, s, H16), 4.93 (1H, dquin, J = 10.0, 1.7 Hz, H5), 3.79 (1H, dd, J = 10.0, 1.6 Hz, H6), 3.37 (1H, *app* dt, J = 11.0, 6.0 Hz, H8), 2.85-2.79 (2H, m, H10), 2.77-2.65 (4H, m, H15 and H11), 2.63 (1H, qdd, J = 7.2, 5.5, 1.7 Hz, H7), 2.24-2.17 (1H, m, H9), 1.95 (3H, t, J = 1.7 Hz, H23), 1.99-1.82 (1H, m, H13), 1.82-1.73 (2H, m, H13 and H9), 1.73-1.63 (2H, H14 and H12), 1.62-1.45 (2H, m, H14 and H12), 0.83 (3H, d, J = 7.2 Hz, H22); **^{13}C NMR (101 MHz, $CDCl_3$)** δ_C 174.1, 149.1, 147.8, 144.8, 143.9, 131.8, 130.0, 122.9, 112.8, 82.3, 78.5, 36.9, 36.9, 32.9, 31.3, 31.2, 30.8, 28.7, 28.6, 28.2, 13.0, 10.9; **HRMS (ESI$^+$)** calc. for $C_{22}H_{26}NaO_3$ $[M+Na]^+$: 361.1774; found: 361.1768.

Stereochemical assignment of 82 and 83

The stereochemical assignment of **82** and **83** was achieved by 2D NOESY NMR experiments, and by comparison of the NMR spectra of both C5-epimers (in d_5-pyridine) with the natural product. The NOESY spectra showed that both compounds possess the same stereochemistry in the F ring (Figure 1), with H6 showing through-space correlations with the hydrogen atoms of the C22 methyl group. However, differences in the through-space interactions of H4 and H5 with hydrogens on the F ring suggest a difference in stereochemistry at C5. Blue = top face nOe. Red = Bottom face nOe.

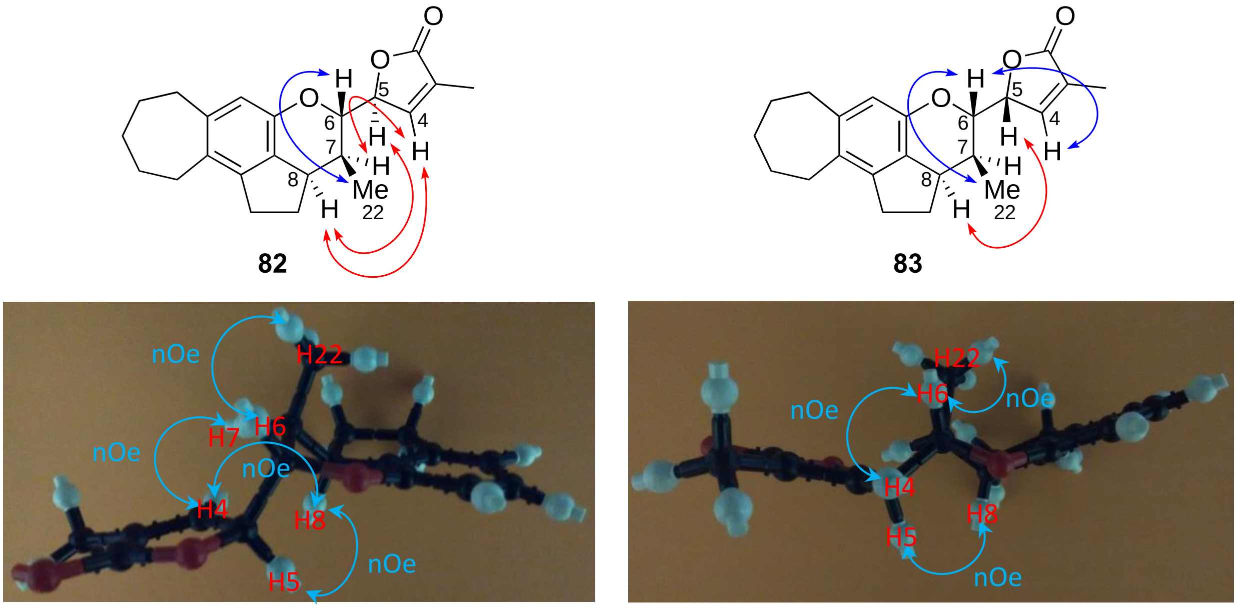

Figure 1. NOESY correlations for CDEFG rings C5-epimers **82** and **83.** Blue = top face nOe. Red = Bottom face nOe.

For **82**, the NOE correlations between H5 and H8, and from H4 to both H7 and H8 are consistent with the expected through-space interactions in the structure possessing (5*S*)-stereochemistry as shown in Figure 1 (left). Here, the model is orientated in a conformation in which H5 and H6 are anti-periplanar, where there is little steric overlap between the axial C22 methyl group and butenolide G ring substituents. In contrast, NOE correlations between H5 and C8, and from H4 to H6 observed for **83** matched the structure of the (5*R*)-epimer. These through-space interactions are evident in the model (right, Figure 1) orientated in a preferred conformation where H5 is anticlinal to H6, and where there is little steric overlap between the equatorial C22 methyl group and butenolide G ring substituents. The structural information elucidated from these spectra indicated that **82** was the desired C5-epimer.

To corroborate our assignment based on 2D NOESY NMR, a comparison of the key ^{13}C chemical shifts of both C5-epimers with rubriflordilactone A was conducted. Table 1 shows a strong correlation between the ^{13}C NMR shifts of compound **82** with rubriflordilactone A, supporting our assignment of this compound as the desired epimer. In contrast, compound **83** showed significant deviations in its ^{13}C NMR shifts from the natural product.

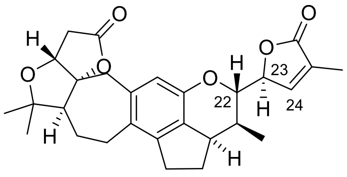

**Carbon No.**	**δ_C / ppm (C_5D_5N)**		
	**Rubriflordilactone A**	**82**[a]	**83**[a]
**22**	83.5	83.3	82.4
**23**	82.2	82.8	79.4
**24**	145.1	145.9	149.6

[a] Data recorded in C_5D_5N on a Bruker AVII 500 MHz spectrometer with cryoprobe (^1H, ^{13}C, COSY, HSQC, HMBC).

Table 1. Comparison of key ^{13}C signals for CDEFG rings C5-epimers 82 and 83 with rubriflordilactone A.

1.2.5. Rubriflordilactone A (2) and C23-*epi*-rubriflordilactone A (94)

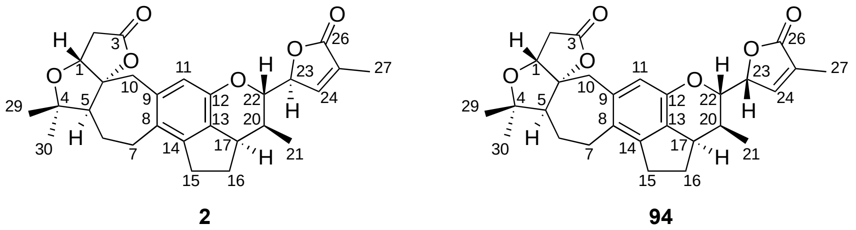

An oven-dried flask equipped with a stirrer bar and charged with anhydrous $ZnCl_2$ (2.9 mg, 0.021 mmol, 0.4 equiv.) was heated to 150 °C under vacuum overnight to remove any traces of water, cooled to RT and refilled with argon, then cooled to –30 °C. In a separate flask, to a solution of crude chloropyran **92** in dichloromethane (0.8 mL) under argon at –30 °C was added triisopropyl((3-methylfuran-2-yl)oxy)silane **55** (27.1 mg, 0.107 mmol, 2.0 equiv.). The resulting solution was transferred to the flask containing $ZnCl_2$, and the reaction mixture was stirred, warming to RT, overnight. The reaction mixture was then filtered through a short pad of Celite®, and washed with dichloromethane. The solvent was removed *in vacuo* and the crude product was purified by flash chromatography (9:1 → 4:1 → 1:1 Petroleum Ether / EtOAc) to yield rubriflordilactone A **2** (9.3 mg, 0.020 mmol, 38%) as a white solid and its C-23 epimer **94** (8.2 mg, 0.018 mmol, 33%) as a white solid.

Characterization for rubriflordilactone A (2): R*f* 0.28 (1:1 Petroleum Ether / EtOAc); $[\alpha]_D^{25}$ +58.3 (*c* = 0.114, MeOH); ν_{max} **(thin film) /cm^{-1}** 2970, 2341, 2327, 1760, 1610, 1479, 1199, 1025, 934, 654; **^{1}H NMR** (500 MHz, C_5H_5N) δ_H 7.30 (1H, s, H24), 6.52 (1H, s, H11), 5.07 (1H, d, *J* = 7.9 Hz, H23), 4.33 (1H, d, *J* = 6.1 Hz, H1), 4.08 (1H, dd, *J* = 7.8, 1.3 Hz, H22), 3.56 (1H, d, *J* = 15.6 Hz, H19), 3.26-3.17 (1H, m, H17), 3.20 (1H, dd, *J* = 18.3, 6.1 Hz, H2), 3.04 (1H, dd, *J* = 17.3 and 3.9 Hz, H7), 2.87 (1H, d, *J* = 15.6 Hz, H19), 2.85 (1H, d, *J* = 18.3 Hz, H2), 2.71 (1H, dd, *J* = 15.3, 8.0 Hz, H15), 2.67-2.56 (2H, m, H7 and H15), 2.38-2.31 (2H, m, H5 and H20), 2.09 (1H, dt, *J* = 11.5, 6.6 Hz, H16), 1.95-1.83 (1H, m, H6), 1.92 (3H, s, H27), 1.81-1.68 (1H, m, H16), 1.66-1.57 (1H, m, H6), 1.35 (3H, s, H30), 1.13 (3H, s, H29), 0.85 (3H, d, *J* = 7.0 Hz, H21); **^{13}C NMR** (126 MHz, C_5H_5N) δ_c 176.0 (C3), 174.2 (C26), 148.6 (C12), 145.5 (C14), 145.5 (C24), 134.5 (C8), 132.4 (C25), 127.1 (C9), 125.2 (C13), 116.5 (C11), 99.6 (C10), 84.5 (C4), 83.9 C22), 82.6 (C23), 80.3 (C1), 60.6 (C5), 41.0 (C19), 38.2 (C17), 36.5 (C2), 31.7 (C15), 31.2 (C7), 31.2 (C16), 30.5 (C20), 28.8 (C29), 24.5 (C6), 21.4 (C30), 13.6 (C21), 11.3 (C27); **HRMS** (ES^+) calc. for $C_{28}H_{32}NaO_6$ $[M+Na]^+$ 487.2091; found 487.2092. Data in accordance with literature.[4-6]

Data for C23-*epi*-rubriflordilactone A (94): R*f* 0.45 (1:1 petroleum ether/ EtOAc); $[\alpha]_D^{25}$ +64.3 (*c* = 0.114, MeOH); ν_{max} **(thin film) /cm^{-1}** 2954, 2361, 1756, 1613, 1485, 1198, 1062, 931, 813, 670; **^{1}H NMR** (500 MHz, $CDCl_3$) δ_H 7.32 (1H, app. quint, *J* = 1.6 Hz, H24), 6.59 (1H, s, H11), 4.90 (1H, app dquint, *J* = 9.5 and 1.7 Hz, H23), 4.36 (1H, d, *J* = 5.9 Hz, H1), 3.92 (1H, dd, *J* = 9.5 and 1.6 Hz, H22), 3.62 (1H, d, *J* = 15.6 Hz, H19), 3.34 (1H, dt, *J* = 11.5 and 6.0 Hz, H17), 3.18 (1H, dd, *J* = 18.4 and 6.1 Hz, H2), 3.03 (1H, m, H7), 2.93 (1H, d, *J* = 15.6 Hz, H19), 2.87 (1H, d, *J* = 18.4 Hz, H2), 2.68 (1H, dd, *J* = 15.4 and 8.3 Hz, H15), 2.65-2.57 (2H, m, H15 and H7), 2.55 (1H, qdd, *J* = 7.1, 5.6 and 1.5 Hz, H20), 2.37 (1H, dd, *J* = 12.6 and 3.3 Hz, H5), 2.05 (1H, dt, *J* = 6.4 and 11.9 Hz, H16), 1.93 (1H, m, H6), 1.87 (3H, app t, *J* = 1.7 Hz, H27), 1.70 (1H, ddd, *J* = 19.6, 11.3 and 8.5 Hz, H16), 1.62 (1H, ddt, *J* = 14.1, 6.1 and 3.3 Hz, H6), 1.36 (3H, s, H30), 1.14

(3H, s, H29), 0.80 (3H, d, *J* = 7.1 Hz, H21); ^{13}**C NMR** (126 MHz, $CDCl_3$) δ_C 176.1 (C3), 174.3 (C26), 149.7 (C24), 149.0 (C12), 145.8(C14), 134.9 (C8), 130.2 (C25), 127.2 (C8), 125.4 (C13), 115.9 (C11), 99.8 (C10), 84.7 (C4), 82.7 (C22), 80.4 (C1), 79.5 (C23), 60.3 (C5), 41.0 (C19), 37.4 (C17), 36.5 (C2), 31.8 (C15), 31.3 (C7), 31.2 (C16), 29.5 (C20), 28.9 (C30), 24.5 (C6), 21.5 (C29), 13.4 (C21), 11.1 (C27); **HRMS** (ES^+) calc. for $C_{28}H_{32}NaO_6$ $[M+Na]^+$ 487.2091; found 487.2092.

Comparison Table of ^{1}H NMR data for rubriflordilactone A

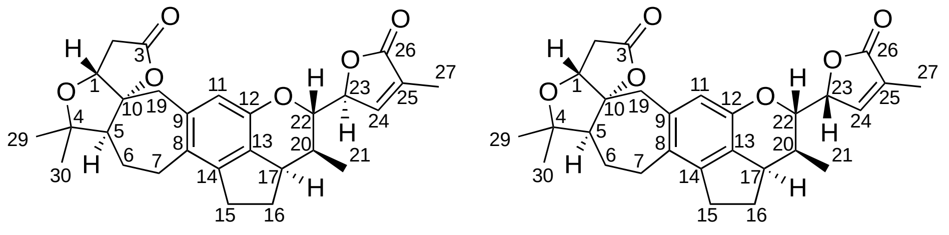

atom	Natural (Sun)[9] δH, [ppm, mult., *J* (Hz)] 400 MHz		Synthetic (Li)[10] δH, [ppm, mult., *J* (Hz)] 500 MHz		Synthetic (Anderson) δH, [ppm, mult., *J* (Hz)] 500 MHz	
1	4.30	1H, d, 6.1	4.32	1H, d, 6.1	4.33	1H, d, 6.1
2α	2.83	1H, d, 18.3	2.85	1H, d, 18.3	2.85	1H, d, 18.3
2β	3.19	1H, dd, 18.3, 6.1	3.21	1H, dd, 18.3, 6.1	3.21	1H, dd, 18.3, 6.2
5	2.32	1H, overlapped	2.35	1H, dd, 12.6, 2.0	2.36	1H, dd, 12.5, 3.2
6α	1.83	1H, m	1.96-1.83	1H, m	1.95-1.83	1H, m
6β	1.58	1H, m	1.65-1.58	1H, m	1.66-1.57	1H, m
7α	2.99	1H, dd, 16.2, 2.6	3.02	1H, dd, 17.3, 4.0	3.03	1H, dd, 17.3, 3.9
7β	2.71	1H, overlapped	2.70	1H, dd, 15.4, 8.2	2.70	1H, dd, 15.4, 8.5
11	6.50	1H, s	6.52	1H, s	6.52	1H, s
15α	2.59	1H, overlapped	2.67-2.57	1H, m	2.67-2.56	1H, m
15β	1.69	1H, m	1.78-1.67	1H, m	2.71	1H, dd, 15.3, 8.0
16α	2.67	1H, m	2.67-2.57	1H, m	1.81-1.68	1H, m
16β	2.06	1H, m	2.11-2.05	1H, m	2.09	1H, dt, 11.8, 6.3
17	3.21	1H, m	3.26-3.20	1H, m	3.26-3.20	1H, m
19α	2.84	1H, d, 15.6	2.87	1H, d, 15.6	2.87	1H, d, 15.6
19β	3.54	1H, d, 15.6	3.56	1H, d, 15.6	3.56	1H, d, 15.6
20	2.30	1H, m	2.34-2.29	1H, m	2.33-2.30	1H, m
21	0.82	3H, d, 7.1	0.84	3H, d, 6.8	0.85	3H, d, 7.0
22	4.05	1H, dd, 7.8, 1.3	4.08	1H, d, 8.0	4.08	1H, d, 8.0
23	4.96	1H, overlapped	5.08-5.04	1H, m	5.07	1H, d, 7.9
24	7.29	1H, br s	7.31	1H, s	7.30	1H, s
27	1.89	3H, s	1.92	3H, s	1.92	3 H, s
29	1.10	3H, s	1.13	3H, s	1.13	3H, s
30	1.32	3H, s	1.35	3H, s	1.35	3H, s

Blue highlighted rows indicate a reassignment of data based on our HSQC and COSY experiments; see the spectral data below for copies of these spectra.

Comparison Table of ^{13}C NMR data for rubriflordilactone A

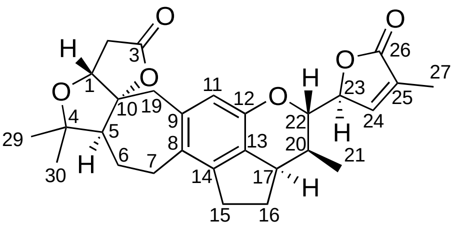

1

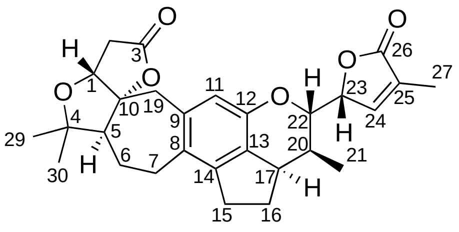

30

atom	Natural (Sun)[9] δ_C, [ppm] 101 MHz	Synthetic (Li)[10] δ_C, [ppm] 125 MHz	Synthetic (Anderson) δ_C, [ppm] 125 MHz
1	80.0	80.4	80.4
2	36.1	36.6	36.5
3	175.5	176.0	175.9
4	84.1	84.6	84.5
5	60.2	60.7	60.6
6	24.1	24.5	24.5
7	30.8	31.3	31.2
8	134.5	135.0	134.9
9	126.7	127.2	127.1
10	99.1	99.6	99.5
11	116.1	116.6	116.5
12	148.3	148.7	148.7
13	124.8	125.2	125.2
14	145.1	145.6	145.5
15	31.3	31.8	31.7
16	30.0	30.6	31.2
17	37.8	38.2	38.2
19	40.7	41.1	41.1
20	30.1	31.3	30.5
21	13.2	13.7	13.6
22	83.5	84.0	83.9
23	82.2	82.7	82.6
24	145.1	145.6	145.5
25	132.0	132.5	132.4
26	171.9	174.2	174.2
27	10.8	11.4	11.3
29	28.4	28.8	28.8
30	21.0	21.5	21.4

Blue highlighted rows indicate a reassignment of data based on our HSQC and COSY experiments; see the spectral data below for copies of these spectra.

2. REFERENCES

1. B. Gockel, S. S. Goh, E. J. Puttock, H. Baars, G. Chaubet, E. A. Anderson, *Org. Lett.* **2014**, *16*, 4480.
2. S. S. Goh, H. Baars, B. Gockel, E. A. Anderson, *Org. Lett.* **2012**, *14*, 6278.
3. L. Minuti, A. Taticchi, A. Marrocchi, D. Lanari, A. Broggi, and E. Gacs-Baitz, *Tetrahedron: Asymmetry*, **2003**, *14*, 481–487.
4. W.-L. Xiao, L.-M. Yang, N.-B. Gong, L. Wu, R.-R. Wang, J.-X. Pu, X.-L. Li, S.-X. Huang, Y.-T. Zheng, R.-T. Li, Y. Lu, Q.-T. Zheng, and H.-D. Sun, *Org. Lett.*, **2006**, *8*, 991–994.
5. J. Li, P. Yang, M. Yao, J. Deng, and A. Li, *J. Am. Chem. Soc.*, **2014**, *136*, 16477–16480.
6. S. S. Goh, G. Chaubet, B. Gockel, M.-C. A. Cordonnier, H. Baars, A. W. Phillips, E. A. Anderson, *Angew. Chem.* **2015**, *127*, 12809; *Angew. Chem. Int. Ed.* **2015**, *54*, 12618.

3. NMR Spectra

(2*Z*,5*Z*)-2,5-bis(2-((*tert*-butyldimethylsilyl)oxy)ethylidene)-3-methylenehexane-1,6-diol, 27

^{1}H NMR (500 MHz, $CDCl_3$)

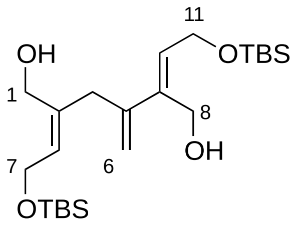

27

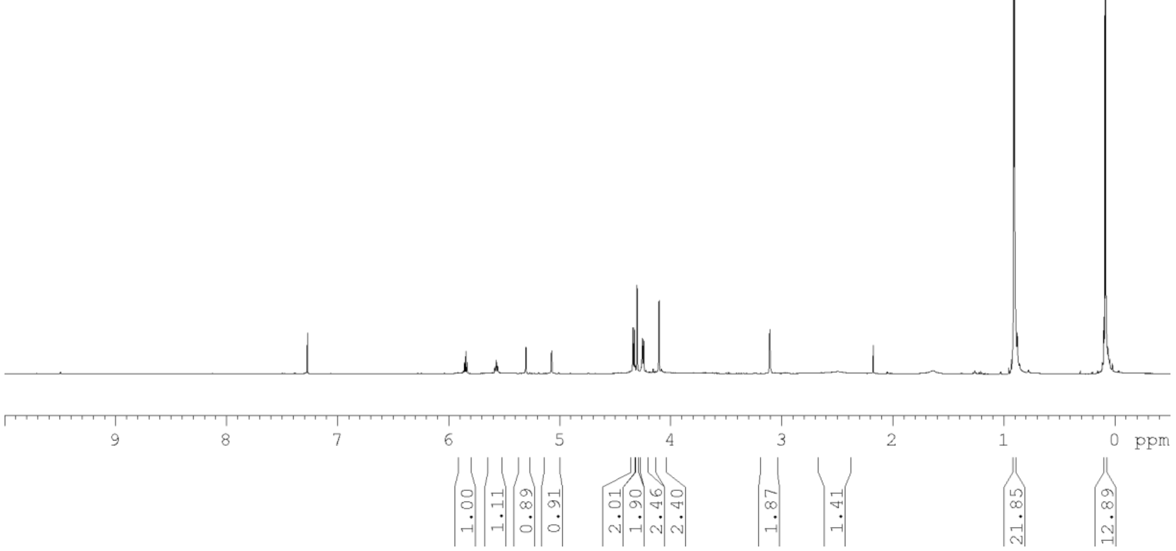

^{13}C NMR (125 MHz, $CDCl_3$)

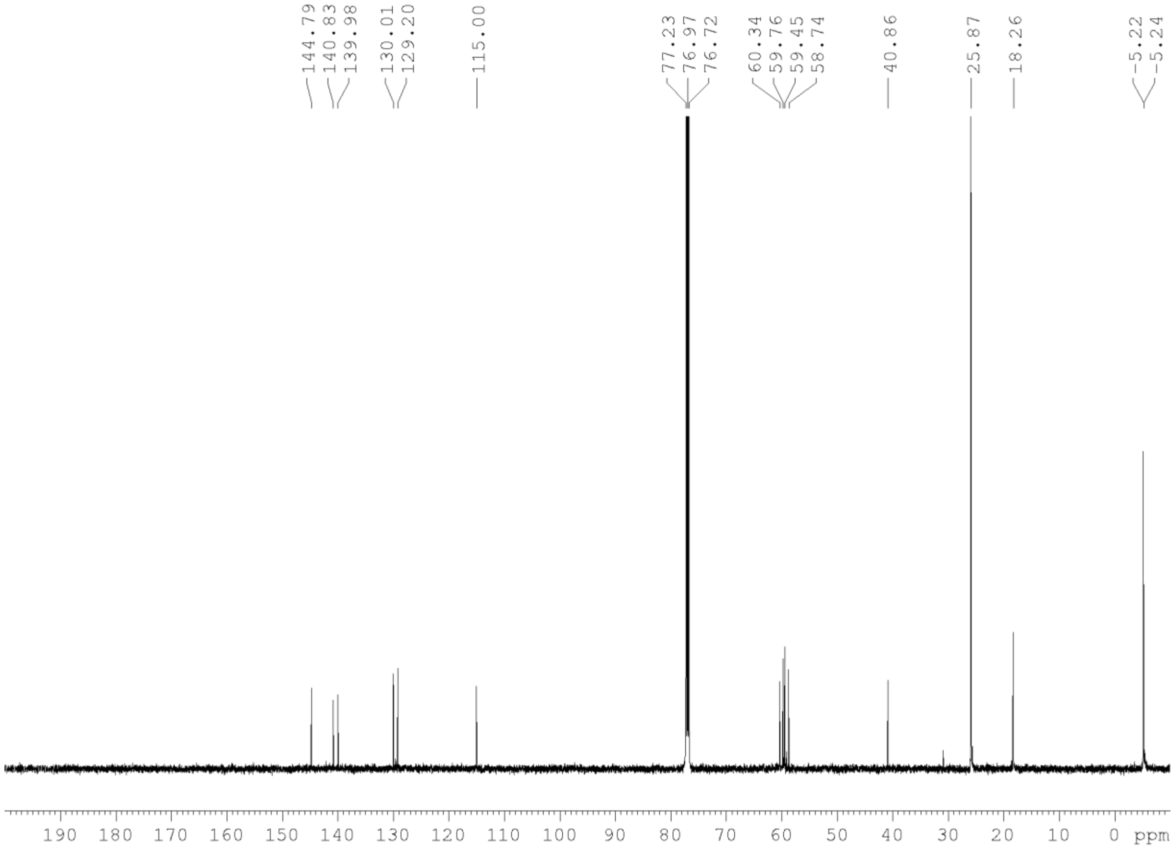

(*R*)-3-Allyl-6-bromo-4-hydroxy-4-((4-methoxybenzyloxy)methyl)hept-6-en-2-one, 22

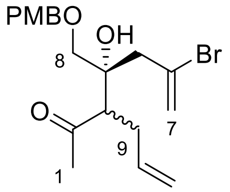

^{1}H (500 MHz, $CDCl_3$)
^{13}C (125 MHz, $CDCl_3$)

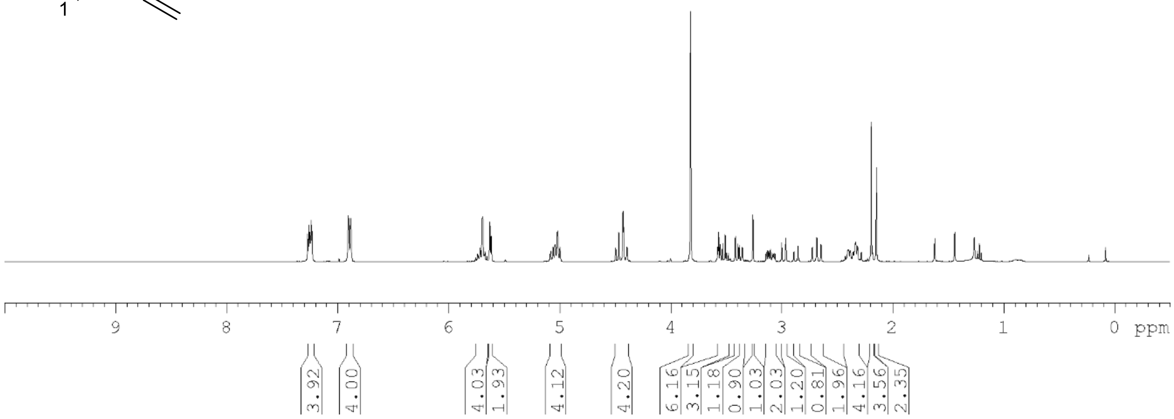

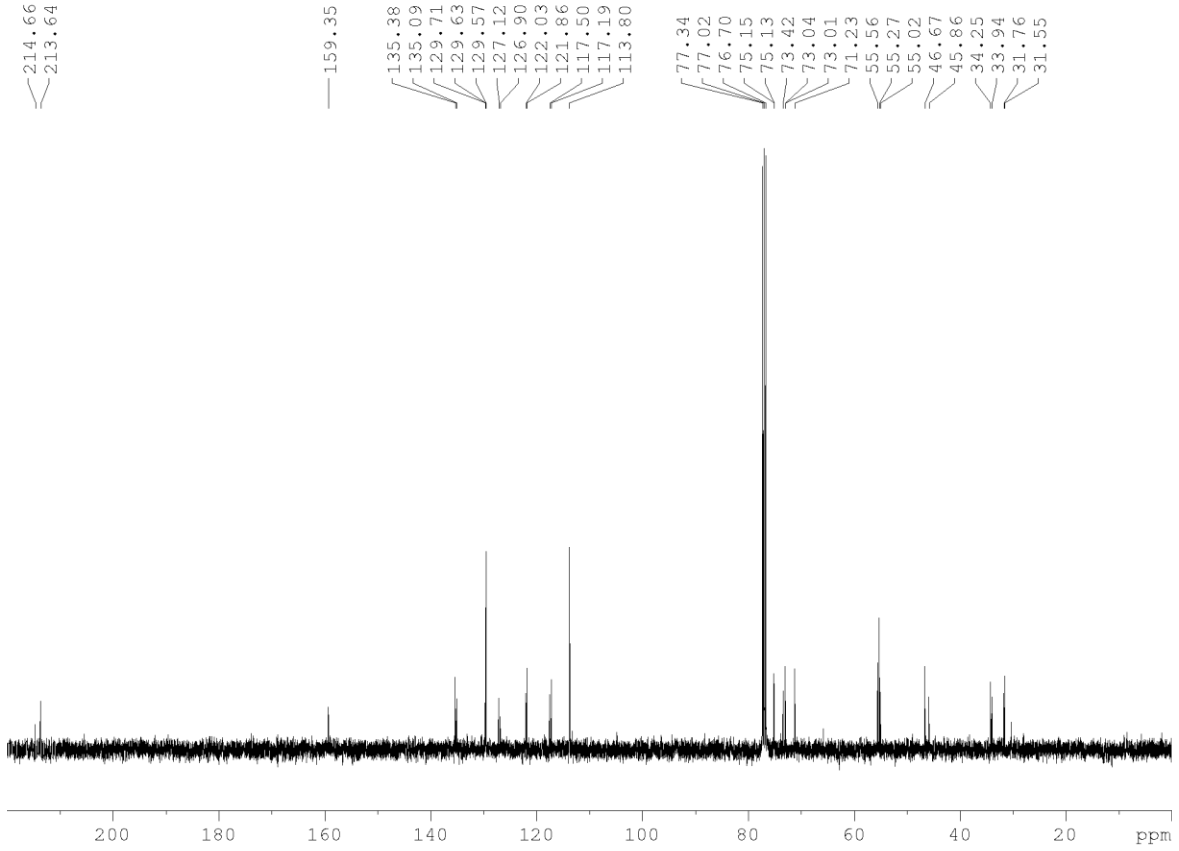

(*Z*)-4-((*tert*-butyldimethylsilyl)oxy)-2-(3-hydroxyprop-1-en-2-yl)but-2-en-1-yl methyl carbonate 28 and (*Z*)-5-((*tert*-butyldimethylsilyl)oxy)-3-(hydroxymethyl)-2-methylenepent-3-en-1-yl methyl carbonate, 29

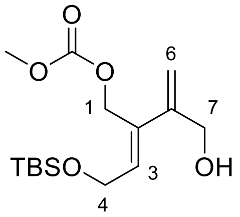

28
major

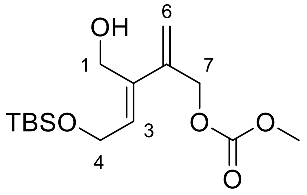

29
minor

^{1}H (400MHz, $CDCl_3$)
^{13}C (101MHz, $CDCl_3$)

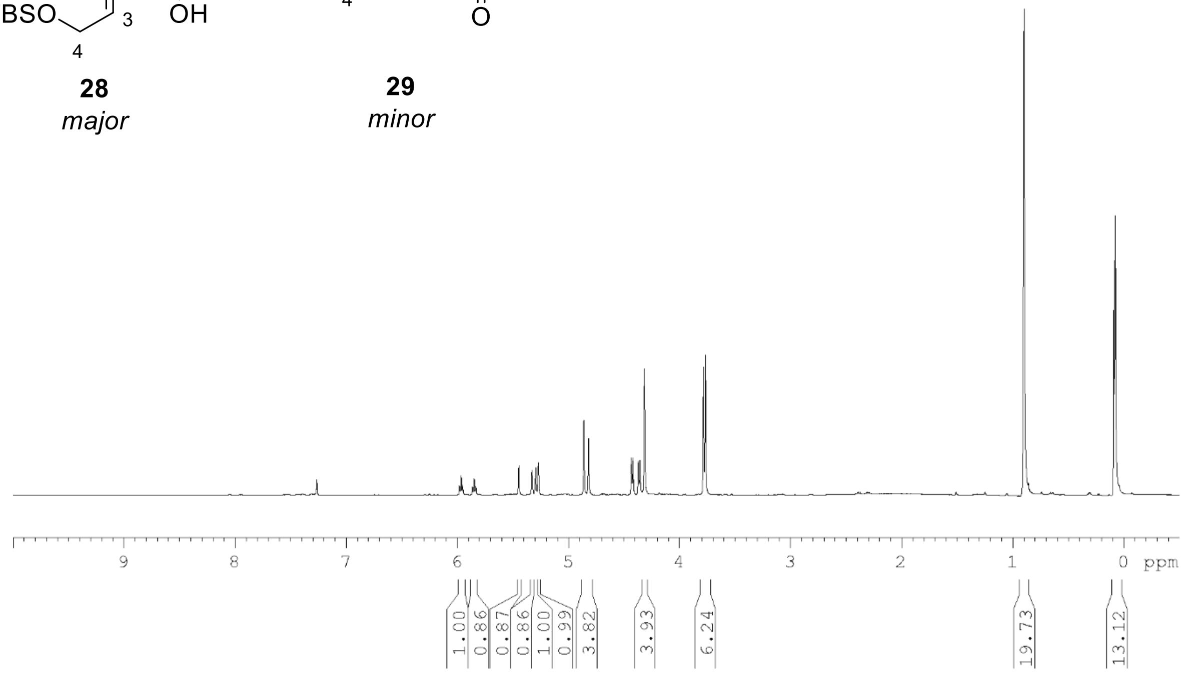

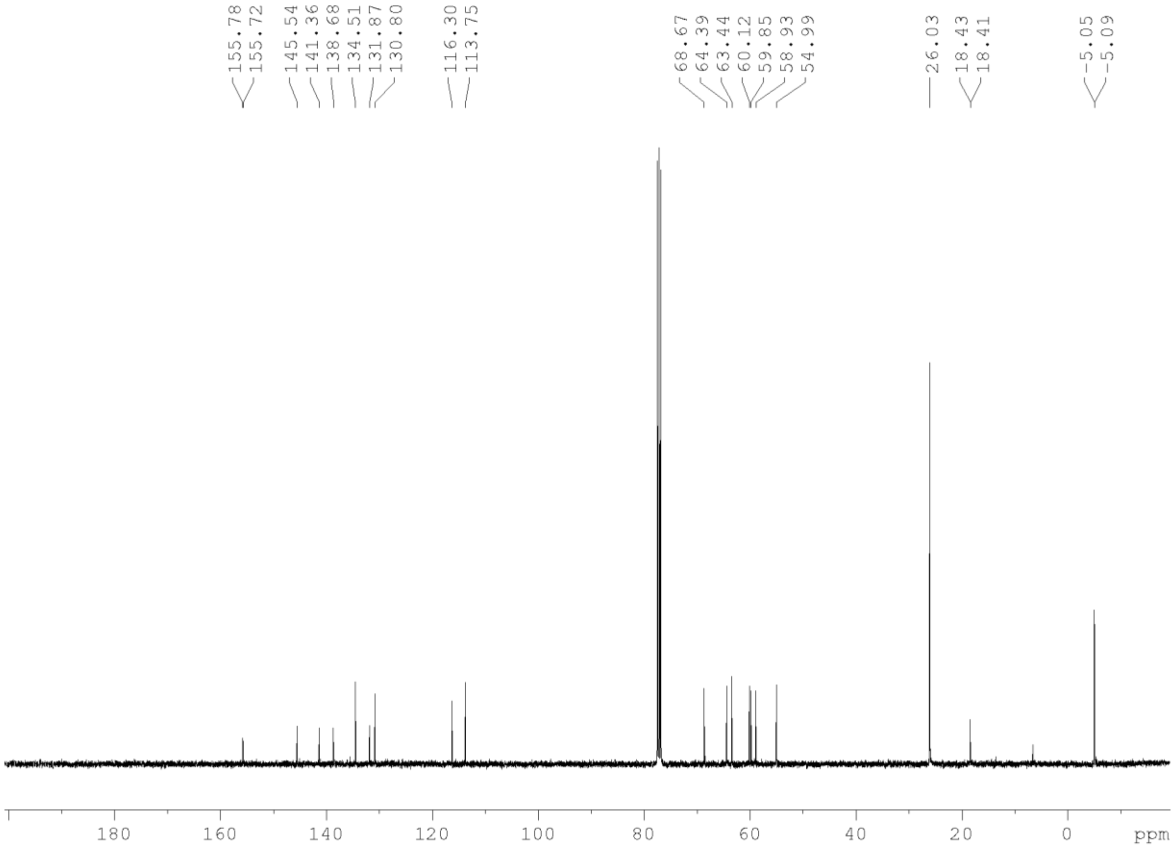

(12*R*,13*R*,*E*)-12-((Benzyldimethylsilyl)ethynyl)-2-bromo-13-methylhexadeca-1,14-dien-8-yn-7-ol, S6

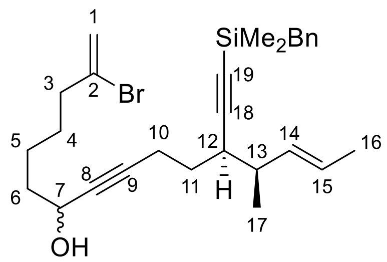

^{1}H (500 MHz, $CDCl_3$)
^{13}C (125 MHz, $CDCl_3$)

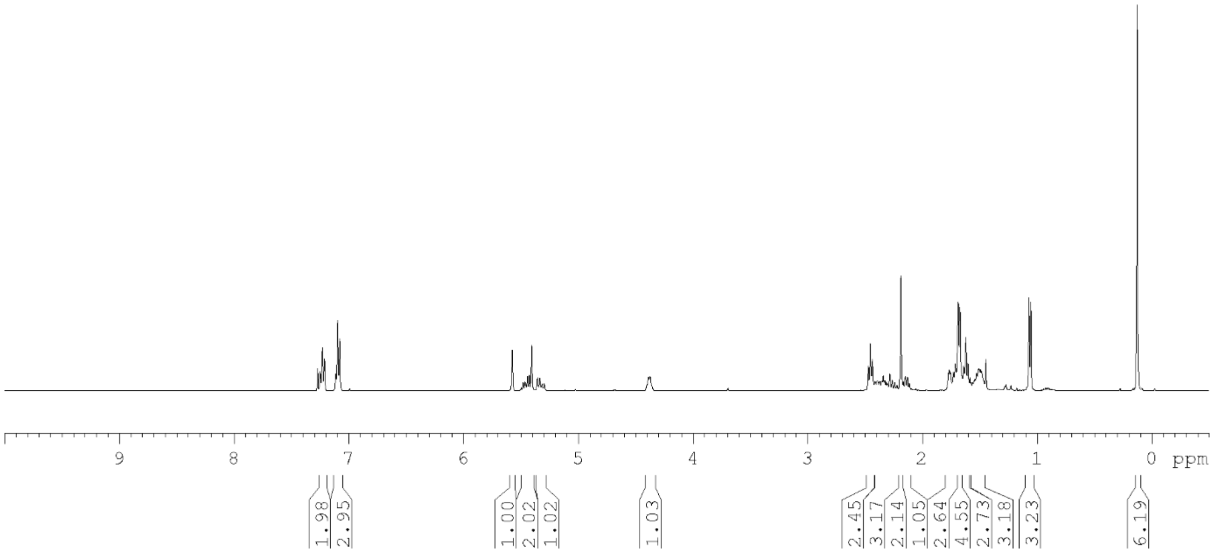

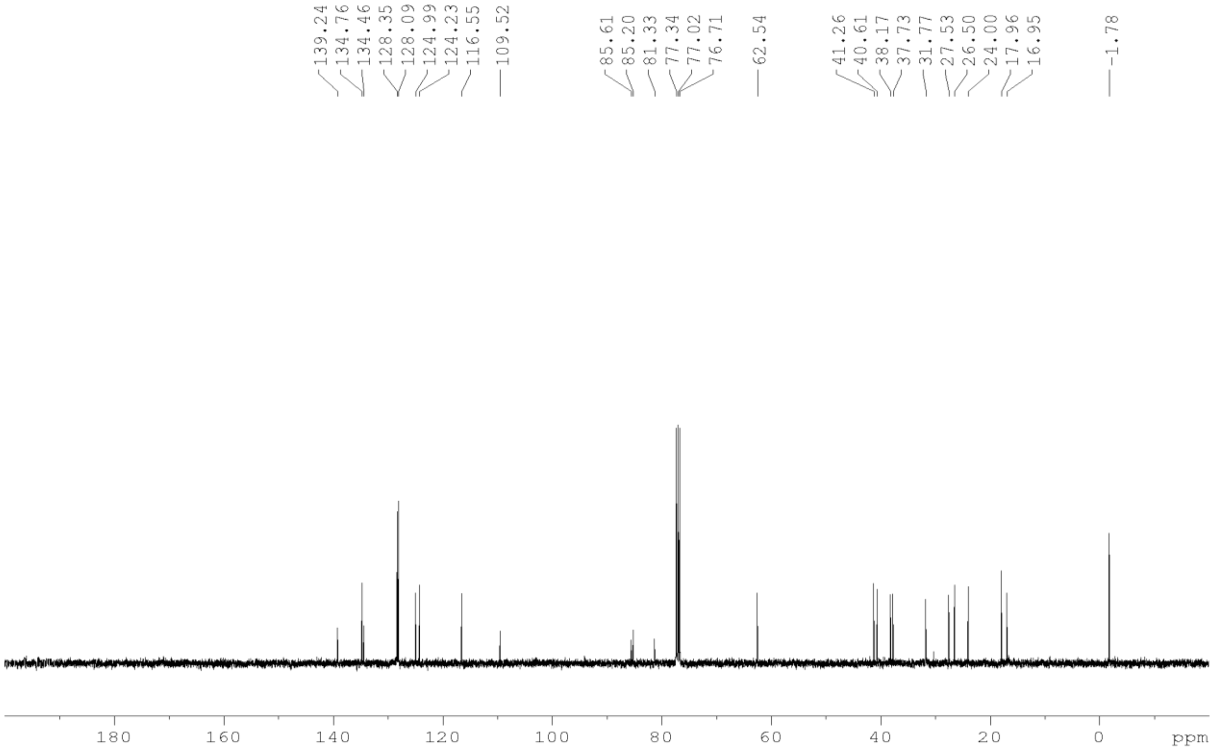

Benzyl((3*R*)-13-bromo-8-((*tert*-butyldimethylsilyl)oxy)-3-((*R*,*E*)-pent-3-en-2-yl)tetradeca-13-en-1,6-diyn-1-yl)dimethylsilane, 51

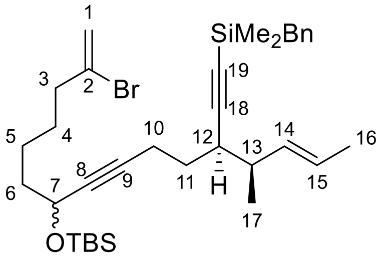

^{1}H (500 MHz, $CDCl_3$)
^{13}C (125 MHz, $CDCl_3$)

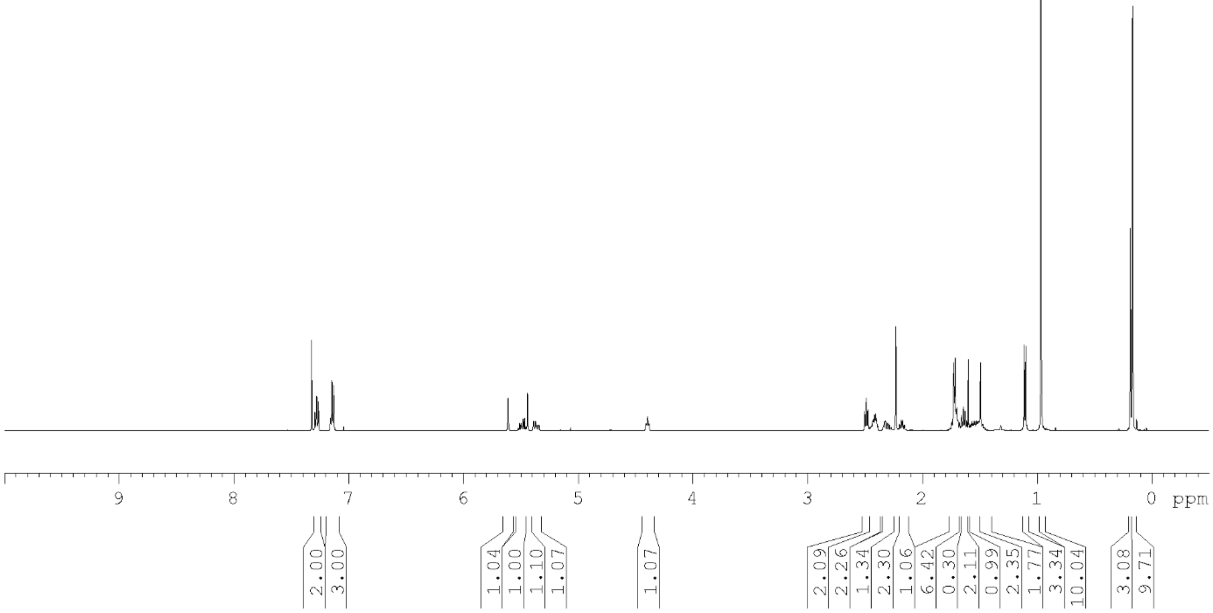

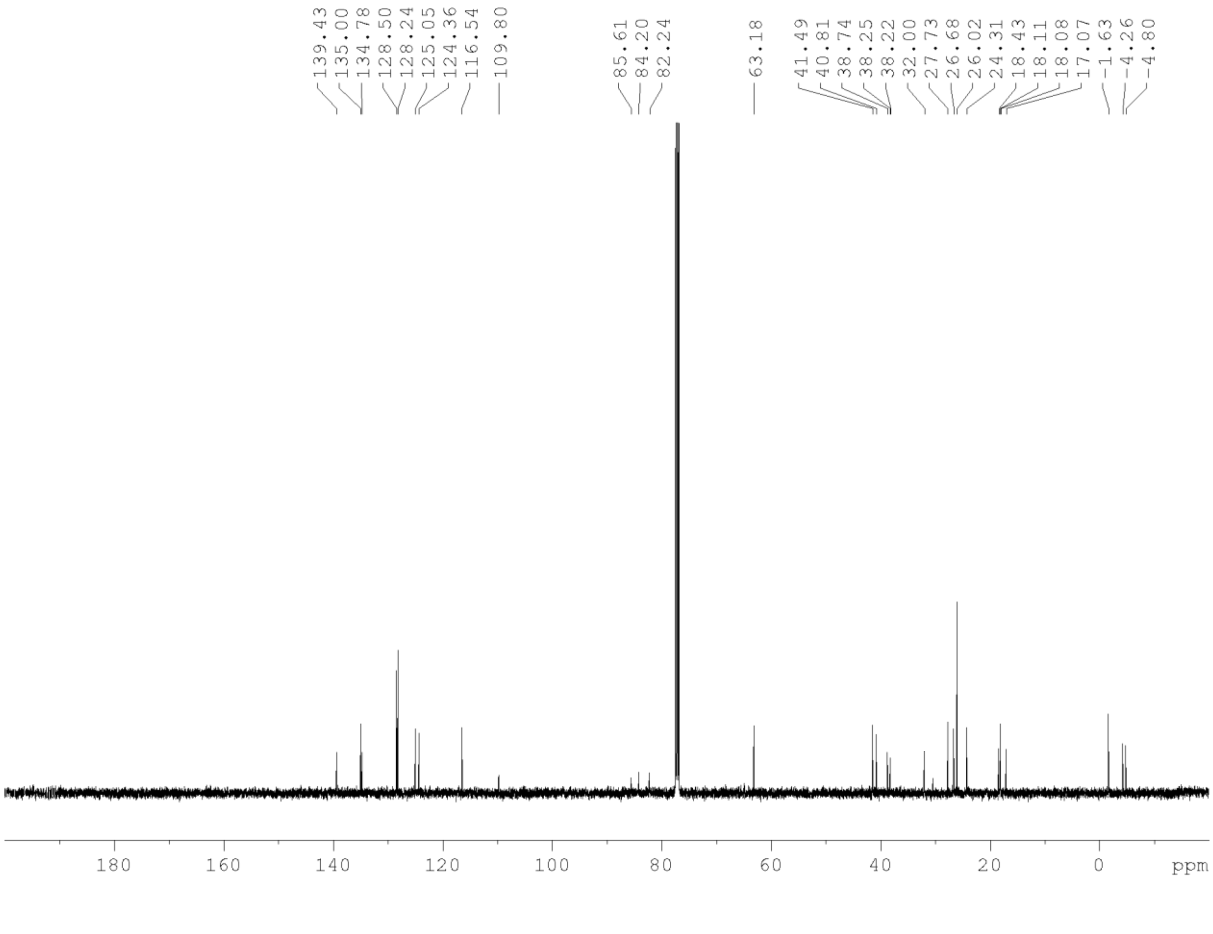

Benzyl((3*R*)-10-((*tert*-butyldimethylsilyl)oxy)-3-((*R*,*E*)-pent-3-en-2-yl)-1,2,3,6,7,8,9,10-octahydrocyclohepta[*e*]inden-4-yl)dimethylsilane, 53

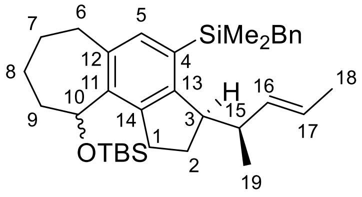

^{1}H (500 MHz, $CDCl_3$)
^{13}C (125 MHz, $CDCl_3$)

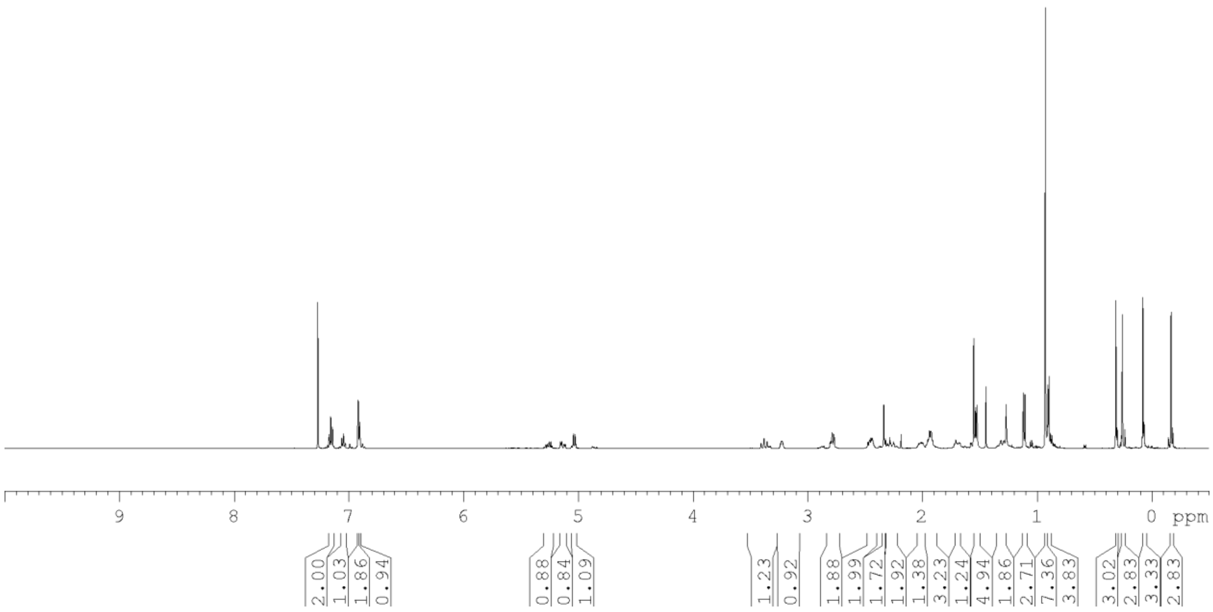

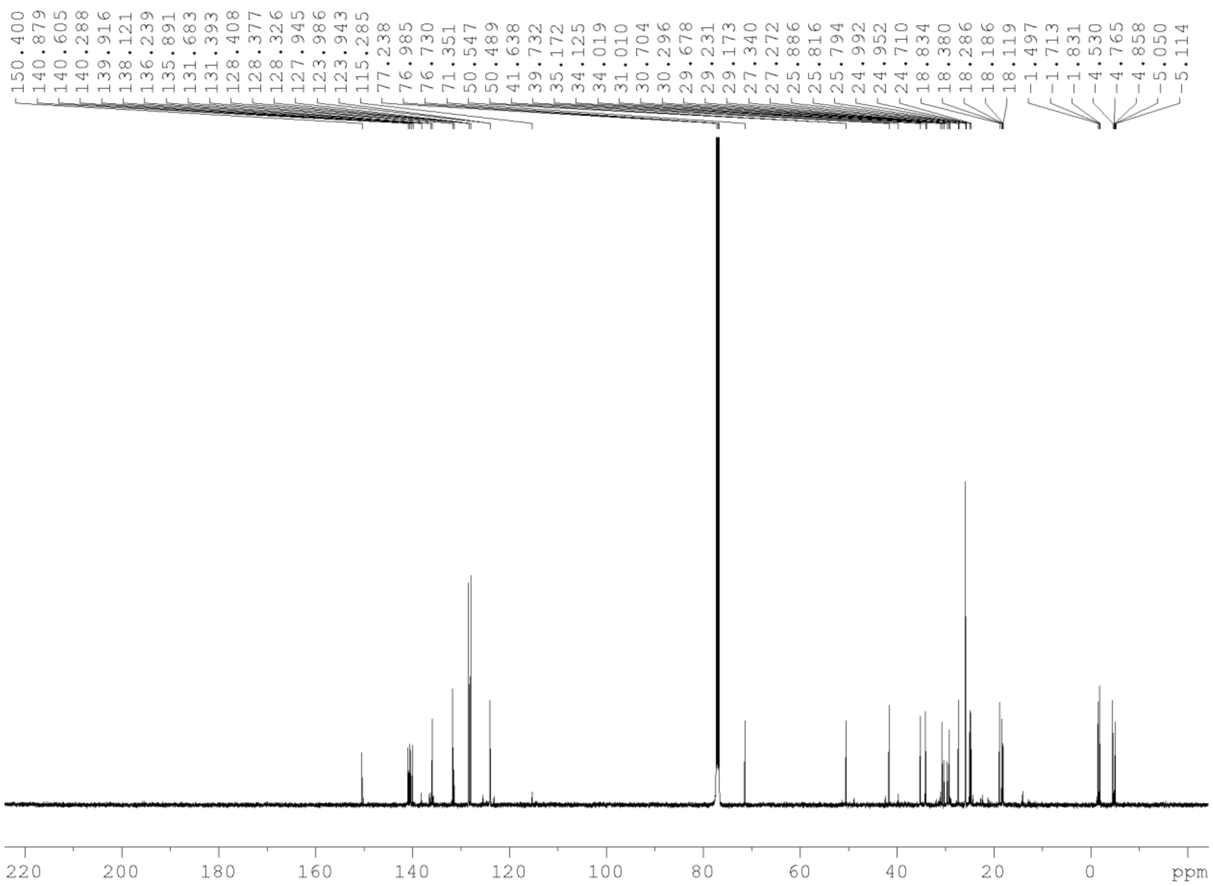

(12*R*,13*R*,*E*)-12-((Benzyldimethylsilyl)ethynyl)-13-methylhexadeca-14-en-1,8-diyn-7-ol, 54

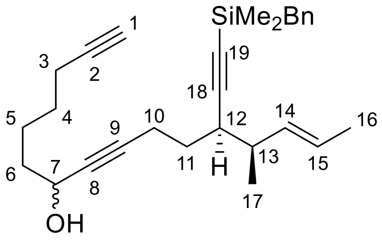

^{1}H (500 MHz, $CDCl_3$)
^{13}C (125 MHz, $CDCl_3$)

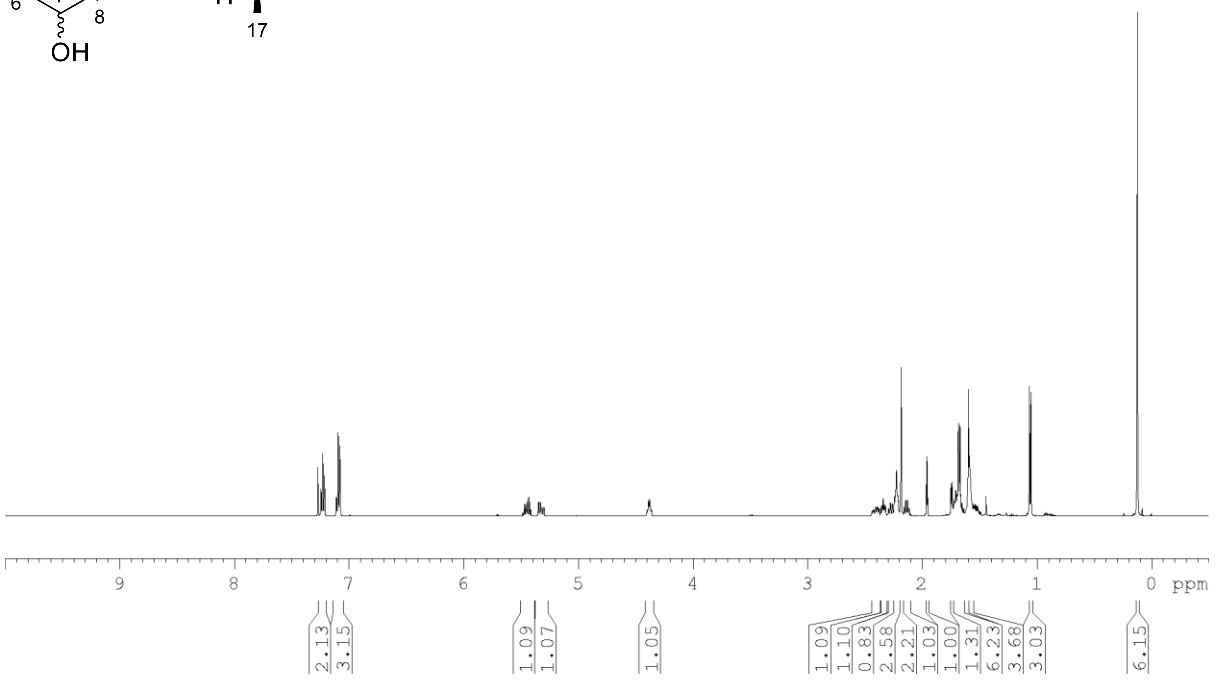

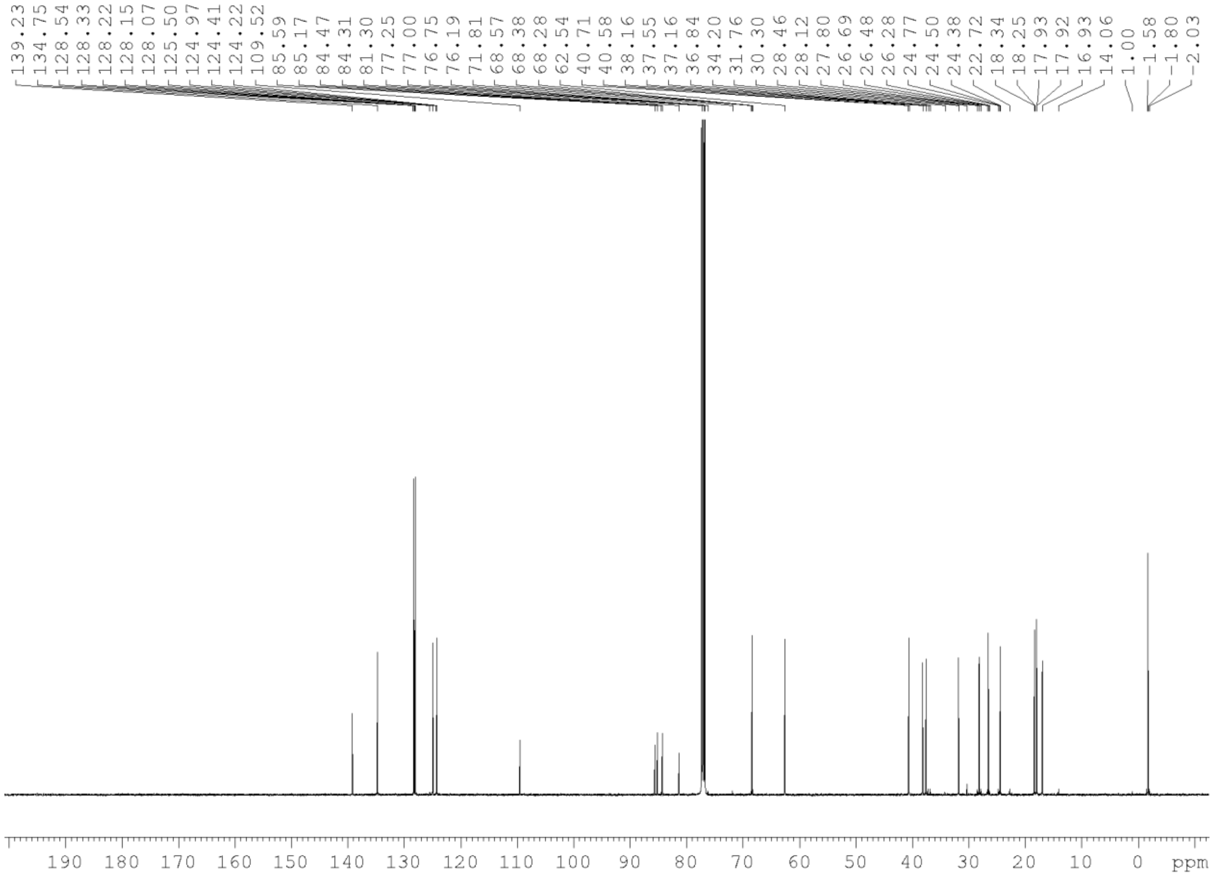

(3*R*)-4-(Benzyldimethylsilyl)-3-((*R*,*E*)-pent-3-en-2-yl)-1,2,3,6,7,8,9,10-octahydrocyclohepta[*e*]inden-10-ol, 56A (unassigned)

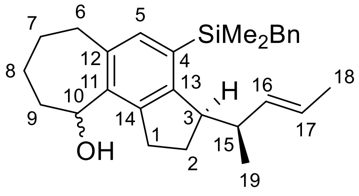

^{1}H (500 MHz, $CDCl_3$)
^{13}C (125 MHz, $CDCl_3$)

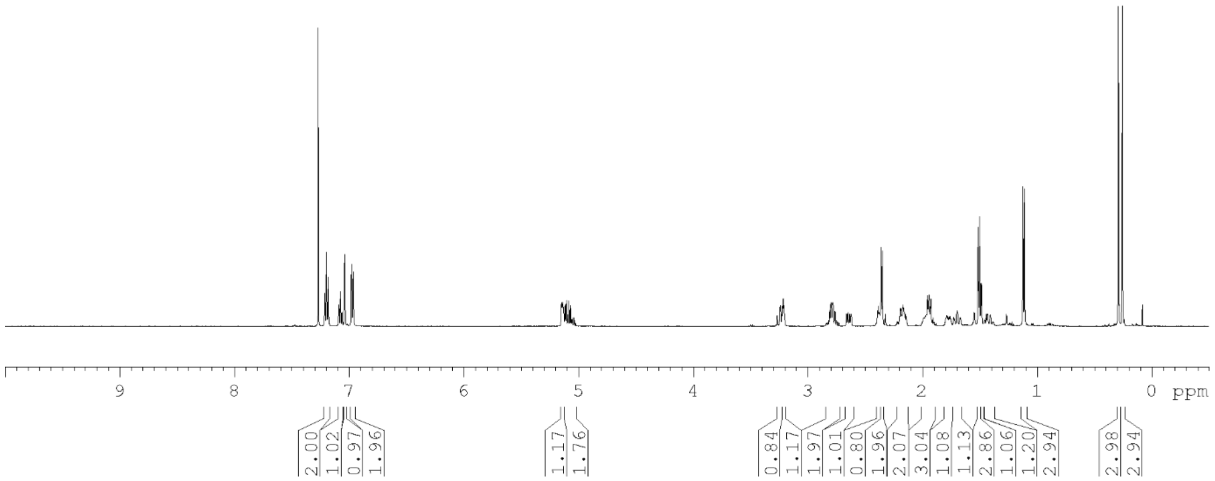

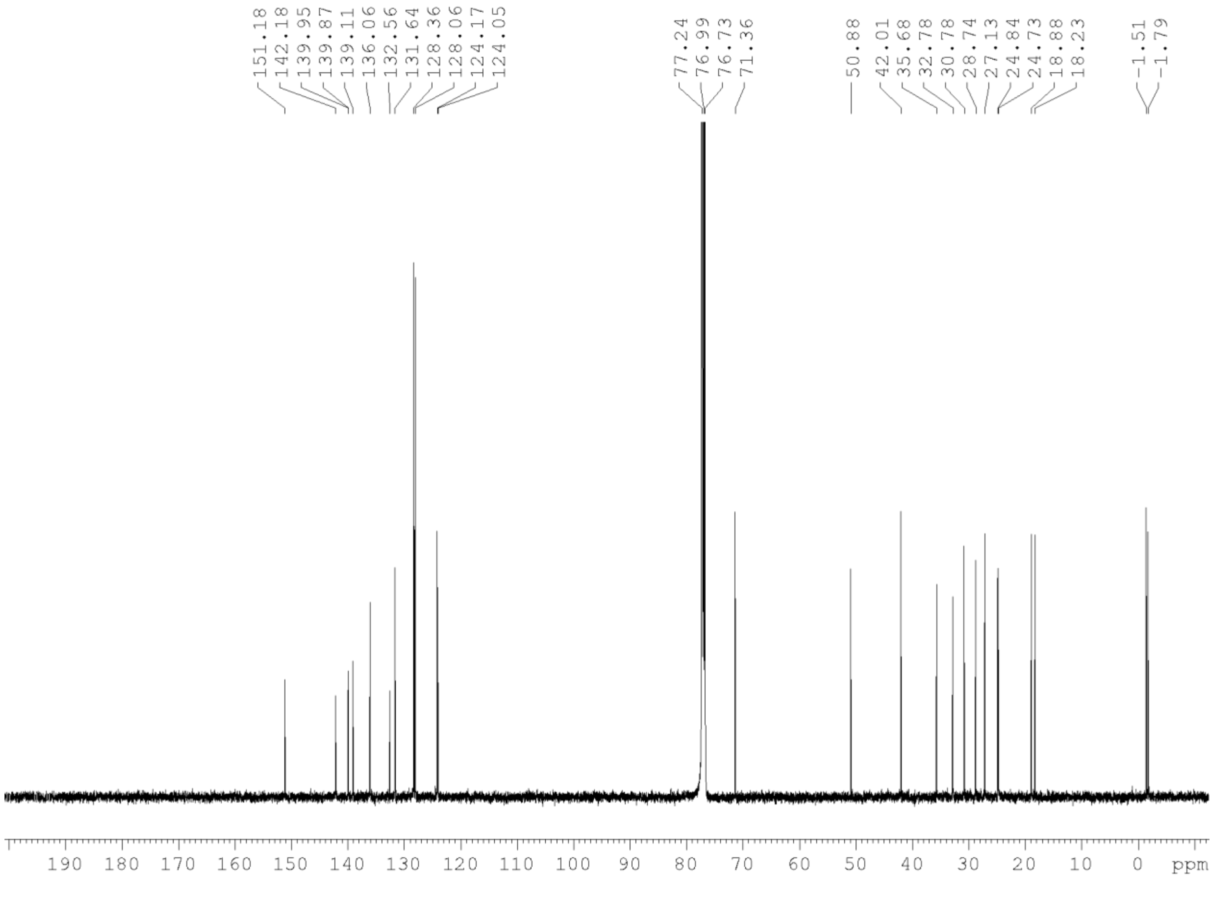

(3*R*)-4-(Benzyldimethylsilyl)-3-((*R*,*E*)-pent-3-en-2-yl)-1,2,3,6,7,8,9,10-octahydrocyclohepta[*e*]inden-10-ol, 56B (unassigned)

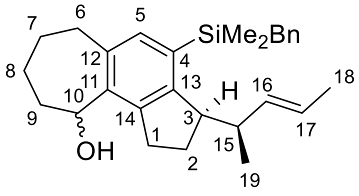

^{1}H (500 MHz, $CDCl_3$)
^{13}C (125 MHz, $CDCl_3$)

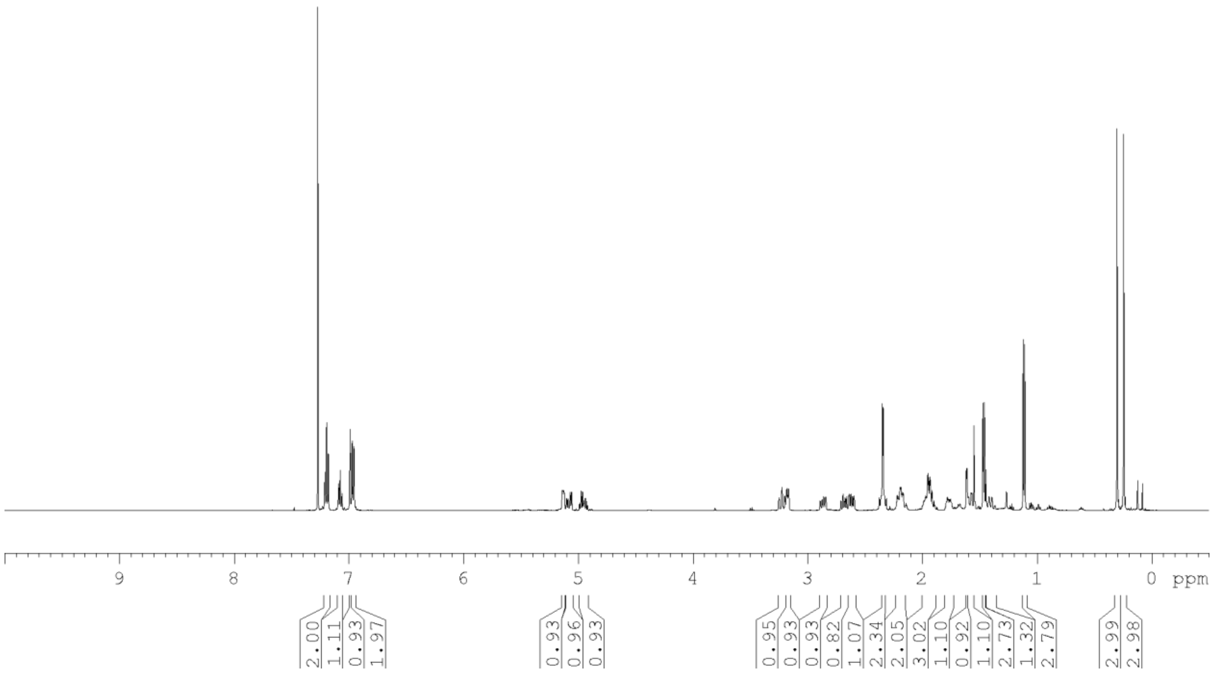

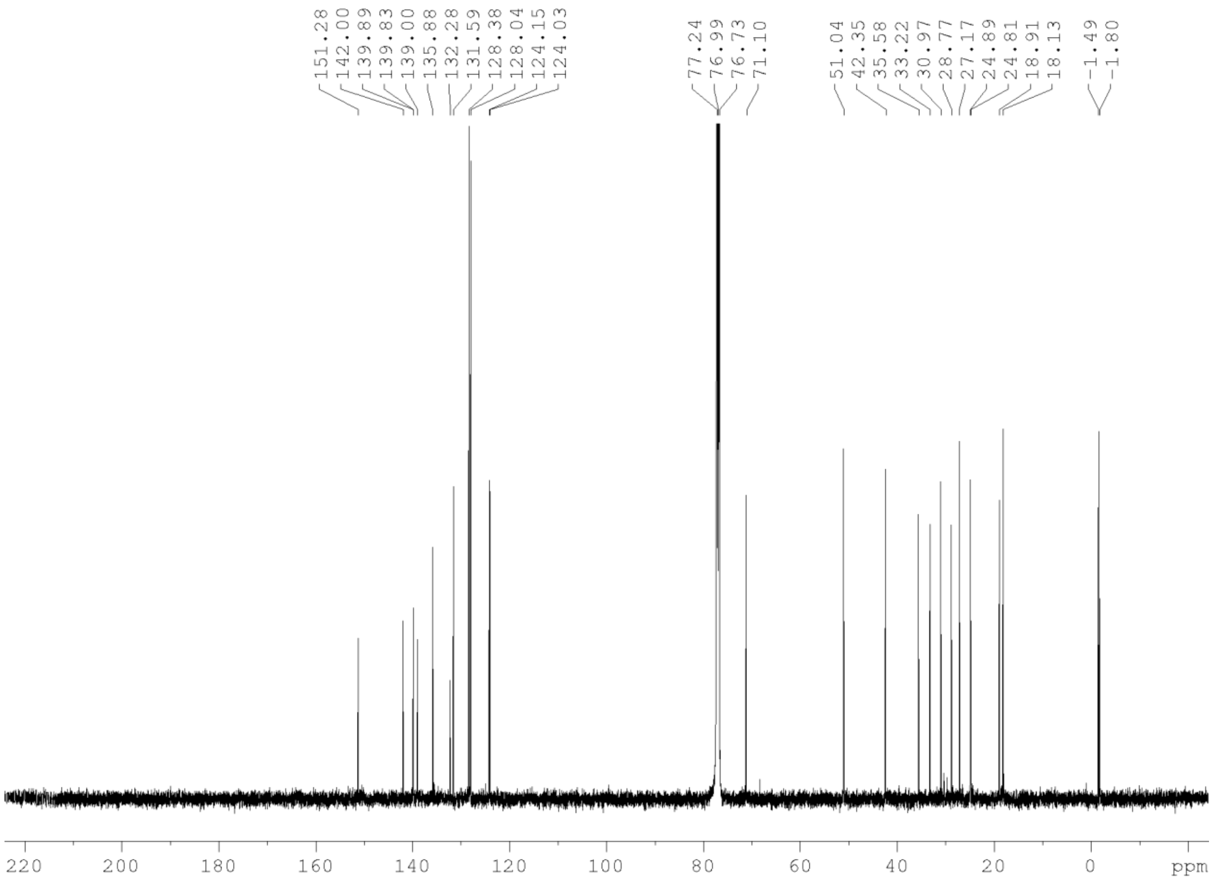

Benzyl((3*R*)-13-bromo-8-((*tert*-butyldimethylsilyl)oxy)-3-((*R*,*E*)-pent-3-en-2-yl)tetradeca-13-en-1,6-diyn-1-yl)dimethylsilane, 57

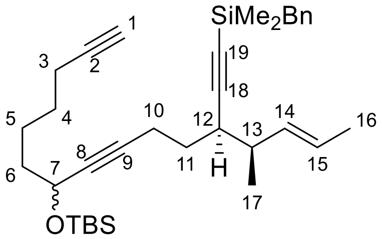

^{1}H (500 MHz, $CDCl_3$)
^{13}C (125 MHz, $CDCl_3$)

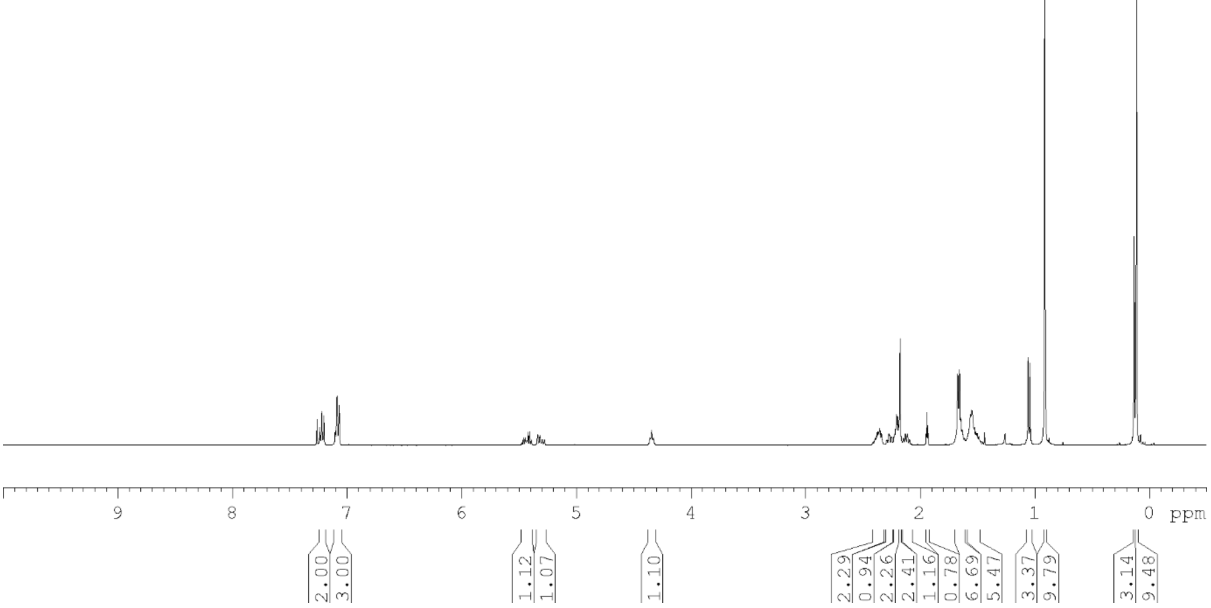

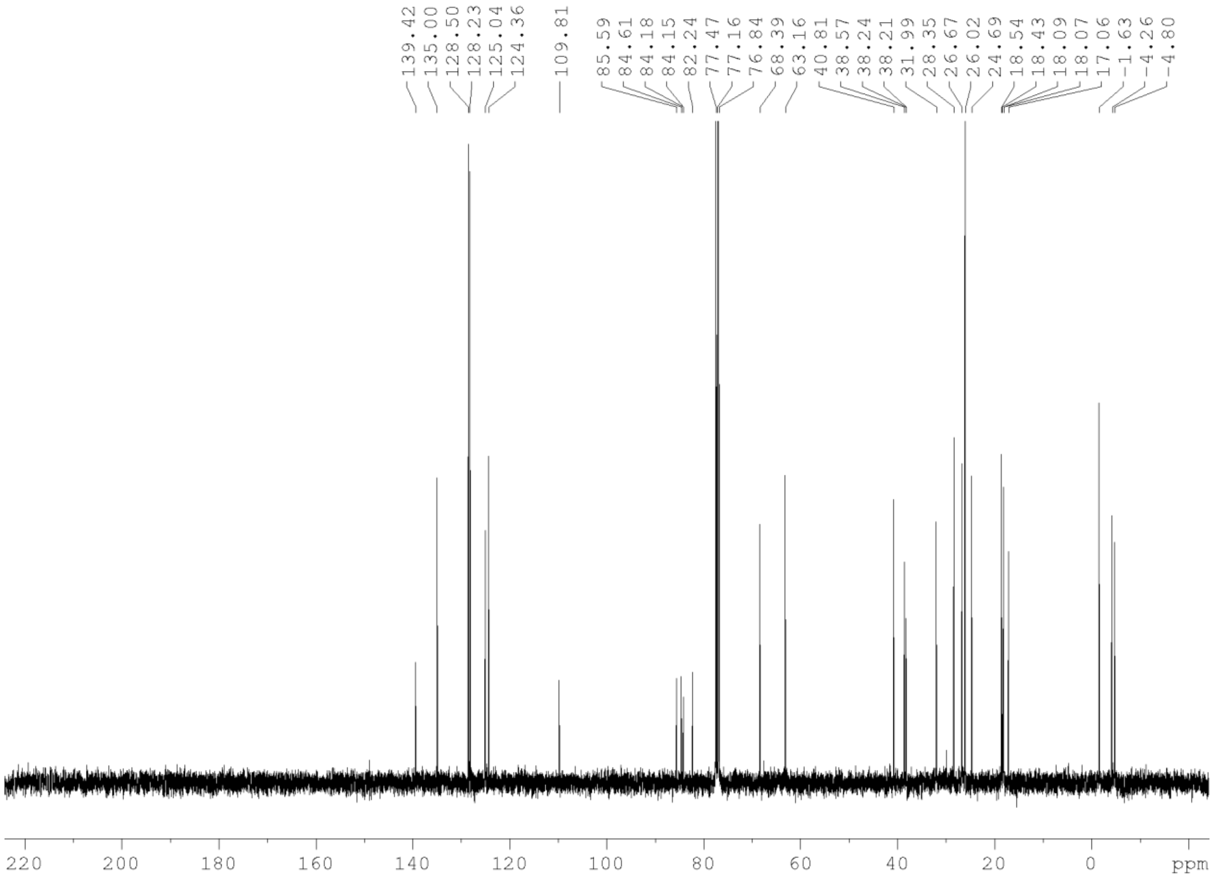

(3*R*)-10-((*tert*-Butyldimethylsilyl)oxy)-3-((*R*,*E*)-pent-3-en-2-yl)-1,2,3,6,7,8,9,10-octahydrocyclohepta[*e*]inden-4-ol, 58

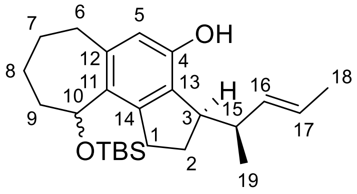

^{1}H (500 MHz, $CDCl_3$)
^{13}C (125 MHz, $CDCl_3$)

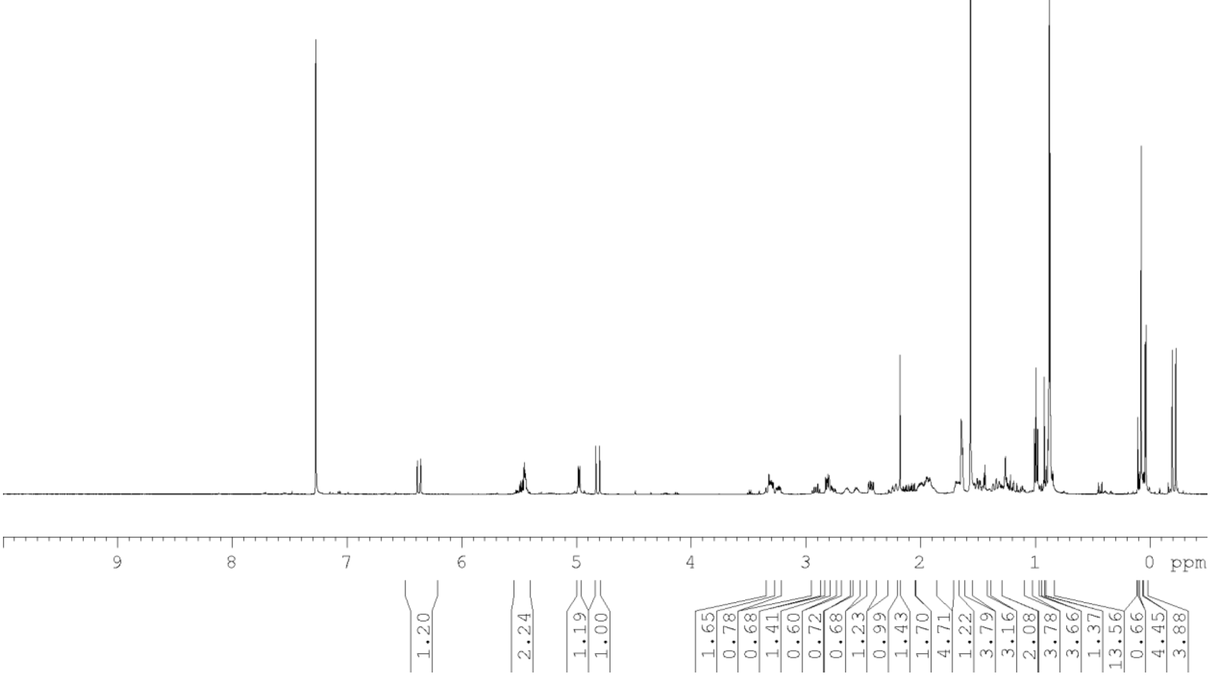

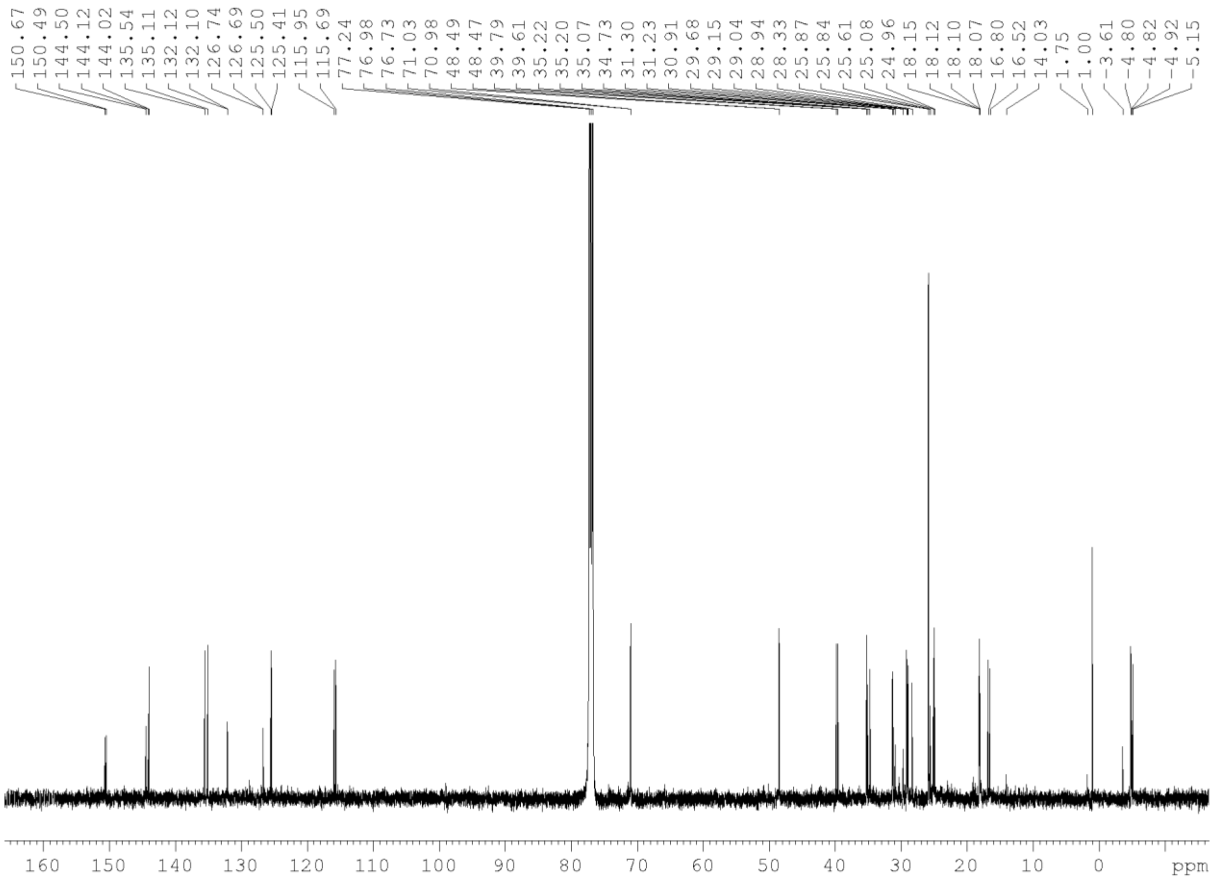

(3*R*)-3-((*R*,*E*)-Pent-3-en-2-yl)-1,2,3,6,7,8,9,10-octahydrocyclohepta[*e*]indene-4,10-diol, 59A (unassigned)

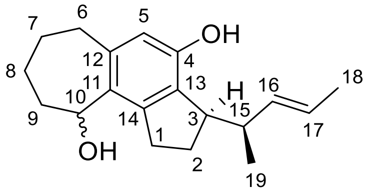

^{1}H (400 MHz, $CDCl_3$)
^{13}C (101 MHz, $CDCl_3$)

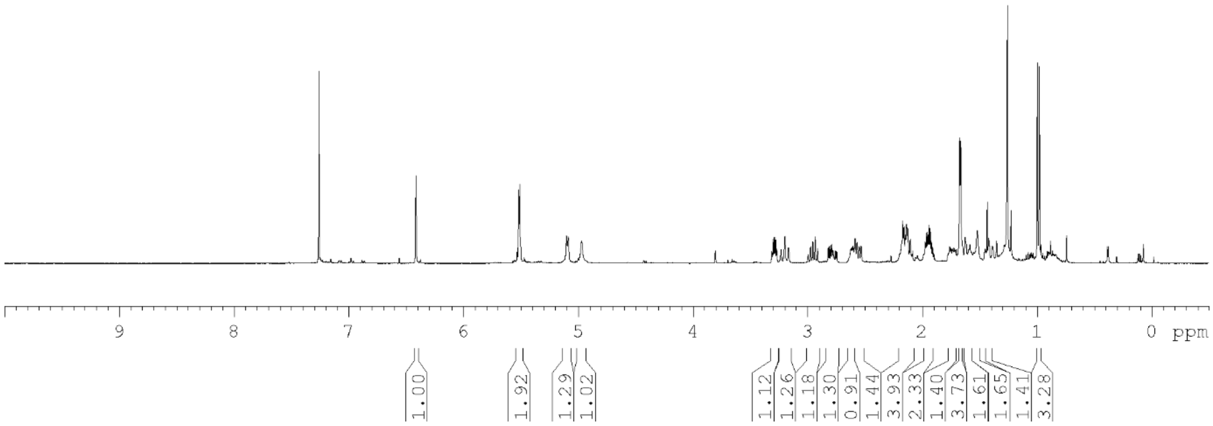

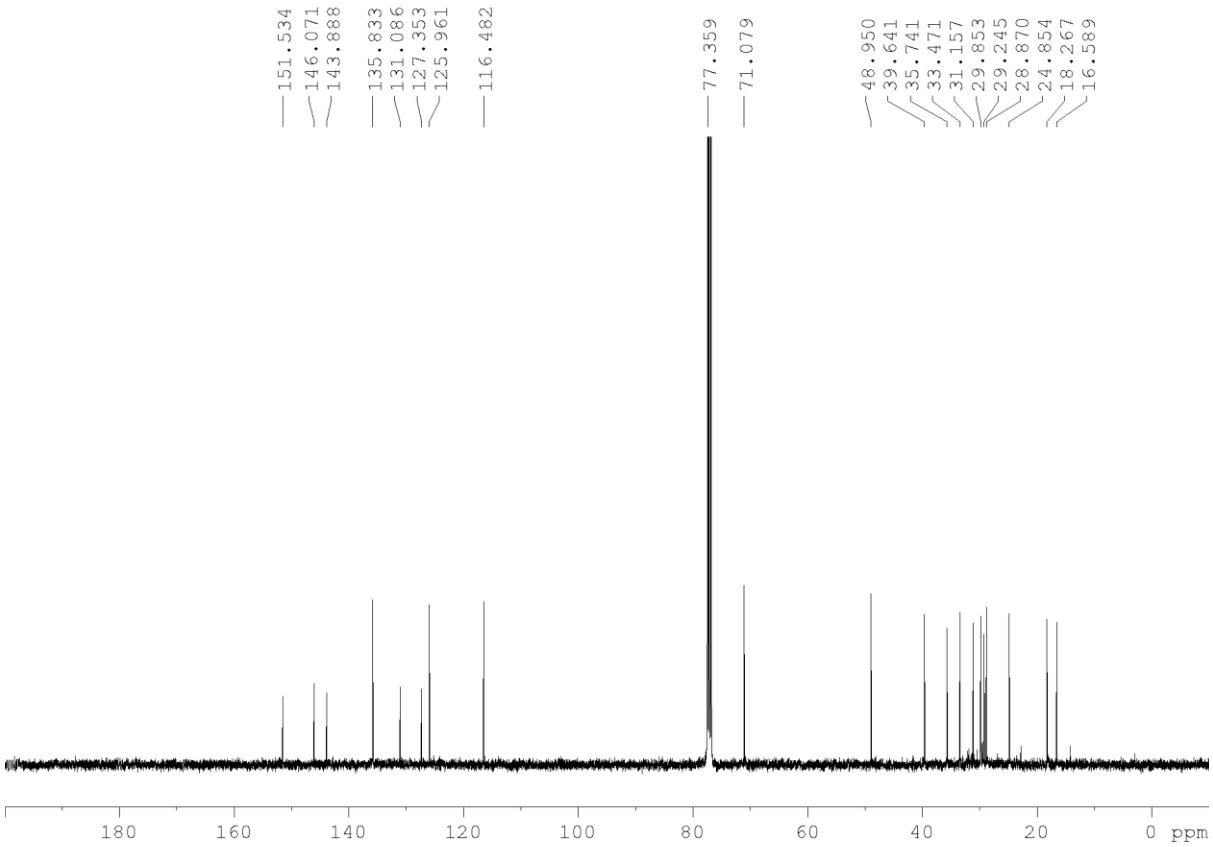

(3*R*)-3-((*R*,*E*)-Pent-3-en-2-yl)-1,2,3,6,7,8,9,10-octahydrocyclohepta[*e*]indene-4,10-diol, 59B (unassigned)

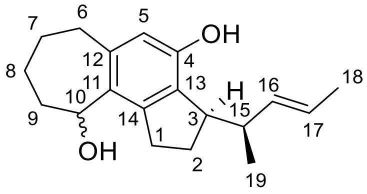

^{1}H (400 MHz, $CDCl_3$)
^{13}C (101 MHz, $CDCl_3$)

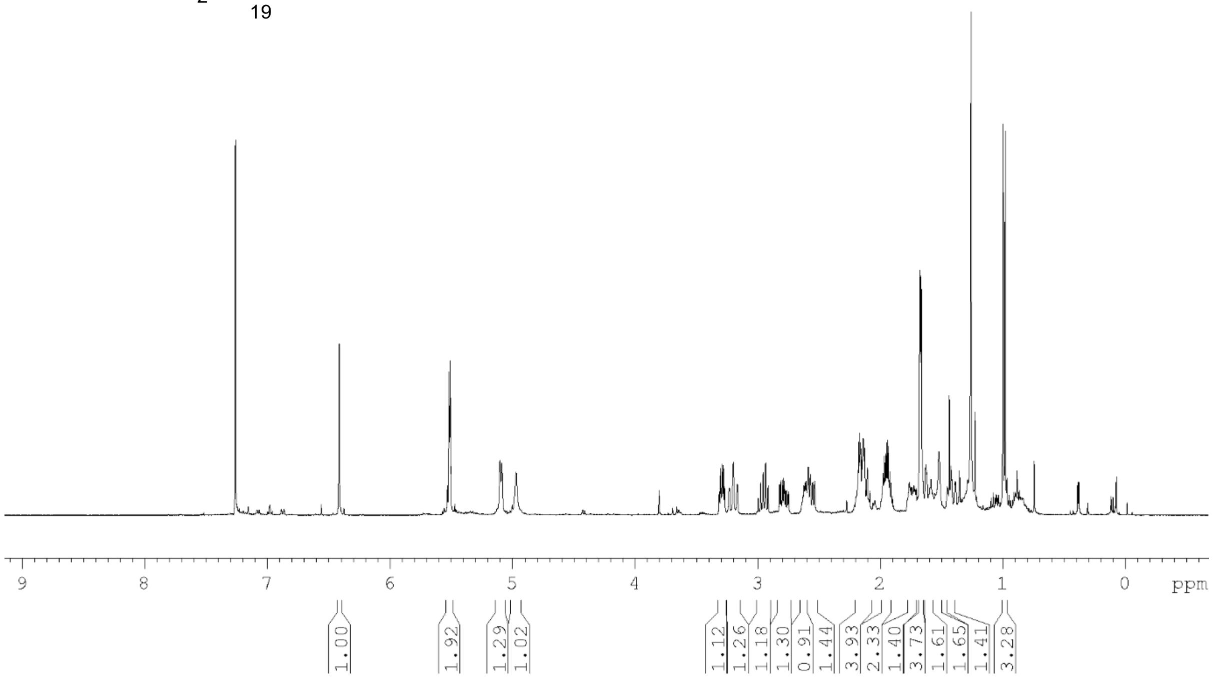

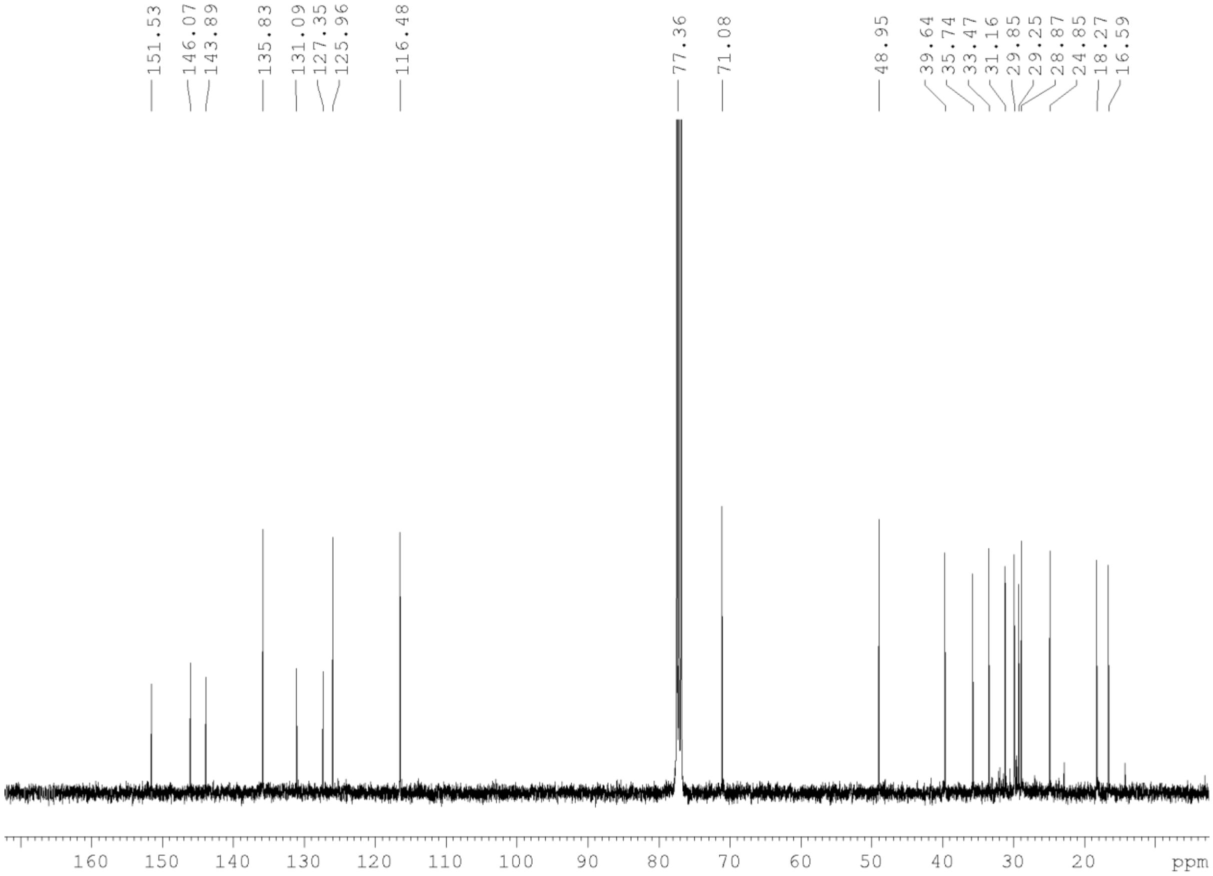

(*R*)-3-((*R*,*E*)-Pent-3-en-2-yl)-1,2,3,6,7,8,9,10-octahydrocyclohepta[e]inden-4-ol, 61

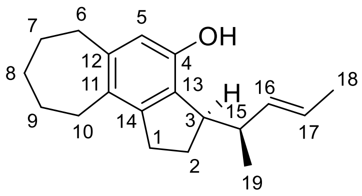

^{1}H (500 MHz, $CDCl_3$)

^{13}C (125 MHz, $CDCl_3$)

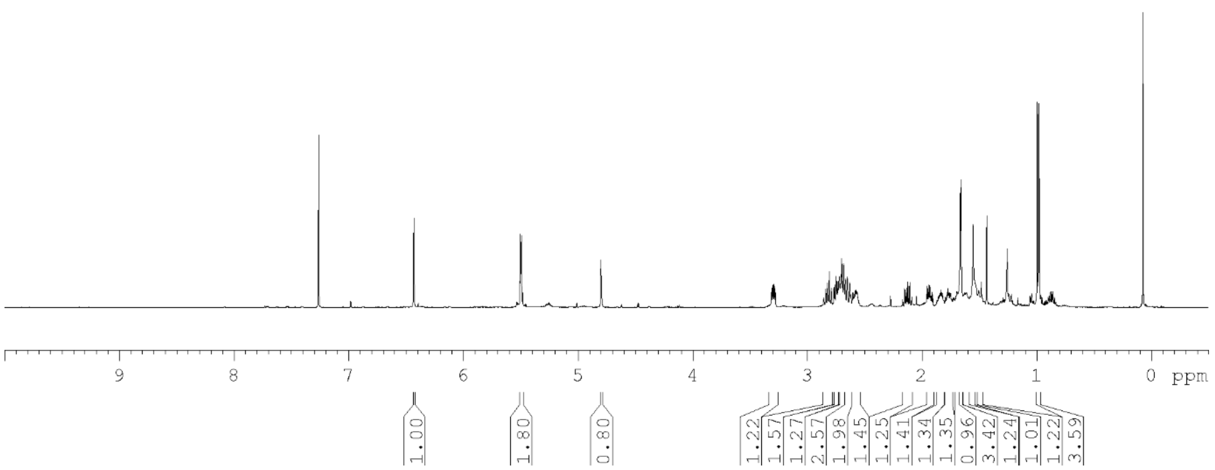

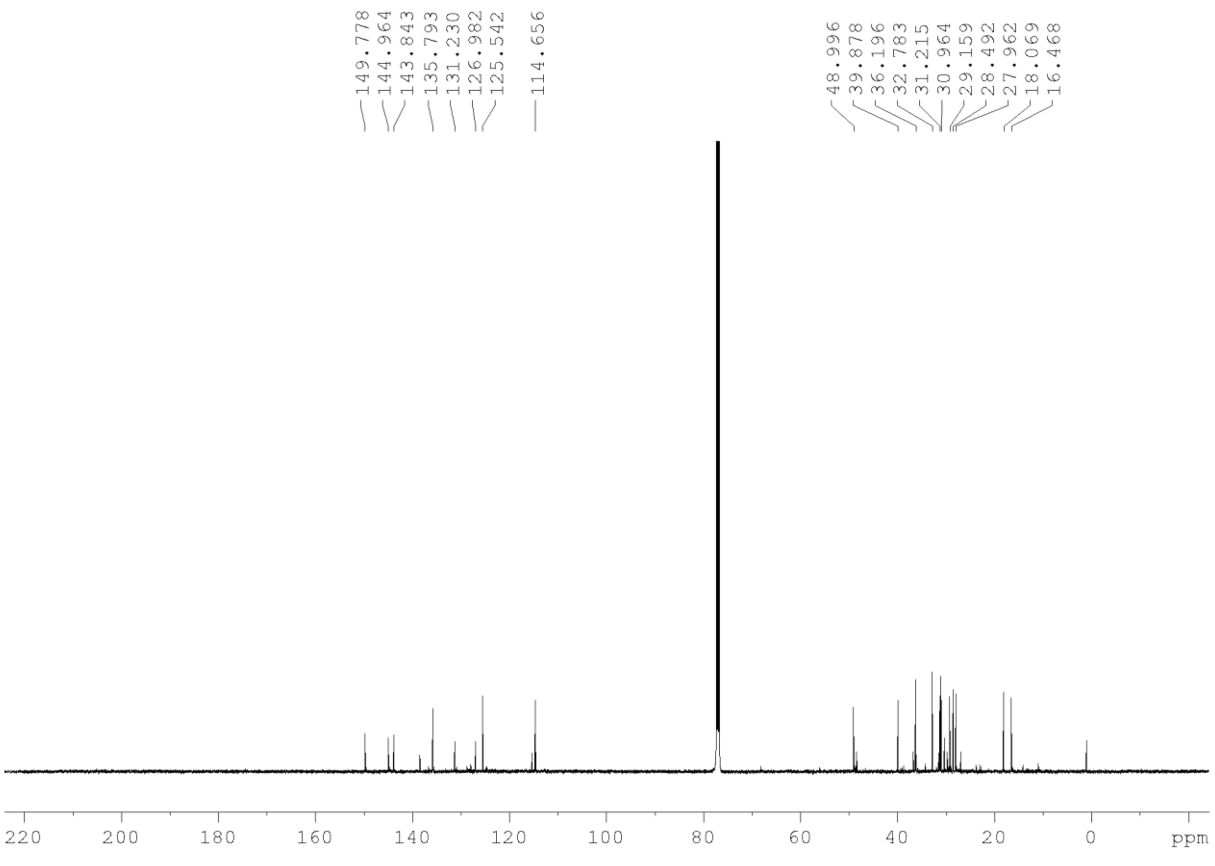

Triethyl(((*R*)-3-((*R*,*E*)-pent-3-en-2-yl)-1,2,3,6,7,8,9,10-octahydrocyclohepta[*e*]inden-4-yl)oxy)silane, S7

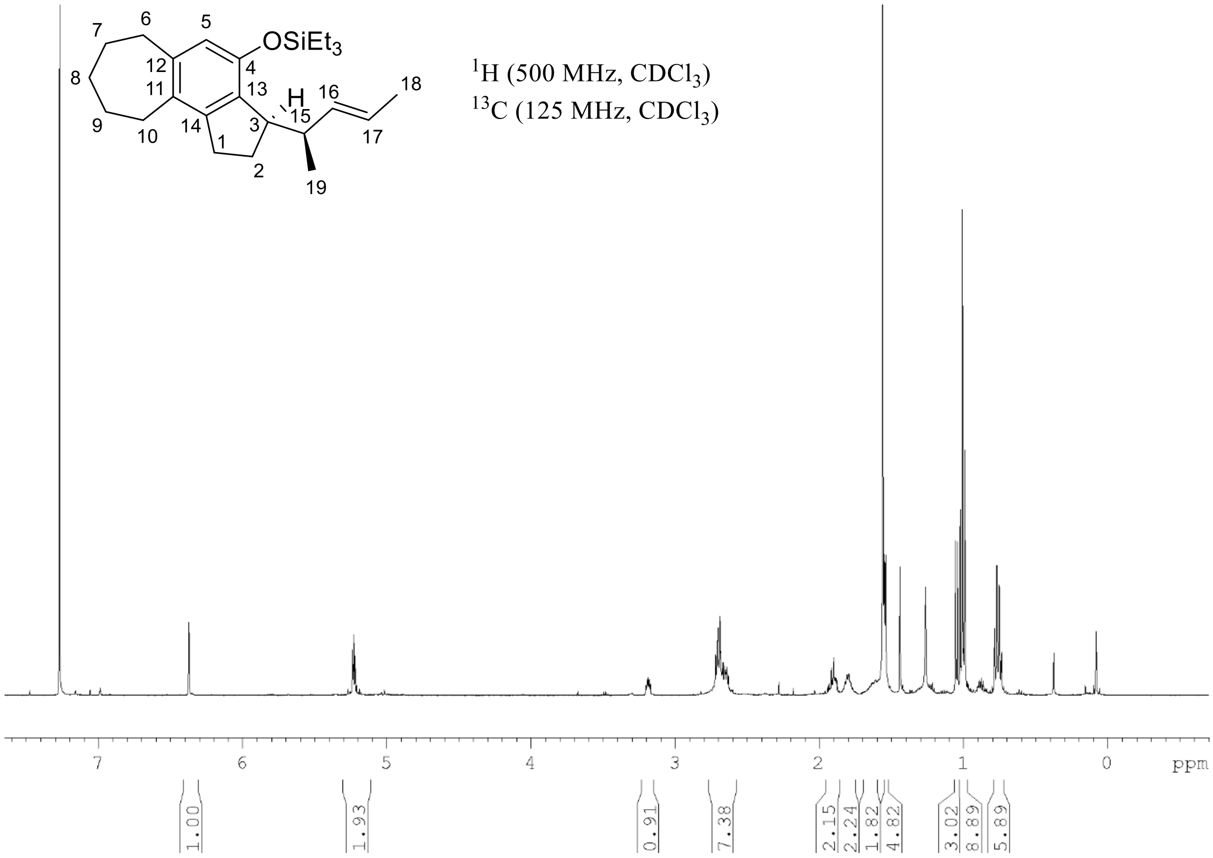

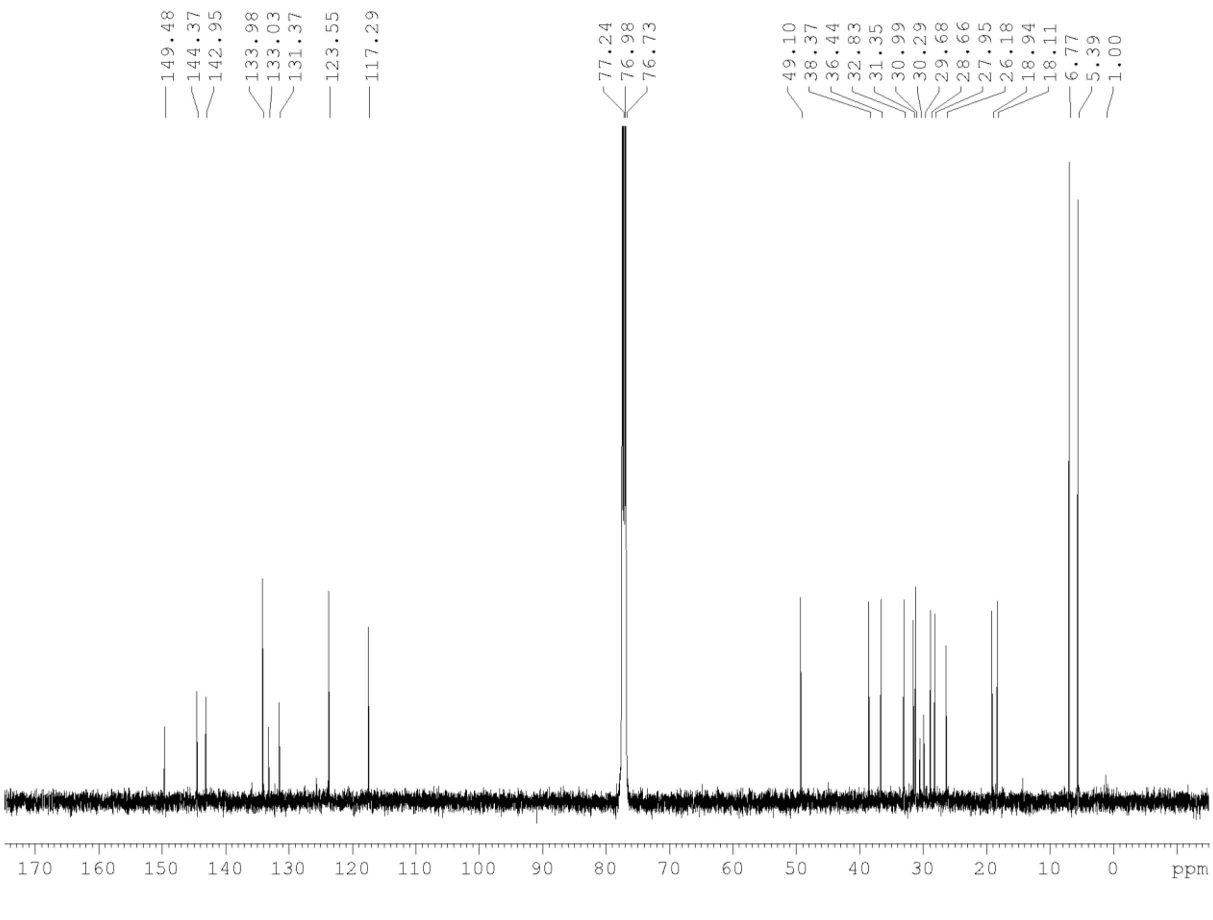

(2*R*,3*R*,4*S*)-4-((*R*)-4-hydroxy-1,2,3,6,7,8,9,10-octahydrocyclohepta[*e*]inden-3-yl)pentane-2,3-diol, S8 (unassigned)

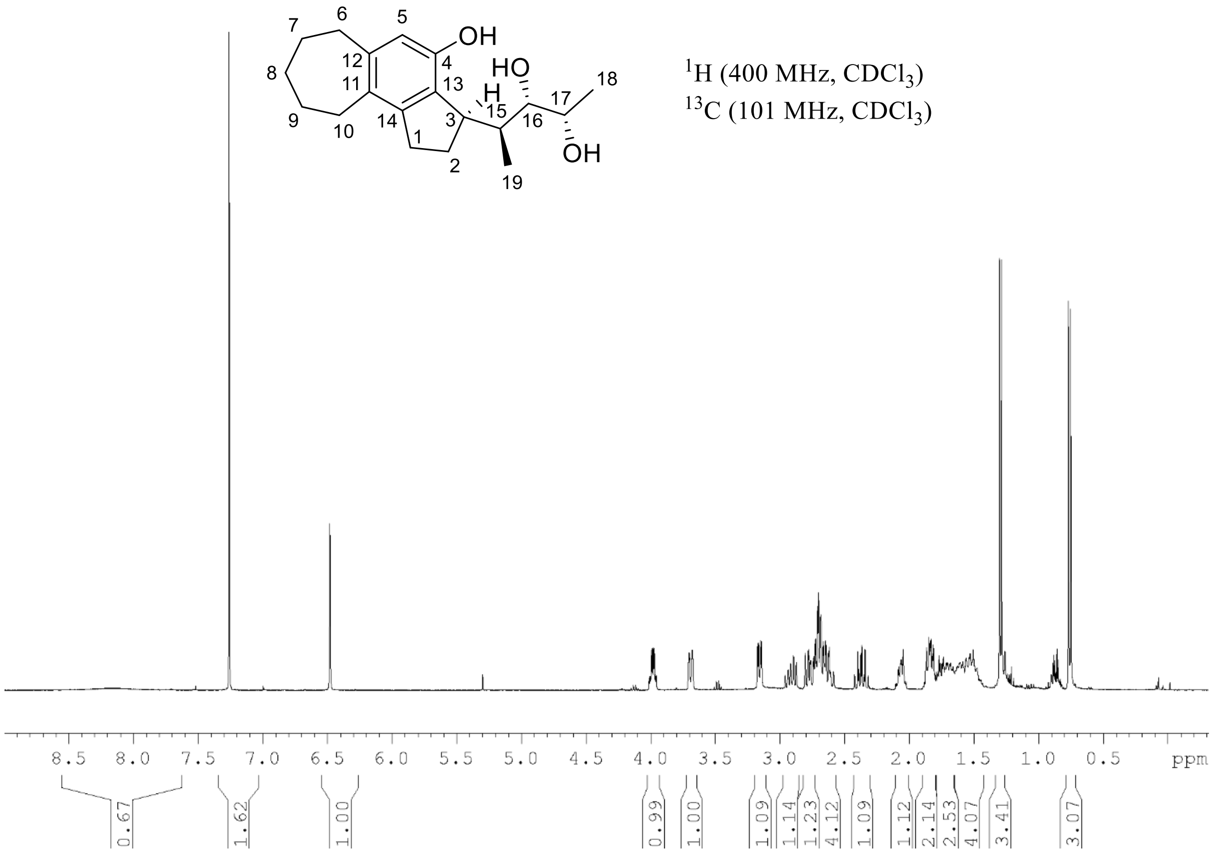

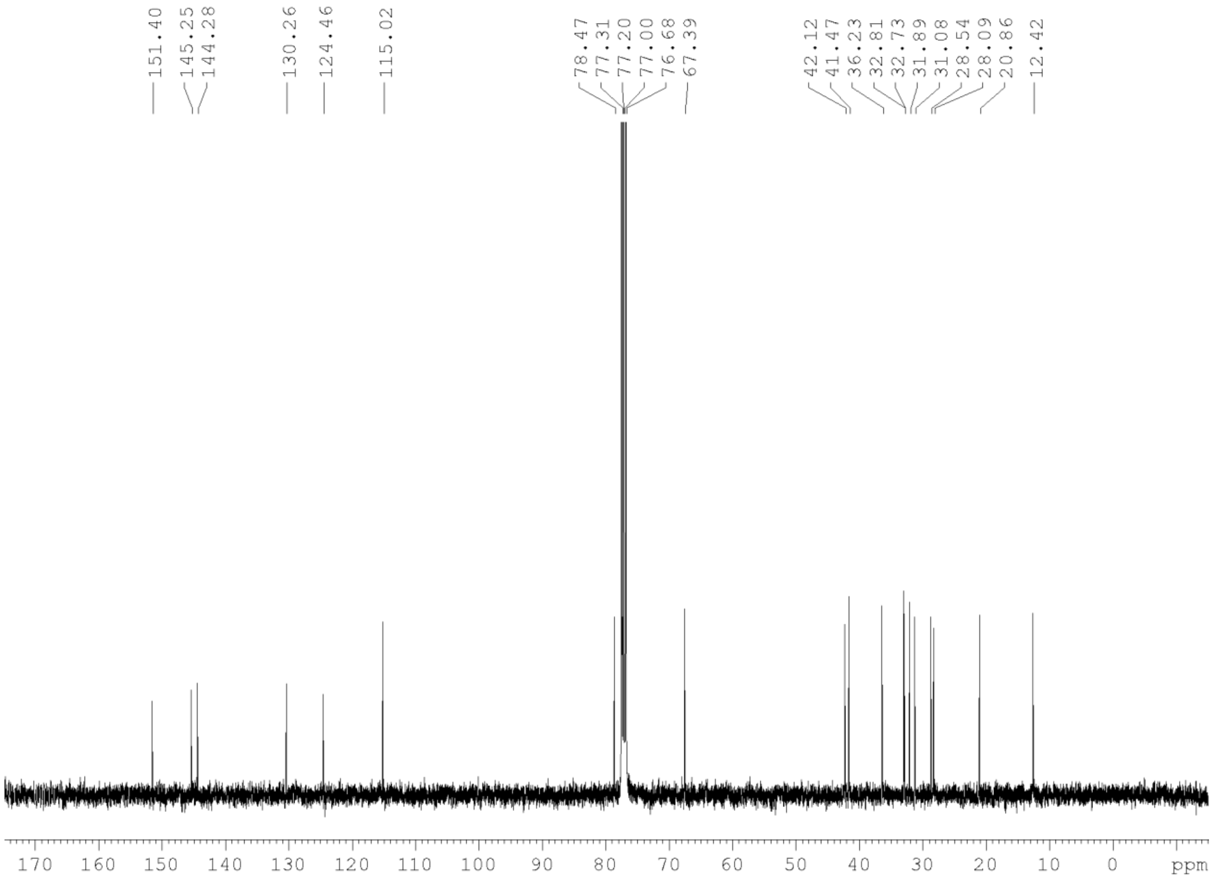

(2*R*,3*R*,4*S*)-4-((*R*)-4-hydroxy-1,2,3,6,7,8,9,10-octahydrocyclohepta[*e*]inden-3-yl)pentane-2,3-diol, S9 (unassigned)

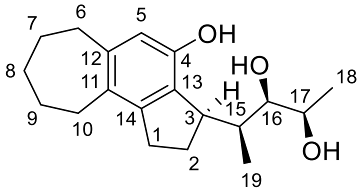

^{1}H (400 MHz, $CDCl_3$)
^{13}C (101 MHz, $CDCl_3$)

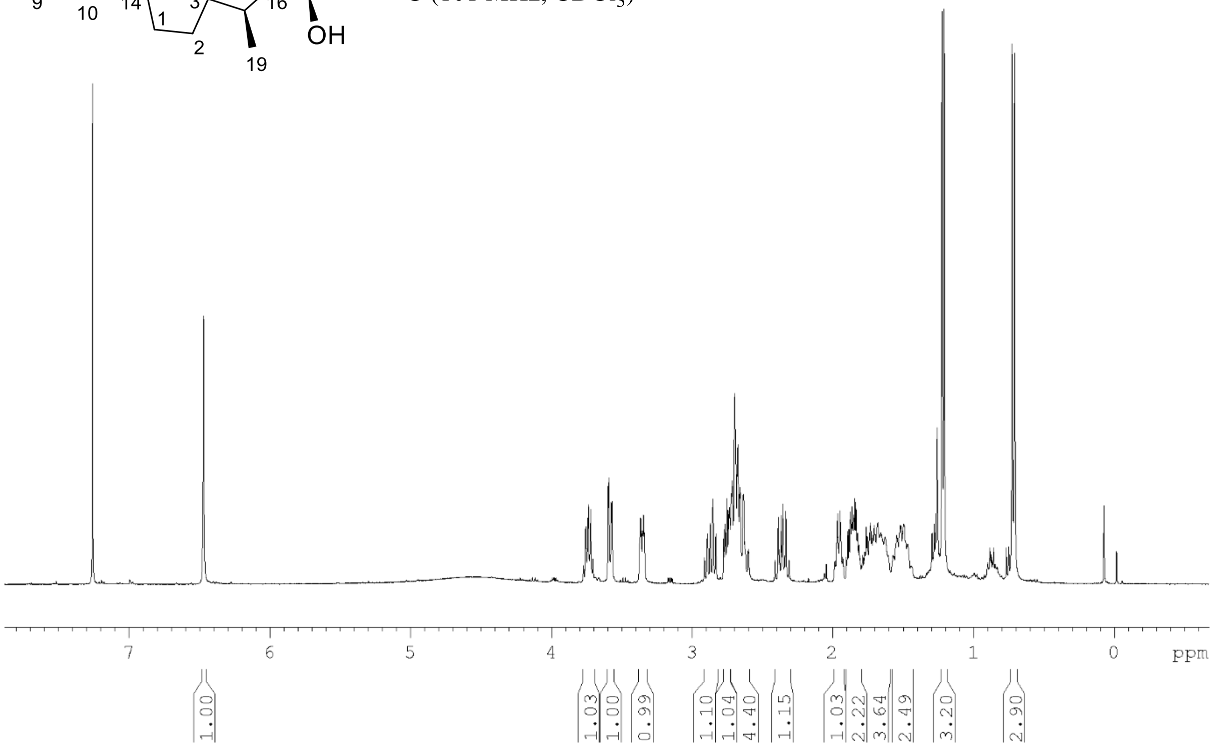

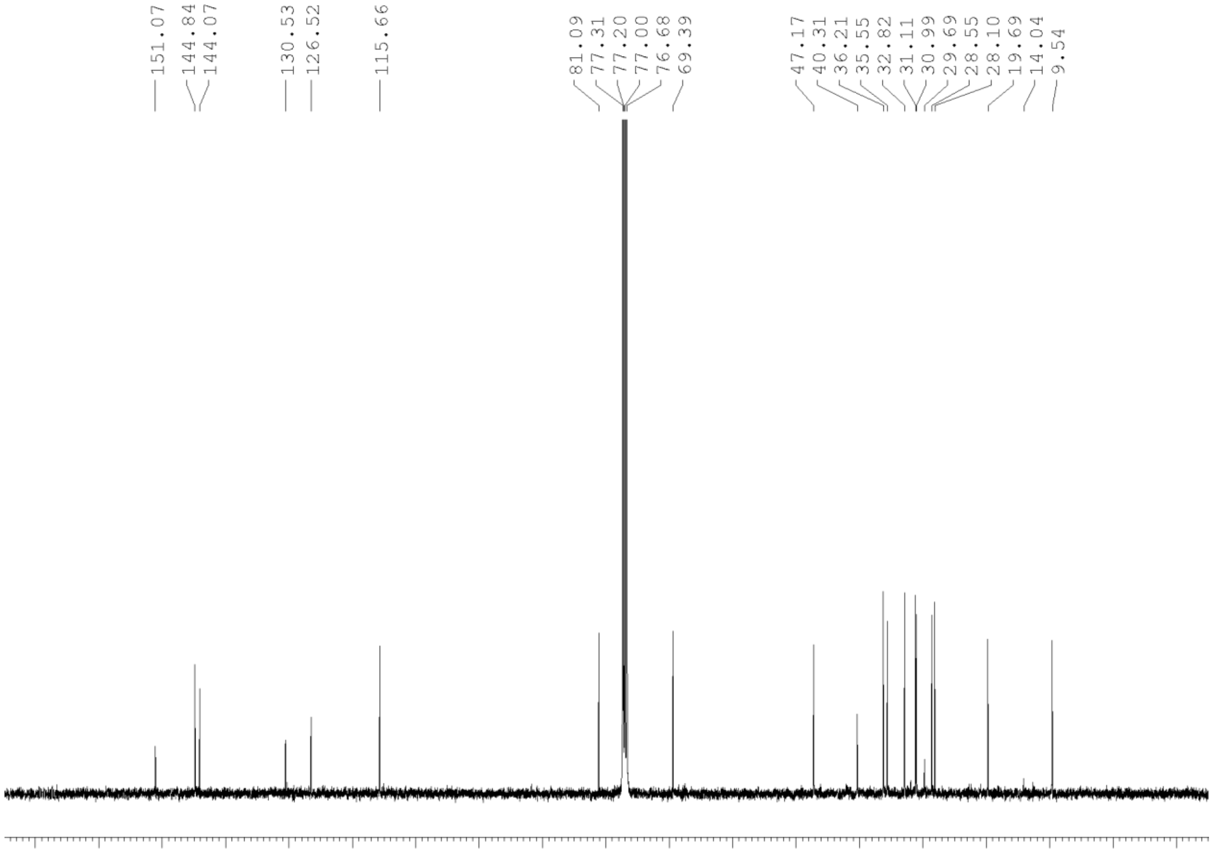

(3*R*,3'*R*)-3,3'-di((*R*,*E*)-pent-3-en-2-yl)-1,1',2,2',3,3',6,6',7,7',8,8',9,9',10,10'-hexadecahydro-[5,5'-bi(cyclohepta[*e*]indene)]-4,4'-diol, 62

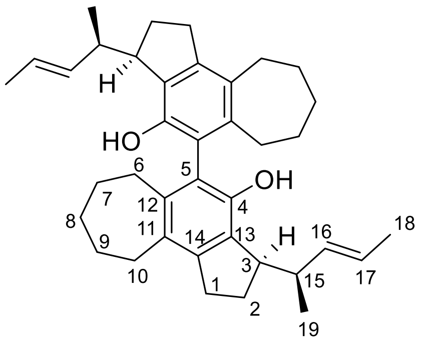

^{1}H (500 MHz, $CDCl_3$)
^{13}C (125 MHz, $CDCl_3$)

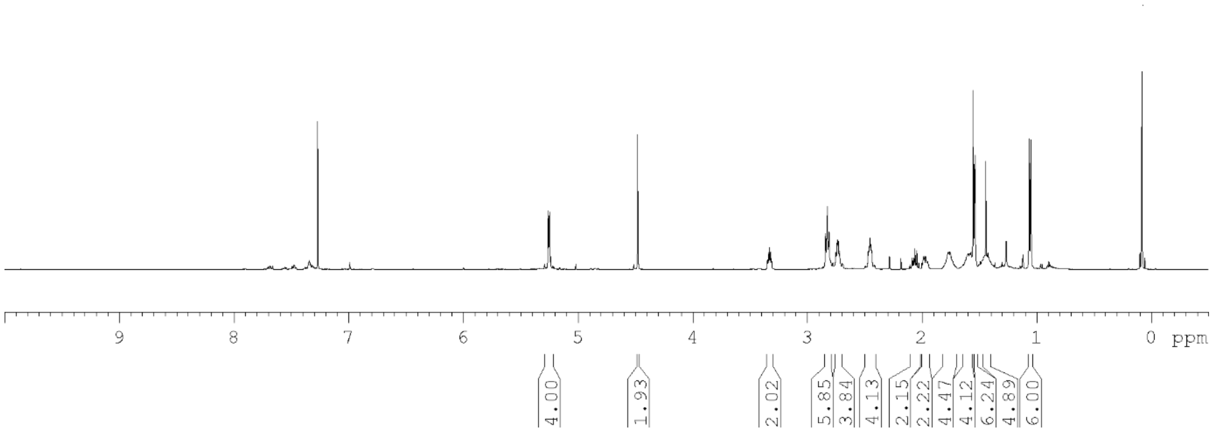

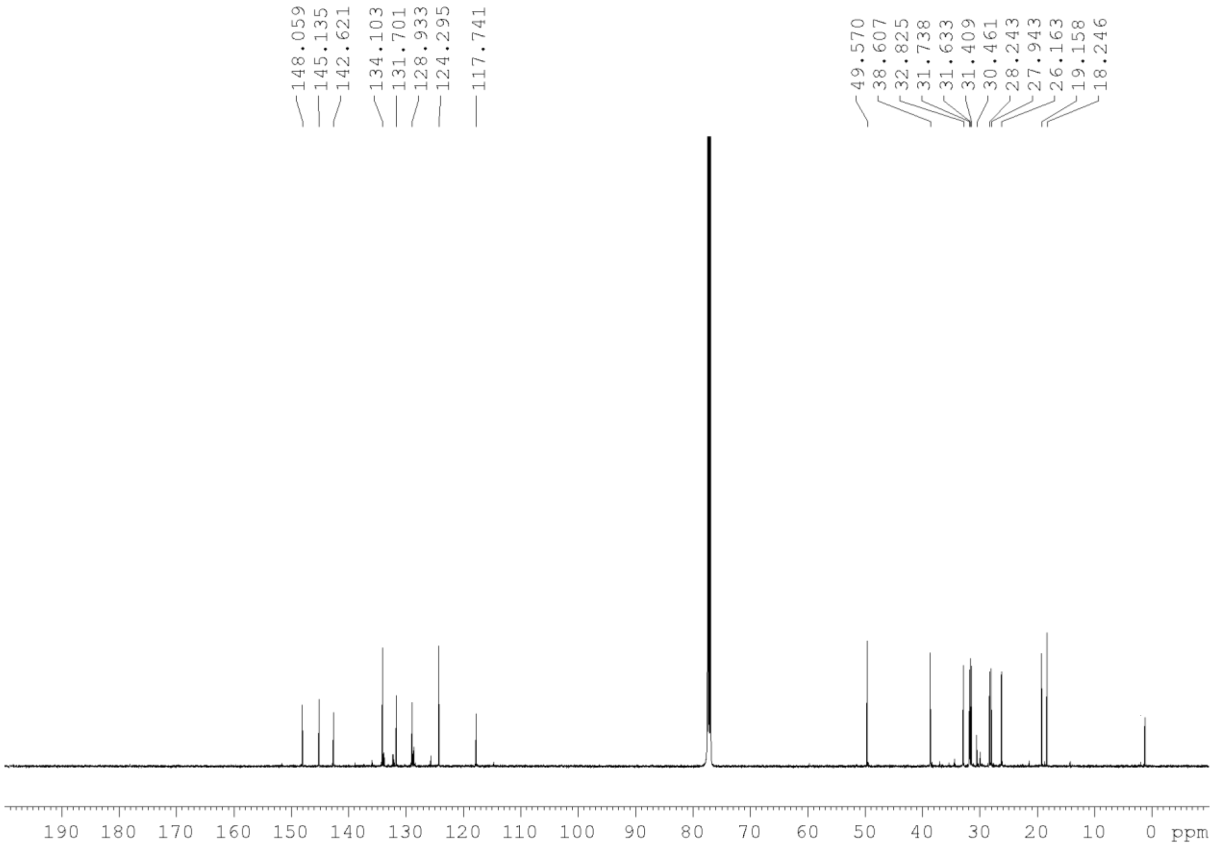

Equilibrium mixture of aldehyde (S)-2-((R)-4-hydroxy-1,2,3,6,7,8,9,10-octahydrocyclohepta[e]inden-3-yl)propanal, 63 and lactols (2a*R*,3*S*)-3-Methyl-1,2,2a,3,4,7,8,9,10,11-decahydrocyclohepta[*g*]cyclopenta[*de*]chromen-4-ol, 64

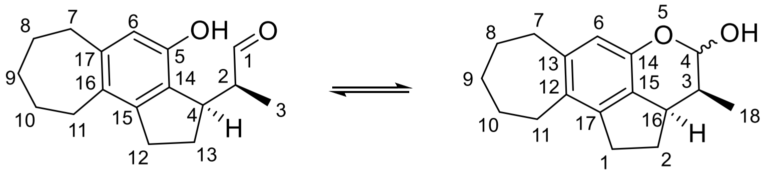

^{1}H (500 MHz, $CDCl_3$)

^{13}C (125 MHz, $CDCl_3$)

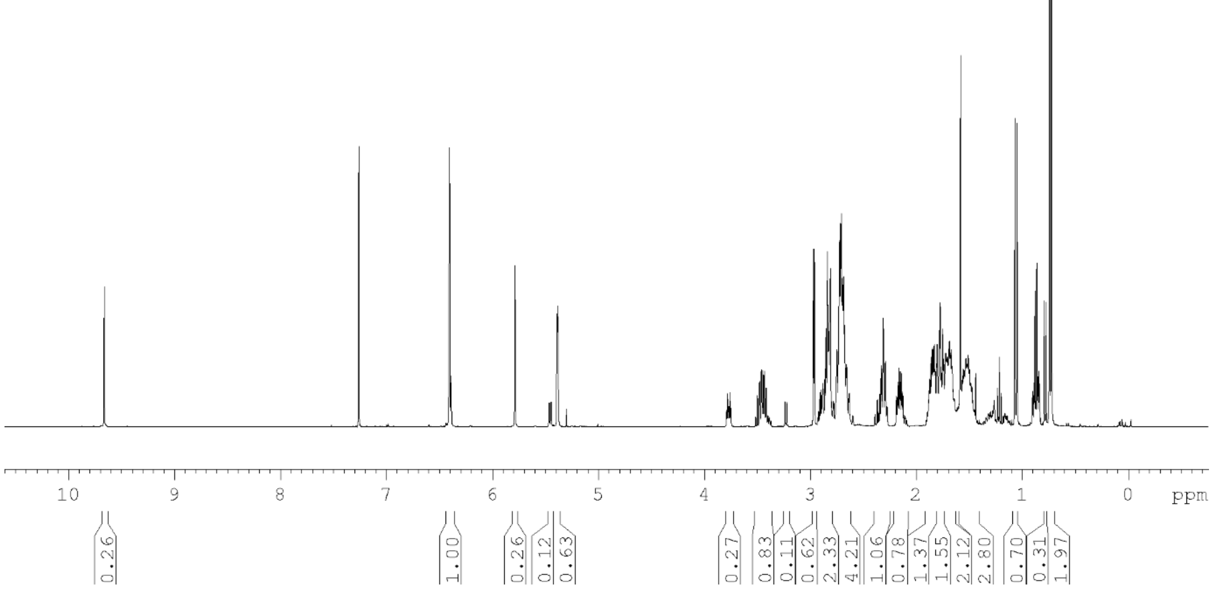

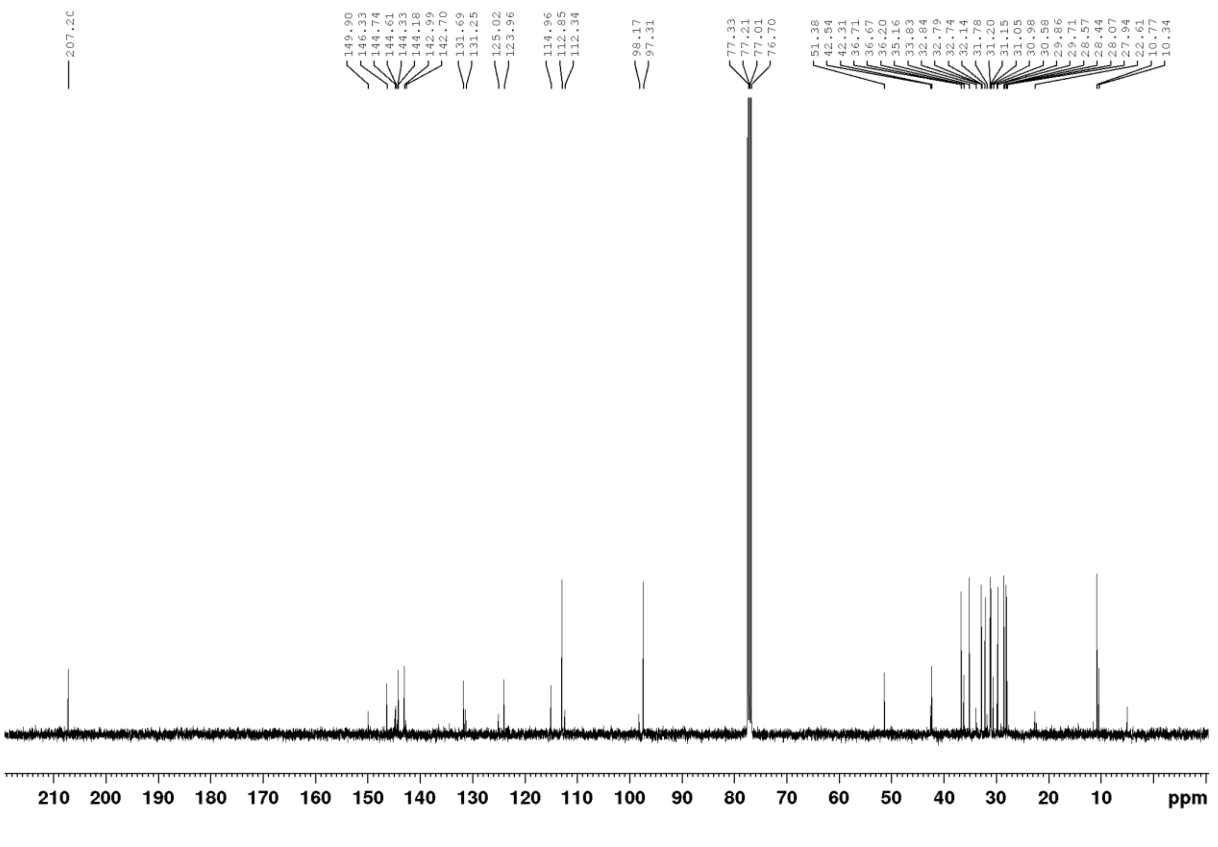

Methyl 2-(7-hydroxy-2,3-dihydro-*1H*-inden-1-yl)acetate, S10

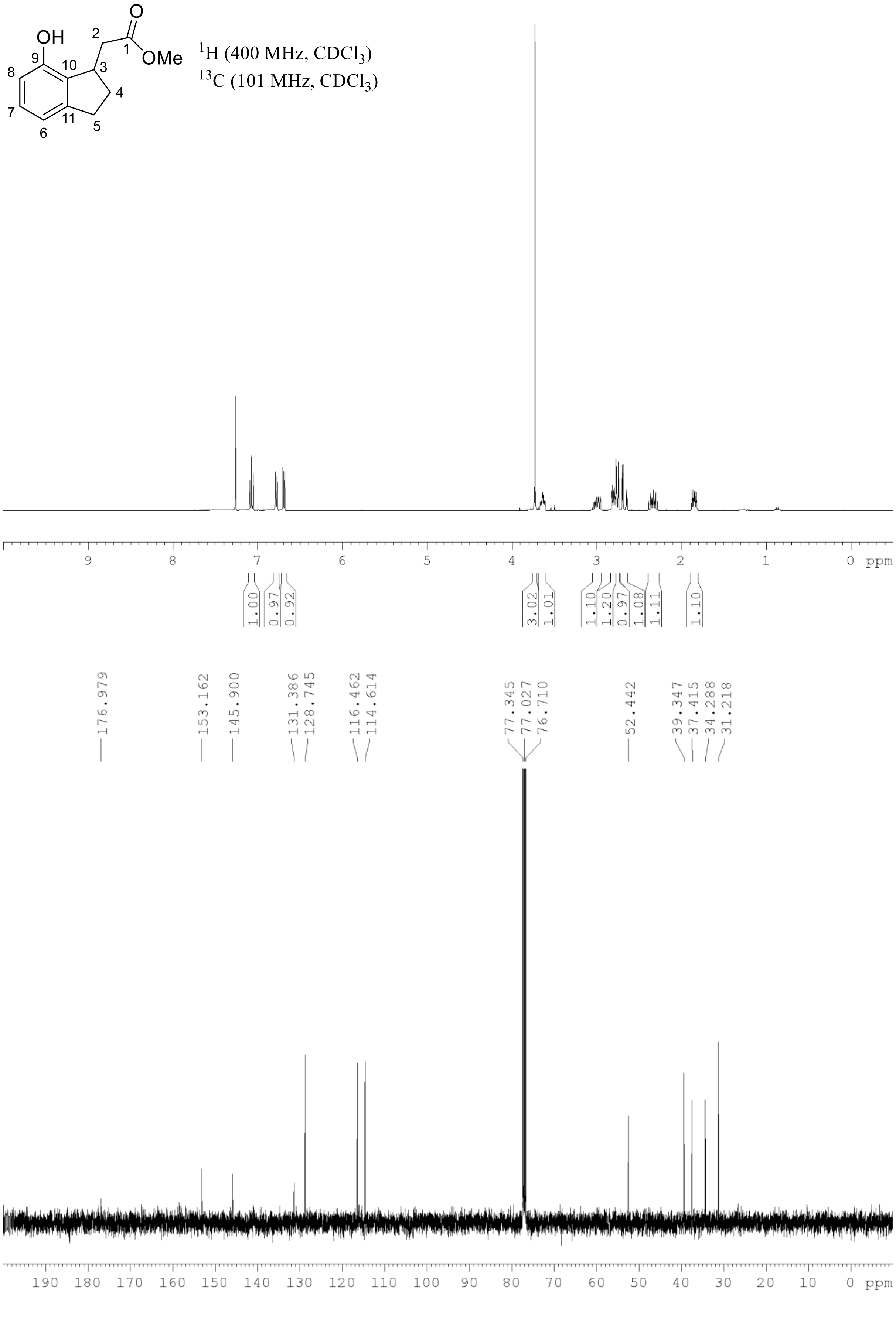

Methyl 2-(7-((triethylsilyl)oxy)-2,3-dihydro-*1H*-inden-1-yl)acetate, S11

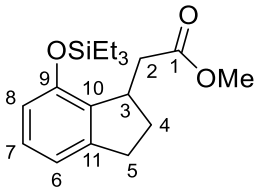

^{1}H (400 MHz, $CDCl_3$)
^{13}C (101 MHz, $CDCl_3$)

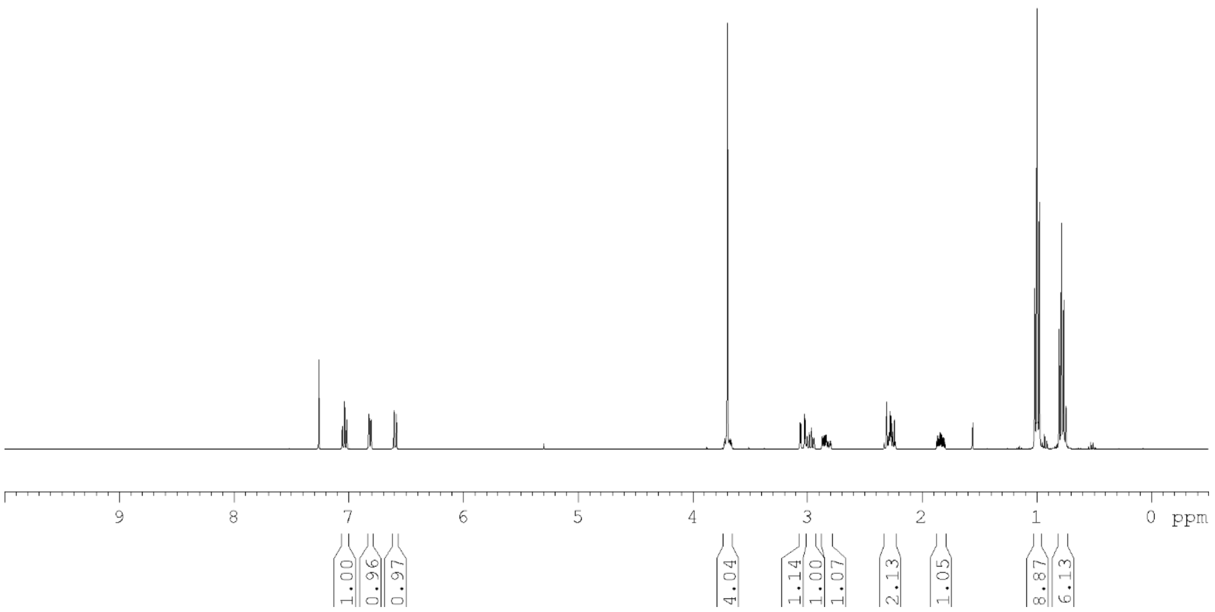

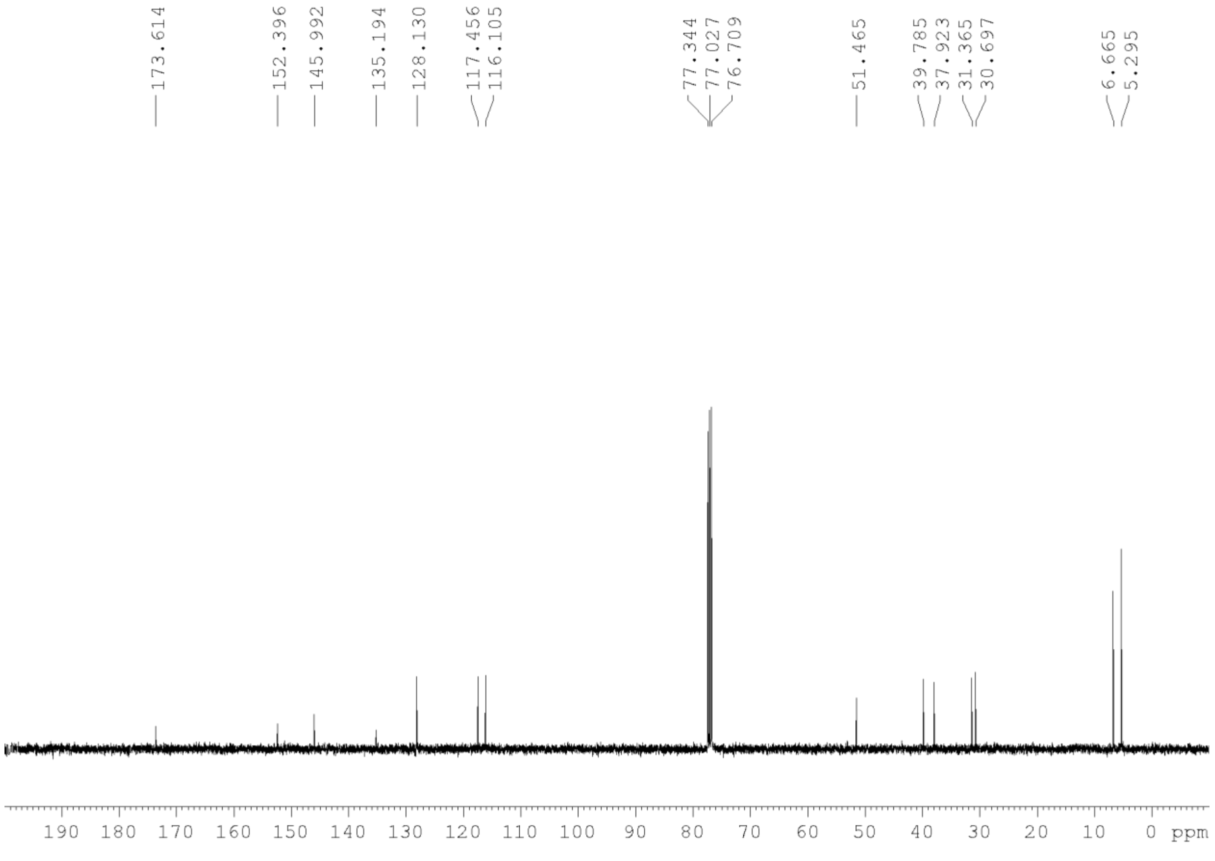

2-(7-((Trimethylsilyl)oxy)-2,3-dihydro-*1H*-inden-1-yl)acetaldehyde, 65

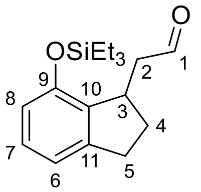

^{1}H (400 MHz, $CDCl_3$)
^{13}C (101 MHz, $CDCl_3$)

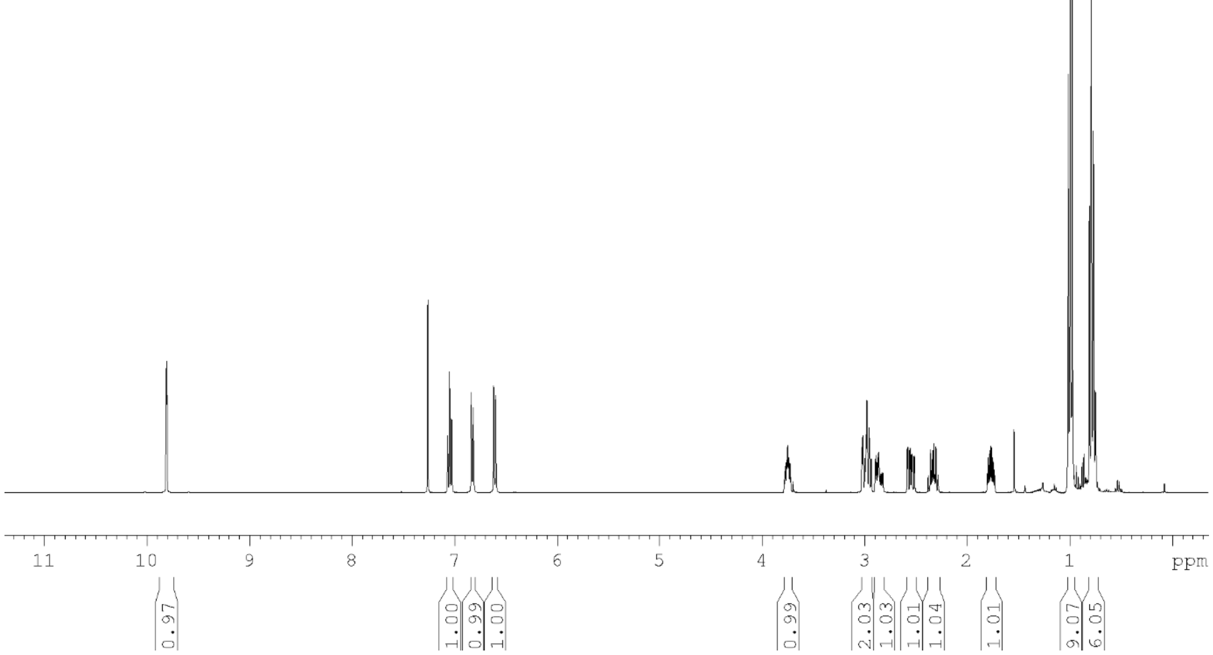

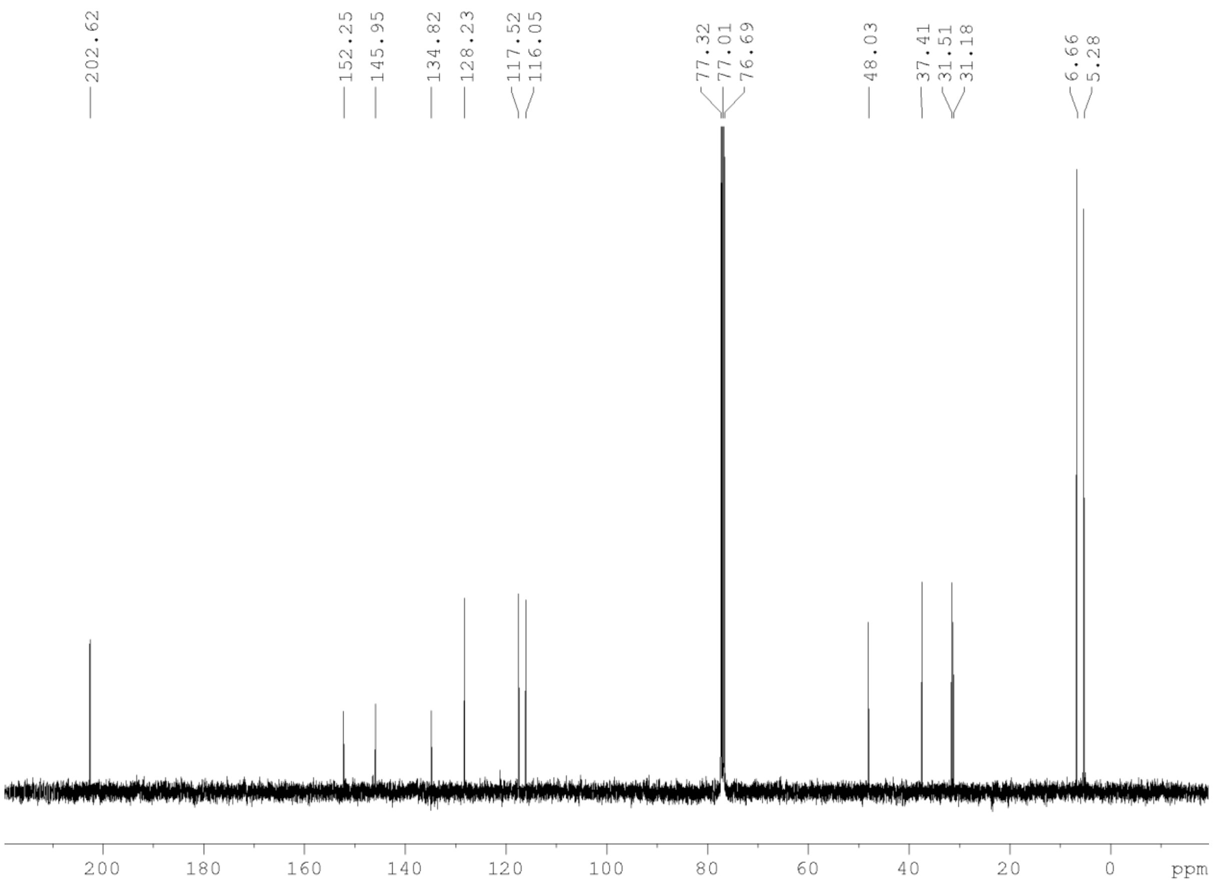

5-(1-Hydroxy-2-(7-((triethylsilyl)oxy)-2,3-dihydro-*1H*-inden-1-yl)ethyl)-3-methylfuran-2(*5H*)-one, 68

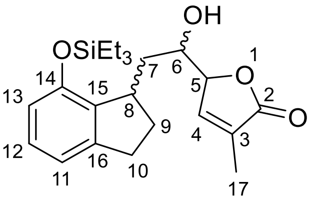

^{1}H (500 MHz, $CDCl_3$)
^{13}C (125 MHz, $CDCl_3$)

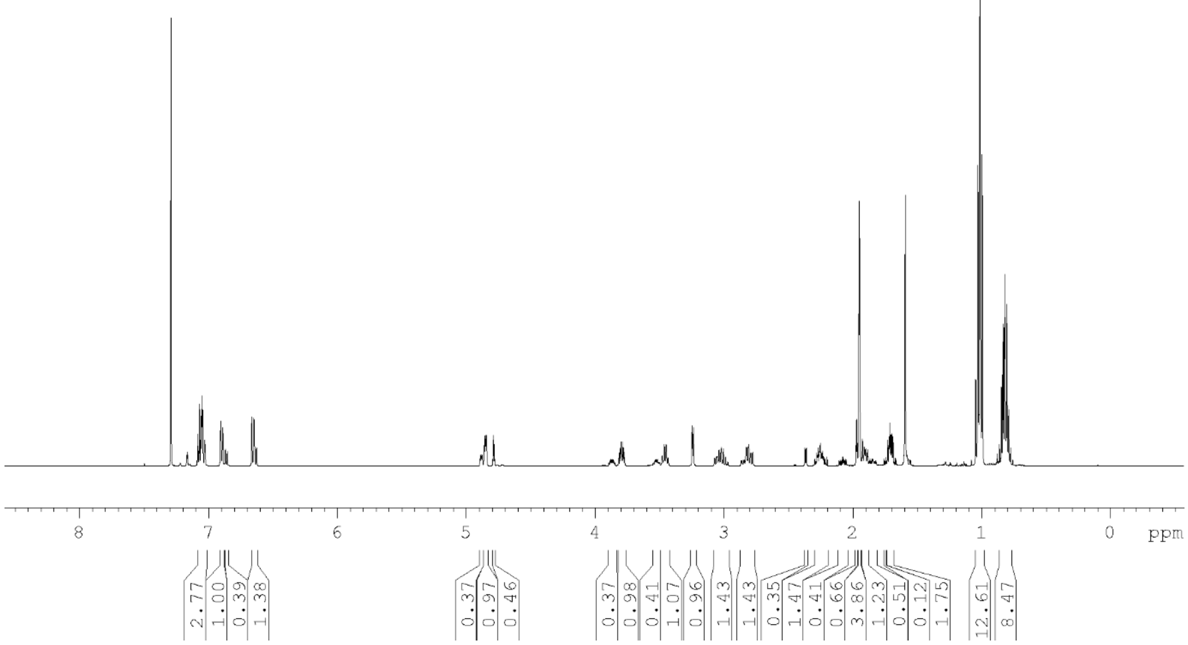

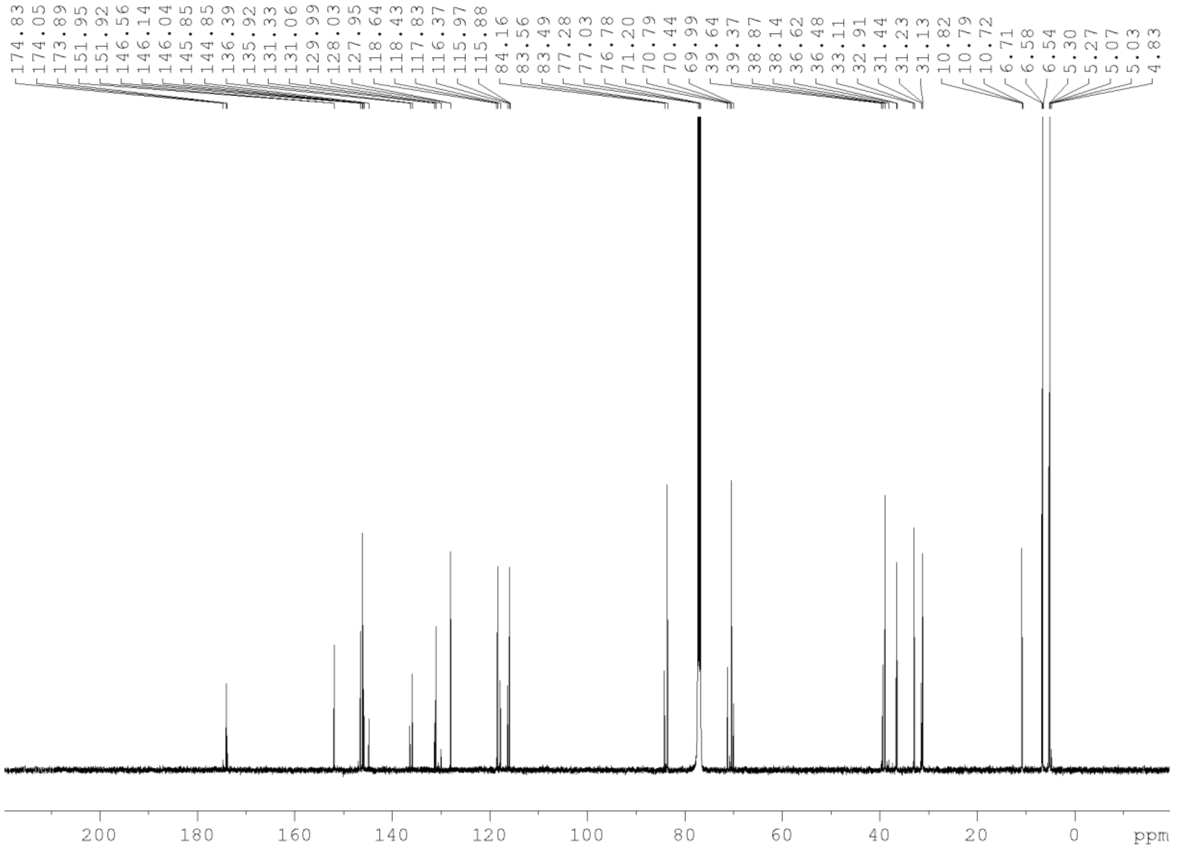

(*E*)-3-Methyl-5-(2-(7-((triethylsilyl)oxy)-2,3-dihydro-*1H*-inden-1-yl)ethylidene)furan-2(*5H*)-one, S12

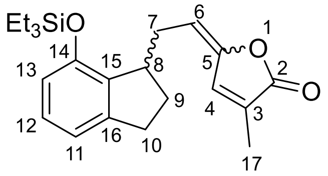

^{1}H (400 MHz, $CDCl_3$)
^{13}C (101 MHz, $CDCl_3$)

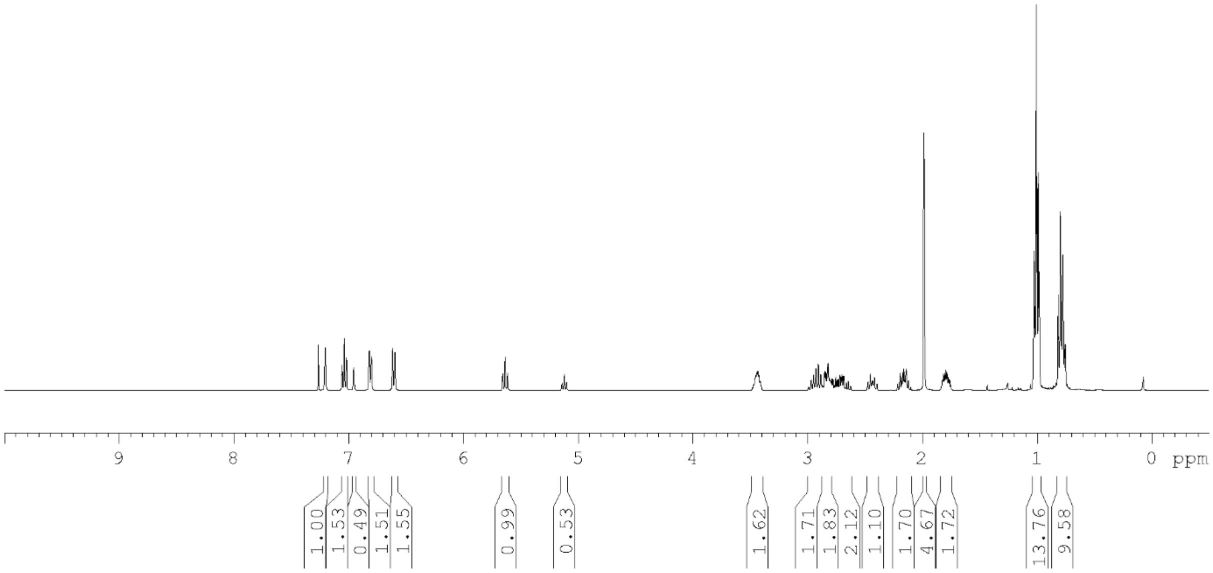

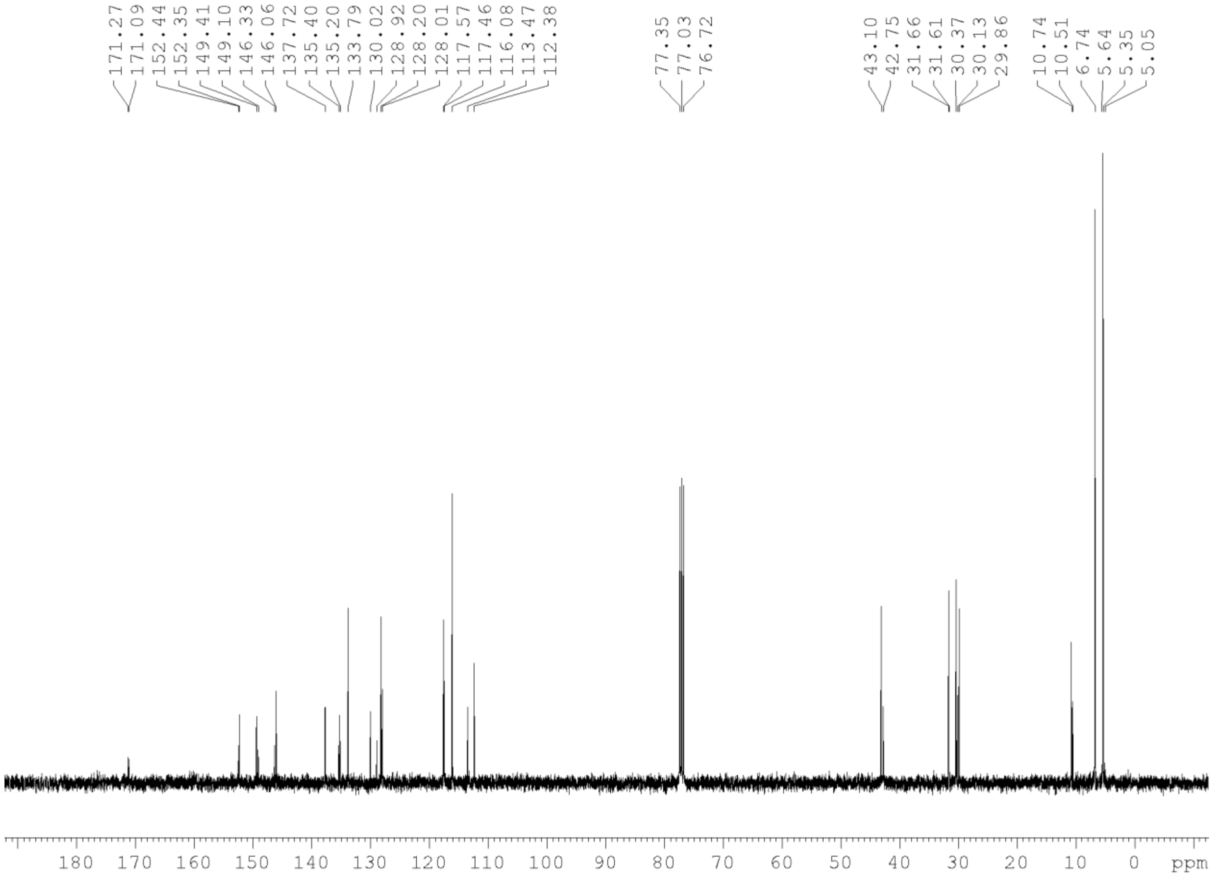

(*E*)-5-(2-(7-hydroxy-2,3-dihydro-*1H*-inden-1-yl)ethylidene)-3-methylfuran-2(*5H*)-one, 69

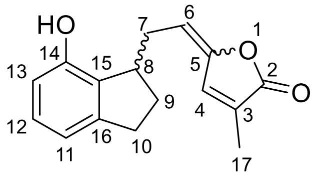

^{1}H (400 MHz, $CDCl_3$)
^{13}C (101 MHz, $CDCl_3$)

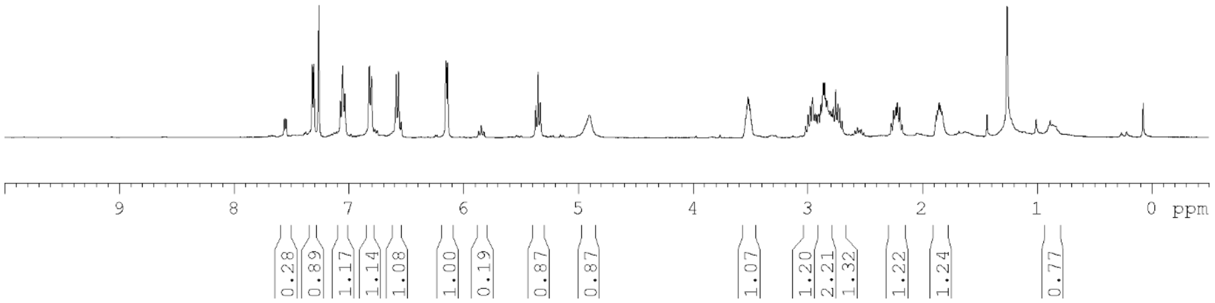

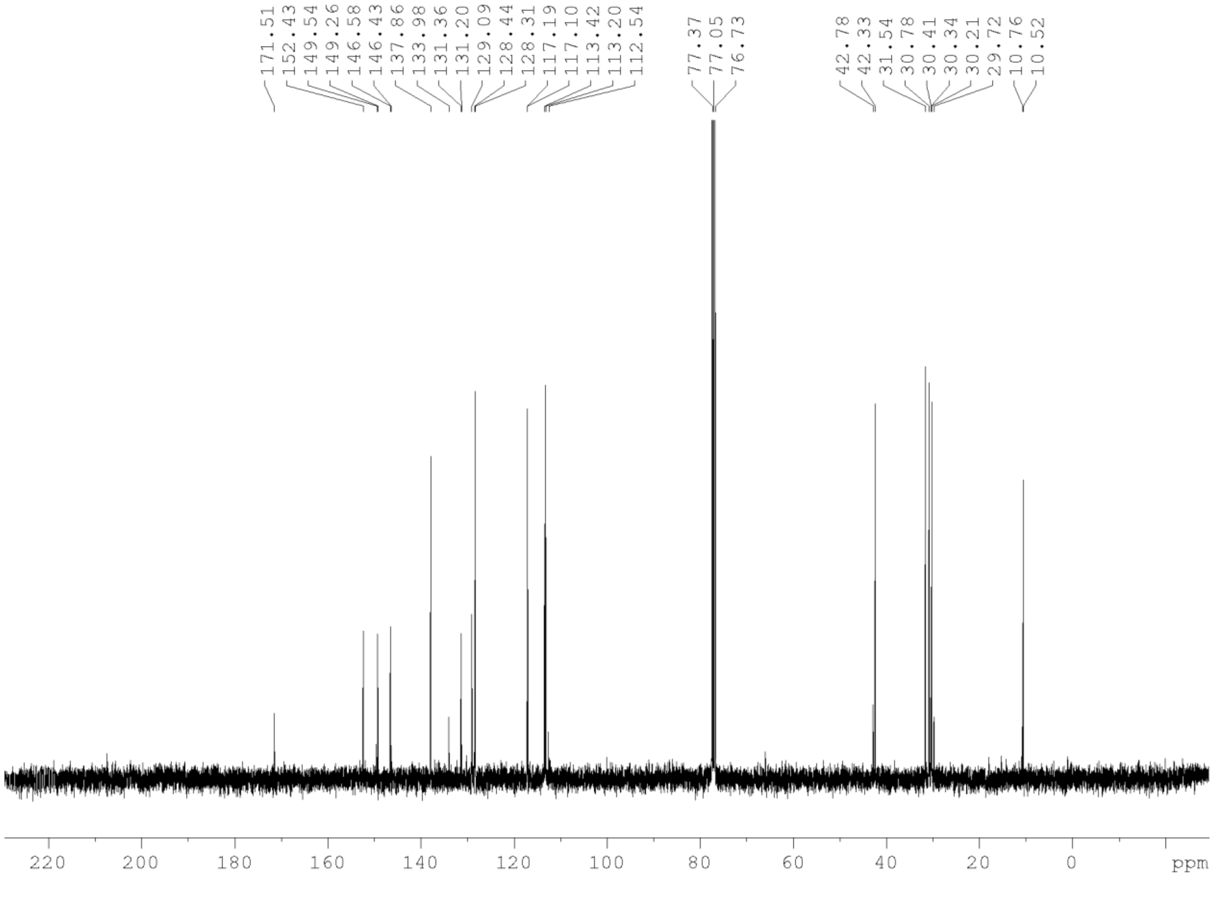

5-(1-Hydroxy-2-(7-hydroxy-2,3-dihydro-*1H*-inden-1-yl)ethyl)-3-methylfuran-2(*5H*)-one, 71

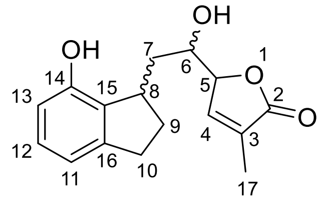

^{1}H (400 MHz, $CDCl_3$)
^{13}C (101 MHz, $CDCl_3$)

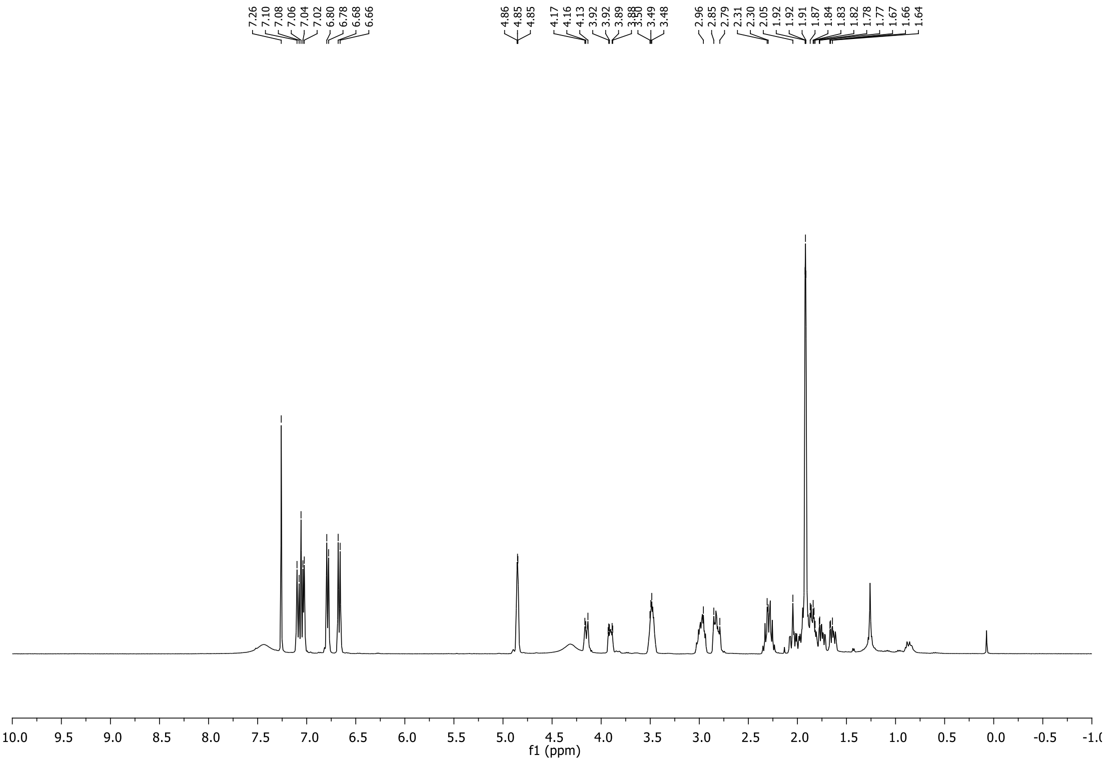

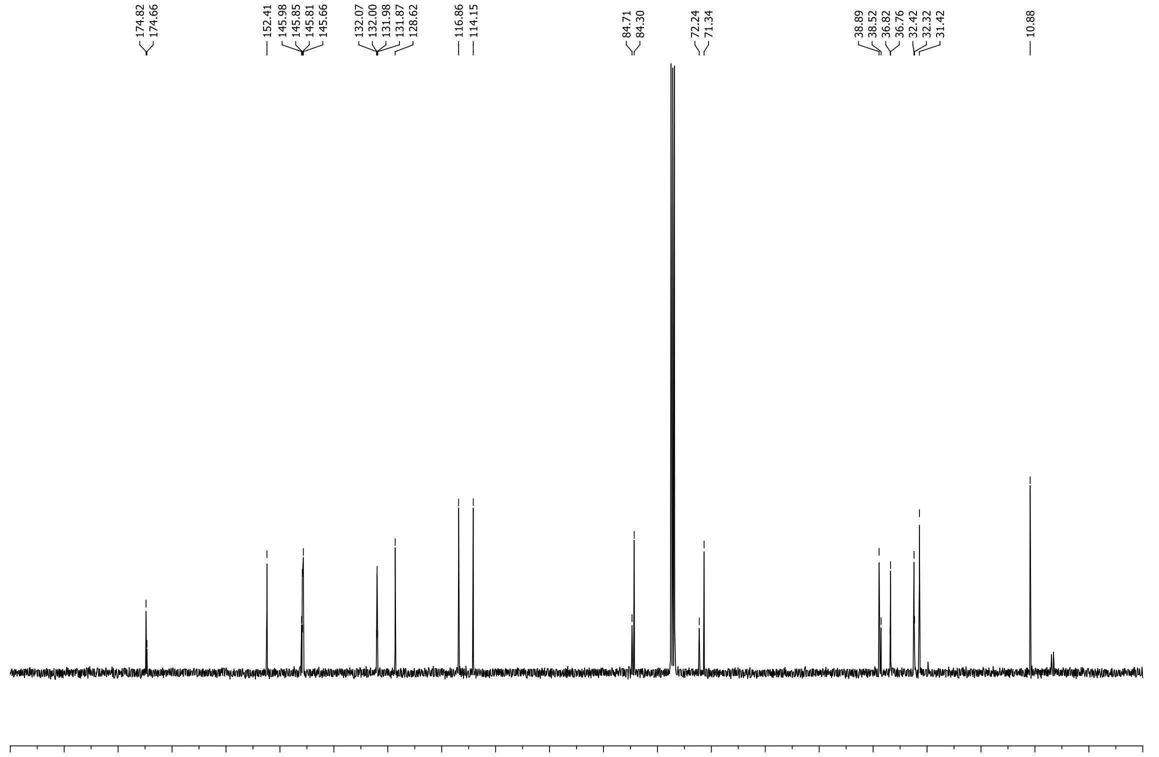

3-(2-Hydroxy-2-(4-methyl-5-oxo-2,5-dihydrofuran-2-yl)ethyl)-2,3-dihydro-*1H*-inden-4-yl trifluoromethanesulfonate, 72

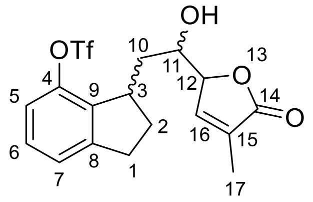

^{1}H (400 MHz, $CDCl_3$)
^{13}C (101 MHz, $CDCl_3$)

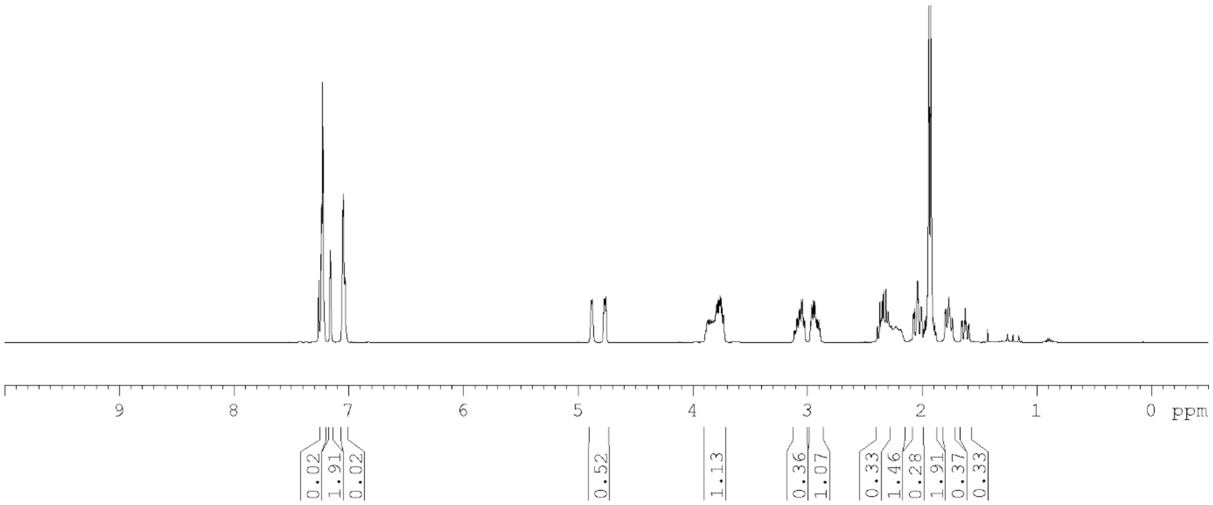

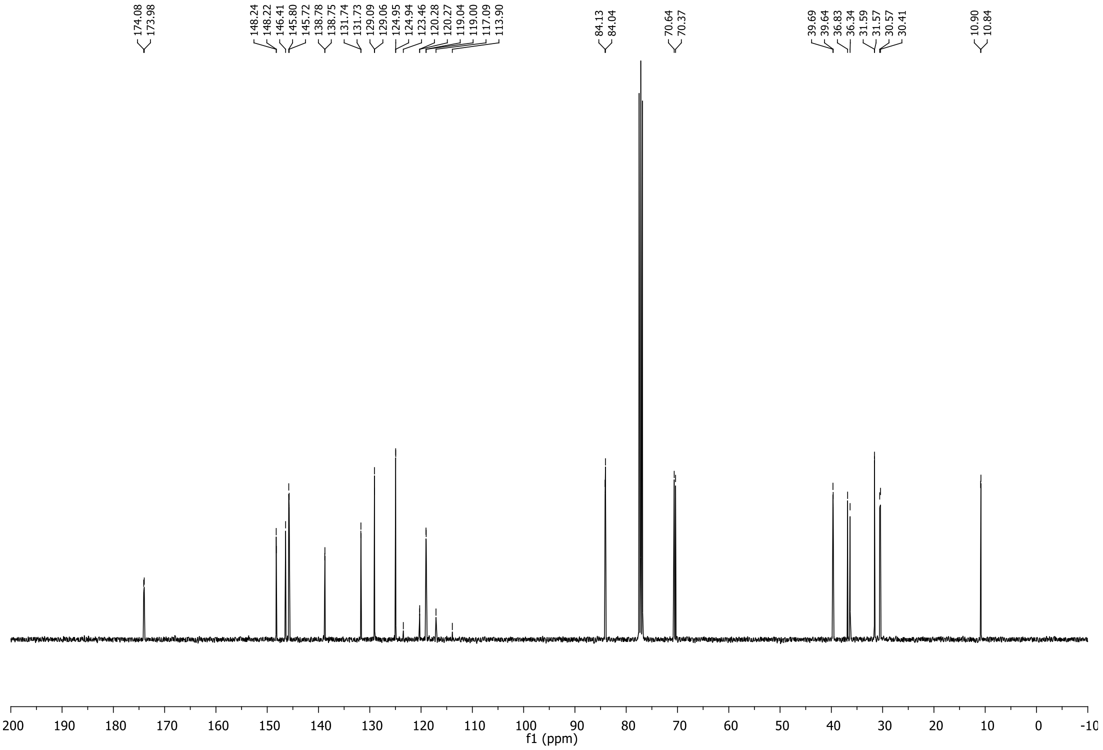

3-Methyl-5-(3,3a,4,5-tetrahydro-*2H*-cyclopenta[*de*]chromen-2-yl)dihydrofuran-2(*3H*)-one, 73a (unassigned)

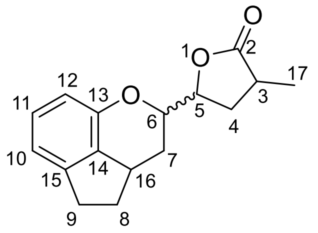

^{1}H (400 MHz, $CDCl_3$)
^{13}C (101 MHz, $CDCl_3$)

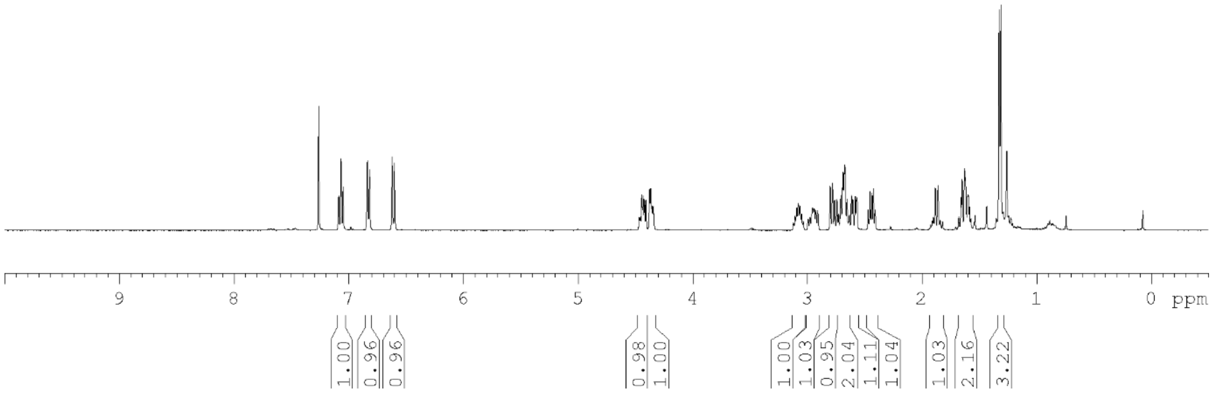

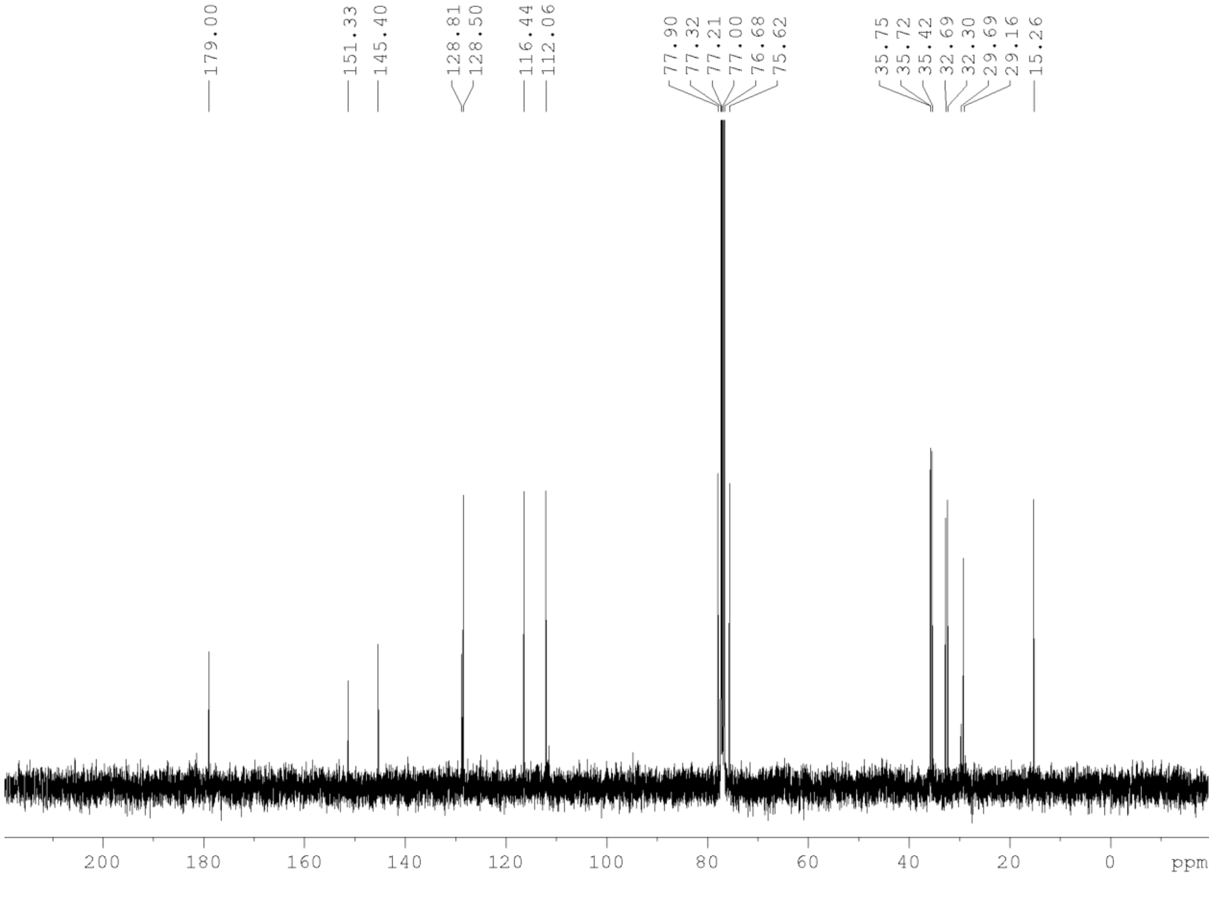

3-Methyl-5-(3,3a,4,5-tetrahydro-*2H*-cyclopenta[*de*]chromen-2-yl)dihydrofuran-2(*3H*)-one, 73b (unassigned)

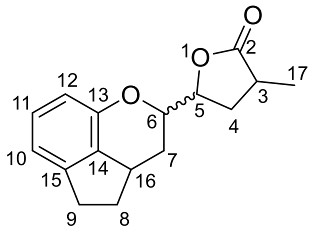

^{1}H (400 MHz, $CDCl_3$)
^{13}C (101 MHz, $CDCl_3$)

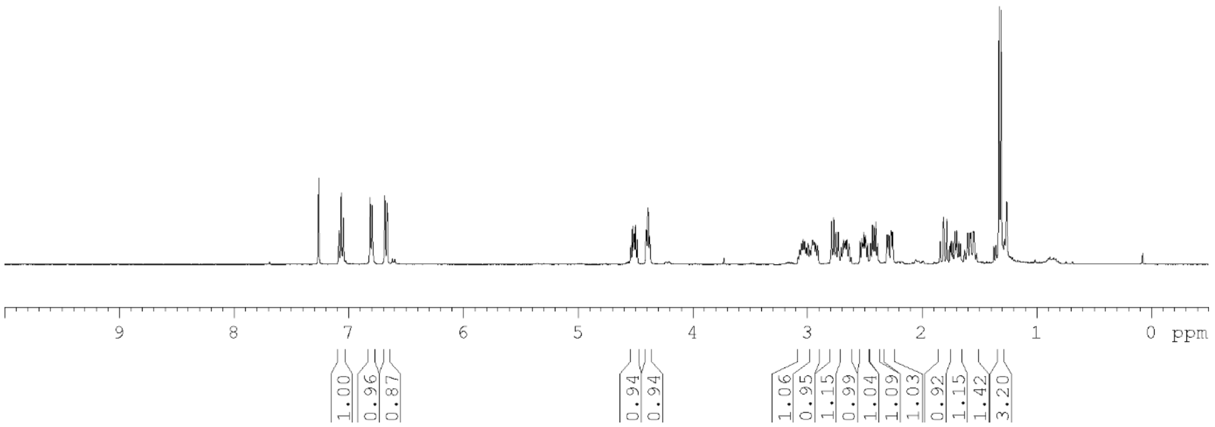

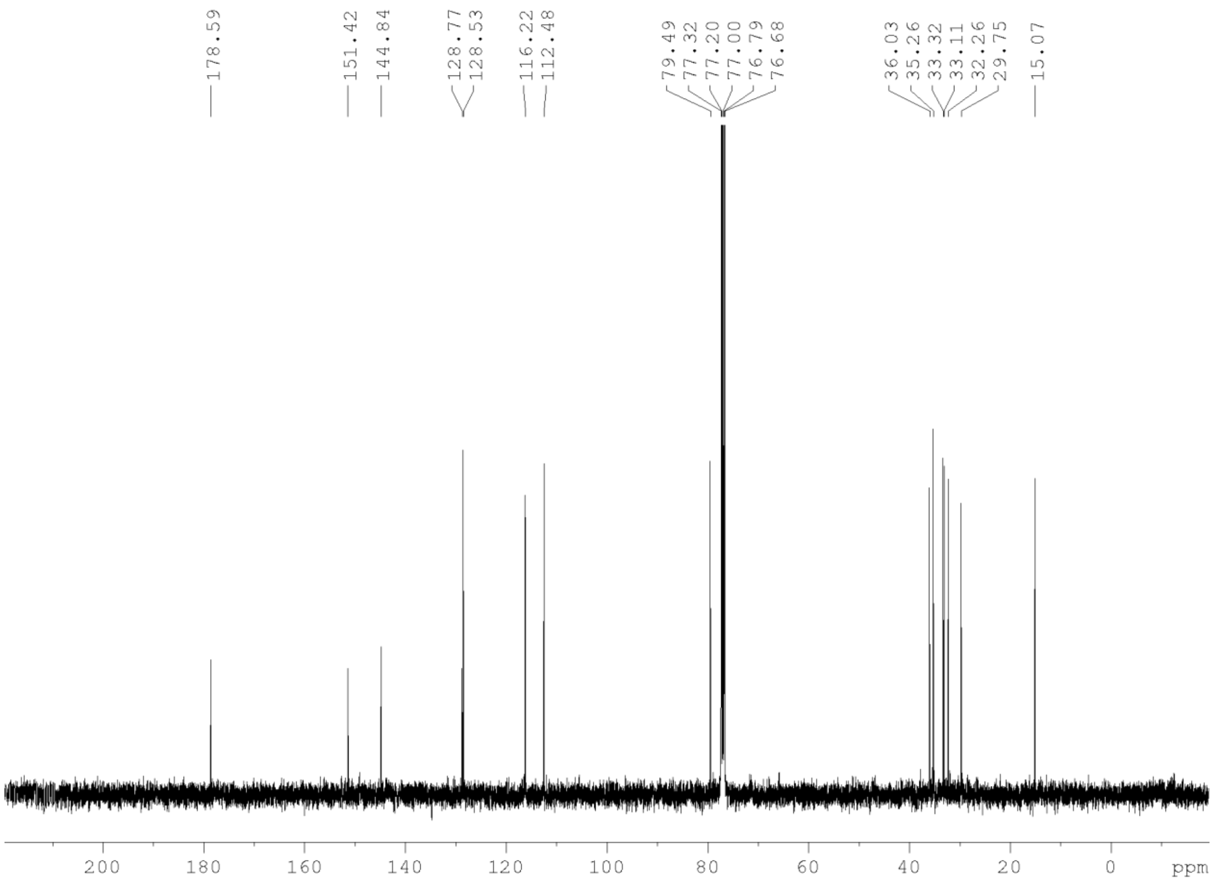

5-(1-Hydroxy-2-(7-hydroxy-2,3-dihydro-*1H*-inden-1-yl)ethyl)-3-methyldihydrofuran-2(*3H*)-one, 74

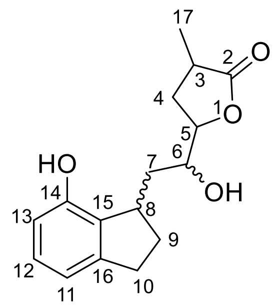

^{1}H (400 MHz, $CDCl_3$)
^{13}C (101 MHz, $CDCl_3$)

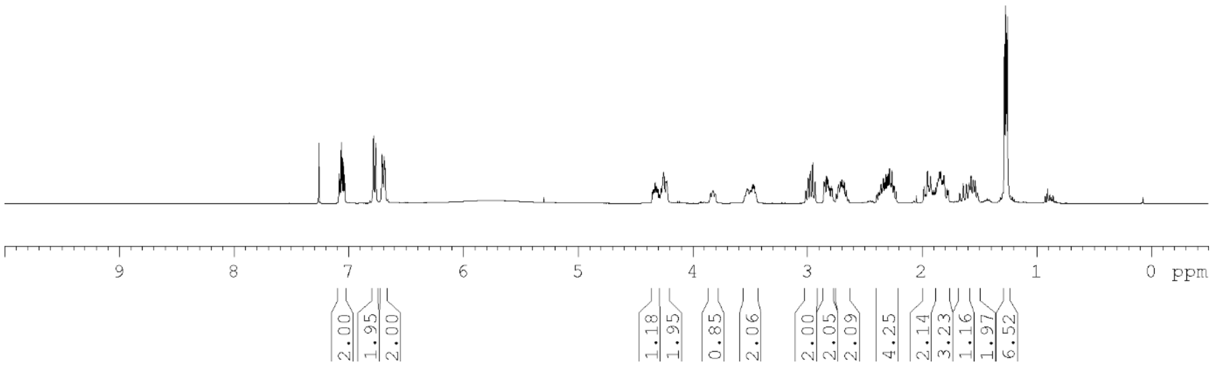

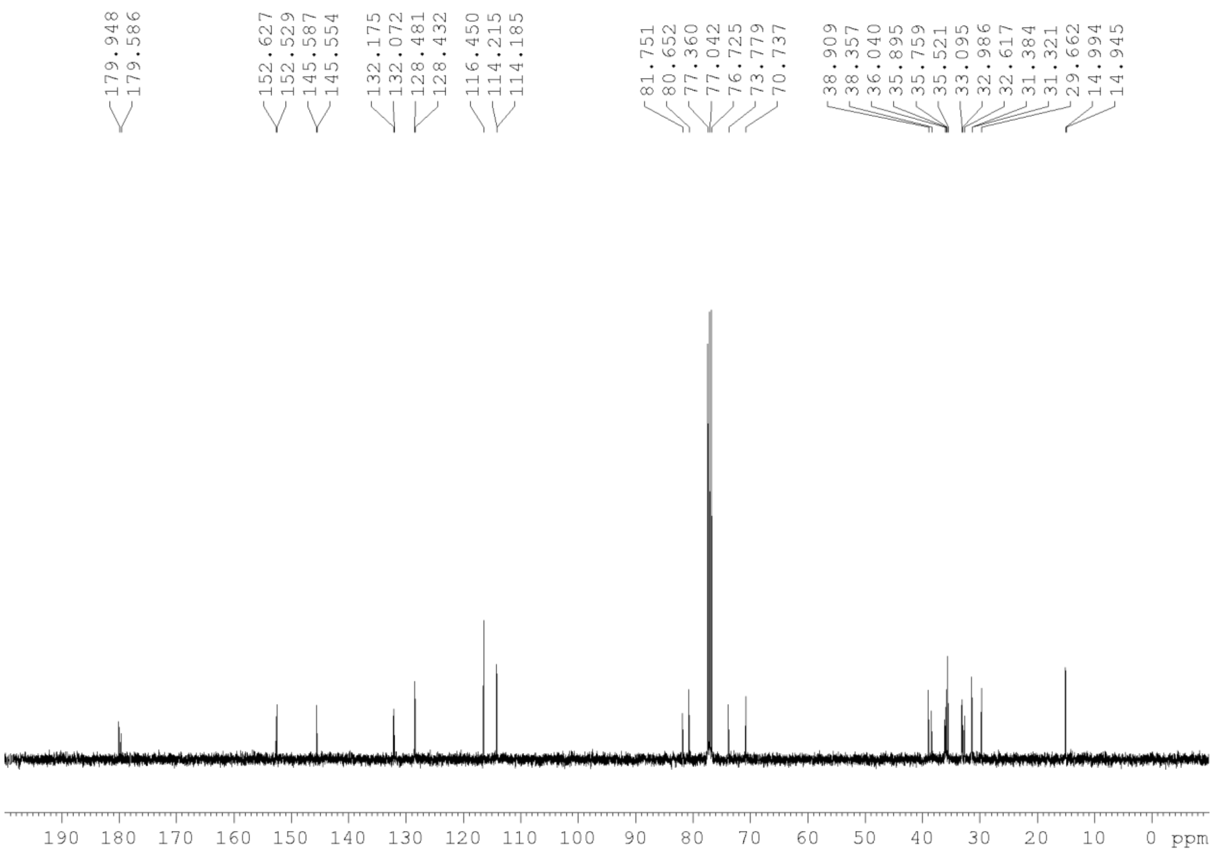

7-(Methoxymethoxy)-2,3-dihydro-1*H*-inden-1-one, S13

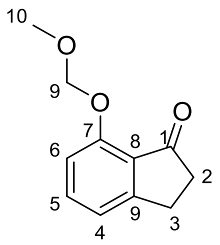

^{1}H (400 MHz, $CDCl_3$)
^{13}C (101 MHz, $CDCl_3$)

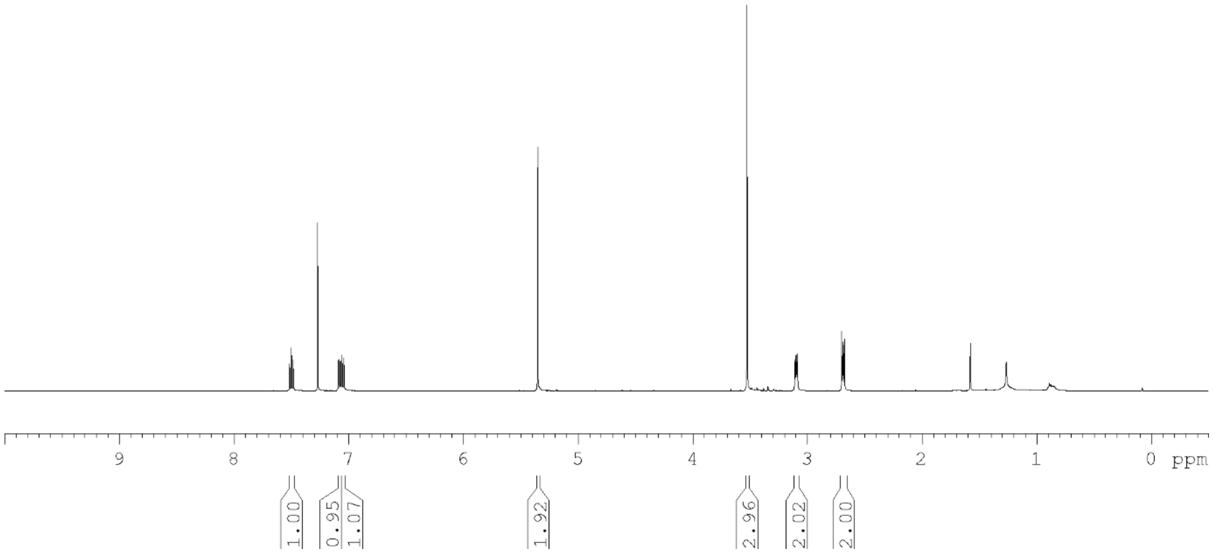

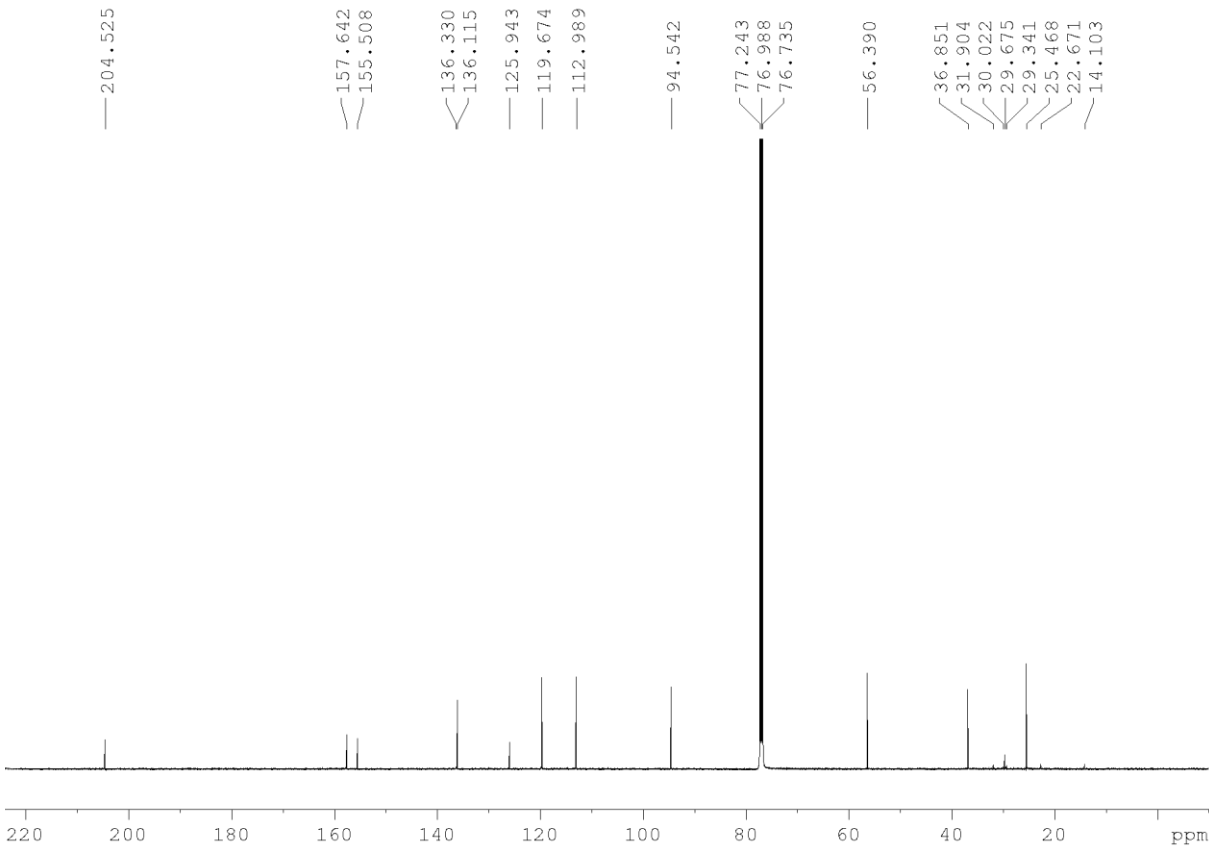

Ethyl (*E*)-2-(7-(methoxymethoxy)-2,3-dihydro-1*H*-inden-1-ylidene)acetate, S14

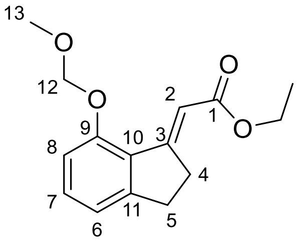

^{1}H (400 MHz, $CDCl_3$)
^{13}C (101 MHz, $CDCl_3$)

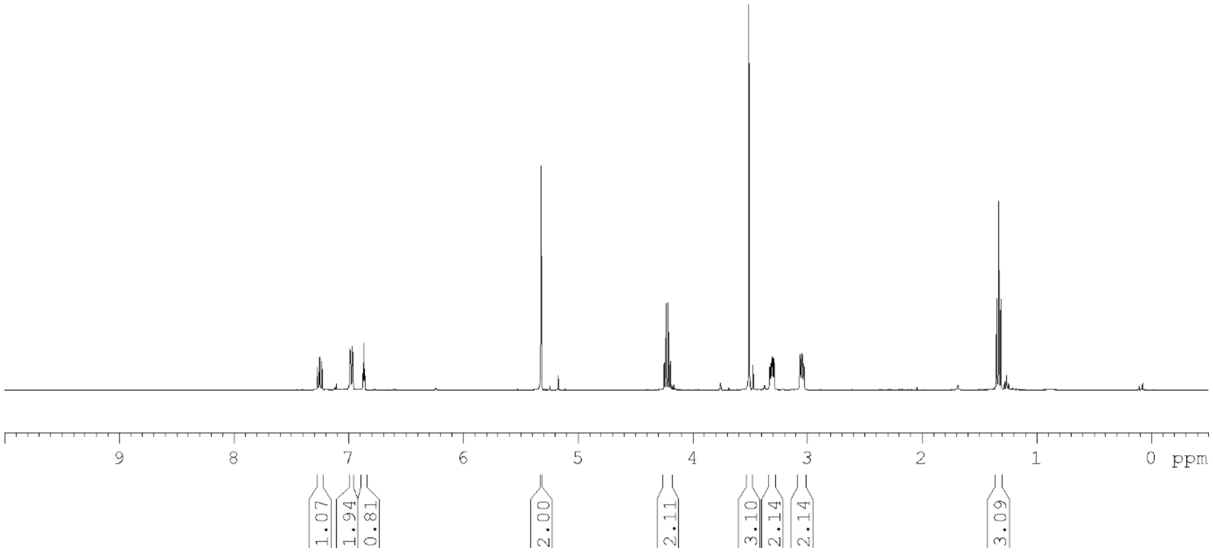

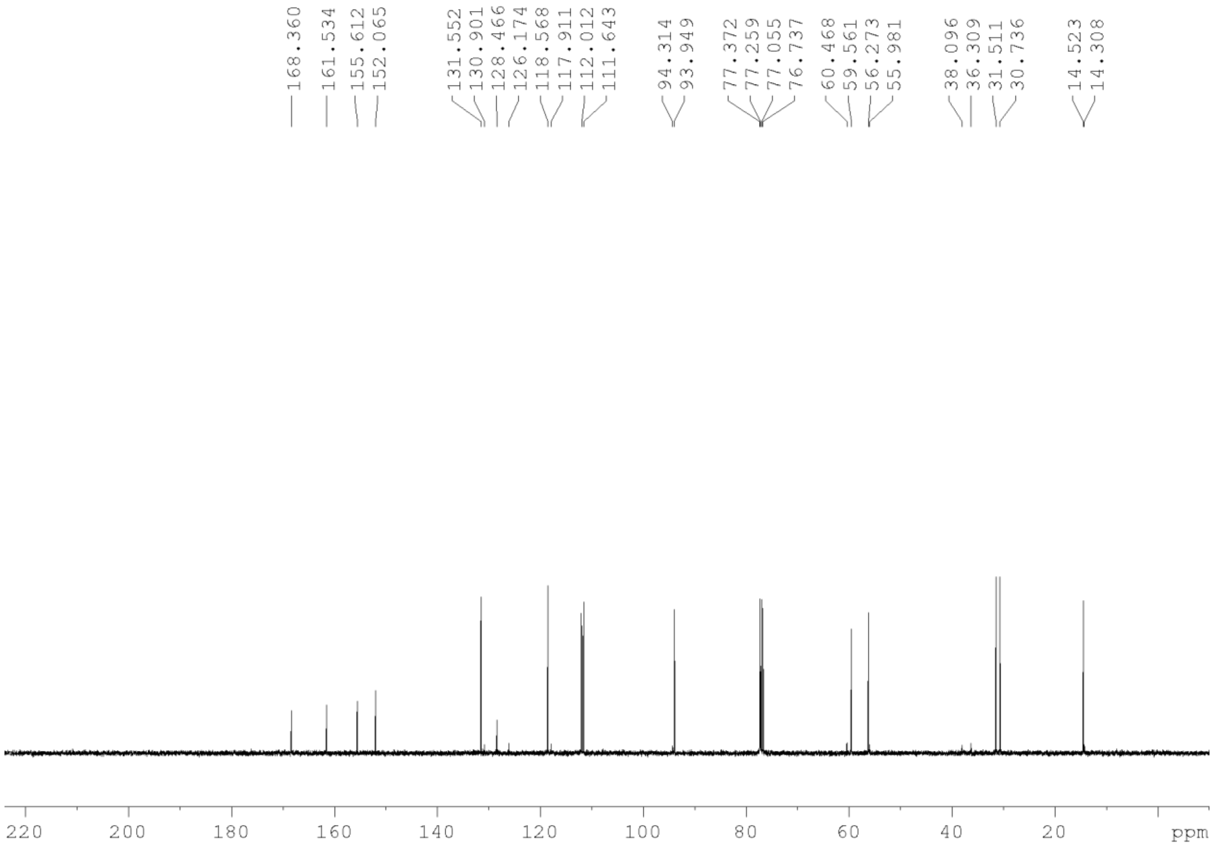

Ethyl 2-(7-(methoxymethoxy)-2,3-dihydro-1*H*-inden-1-yl)acetate, S15

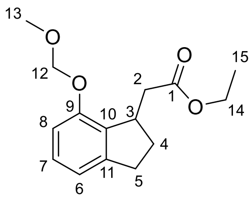

^{1}H (400 MHz, $CDCl_3$)
^{13}C (101 MHz, $CDCl_3$)

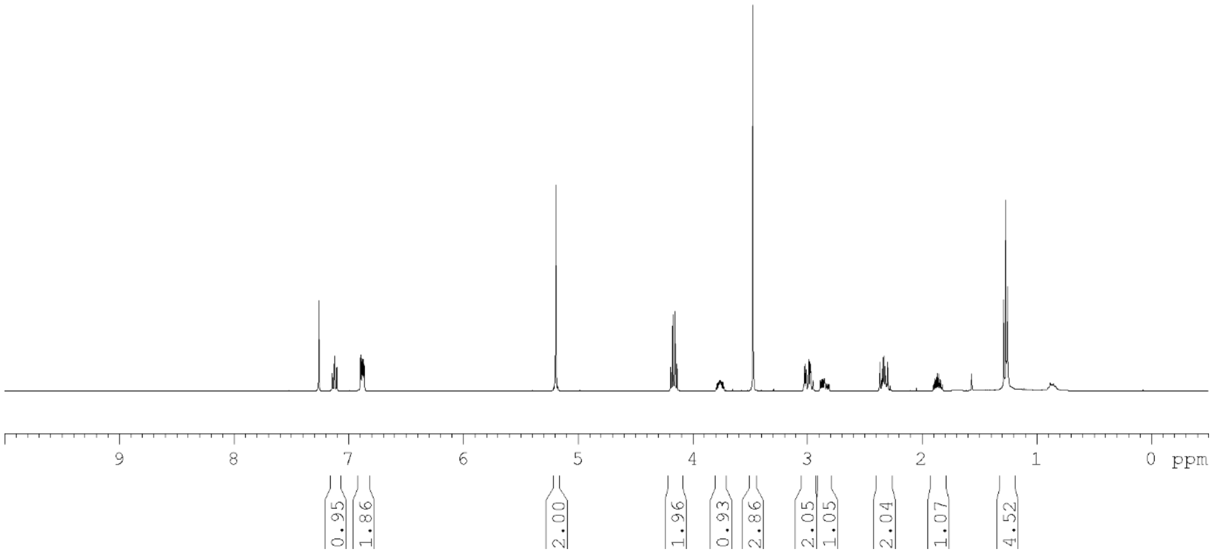

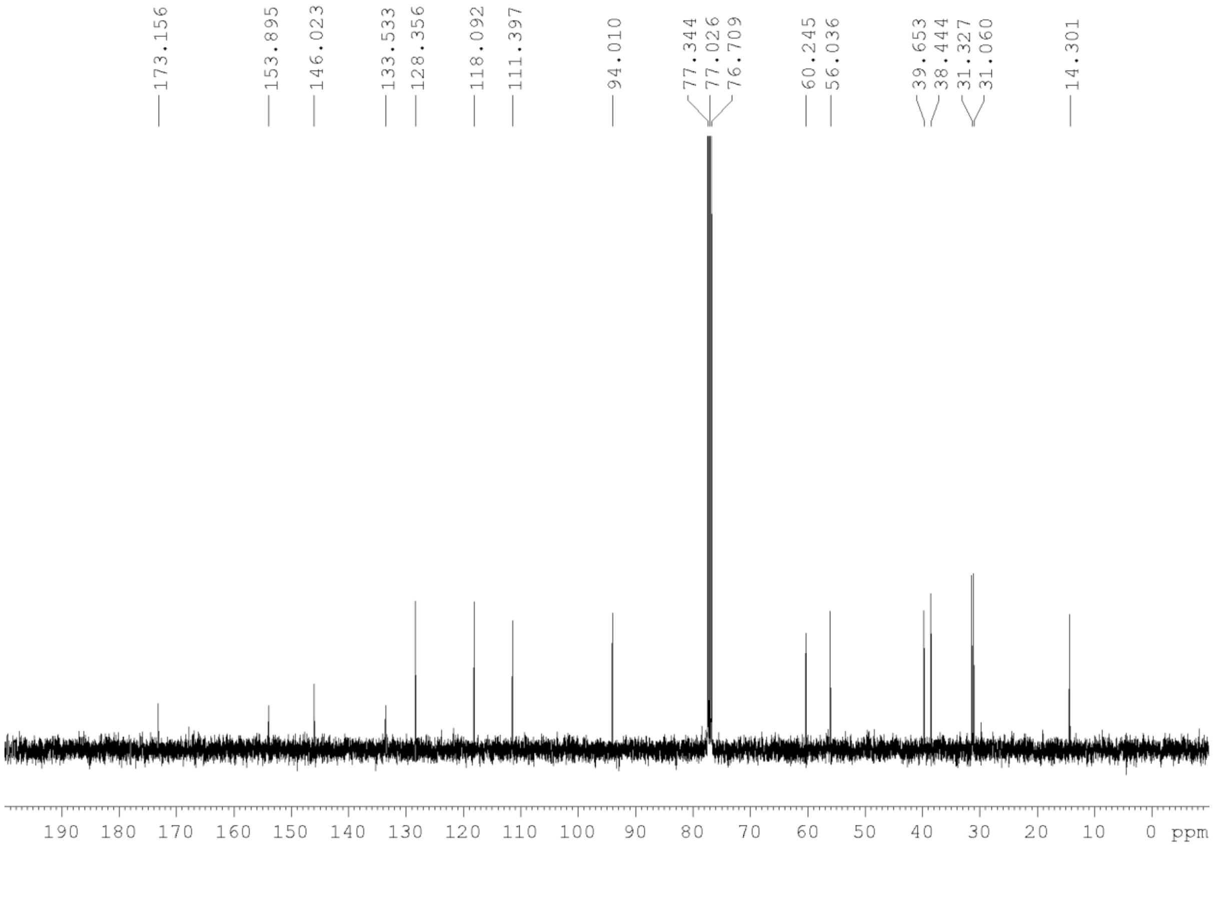

2-(7-(Methoxymethoxy)-2,3-dihydro-1*H*-inden-1-yl)ethan-1-ol, S16

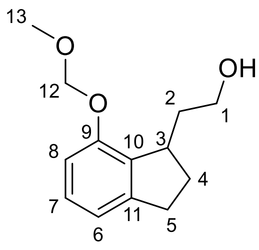

^{1}H (400 MHz, $CDCl_3$)

^{13}C (101 MHz, $CDCl_3$)

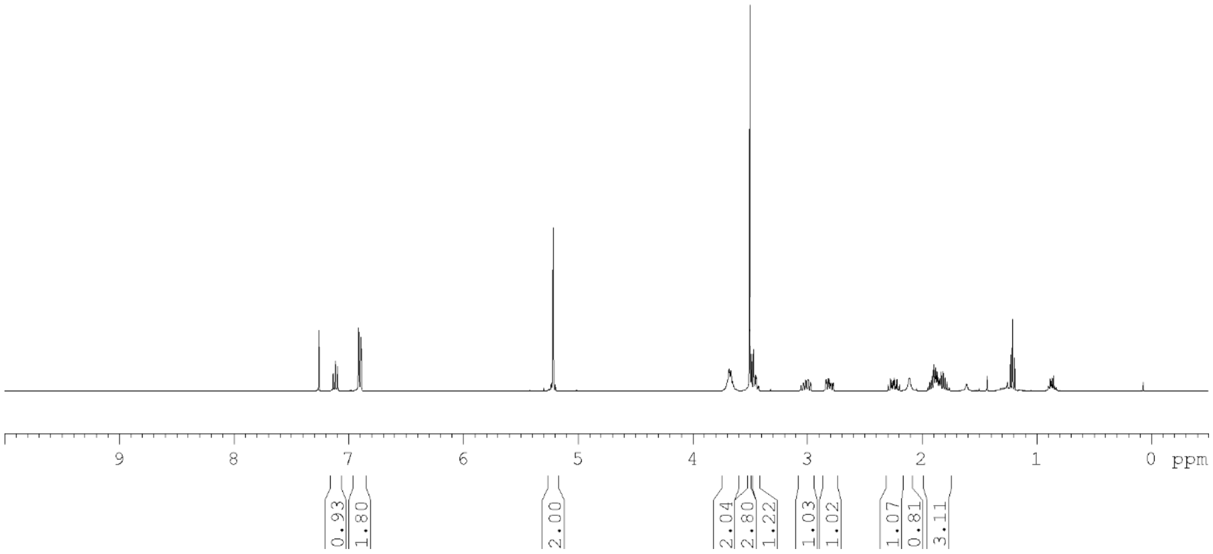

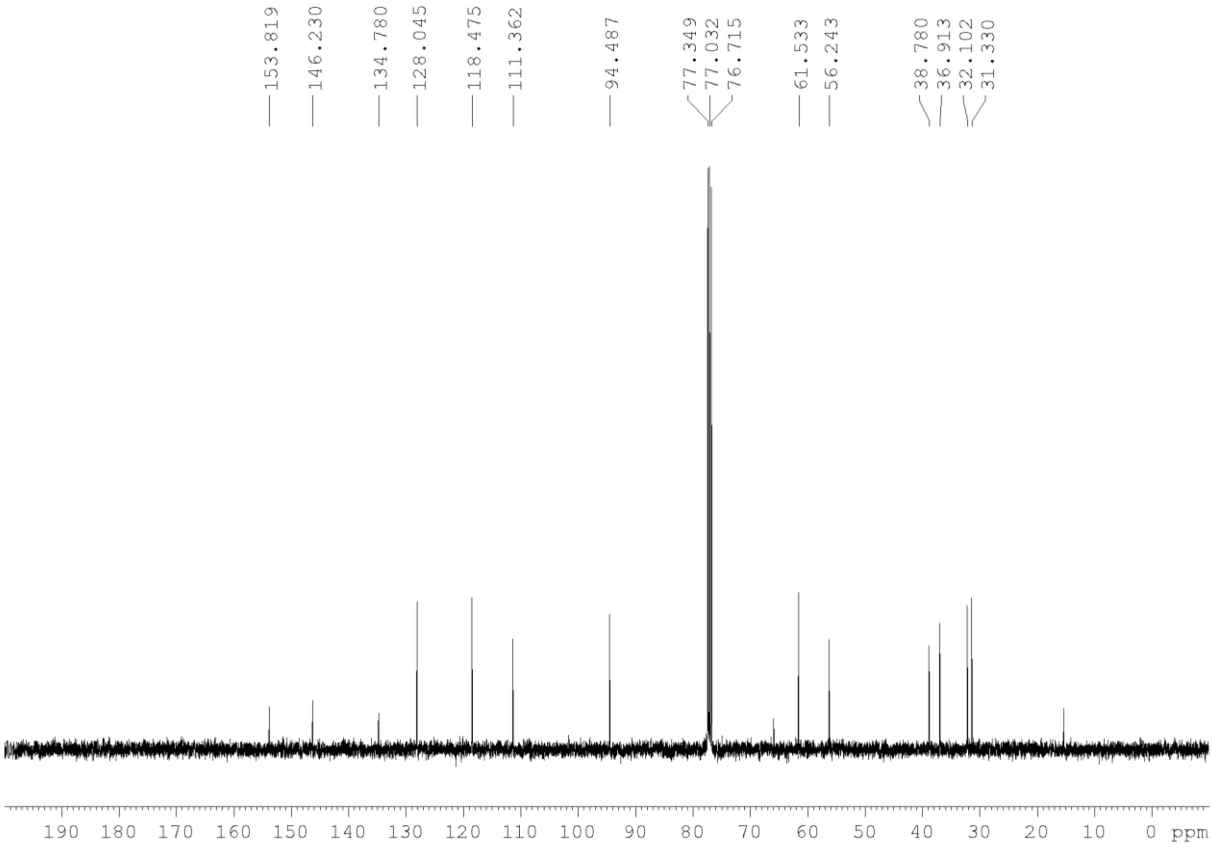

2-(7-(Methoxymethoxy)-2,3-dihydro-1*H*-inden-1-yl)acetaldehyde, S17

^{1}H (400 MHz, $CDCl_3$)
^{13}C (101 MHz, $CDCl_3$)

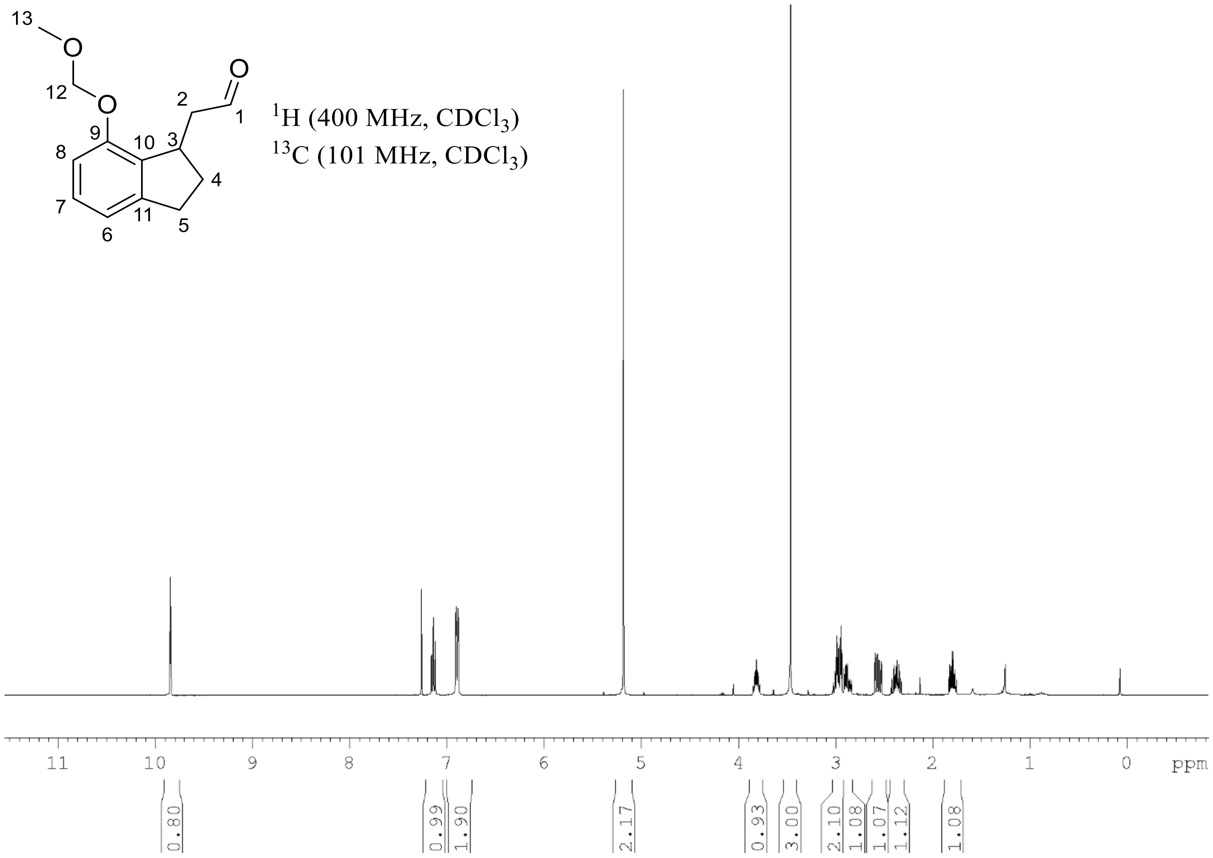

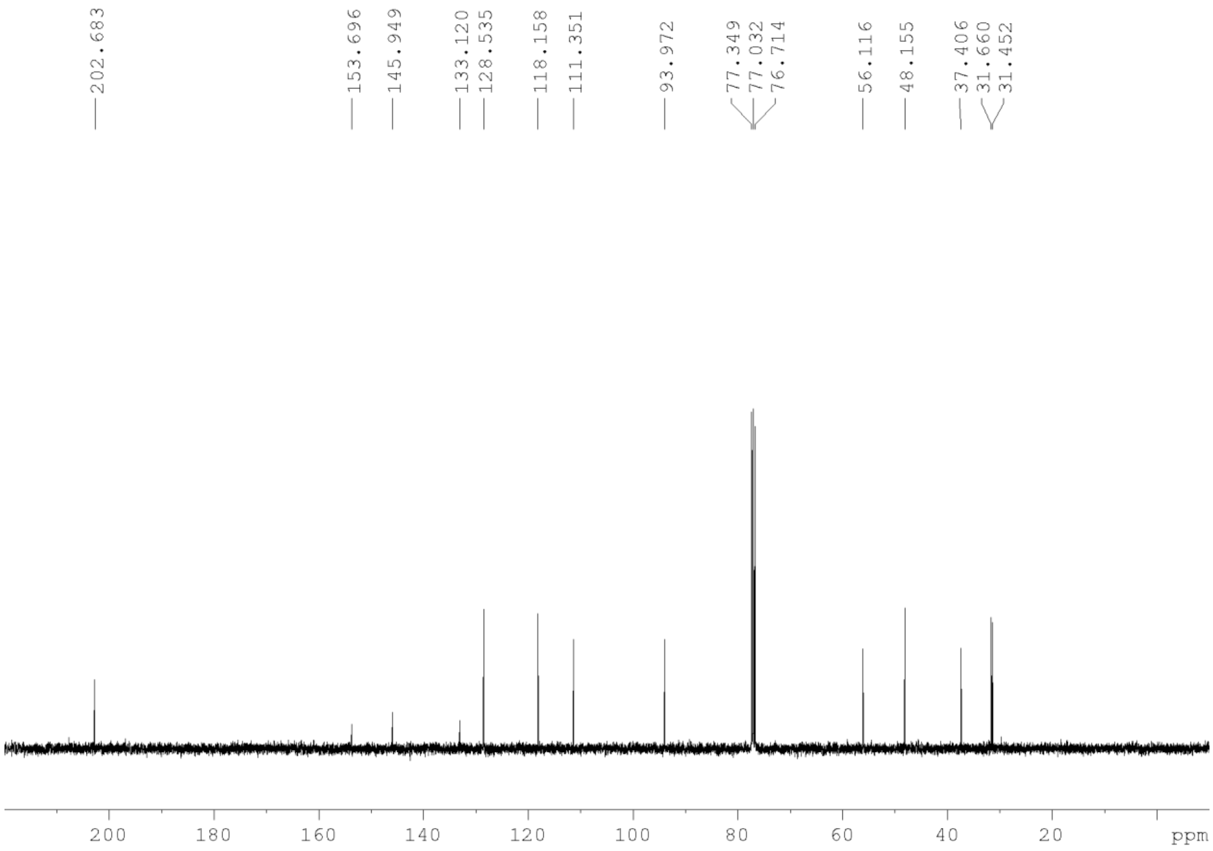

Equilibrium mixture of aldehyde 2-(7-hydroxy-2,3-dihydro-1*H*-inden-1-yl)acetaldehyde and lactols 3,3a,4,5-tetrahydro-2*H*-cyclopenta[*de*]chromen-2-ol, 75

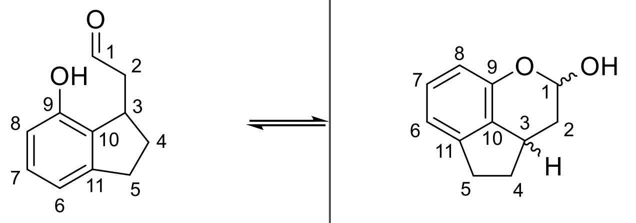

^{1}H (500 MHz, $CDCl_3$)
^{13}C (125 MHz, $CDCl_3$)

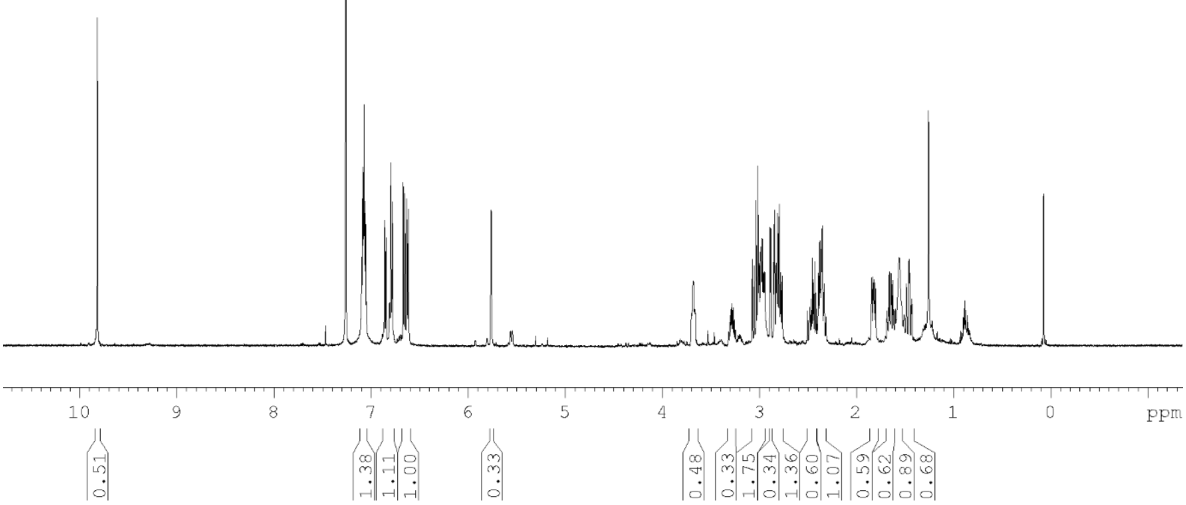

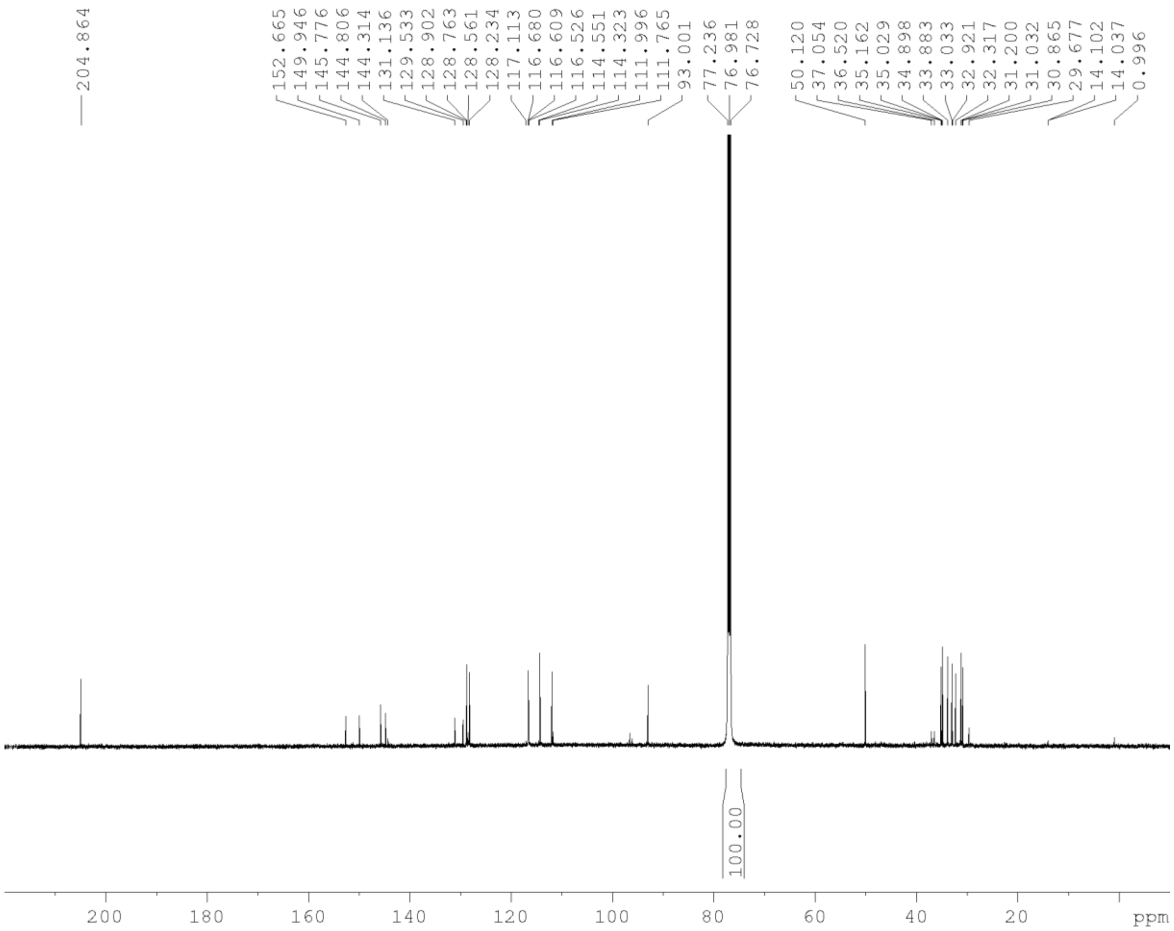

2-Chloro-3,3a,4,5-tetrahydro-*2H*-cyclopenta[*de*]chromene, 76

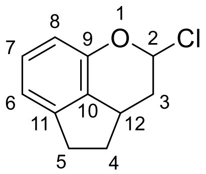

^{1}H (400 MHz, $CDCl_3$)
^{13}C (101 MHz, $CDCl_3$)

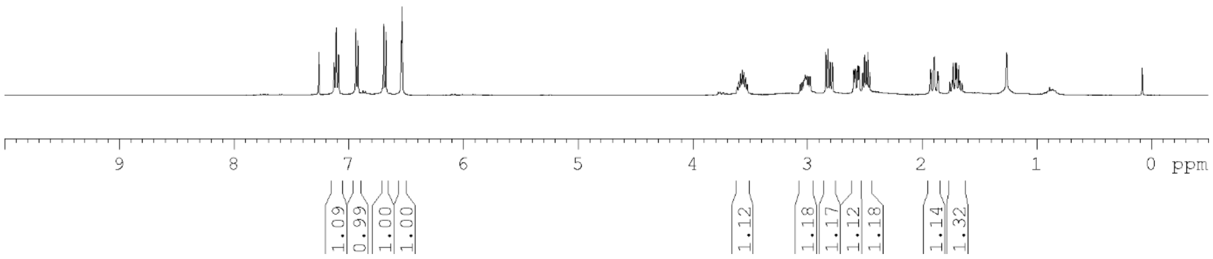

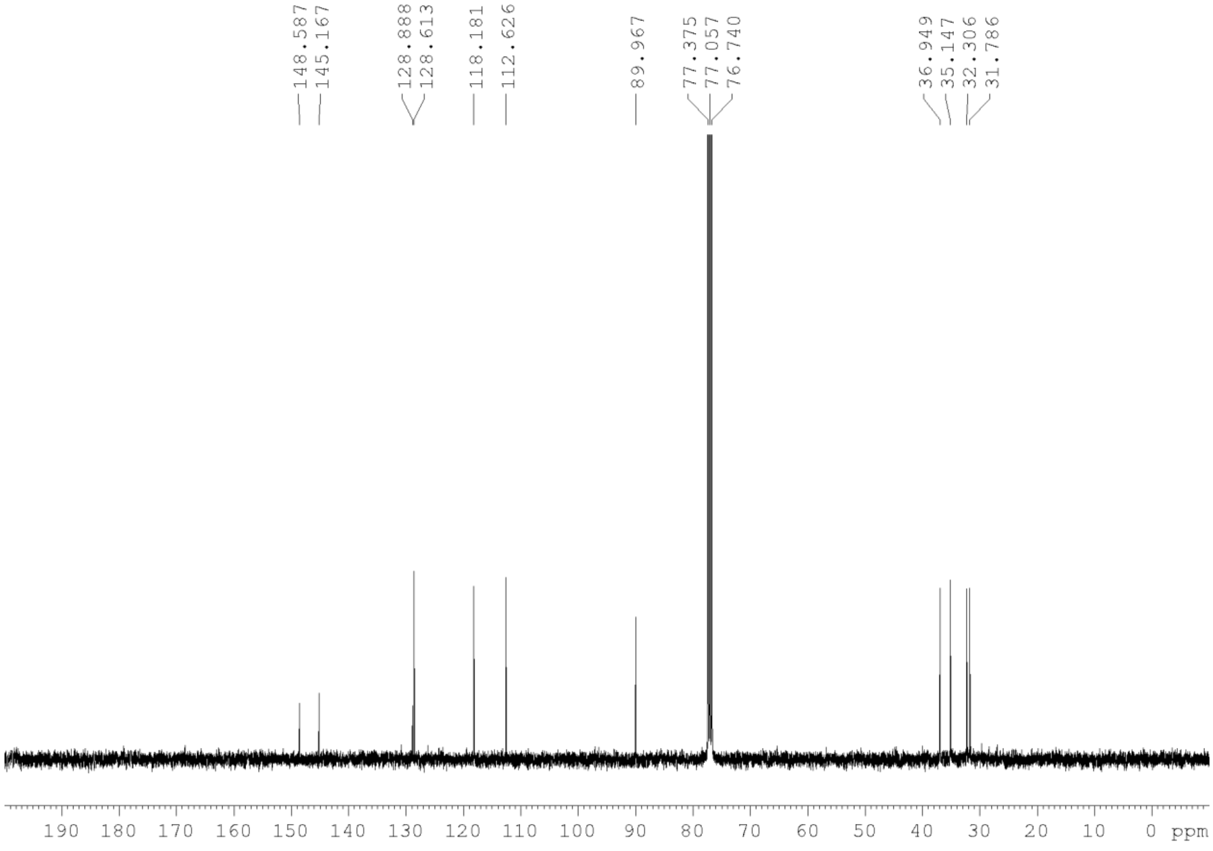

3-Methyl-5-(3,3a,4,5-tetrahydro-*2H*-cyclopenta[*de*]chromen-2-yl)furan-2(*5H*)-one, 78a (unassigned)

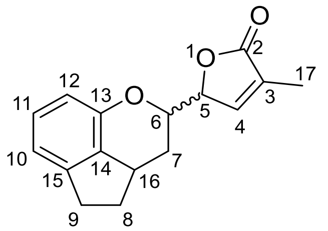

^{1}H (400 MHz, $CDCl_3$)
^{13}C (101 MHz, $CDCl_3$)

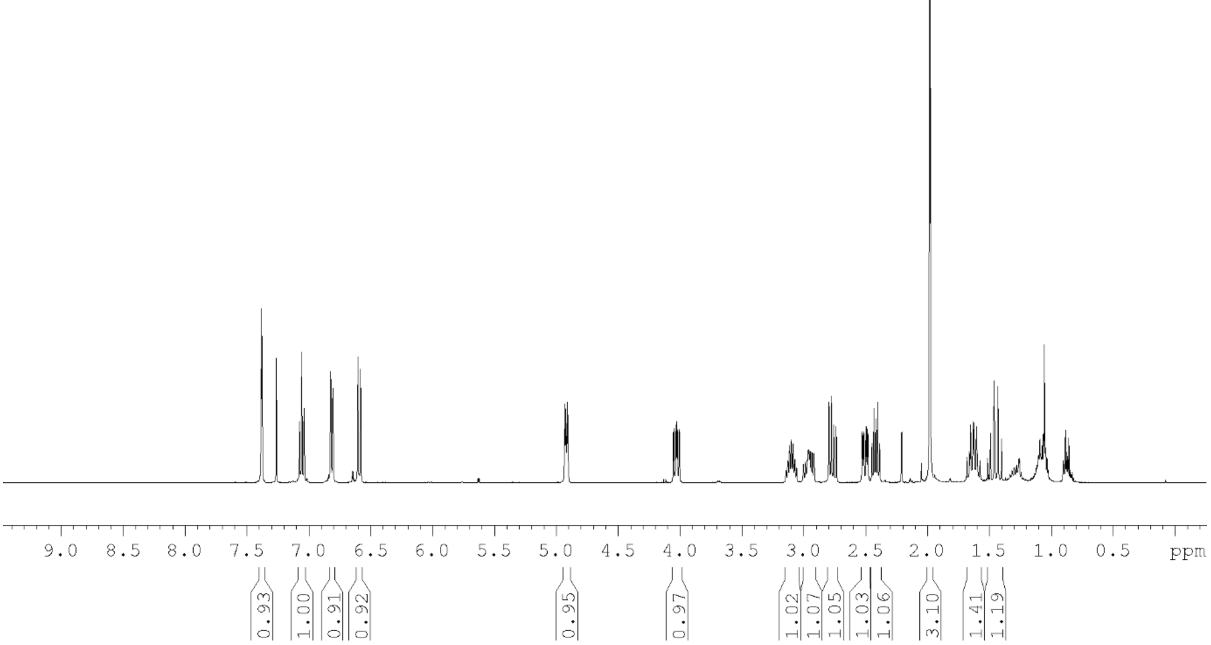

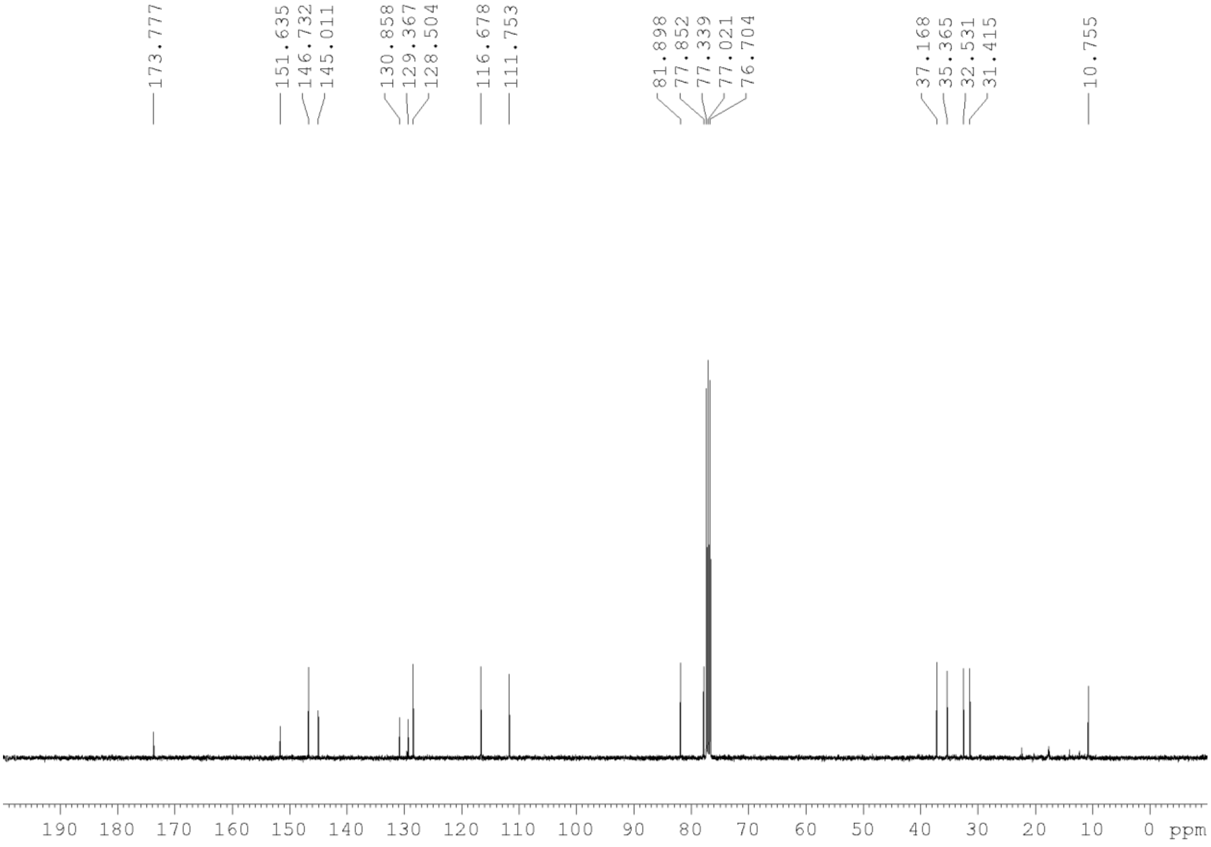

3-Methyl-5-(3,3a,4,5-tetrahydro-*2H*-cyclopenta[*de*]chromen-2-yl)furan-2(*5H*)-one, 78b (unassigned)

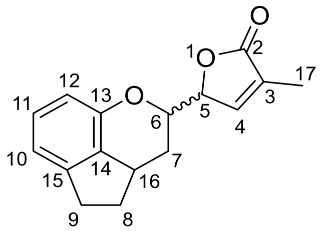

^{1}H (400 MHz, $CDCl_3$)
^{13}C (101 MHz, $CDCl_3$)

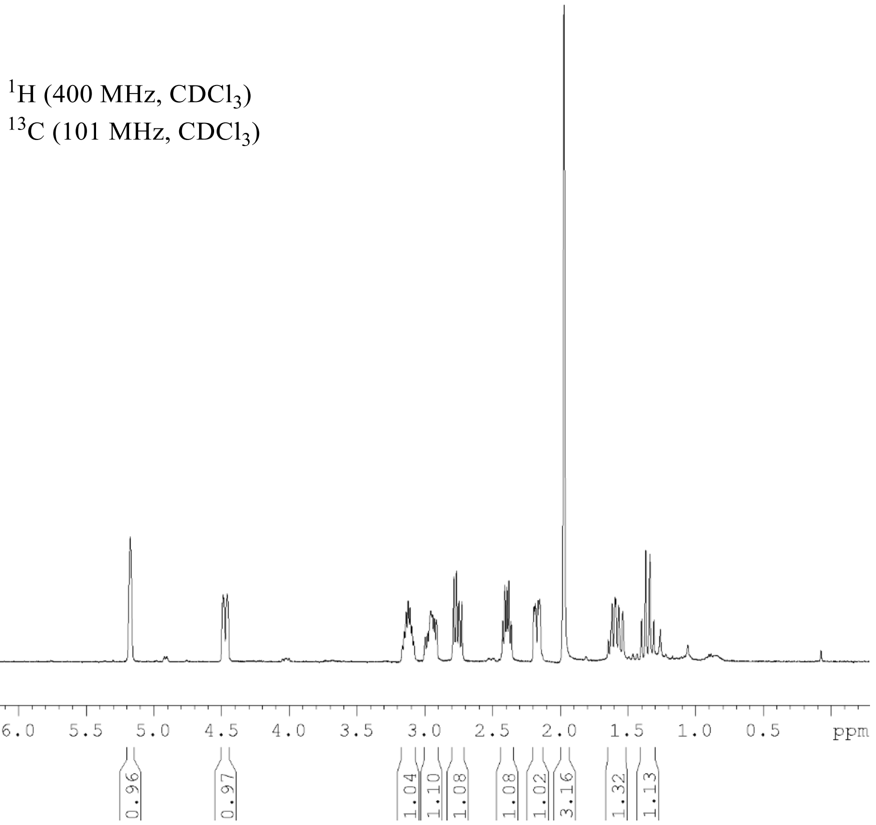

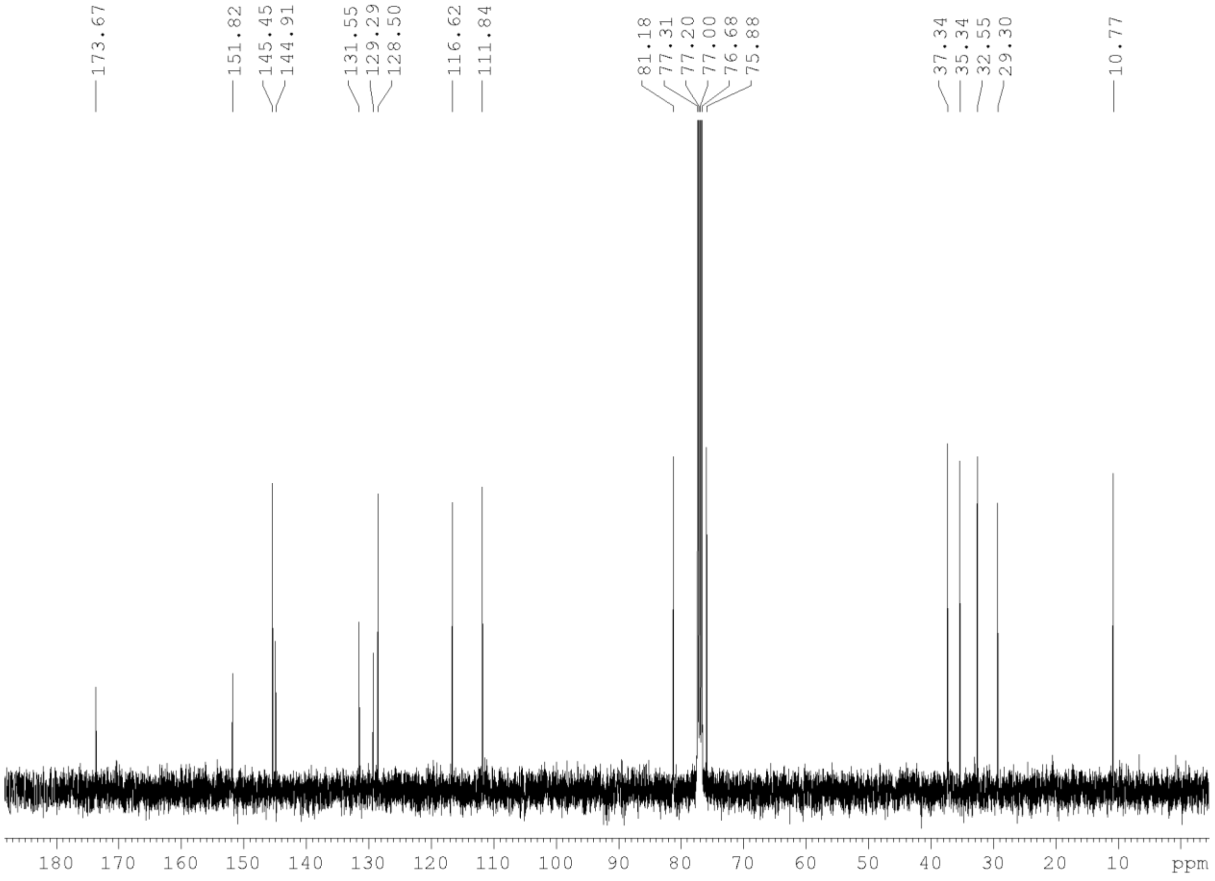

(2a*R*,3*S*)-4-Chloro-3-methyl-1,2,2a,3,4,7,8,9,10,11-decahydrocyclohepta[*g*]cyclopenta[*de*]chromene, 80

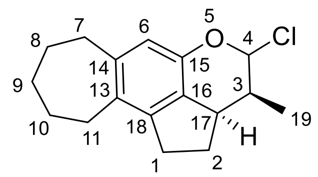

^{1}H (400 MHz, $CDCl_3$)
^{13}C (101 MHz, $CDCl_3$)

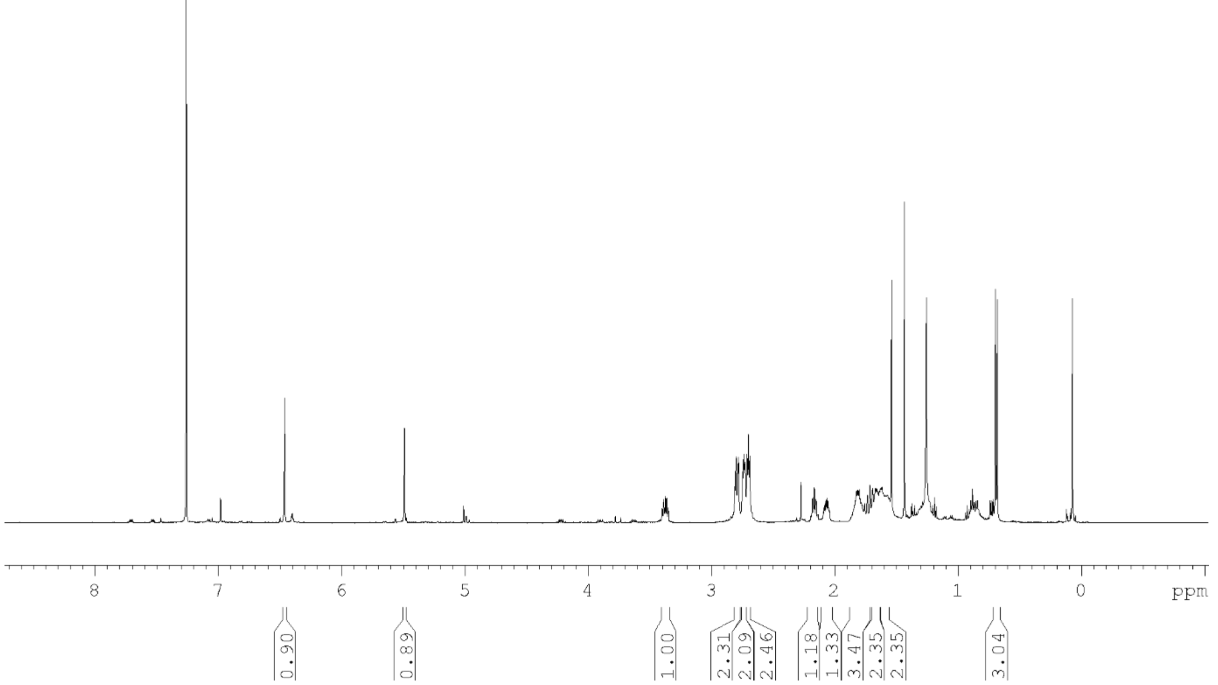

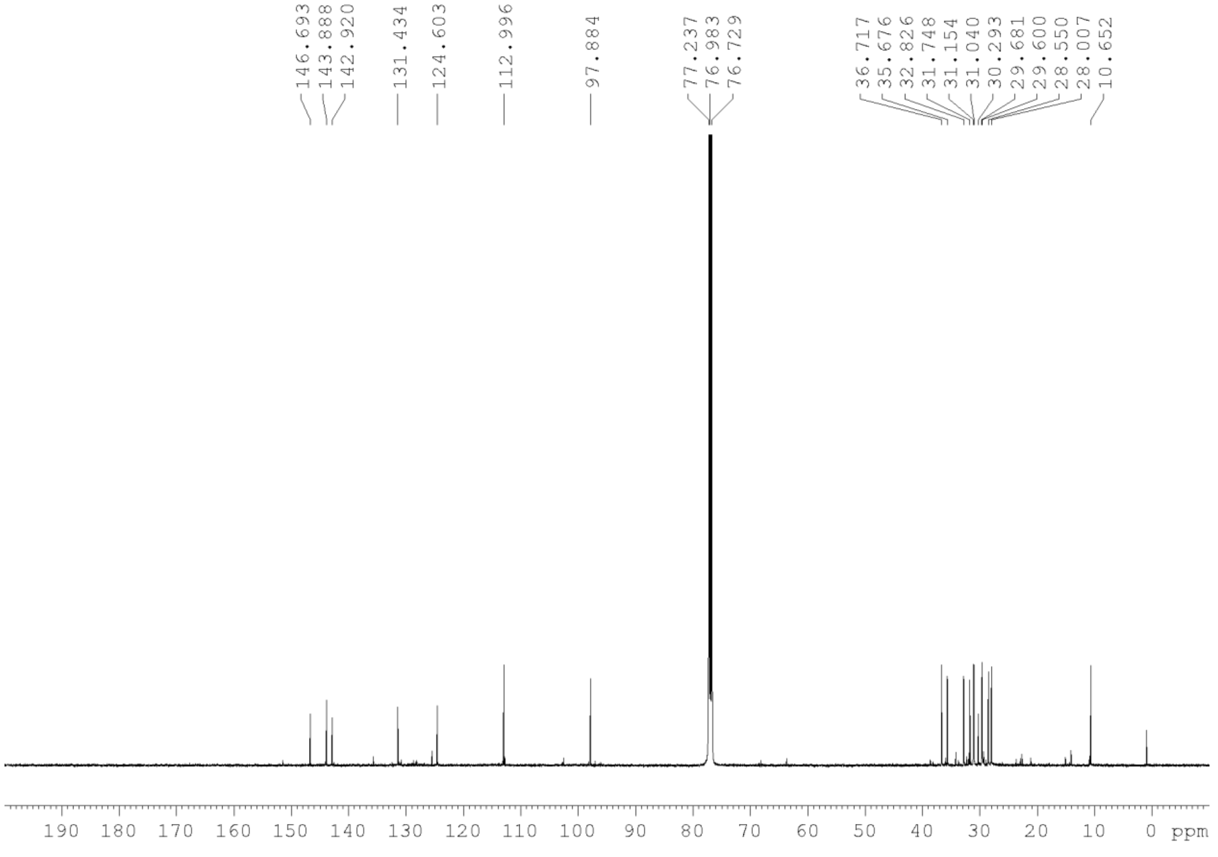

(2a*R*,2a'*R*,3*S*,3'*S*,4*R*,4'*R*)-4,4'-oxybis(3-methyl-1,2,2a,3,4,7,8,9,10,11-decahydrocyclohepta[*g*]cyclopenta[*de*]chromene), 81

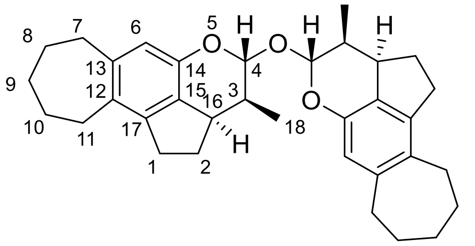

^{1}H (400 MHz, $CDCl_3$)
^{13}C (101 MHz, $CDCl_3$)

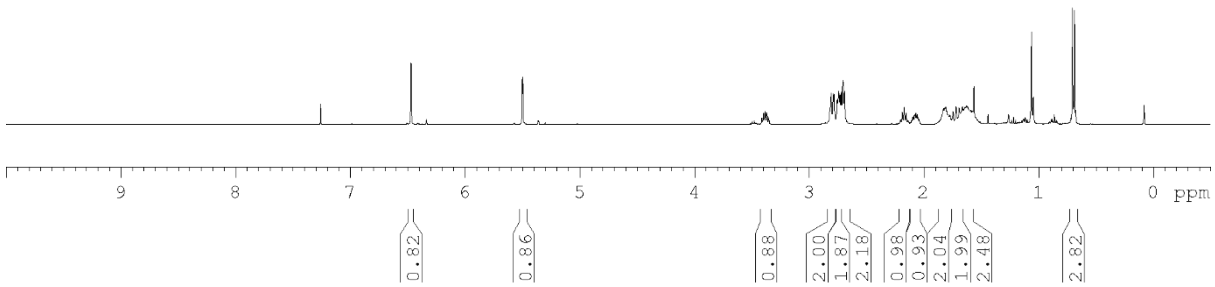

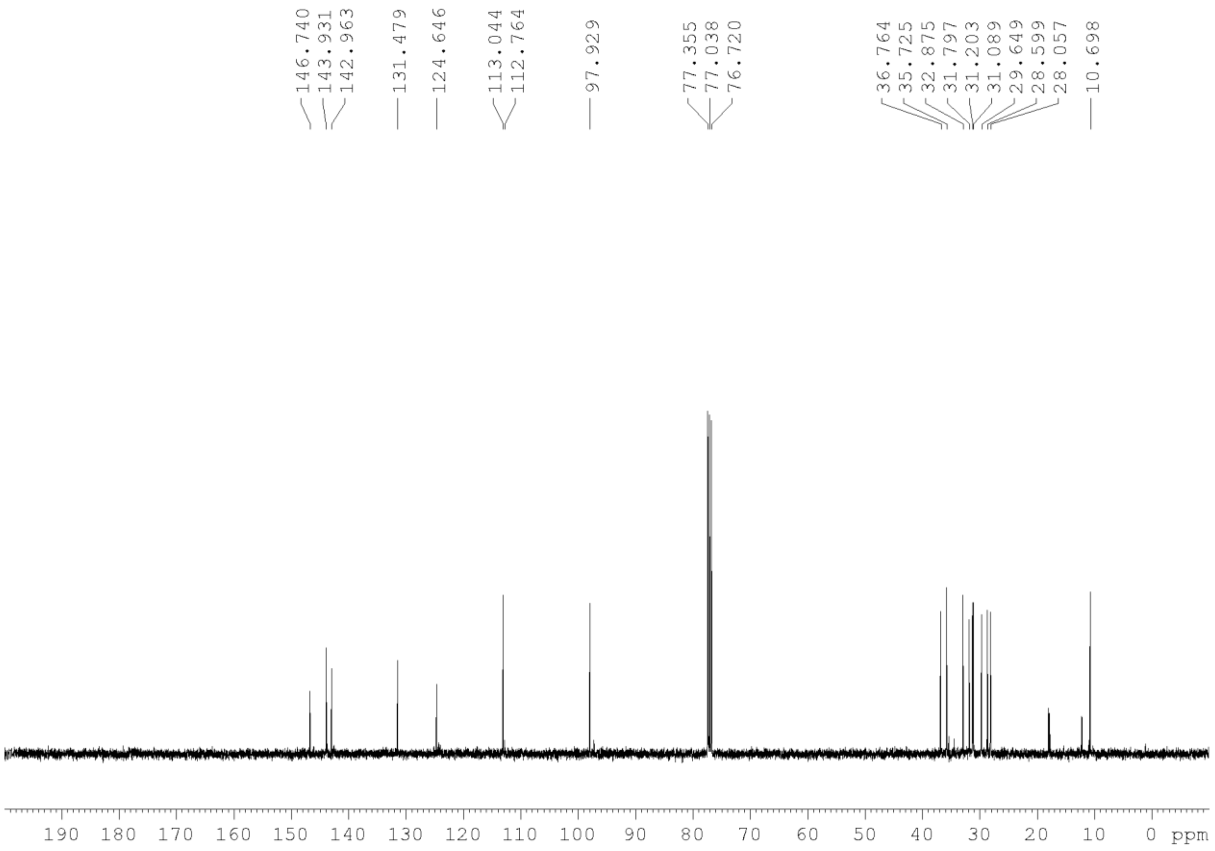

(*S*)- 3-Methyl-5-((2a*R*,3*S*,4*S*)-3-methyl-1,2,2a,3,4,7,8,9,10,11-decahydrocyclohepta[*g*]cyclopenta[*de*]-chromen-4-yl)furan-2(5*H*)-one, 82

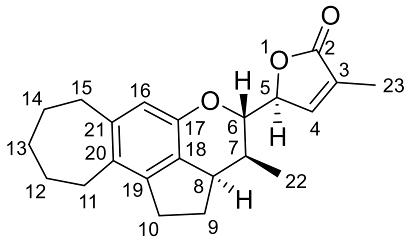

^{1}H (400 MHz, $CDCl_3$)
^{13}C (101 MHz, $CDCl_3$)

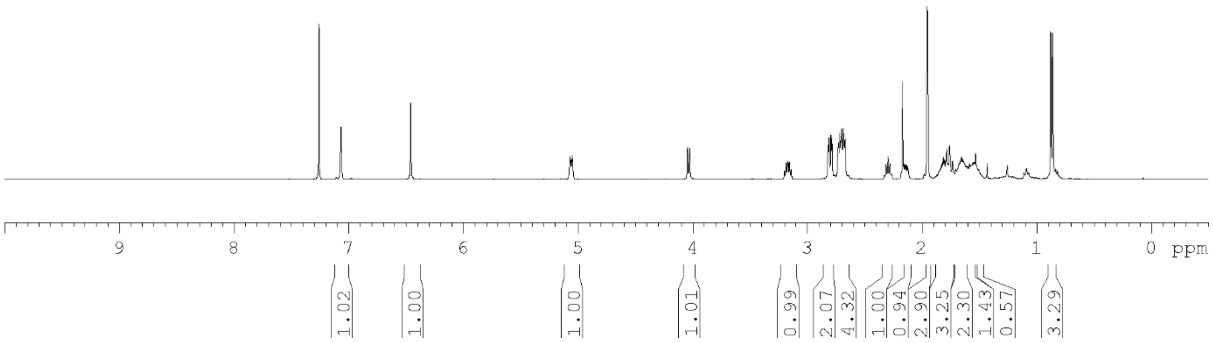

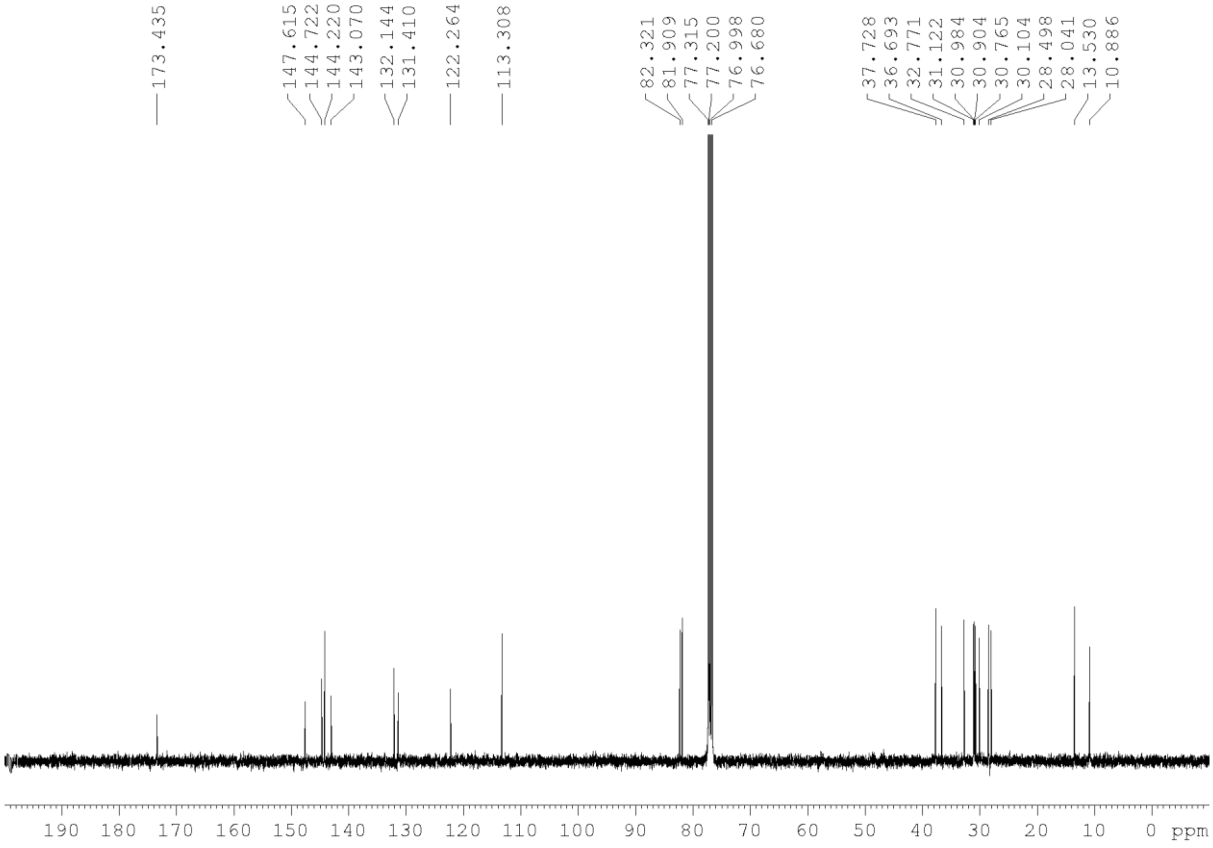

(*R*)- 3-Methyl-5-((2a*R*,3*S*,4*S*)-3-methyl-1,2,2a,3,4,7,8,9,10,11-decahydrocyclohepta[*g*]cyclopenta[*de*]-chromen-4-yl)furan-2(5*H*)-one, 83

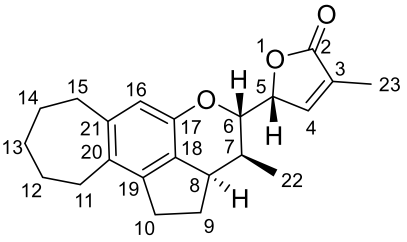

^{1}H (400 MHz, $CDCl_3$)
^{13}C (101 MHz, $CDCl_3$)

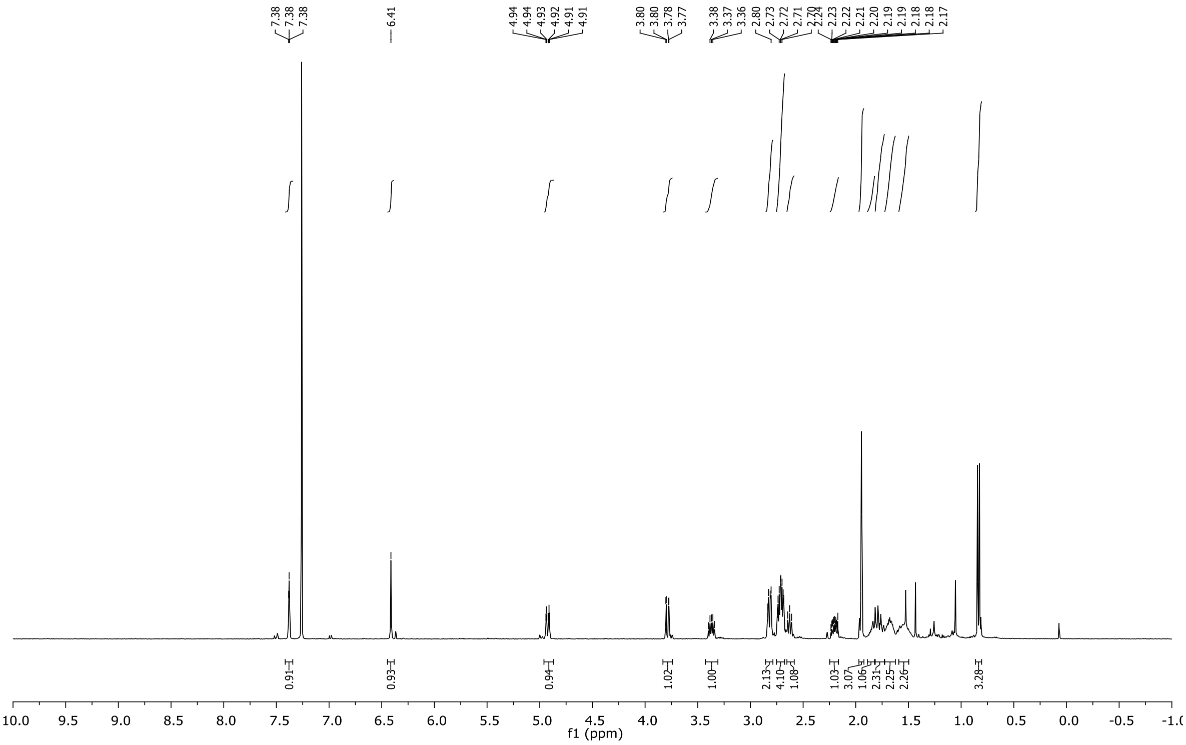

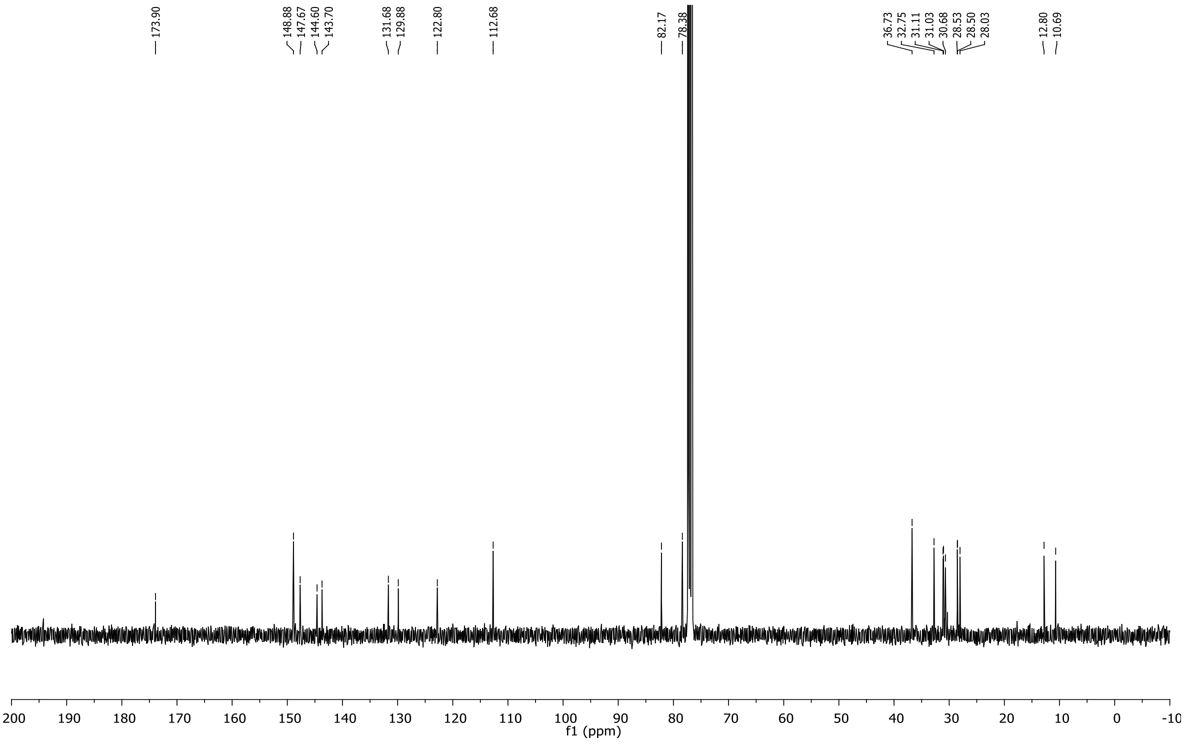

Spectra for rubriflordilactone A and C23-epi- rubriflordilactone A

Rubriflordilactone A, 2

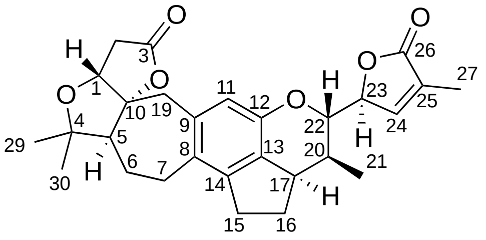

2

^{1}H NMR (500 MHz, pyridine-d_5)

^{13}C NMR (125 MHz, pyridine-d_5)

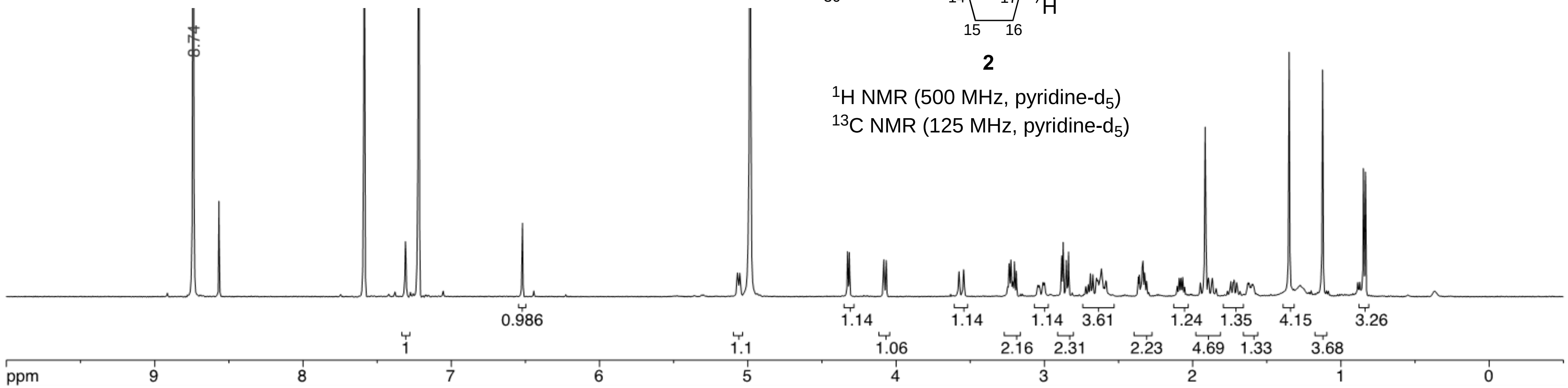

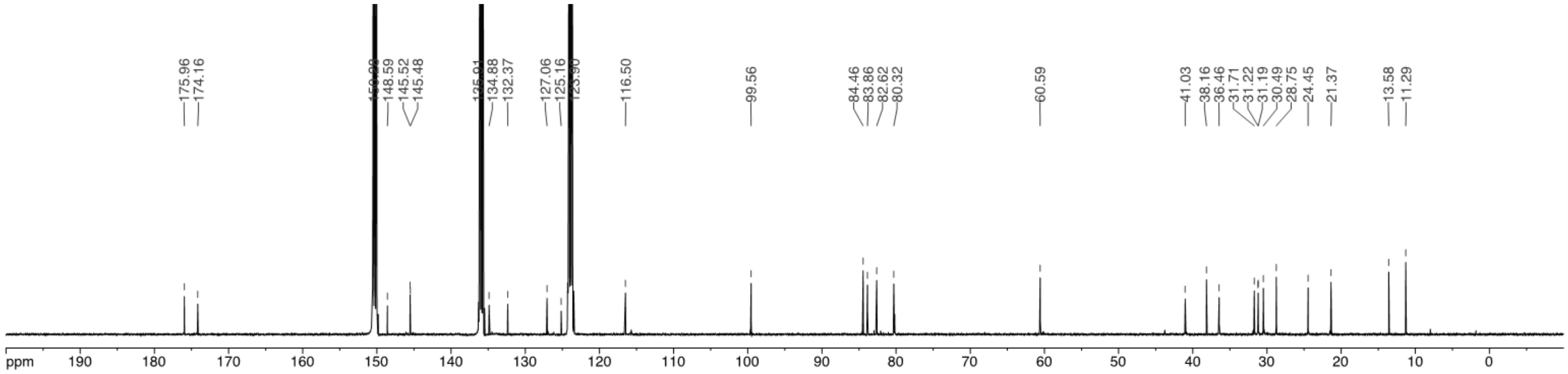

Rubriflordilactone A, HSQC spectrum (500 MHz, py). *This allows assignment of methylene pairs at H15, H16 and H7; and methines at H5, H20.*

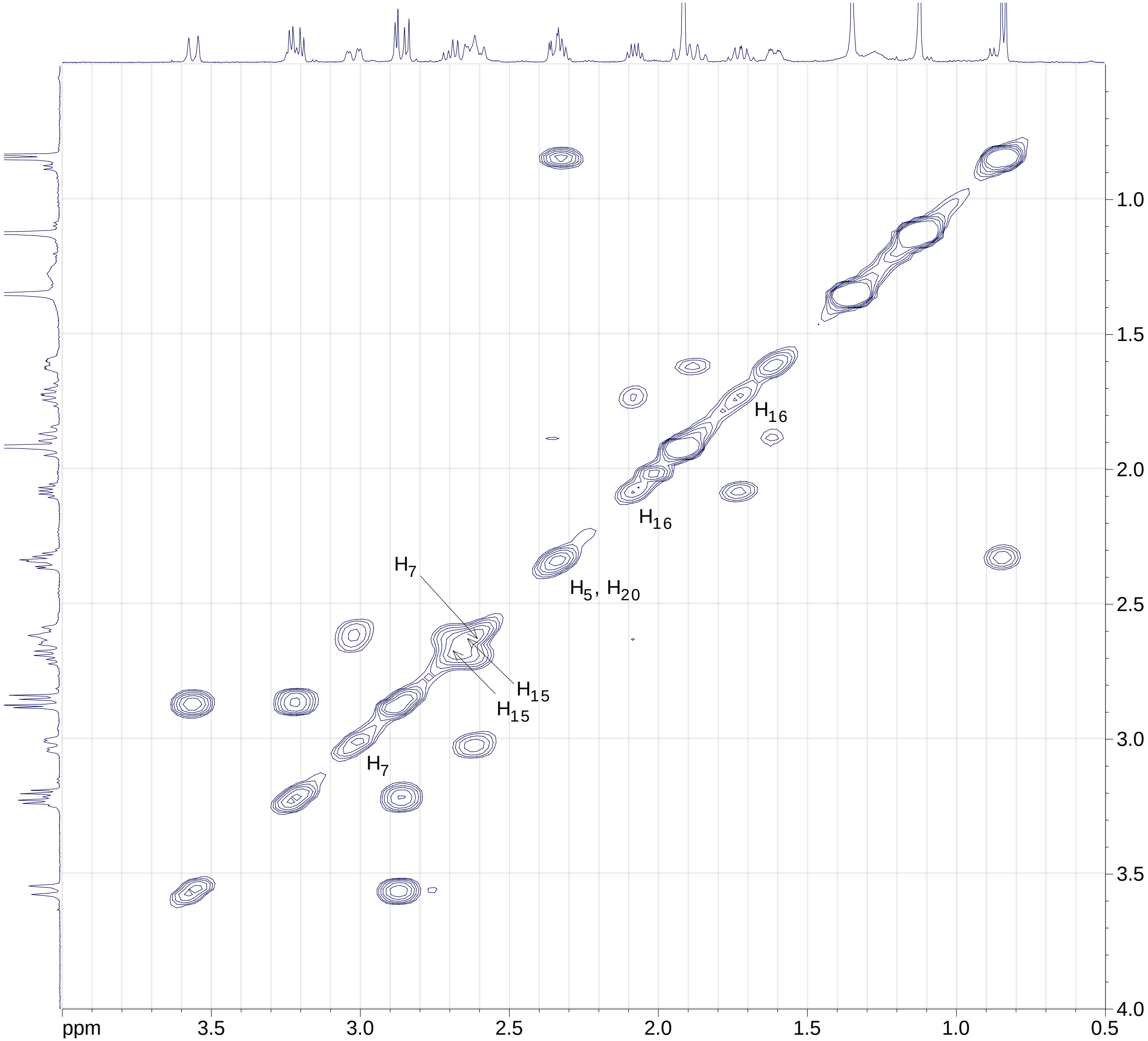

Rubriflordilactone A, HSQC spectrum (500 MHz, py). *This allows reassignment of 15α, 15β, 16α, 16β by correlations with C15 and C16.*

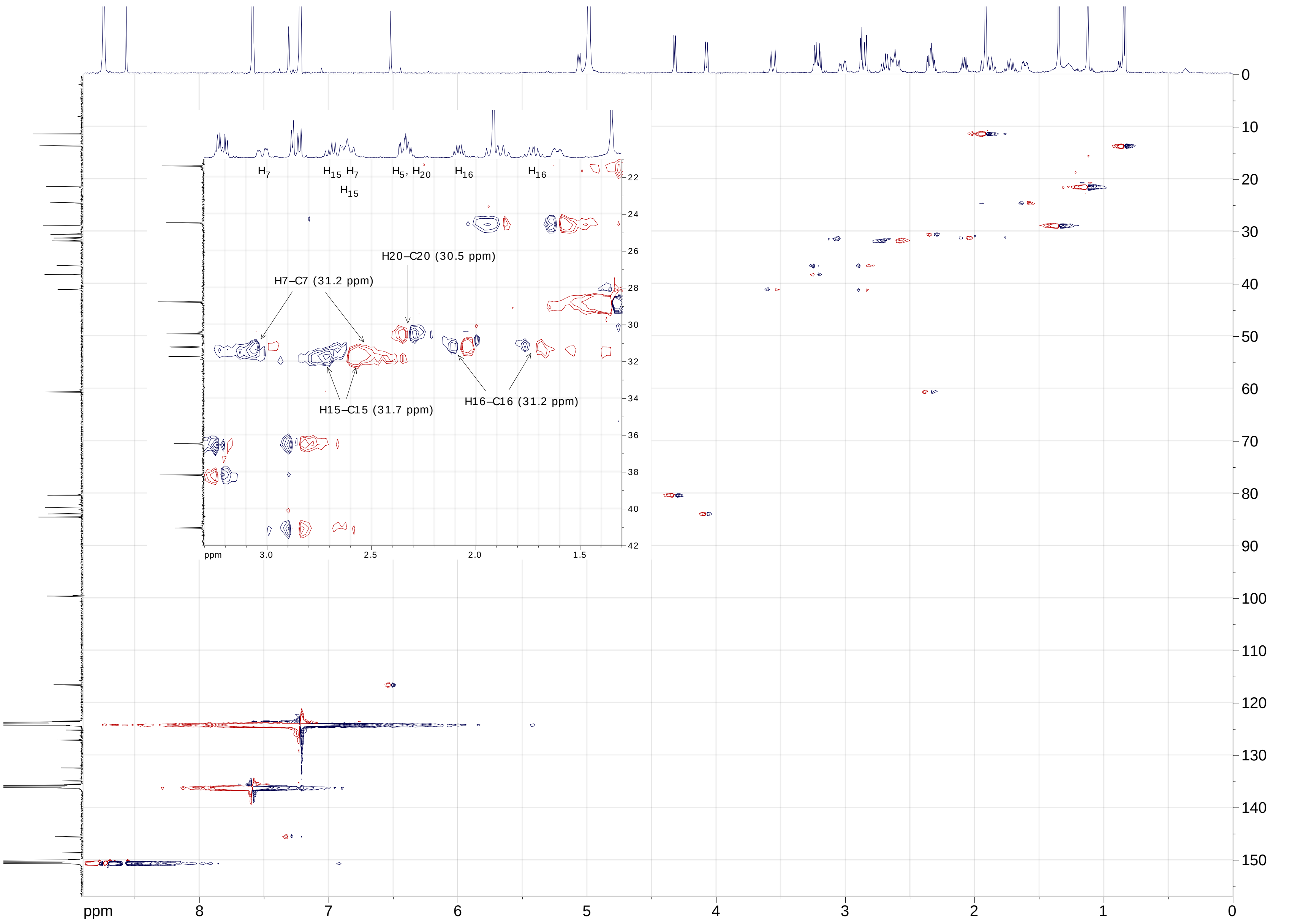

C23-*epi*-Rubriflordilactone A, 94 Note: this purified compound contains a small quantity of an additional inseparable diastereomer (at C22).

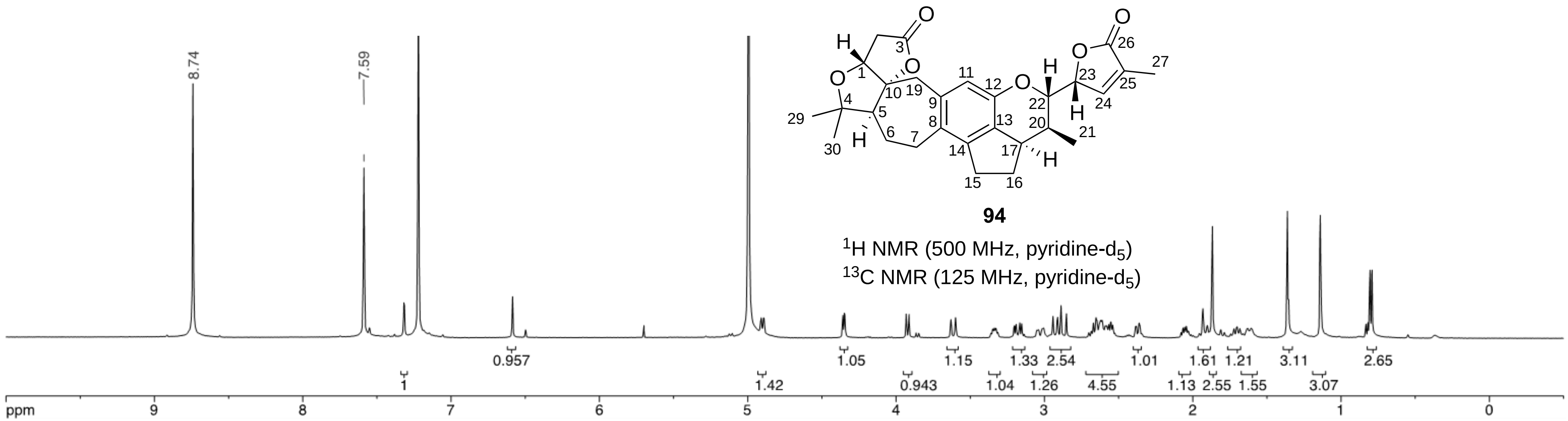

94

^{1}H NMR (500 MHz, pyridine-d_5)

^{13}C NMR (125 MHz, pyridine-d_5)

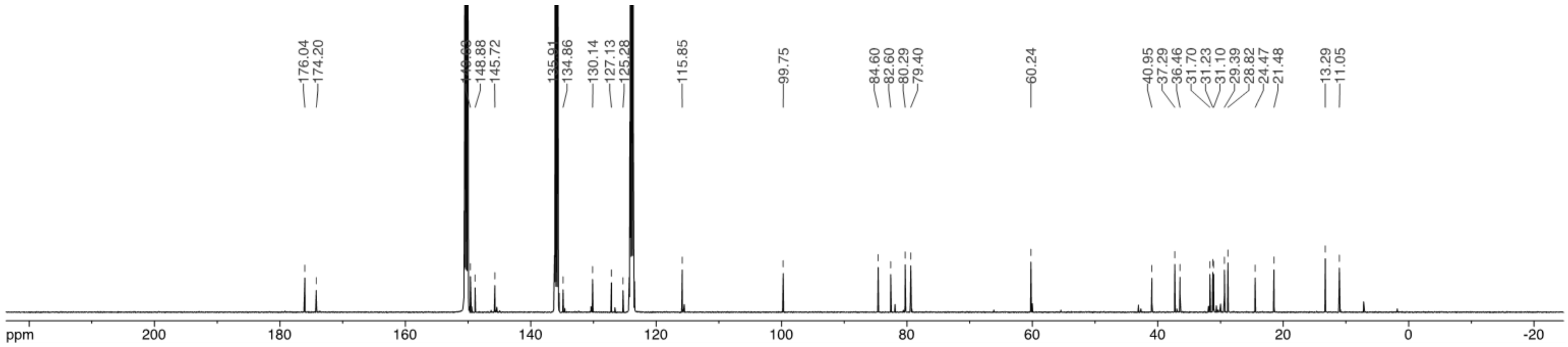

Supplement: Supplementary file 1 — Supplementary [file CHEM-23-14080-s001.pdf]
